# Efficacy of traditional Chinese medicine injection for diabetic kidney disease: A network meta analysis and systematic review

**DOI:** 10.3389/fphar.2023.1028257

**Published:** 2023-02-17

**Authors:** Caiyi Long, Haoyue Feng, Zheng Liu, Zihan Li, Jun Liu, Yayi Jiang, Rensong Yue

**Affiliations:** ^1^ Hospital of Chengdu University of Traditional Chinese Medicine, Chengdu, China; ^2^ Chengdu University of Traditional Chinese Medicine, Chengdu, China; ^3^ People’s Hospital of Nanjiang County, Bazhong, China

**Keywords:** traditional medicine, diabetic kidney disease, randomized controlled trial, network meta-analysis, injections

## Abstract

**Background:** Diabetic kidney disease (DKD) is an important public health problem worldwide that increases the mortality of patients and incurs high medical costs. Traditional Chinese Medicine injections (TCMIs) are widely used in clinical practice. However, their efficacy is unknown owing to a lack of definitive evidence. This study conducted a network meta-analysis (NMA) to evaluate the efficacy and safety of traditional Chinese medicine injections in the treatment of DKD to provide a reference for clinical treatment.

**Methods:** Total 7 databases had been searched, which included PubMed, Embase, Cochrane Library, Web of Science, China National Knowledge Infrastructure (CNKI), Chinese scientific journal database (VIP), WanFang, and SinoMed. Only randomised controlled trials (RCT) had been included for analysis. The retrieval time limit was from the establishment of the database until 20 July 2022. Cochrane Risk of Bias 2.0 tool was used to evaluate the quality of the studies. Network meta-analyses, and Trial Sequential Analyses (TSA) were used to analysis the effectiveness of the included RCTs for DKD. The Stata 15.1 and R 4.0.4 were used to perform the network meta-analysis. Sensitivity analysis was used to assess the robustness of the findings. The effect of the intervention evidence are summarized on the basis of the minimum background framework.

**Results:** NMA showed that the total effective rate of SMI, DCI, DHI, HQI, and SKI combined with alprostadil injection (PGE1) was better than PGE1 single used. Based on the surface under the cumulative ranking curve values, PGE1+DHI was the most effective for urinary albumin excretion rate and 24 h urinary albumin, PGE1+HQI was the most effective for the total response rate and β2-MG, and PGE1+SKI was the most effective for serum creatinine and blood urea nitrogen. Cluster analysis found that PGE1+HQI and PGE1+SKI could be the best treatments in terms of primary outcome measures. PGE1+SKI was found to be most effective on glomerular filtration function. PGE1+DHI was most effective for urinary protein-related indices.

**Conclusion:** The efficacy of TCMI combined with PGE1 was higher than PGE1 single used. PGE1+HQI and PGE1+SKI were the most effective treatments. The safety of TCMI treatment should be investigated further. This study needs to be validated using large-sample, double-blind, multicentre RCTs.

**Systematic Review Registration**: [https://www.crd.york.ac.uk/prospero/display_record.php?RecordID=348333], identifier [CRD42022348333].

## 1 Introduction

Diabetic kidney disease (DKD) is one of the most serious microvascular complications of diabetes and has become a global public health challenge. 10.5% of adults have diabetes (Sun et al., 2022), and 40% of them developed into DKD (Alicic et al., 2017). This situation causing a heavy socioeconomic burden (de Boer et al., 2011; Afkarian et al., 2016; Kramer et al., 2018; Kume et al., 2019). DKD caused worse prognosis and increased risk of death in diabetic patients (Groop et al., 2009; Fox et al., 2012; Penno et al., 2018; Skupien et al., 2019). Preventing and delaying DKD progression is important in disease management for diabetes patients.

Currently, the main treatment methods for DKD are renin-angiotensin-aldosterone system (RAAS) blockers to regulate blood pressure, sodium-dependent glucose transporter 2 (SGLT-2) inhibitors, intensive insulin therapy to control blood glucose and intensive life management to improve obesity (Navaneethan et al., 2021). Urinary albumin is an important indicator for the evaluation and early diagnosis of DKD; a reduction in its levels can also alleviate DKD (Foundation, 2012). Recent studies have shown that Prostaglandin E1 (PGE1) can improve insulin resistance (Wei et al., 2018), reduce proximal tubular apoptosis (Mou et al., 2018; Zhang et al., 2020), and prevent vascular, glomerular, tubular, and interstitial changes(Bersani-Amado et al., 2020). A meta-analysis showed that PGE1 may positively affect DKD by reducing the urinary albumin excretion rate (UAER) and proteinuria (Wang et al., 2010).

Modern drugs mainly focus on delaying the disease process; hence, reversing DKD is a challenge and many new drugs are not approved for patients with an eGFR <30 mL/min. Traditional Chinese medicine (TCM) is widely used in the clinical prevention and treatment of DKD in China and has synergistic effects and safety advantages. The specific chemical mechanism of DKD protection by Chinese herbal medicine has been reviewed (Tang et al., 2021), which includes anti-inflammatory and antioxidant effects, inhibition of mesangial cell expansion, and reduction of podocyte injury (Xue et al., 2019; Zhong et al., 2019; Yang et al., 2022). Traditional Chinese medicine injection (TCMI) is a patented traditional Chinese drug registered by the National Medical Products Administration. In clinical practice, it is often combined with modern drug therapy to treat DKD. In recent years, several studies have demonstrated the efficacy of various TCMIs for the treatment of DKD (Yin et al., 2014; Liao et al., 2017; Wang et al., 2021; Xie et al., 2021).

The specific efficacies and therapeutic advantages of TCMIs are unclear, which causes clinical application problems. This study is the first article to systematically evaluate and compare the clinical efficacies, laboratory indicators, and safety of several commonly used TCMIs in combination with PGE1. The purpose of this study was to provide sufficient clinical evidence for TCM medicine and to provide a reference for the clinical use of TCMIs in the treatment of DKD.

## 2 Materials and methods

### 2.1 Standard evaluation of traditional Chinese medicine

In order to make the study more accurate and reproducible, this study reported traditional Chinese medicine injections by referring to The ConPhyMP consensus (Heinrich et al., 2022). At the same time, we standardized the scientific names of botanical drug components with reference to Rivera et al. (2014). And validated in the databases of “Plant of the World Online” (http://www.plantsoftheworldonline.org) and “The World Flora Online” (WFO, http://www.worldfloraonline.org/). Summary tables describing the composition of agents and how they were reported in the original study were prepared in accordance with the principles described in the four pillars of ethnopharmacology. The composition and standard name of each injection are shown in Table 1. Other details are shown in Supplementary Tables S12, S13 (page 142–147).

**TABLE 1:** Composition of the traditional Chinese medicine injections.

Drug name	Botanical plant names	Species	Plant parts used
Danshen injection	*Salvia miltiorrhiza Bunge*	*Lamiaceae*	*Salviae miltiorrhizae radix et rhizoma*
Danshen-Chuanxiongqin injection	*Salvia miltiorrhiza Bunge*	*Lamiaceae*	*Salviae miltiorrhizae radix et rhizoma*
	*Ligustrazine*	—	—
Danhong injection	*Salvia miltiorrhiza Bunge*	*Lamiaceae*	*Salviae miltiorrhizae radix et rhizoma*
	*Carthamus tinctorius L.*	*Asteraceae*	*Carthamus tinctorius L. flower buds*
Huangqi injection	*Astragalus mongholicus Bunge*	*Fabaceae*	*Astragalus mongholicus Bunge radix et rhizoma*
Shenkang injection	*Salvia miltiorrhiza Bunge*	*Lamiaceae*	*Salviae miltiorrhizae radix et rhizoma*
	*Astragalus mongholicus Bunge*	*Fabaceae*	*Astragalus mongholicus Bunge radix et rhizoma*
	*Carthamus tinctorius L.*	*Asteraceae*	*Carthamus tinctorius L.radix et rhizoma*
	*Rheum palmatum L*	*Polygonaceae*	*Rheum palmatum L radix et rhizoma*
Shuxuetong injection	*Hirudo*	*Hirudinidae*	—
	*Pheretima*	*Megascolecidae*	—
Xuebijing injection	*Carthamus tinctorius L.*	*Asteraceae*	*Carthamus tinctorius L. flower buds*
	*Salvia miltiorrhiza Bunge*	*Lamiaceae*	*Salviae miltiorrhizae radix et rhizoma*
	*Angelica sinensis (Oliv.) Diels*	*Apiaceae*	*Angelica sinensis (Oliv.) Diels radix et rhizoma*
	*Paeonia lactiflora Pall.*	*Paeoniaceae*	*Paeonia lactiflora Pall. radix et rhizoma*
	*Ligusticum chuanxiong Hort.*	*Apiaceae*	*Ligusticum chuanxiong Hort. et rhizoma*

### 2.2 Systematic review protocol and registration

The network meta-analysis was registered with the International Prospective Register of Systematic Reviews (PROSPERO) under the registration number CRD42022348333. We followed the Preferred Reporting Items for Systematic Reviews and Meta-Analyses (PRISMA), its protocols, and the PRISMA-extension statement for network meta-analysis to report the current results (Shamseer et al., 2015; Page et al., 2021).

### 2.3 Literature search

This study searched PubMed, Embase, Cochrane Library, CNKI, VIP, Wanfang, and SinoMed databases, total 7 databases. The main search terms included “injection*,” “Diabetic Nephropathies,” “Nephropathies, Diabetic,” “Nephropathy, Diabetic,” “Diabetic Kidney Disease,” “Kidney Disease, Diabetic,” “Alprostadil,” “PGE1alpha,” “Prostaglandin E1alpha,” “PGE1,” “Lipo-PGE1” and others. References from previous systematic reviews and meta-analyses with similar topics were scanned for supplementation in the preliminary screening stage, references from eligible articles were scanned for supplementation in the full-text screening stage, and unpublished studies were not retrieved. The detailed search strategy is presented in Supplementary Tables S3–S10 (page 131–136). The retrieval time for each database was from database construction until 20 July 2022.

### 2.4 Inclusion and exclusion criteria

Inclusion criteria were determined based on PICO: (a) type of included studies: randomised controlled trials (RCTs); (b) patients: the subjects of the study were those who met the requirements of the DKD diagnostic criteria—no limitations existed in age, sex, or nationality; (c) interventions: in the treatment group, the intervention was TCMI + PGE1, which could be combined with conventional treatment (including the control of blood glucose, blood pressure, and blood lipids). The control group was treated with PGE1 in combination with conventional treatment; (d) outcome measures: the primary outcomes in this study were total effective rate (the calculation formula was as follows: total effective rate = marked effective rate + effective rate, markedly effective was defined as the main symptoms disappeared, and at least 50% reduction in the urine protein, or blood urea nitrogen (BUN) returned to normal, a decrease in at least 88.4 mmol L^−1^ of serum creatinine (Scr). Effective treatment showed that the main clinical symptoms were improved, the degree of urinary protein reduction was more than 33.3%, and BUN and Scr were decreased. The secondary outcomes included UAER, BUN, 24 h urinary albumin (24 h Alb), and urinary β2-microglobulin (β2-MG) levels. Studies that included only one outcome measure were eligible for inclusion. (e) The number of papers on the same TCMI should be greater than or equal to two.

The following exclusion criteria were used: (a) repeated articles; (b) incomplete or incorrect data; (c) non-conforming studies (including reviews, systematic reviews, meta-analyses, animal experiments, conference abstracts, reports, letters, case reports, etc.).

### 2.5 Study selection and data extraction

Two researchers (CYL and HYF) from related disciplines independently screened and crosschecked for inclusion. In the case of disagreement, a third researcher (RSY) can judge and provide a solution. Preliminary screening was carried out according to the title and abstract, and the included studies were then determined by reading the full text. Two researchers used uniform criteria for data extraction: the first author, year of publication, classification of DKD, duration of DM, sample size, male-to-female ratio, age, interventions, course of treatment, and outcomes.

### 2.6 Risk of bias assessment and quality assessment

The quality of the included studies was assessed by two investigators (CYL & HYF) using the Cochrane Risk of Bias 2.0 tool (Sterne et al., 2019) which included the randomisation process, deviations from intended interventions, missing outcome data, measurement of the outcome, selection of the reported result, selection of the reported result, and overall bias. The risk of bias was classified as “low risk,” “high risk,” and “some concerns.” We used the GRADE method for the entire network to provide a framework for the deterministic rating of each paired comparison evidence, divided into high, medium, low or very low (Puhan et al., 2014; Brignardello-Petersen et al., 2019).

### 2.7 Statistical analysis

This study used R4.0.4 and Stata15.1 software to calculate and draw graphs. For binary results, the combined results were calculated as odds ratio (OR). For continuous outcomes, this study used mean differences (MD), and standardised mean differences (SMD) were used when data units were inconsistent. All results are shown with 95% confidence intervals (95% CI). The league table was calculated using the Markov chain Monte Carlo method of the random-effects model through R4.0.4. The number of iterations was set to 200,000, and the first 100,000 iterations were used in the annealing algorithm to eliminate the influence of the initial values. Network diagrams were constructed using the Stata software to compare different interventions. Surface under the cumulative ranking curve (SUCRA) probability values were used to rank the detected treatments, with SUCRA values of 100% and 0% assigned to the best and worst treatments, respectively. Cluster analysis was used to compare the efficacy of TCMIs for different functions. The minimal contextualization framework was developed based on the results of SUCRA and GRADE assessments (Brignardello-Petersen et al., 2020). The bias information criterion was used to compare the fit of consistent and inconsistent models, and Cochran’s *I* ^*2*^ statistic was used to assess statistical heterogeneity, with low, medium, and high *I* ^*2*^ values of 25, 50, and 75%, respectively (Higgins et al., 2003). Funnel plots were used to detect publication bias in the primary outcome measures. Sensitivity analyses were carried out by excluding studies with a high-risk bias and those with courses of treatment that did not fall within 14–30 days. According to the information collected so far, TSA version 0.9 beta was used to calculate and draw the required information size and trial sequential monitoring boundaries.

## 3 Results

### 3.1 Study selection and characteristics

**FIGURE 1:**
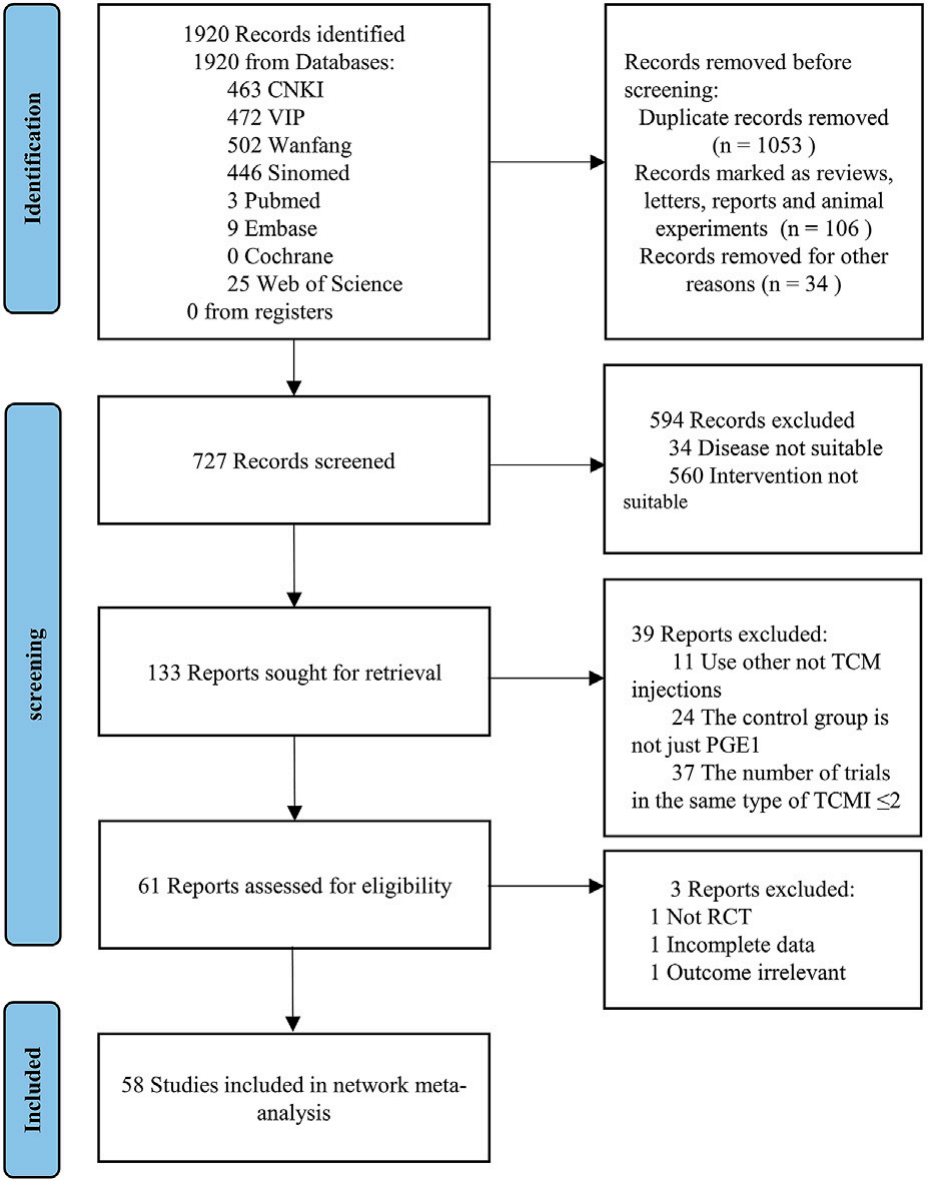
Study flow diagram.

A total of 1920 studies were initially identified from the search, and 727 studies were retained after excluding duplicate literature, animal experiments, meetings, reports, and letters by screening titles and abstracts. After reading the titles and abstracts of the 727 studies, 34 studies in which disease was not suitable and 580 studies in which intervention was not suitable (including 184 studies with only PGE1 without comparison, 25 studies not combining PGE1, and 351 studies not combining TCMIs) were excluded, and 133 studies were retained. After reading the full text of the remaining literature, 11 studies without TCMI, 24 not only used PGE1 in the control group, and 37 studies in which the number of studies with the same type of TCMI ≤2 were excluded. Among the remaining 61 studies, one without an after-before control, one with incomplete data, and one with irrelevant outcome indicators from the input data were excluded. Finally, 58 studies from 2002 to 2022 were retained (Xie and Zhang, 2002; Ru et al., 2008b; Gong and Xie, 2009; Wang et al., 2009; Min et al., 2010; Zhao and Dong, 2010; Pang et al., 2011; Wu, 2011; Xing, 2012; Zhou and Lai, 2012a; b; Han and Zhang, 2013; Lin, 2013; Liu, 2013; Pu et al., 2013; Zhang et al., 2013; Ding, 2014; Lan, 2014; Mei et al., 2014; Wang et al., 2014; Yin, 2014; Zhang, 2014; Zhou et al., 2014; Cai et al., 2015; He, 2015; Li and Li, 2015; Liu and Guo, 2015; Yang et al., 2015; Cao et al., 2016; Fang et al., 2016; Li et al., 2016; Liu et al., 2016; Liu and Sun, 2016; Zhang, 2016; Zhang and Peng, 2016; Cui et al., 2017; Jiang and Qu, 2017; Liu, 2017; Mai et al., 2017; Zhang, 2017b; Zhang, 2017a; Zhong, 2017; Chen and Fu, 2018; Jia, 2018; Liang, 2018; Shen, 2018; Su, 2018; Tian et al., 2018; Wang, 2018; Xu and Liu, 2018; Ye, 2018; Zheng et al., 2018; Lin et al., 2019; Wang et al., 2019; Xiao, 2019; Zhang and Nan, 2019; Wang, 2021; Wen et al., 2021; Nie, 2022). The specific screening process is shown in Figure 1.

**FIGURE 2:**
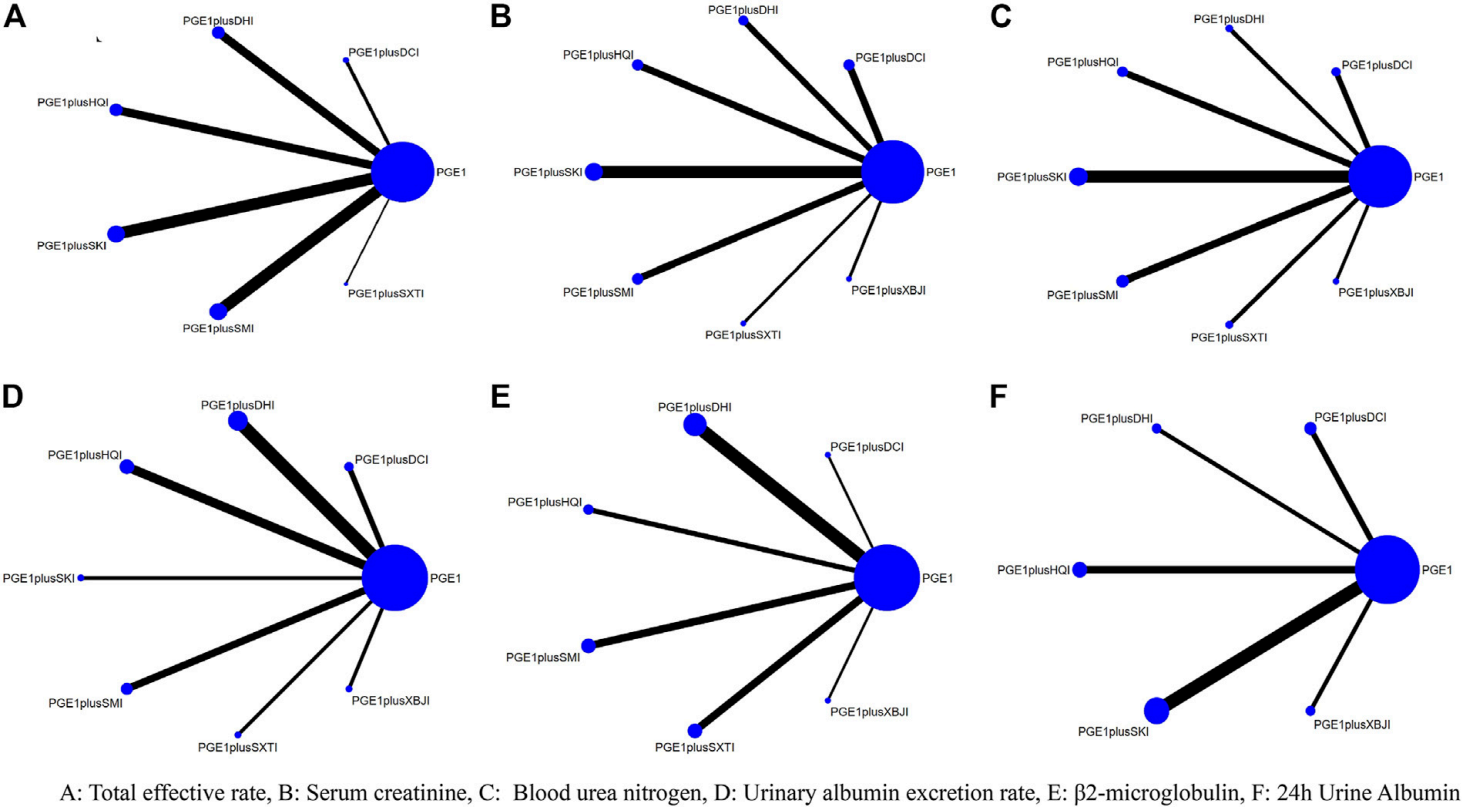
Network diagrams for different outcomes.

58 articles included in this study included a total of 4808 subjects and seven types of TCMIs, namely *Salvia miltiorrhiza* injection (SMI; 12 RCTS) (Zhou and Lai, 2012a; Han and Zhang, 2013; Wang et al., 2014; Cai et al., 2015; He, 2015; Liu and Guo, 2015; Zhang, 2017a; Liu, 2017; Zhong, 2017; Chen and Fu, 2018; Liang, 2018; Wang, 2018), Danshen-Chuanxiongqin injection (DCI; 7 RCTs) (Yang et al., 2015; Zhang and Peng, 2016; Cui et al., 2017; Mai et al., 2017; Shen, 2018; Su, 2018; Wang, 2021), Danhong injection (DHI; 8 RCTs) (Min et al., 2010; Pu et al., 2013; Li and Li, 2015; Liu et al., 2016; Liu and Sun, 2016; Jia, 2018; Lin et al., 2019; Xiao, 2019), Huangqi injection (HQI; 9 RCTs) (Xie and Zhang, 2002; Zhao and Dong, 2010; Wu, 2011; Lin, 2013; Liu, 2013; Zhou et al., 2014; Fang et al., 2016; Zhang, 2016; Xu and Liu, 2018), Shenkang injection (SKI;15 RCTs) (Wang et al., 2009; Pang et al., 2011; Zhang et al., 2013; Ding, 2014; Lan, 2014; Mei et al., 2014; Yin, 2014; Zhang, 2014; Zhang, 2017b; Ye, 2018; Zheng et al., 2018; Wang et al., 2019; Zhang and Nan, 2019; Wen et al., 2021; Nie, 2022), Shuxuetong injection (SXTI; 4 RCTs) (Ru et al., 2008a; Gong and Xie, 2009; Xing, 2012; Tian et al., 2018), and Xuebijing injection (XBJI; 3 RCTs) (Cao et al., 2016; Li et al., 2016; Jiang and Qu, 2017). The course of treatment ranged from 7 days to 3 months. The basic characteristics are shown in Table 2 and the comparative associations between each intervention and each outcome measure are shown in Figure 2. In addition, we collected the specific intervention method of each included study (Supplementary Table S14, page148–153).

**TABLE 2:** Characteristics of the studies included in this network meta-analysis.

Study	Classification of DKD	Duration of DM		Sample size			Sex(M/F)		Age (mean or range)		Interventions		Course of treatment	Outcomes
		T	C	T	C	T		C	T	C	T	C		
Zhoufangmin (2012)	NA	NA	NA	60	60	37/23		35/25	60.2 ± 9.4	58.6 ± 8.7	PGE1 (4 mL/d) + SMI(20 mL/d)	PGE1 (4 mL/d)	28d	②③④⑤
Hanpeng (2013)	III-IV	8.25 ± 0.44		40	40	44/36			55.92 ± 11.40		PGE1 (2 mL/d) + SMI(1 mL/d)	PGE1 (2 mL/d)	15d	①
Wangqun (2014)	III	NA	NA	16	18	32/21a			56.3 ± 16.8		PGE1 (4 mL/d) + SMI (20 mL/d)	PGE1 (4 mL/d)	14d	②③⑤
Caiwenting (2015)	NA	10.7 ± 3.4	9.7 ± 3.4	40	40	24/16		22/18	58 ± 9.7	53.6 ± 4.7	PGE1 (4 mL/d) + SMI (20 mL/d)	PGE1 (4 mL/d)	28d	②③④⑤
Hemingwu (2015)	NA	6–13	7–14	40	40	21/19		18/22	53.2 ± 7.6	51.7 ± 8.4	PGE1 (4 mL/d) + SMI (20 mL/d)	PGE1 (4 mL/d)	28d	①
Liugang (2015)	early stage	NA	NA	52	52	28/24		30/22	59.33 ± 5.16	60.25 ± 4.94	PGE1 (4 mL/d) + SMI (20 mL/d)	PGE1 (4 mL/d)	30d	①②③④
Liuying (2017)	early stage	NA	NA	29	29	14/15		15/14	58.65 ± 6.23	59.36 ± 6.54	PGE1 (4 mL/d) + SMI (20 mL/d)	PGE1 (4 mL/d)	30d	①
Zhangyarong (2017)	early stage	NA	NA	43	43	51/35			48.21 ± 4.67		PGE1 (4 mL/d) + SMI (20 mL/d)	PGE1 (4 mL/d)	28d	①③
Zhongchao (2017)	III	9.2 ± 1.1	9.3 ± 1.2	47	47	28/19		31/16	62.21 ± 2.21	63.34 ± 2.18	PGE1 (4 mL/d) + SMI (20 mL/d)	PGE1 (2 mL/d)	28d	①
Chenxuan (2018)	III	NA	NA	49	49	29/20		27/22	61.5 ± 2.0	60.2 ± 4.5	PGE1 (2 mL/d) + SMI (20 mL/d)	PGE1 (2 mL/d)	28d	②③④
Liangliang (2018)	NA	NA	NA	48	48	21/27		22/26	55.7 ± 3.3	55.9 ± 2.5	PGE1(4 mL/d) + SMI (20 mL/d)	PGE1 (4 mL/d)	28d	①
Wanganwen (2018)	I-III	8.38 ± 4.22	8.38 ± 4.22	38	38	22/16		23/15	63.28 ± 8.45	63.28 ± 8.45	PGE1 (4 mL/d) + SMI (20 mL/d)	PGE1 (4 mL/d)	28d	②③
Yangxu (2015)	early stage	9.8 ± 1.9	9.0 ± 1.7	20	20	12/8		10/10	9.8 ± 1.9	9.0 ± 1.7	PGE1 (2 mL/d) + DCI(10 mL/d)	PGE1 (2 mL/d)	14d	②⑥
Zhangyin (2016)	NA	3.5 ± 1.4	4.2 ± 3.1	42	42	27/15		24/18	56.7 ± 0.8	56.5 ± 0.8	PGE1 (1 mL/d) + DCI(5 mL/d)	PGE1 (1 mL/d)	28d	①②③
Cuiyi (2017)	early stage	NA	NA	51	51	27/24		26/25	61.5 ± 14.2	60.3 ± 14.7	PGE1 (2 mL/d) + DCI(10 mL/d)	PGE1 (2 mL/d)	30d	②③
Maigaoyang (2017)	III	10.2 ± 4.0	10.6 ± 4.2	35	35	24/11		22/13	59.1 ± 9.3	58.2 ± 8.9	PGE1 (4 mL/d) + DCI(20 mL/d)	PGE1 (4 mL/d)	14d	②③④⑤
Shenjinsong (2018)	early stage	10.3 ± 3.9	10.2 ± 3.6	20	20	11/9		9/11	58.1 ± 9.1	57.9 ± 8.7	PGE1 (4 mL/d) + DCI(20 mL/d)	PGE1 (4 mL/d)	28d	①②⑥
Subaoting (2018)	early stage	5.21 ± 1.08	5.30 ± 1.01	44	44	26/18		24/20	60.42 ± 5.75	60.29 ± 5.90	PGE1 (2 mL/d) + DCI(10 mL/d)	PGE1 (2 mL/d)	28d	②③④
Wangjing (2021)	early stage	11.47 ± 1.66	11.65 ± 1.72	65	65	34/31		33/32	64.14 ± 3.12	64.58 ± 3.17	PGE1 (2 mL/d) + DCI(10 mL/d)	PGE1 (2 mL/d)	21d	②③④⑦
Fanmin (2010)	III	NA	NA	92	67	44/48		36/31	56–91	57–85	PGE1 (2 mL/d) + DHI(30–50 mL/d)	PGE1 (2 mL/d)	14d	②③④⑤
Puhongmei (2013)	III	NA	NA	26	26	NA		NA	NA	NA	PGE1 (2 mL/d) + DHI(20 mL/d)	PGE1 (2 mL/d)	90d	②④⑤⑦
Liqiuxia (2015)	III	11.2 ± 2.3	10.4 ± 2.6	24	24	12/12		13/11	55.7 ± 7.5	56.8 ± 7.2	PGE1 (2 mL/d) + DHI(40 mL/d)	PGE1 (2 mL/d)	14d	①④⑥
Liuqingyuan (2016)	III	13.21 ± 8.25	11.35 ± 9.18	24	21	11/13		10/11	52.10 ± 11.86	52.50 ± 12.16	PGE1 (2 mL/d) + DHI(20 mL/d)	PGE1 (2 mL/d)	14d	②③④⑤⑦
Liuzhen (2016)	III-IV	5.65 ± 1.90	5.54 ± 1.85	15	15	9/6		8/7	59.94 ± 5.21	60.24 ± 5.13	PGE1 (2 mL/d) + DHI(20–40 mL/d)	PGE1 (2 mL/d)	21d	①②③④⑤
Jiayinji (2018)	IV	8.8 ± 1.3	7.7 ± 1.5	35	36	18/17		18/18	62.7 ± 2.4	65.2 ± 2.1	PGE1 (4 mL/d) + DHI(30 mL/d)	PGE1 (4 mL/d)	28d	①②⑥
Linchenxin (2019)	NA	14.62 ± 3.05	14.58 ± 3.10	32	32	17/15		19/13	82.09 ± 4.42	82.11 ± 4.23	PGE1 (2 mL/d) + DHI(20 mL/d)	PGE1 (2 mL/d)	14d	①④⑤⑥⑦
Xiaoqianfeng (2019)	NA	7.2 ± 2.1	7.1 ± 1.8	40	40	22/18		24/16	58.3 ± 7.6	60.8 ± 8.2	PGE1(2 mL/d) + DHI(20–40 mL/d)	PGE1 (2 mL/d)	21d	①②③④⑦
Xiebinxuan (2002)	NA	3.52	3.48	20	10	12/8		7/3	64.32 ± 6.94	63.47 ± 7.11	PGE1 (20 mL/d) + HQI (40 mL/d)	PGE1 (20 mL/d)	21d	③⑥⑦
Zhaolijun (2010)	early stage	10 ± 3	8 ± 3	169	157	82/87		84/73	55 ± 12	57 ± 10	PGE1(100 mL/d) + HQI (60 mL/d)	PGE1 (100 mL/d)	14d	⑥⑦
Wuyanbo (2011)	III	NA	NA	40	40	22/18		23/17	55.3 ± 6.9	56.9 ± 7.1	PGE1(2 mL/d) + HQI (30 mL/d)	PGE1 (2 mL/d)	14d	②③④
Linjixiang (2013)	IV	NA	NA	45	45	22/23		24/21	64.1 ± 11.2	63.4 ± 10.6	PGE1(4 mL/d) + HQI (15 mL/d)	PGE1 (4 mL/d)	15d	①②⑥
Liusiyan (2013)	NA	2–13	2–11	16	17	10/6		11/6	44.6 ± 7.1	44.5 ± 7.2	PGE1(2 mL/d) + HQI (30 mL/d)	PGE1 (2 mL/d)	14d	①②③④⑦
Zhoubin (2014)	NA	NA	NA	30	30	15/15		16/14	41.2 ± 11.3	43.1 ± 9.8	PGE1(4 mL/d) + HQI (20 mL/d)	PGE1 (4 mL/d)	28d	①②③⑥
Fangwenjuan (2016)	NA	12.1 ± 2.3		40	40	47/33			61.9 ± 3.6		PGE1(2 mL/d) + HQI (30 mL/d)	PGE1 (2 mL/d)	14d	①④⑥
Zhangliang (2016)	NA	2–8	3–8	45	45	28/20		27/18	46–68	45–70	PGE1(2 mL/d) + HQI (30 mL/d)	PGE1 (2 mL/d)	28d	①②③④⑤⑦
Xufei (2018)	III	9.8 ± 2.7	10.2 ± 2.8	43	43	26/17		27/16	58.4 ± 8.5	59.2 ± 9.1	PGE1(4 mL/d) + HQI (30 mL/d)	PGE1 (4 mL/d)	28d	②③④⑤⑦
Wanghuibin (2009)	III	NA	NA	28	16	14/14		7/9	47 ± 7	45 ± 10	PGE1(2 mL/d) + SKI(100 mL/d)	PGE1 (2 mL/d)	14d	②③⑥⑦
Pangjialiang (2011)	III	8.1 ± 4.6	8.3 ± 5.1	30	30	16/14		17/13	53.5 ± 7.3	52.2 ± 6.8	PGE1(2 mL/d) + SKI(60 mL/d)	PGE1 (2 mL/d)	14d	⑦
Zhanghong (2013)	NA	3.45 ± 0.76		30	30	14/16		17/13	53.73	52.41	PGE1(2 mL/d) + SKI(100 mL/d)	PGE1 (2 mL/d)	28d	②③⑥
Dingxuemei (2014)	III-IV	NA	NA	36	34	NA		NA	NA	NA	PGE1(2 mL/d) + SKI(100 mL/d)	PGE1 (2 mL/d)	28d	②③⑦
Lanchunying (2014)	III-IV	10.0 ± 3.0		40	40	19/21		22/18	55.0 ± 6.0		PGE1 (2 mL/d) + SKI(60 mL/d)	PGE1 (2 mL/d)	28d	①②③⑥
Meidongdong (2014)	II-IV	10.1 ± 2.5	10.4 ± 2.7	20	20	12/8		12/8	60.9 ± 8.7	61.6 ± 7.6	PGE1 (2 mL/d) + SKI(60 mL/d)	PGE1 (2 mL/d)	14d	②③⑥
Yinlili (2014)	NA	7.1 ± 0.5		44	44	NA		NA	50.43 ± 6.90		PGE1 + SKI(100 mL/d)	PGE1	NA	①
Zhangbailing (2014)	IV	13.9 ± 4.3		35	35	38/32			54.5 ± 8.3		PGE1 (2 mL/d) + SKI(100 mL/d)	PGE1 (2 mL/d)	28d	②③④⑥
Zhangyunpin (2017)	NA	7.6 ± 2.3	7.5 ± 2.1	58	58	26/22		28/20	64.8 ± 8.3	64.7 ± 9.5	PGE1 (2 mL/d) + SKI(100 mL/d)	PGE1 (2 mL/d)	28d	①②③
Quanye (2018)	NA	8.6 ± 2.4	8.1 ± 2.3	30	30	18/12		17/13	64.8 ± 8.7	64.4 ± 8.5	PGE1 (2 mL/d) + SKI(60 mL/d)	PGE1 (2 mL/d)	14d	①②③⑥
Zhengwenwu (2018)	early stage	4.35 ± 1.26	4.61 ± 1.18	30	30	16/14		17/13	58.37 ± 4.49	58.19 ± 4.21	PGE1 (2 mL/d) + SKI(100 mL/d)	PGE1 (2 mL/d)	28d	②③
Wangxiaojun (2019)	NA	7.5 ± 1.2	7.7 ± 1.3	50	50	31/19		32/18	65.1 ± 3.1	65.3 ± 3.2	PGE1(2 mL/d) + SKI(100 mL/d)	PGE1 (2 mL/d)	30d	①
Zhangmailang (2019)	III-IV	NA	NA	48	48	25/23		26/22	54.26 ± 13.85	53.79 ± 13.42	PGE1 (2 mL/d) + SKI(60 mL/d)	PGE1 (2 mL/d)	30d	①②③⑥
Wenxusheng (2021)	IV	9.69 ± 2.26	10.23 ± 2.13	36	36	25/17		23/13	51.46 ± 3.37	52.37 ± 3.11	PGE1 (2 mL/d) + SKI(60 mL/d)	PGE1 (2 mL/d)	28d	①②④⑥⑦
Niexing (2022)	NA	NA	NA	50	50	29/21		27/23	63.12 ± 7.71	63.85 ± 7.62	PGE1 (2 mL/d) + SKI(60 mL/d)	PGE1 (2 mL/d)	28d	②③⑥
Rujianyong (2008)	III	12 ± 10	13 ± 9	64	64	34/30		35/29	58 ± 10	56 ± 9	PGE1 (2 mL/d) + SXTI(6 mL/d)	PGE1 (2 mL/d)	56d	②③④⑤
Gongyun (2009)	III	NA	NA	36	30	NA		NA	NA	NA	PGE1 (4 mL/d) + SXTI(6 mL/d)	PGE1 (4 mL/d)	15-20d	③④⑦
Xingyelan (2012)	III-IV	6.2 ± 2.6	5.7 ± 2.8	60	60	38/22		47/13	66.4 ± 9.7	65.2 ± 9.8	PGE1 (20 mL/d) + SXTI(6 mL/d)	PGE1 (20 mL/d)	28d	②③⑤⑦
Tiantian (2018)	III-IV	5.71 ± 0.72	5.68 ± 0.70	57	57	34/23		37/20	56.24 ± 6.12	55.78 ± 7.32	PGE1 (4 mL/d) + SXTI(2 mL/d)	PGE1 (4 mL/d)	14d	①②③⑤
Caoli (2016)	IV	8.3 ± 1.2	9.2 ± 1.3	33	31	18/15		17/14	54.2 ± 9.86	53.9 ± 12.5	PGE1 (2 mL/d) +XBJI (10 mL/d)	PGE1 (2 mL/d)	14d	②③④⑤⑥
Liqing (2016)	NA	5.9 ± 2.6	5.6 ± 2.7	30	30	17/13		13/17	65.6 ± 7.3	63.1 ± 9.5	PGE1 (2 mL/d) + XBJI (40 mL/d)	PGE1 (2 mL/d)	7d	②③⑥⑦
Jiangqiang (2017)	NA	6.02 ± 2.20	6.11 ± 2.44	78	78	48/30		44/34	59.54 ± 10.13	60.52 ± 10.34	PGE1 (4 mL/d) + XBJI (50 mL/d)	PGE1 (4 mL/d)	14d	②③④⑥

1 Total effective rate,② Serum Creatinine,③ Blood Urea Nitrogen, ④ Urinary Albumin excretion rates, ⑤ Urinary beta 2-microglobulin,⑥ 24h Urine Albumin, ⑦ Adverse reactions, SMI, Salvia miltiorrhiza injection; DCI, Danshen-Chuanxiongqin injection; DHI, Danhong injection; HQI, Huangqi injection; SKI, Shenkang injection; SXTI, Shuxuetong injection; XBJI, Xuebijing injection; PGE1, alprostadil injection.

### 3.2 Bias risk assessment and the grade of evidence

Among the 58 included studies, 15 studies described the methods used to generate the allocation sequence (Min et al., 2010; Zhou and Lai, 2012a; Pu et al., 2013; Yang et al., 2015; Cao et al., 2016; Liu et al., 2016; Zhang and Peng, 2016; Cui et al., 2017; Jiang and Qu, 2017; Liu, 2017; Mai et al., 2017; Zhong, 2017; Su, 2018; Tian et al., 2018; Wang, 2018), five studies were not random (Han and Zhang, 2013; Cai et al., 2015; Zhang, 2017a; Liang, 2018; Wen et al., 2021), the remaining studies did not explicitly address the random approach. None of the studies stated a pre-established research plan or analysis protocol. Overall, nine studies had a high risk (Han and Zhang, 2013; Lin, 2013; Yin, 2014; Cai et al., 2015; He, 2015; Zhang, 2017a; Zhang, 2017b; Liang, 2018; Wen et al., 2021), 49 studies had some concerns of bias(Xie and Zhang, 2002; Ru et al., 2008b; Gong and Xie, 2009; Wang et al., 2009; Min et al., 2010; Zhao and Dong, 2010; Pang et al., 2011; Wu, 2011; Zhou and Lai, 2012a; Xing, 2012; Liu, 2013; Pu et al., 2013; Zhang et al., 2013; Ding, 2014; Lan, 2014; Mei et al., 2014; Wang et al., 2014; Zhang, 2014; Zhou et al., 2014; Li and Li, 2015; Liu and Guo, 2015; Fang et al., 2016; Li et al., 2016; Liu et al., 2016; Liu and Sun, 2016; Zhang, 2016; Zhang and Peng, 2016; Cui et al., 2017; Jiang and Qu, 2017; Liu, 2017; Chen and Fu, 2018; Jia, 2018; Shen, 2018; Su, 2018; Tian et al., 2018; Xu and Liu, 2018; Ye, 2018; Zheng et al., 2018; Lin et al., 2019; Wang et al., 2019; Xiao, 2019; Zhang and Nan, 2019; Wang, 2021; Nie, 2022),. The results of the risk of bias assessment of the included studies are shown in Supplementary Figure S1 (page 3), Supplementary Table S1 (page 3–102). There are only indirect comparisons between TCMIs, which results in a very low-quality rating for pairwise comparisons, the details of evidence evaluation utilizing GRADE is available in the Supplementary Material (Supplementary Table S2, page 122–130).

### 3.3 Results of network meta-analysis

#### 3.3.1 Primary outcome measures

##### 3.3.1.1 Total effective rate

**FIGURE 3:**
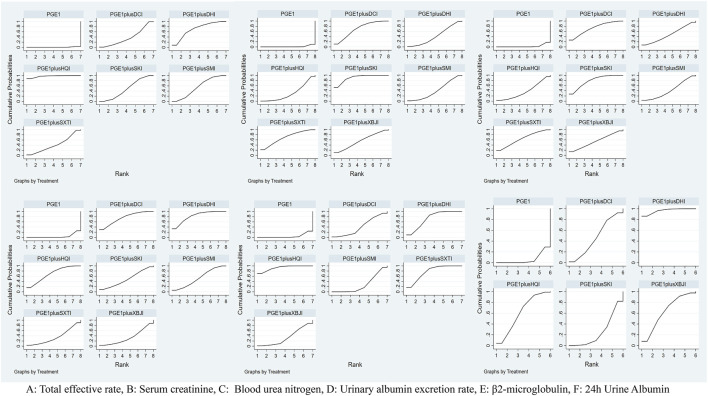
Plot of the surface under the cumulative ranking curves.

A total of 27 RCTs (Han and Zhang, 2013; Lin, 2013; Liu, 2013; Lan, 2014; Yin, 2014; Zhou et al., 2014; He, 2015; Li and Li, 2015; Liu and Guo, 2015; Fang et al., 2016; Liu and Sun, 2016; Zhang, 2016; Zhang and Peng, 2016; Zhang, 2017a; Zhang, 2017b; Liu, 2017; Zhong, 2017; Jia, 2018; Liang, 2018; Shen, 2018; Tian et al., 2018; Ye, 2018; Lin et al., 2019; Wang et al., 2019; Xiao, 2019; Zhang and Nan, 2019; Wen et al., 2021) reported the total effective rate, including six TCMIs and seven interventions. Five TCMIs combined with PGE1 were better than PGE1 alone, including PGE1+ DCI (RR: 1.17, CI: 1.02, 1.37), PGE1+DHI (RR: 1.28, CI: 1.13, 1.46), PGE1+HQI (RR: 1.43, CI: 1.26, 1.66), PGE1+SKI (RR: 1.2, CI: 1.12, 1.3), PGE1+SMI (RR: 1.24, CI: 1.15, 1.35), and PGE1+HQI were better than PGE1+DCI (RR: 1.23, CI: 1, 1.51), which suggested advantages in improving clinical symptoms (Table 3). According to the results of the SUCRA ranking (Table 4; Figure 3), PGE1+HQI (97.4%) was the best treatment, followed by PGE1+DHI (70.1%) and PGE1+SMI (54.9%).

**TABLE 3:** League table for all outcome measures.

Total effective rate	PGE1	PGE1plusDCI	PGE1plusDHI	PGE1plusHQI	PGE1plusSKI	PGE1plusSMI	PGE1plusSXTI	PGE1plusXBJI
PGE1	PGE1	**1.17 (1.02, 1.37)**	**1.28 (1.13, 1.46)**	**1.43 (1.26, 1.66)**	**1.2 (1.12, 1.3)**	**1.24 (1.15, 1.35)**	1.18 (0.99, 1.42)	—
PGE1plusDCI	—	PGE1plusDCI	1.09 (0.9, 1.34)	**1.23 (1, 1.51)**	1.03 (0.86, 1.21)	1.06 (0.89, 1.24)	1.01 (0.8, 1.28)	—
PGE1plusDHI	—	—	PGE1plusDHI	1.12 (0.93, 1.36)	0.94 (0.8, 1.08)	0.97 (0.83, 1.13)	0.92 (0.74, 1.15)	—
PGE1plusHQI	—	—	—	PGE1plusHQI	0.84 (0.71, 0.97)	0.87 (0.73, 1)	0.82 (0.65, 1.04)	—
PGE1plusSKI	—	—	—	—	PGE1plusSKI	1.03 (0.93, 1.15)	0.98 (0.82, 1.2)	—
PGE1plusSMI	—	—	—	—	—	PGE1plusSMI	0.95 (0.79, 1.16)	—
PGE1plusSXTI	—	—	—	—	—	—	PGE1plusSXTI	—
PGE1plusXBJI	—	—	—	—	—	—	—	PGE1plusXBJI
Serum Creatinine	PGE1	PGE1plusDCI	PGE1plusDHI	PGE1plusHQI	PGE1plusSKI	PGE1plusSMI	PGE1plusSXTI	PGE1plusXBJI
PGE1	PGE1	**−1.34 (-2.11, -0.56)**	**−0.87 (-1.7, -0.03)**	−0.65 (−1.42, 0.13)	**−1.78 (-2.39, -1.18)**	**−0.83 (-1.6, -0.06)**	−0.91 (−2.09, 0.28)	−1.1 (−2.27, 0.07)
PGE1plusDCI	—	PGE1plusDCI	0.48 (−0.67, 1.62)	0.69 (−0.41, 1.8)	−0.44 (−1.43, 0.54)	0.51 (−0.59, 1.6)	0.43 (−0.98, 1.86)	0.24 (−1.17, 1.65)
PGE1plusDHI	—	—	PGE1plusDHI	0.22 (−0.93, 1.36)	−0.91 (−1.95, 0.11)	0.03 (−1.1, 1.17)	−0.04 (−1.49, 1.42)	−0.24 (−1.68, 1.21)
PGE1plusHQI	—	—	—	PGE1plusHQI	**−1.13 (-2.12, -0.16)**	−0.19 (−1.28, 0.91)	−0.26 (−1.68, 1.16)	−0.46 (−1.86, 0.95)
PGE1plusSKI	—	—	—	—	PGE1plusSKI	0.94 (−0.03, 1.93)	0.87 (−0.45, 2.21)	0.67 (−0.64, 2)
PGE1plusSMI	—	—	—	—	—	PGE1plusSMI	−0.07 (−1.48, 1.34)	−0.27 (−1.67, 1.14)
PGE1plusSXTI	—	—	—	—	—	—	PGE1plusSXTI	−0.2 (−1.87, 1.47)
PGE1plusXBJI	—	—	—	—	—	—	—	PGE1plusXBJI
Blood Urea Nitrogen	PGE1	PGE1plusDCI	PGE1plusDHI	PGE1plusHQI	PGE1plusSKI	PGE1plusSMI	PGE1plusSXTI	PGE1plusXBJI
PGE1	PGE1	**−1.11 (-1.93, -0.29)**	−0.73 (−1.66, 0.19)	−0.59 (−1.34, 0.17)	**−1.16 (-1.73, -0.61)**	−0.66 (−1.35, 0.03)	**−1 (-1.91, -0.09)**	−0.83 (−1.89, 0.22)
PGE1plusDCI	—	PGE1plusDCI	0.38 (−0.86, 1.61)	0.52 (−0.59, 1.64)	−0.06 (−1.06, 0.93)	0.45 (−0.62, 1.53)	0.11 (−1.11, 1.34)	0.28 (−1.05, 1.61)
PGE1plusDHI	—	—	PGE1plusDHI	0.15 (−1.05, 1.34)	−0.43 (−1.52, 0.65)	0.08 (−1.08, 1.23)	−0.26 (−1.57, 1.04)	−0.1 (−1.5, 1.31)
PGE1plusHQI	—	—	—	PGE1plusHQI	−0.58 (−1.53, 0.36)	−0.07 (−1.09, 0.95)	−0.41 (−1.59, 0.77)	−0.25 (−1.54, 1.05)
PGE1plusSKI	—	—	—	—	PGE1plusSKI	0.51 (−0.38, 1.41)	0.17 (−0.9, 1.25)	0.33 (−0.86, 1.53)
PGE1plusSMI	—	—	—	—	—	PGE1plusSMI	−0.34 (−1.48, 0.8)	−0.17 (−1.44, 1.08)
PGE1plusSXTI	—	—	—	—	—	—	PGE1plusSXTI	0.16 (−1.23, 1.57)
PGE1plusXBJI	—	—	—	—	—	—	—	PGE1plusXBJI
Urinary Albumin excretion rates	PGE1	PGE1plusDCI	PGE1plusDHI	PGE1plusHQI	PGE1plusSKI	PGE1plusSMI	PGE1plusSXTI	PGE1plusXBJI
PGE1	PGE1	**−1.47 (-2.48, -0.47)**	**−1.57 (-2.25, -0.9)**	**−1.34 (-2.14, -0.56)**	−0.97 (−2.22, 0.26)	**−1.08 (-1.94, -0.22)**	−0.72 (−1.95, 0.5)	−0.65 (−1.86, 0.57)
PGE1plusDCI	—	PGE1plusDCI	−0.1 (−1.31, 1.1)	0.13 (−1.15, 1.4)	0.5 (−1.11, 2.09)	0.39 (−0.93, 1.7)	0.75 (−0.83, 2.34)	0.82 (−0.75, 2.4)
PGE1plusDHI	—	—	PGE1plusDHI	0.23 (−0.81, 1.26)	0.6 (−0.81, 2.01)	0.49 (−0.59, 1.59)	0.85 (−0.54, 2.26)	0.92 (−0.46, 2.32)
PGE1plusHQI	—	—	—	PGE1plusHQI	0.37 (−1.1, 1.84)	0.26 (−0.9, 1.44)	0.63 (−0.83, 2.09)	0.7 (−0.75, 2.16)
PGE1plusSKI	—	—	—	—	PGE1plusSKI	−0.11 (−1.61, 1.41)	0.26 (−1.48, 2.01)	0.33 (−1.41, 2.07)
PGE1plusSMI	—	—	—	—	—	PGE1plusSMI	0.37 (−1.14, 1.85)	0.43 (−1.06, 1.92)
PGE1plusSXTI	—	—	—	—	—	—	PGE1plusSXTI	0.07 (−1.65, 1.8)
PGE1plusXBJI	—	—	—	—	—	—	—	PGE1plusXBJI
Urinary beta 2-microglobulin	PGE1	PGE1plusDCI	PGE1plusDHI	PGE1plusHQI	PGE1plusSKI	PGE1plusSMI	PGE1plusSXTI	PGE1plusXBJI
PGE1	PGE1	−0.72 (−1.99, 0.53)	**−1.37 (-2, -0.81)**	**−1.79 (-2.69, -0.89)**	—	−0.44 (−1.16, 0.31)	**−1.43 (-2.16, -0.73)**	−0.57 (−1.84, 0.7)
PGE1plusDCI	—	PGE1plusDCI	−0.65 (−2.08, 0.71)	−1.07 (−2.62, 0.48)	—	0.28 (−1.16, 1.77)	−0.71 (−2.17, 0.74)	0.15 (−1.64, 1.95)
PGE1plusDHI	—	—	PGE1plusDHI	−0.42 (−1.46, 0.69)	—	**0.92 (0.04, 1.93)**	−0.07 (−0.96, 0.89)	0.8 (−0.56, 2.24)
PGE1plusHQI	—	—	—	PGE1plusHQI	—	**1.35 (0.21, 2.53)**	0.36 (−0.79, 1.5)	1.22 (−0.34, 2.79)
PGE1plusSKI	—	—	—	—	PGE1plusSKI	—	—	—
PGE1plusSMI	—	—	—	—	—	PGE1plusSMI	−0.99 (−2.05, 0.01)	−0.13 (−1.61, 1.32)
PGE1plusSXTI	—	—	—	—	—	—	PGE1plusSXTI	0.86 (−0.59, 2.33)
PGE1plusXBJI	—	—	—	—	—	—	—	PGE1plusXBJI
24 h Urine Albumin	PGE1	PGE1plusDCI	PGE1plusDHI	PGE1plusHQI	PGE1plusSKI	PGE1plusSMI	PGE1plusSXTI	PGE1plusXBJI
PGE1	PGE1	−0.83 (−2.08, 0.43)	**−2.49 (-3.96, -1.03)**	**−1.15 (-2.28, -0.03)**	−0.34 (−1.17, 0.49)	—	—	−1.26 (−2.7, 0.18)
PGE1plusDCI	—	PGE1plusDCI	−1.67 (−3.6, 0.25)	−0.32 (−2.03, 1.35)	0.48 (−1.02, 1.99)	—	—	−0.43 (−2.35, 1.47)
PGE1plusDHI	—	—	PGE1plusDHI	1.34 (−0.49, 3.19)	**2.15 (0.48, 3.84)**	—	—	1.23 (−0.81, 3.29)
PGE1plusHQI	—	—	—	PGE1plusHQI	0.81 (−0.57, 2.21)	—	—	−0.11 (−1.93, 1.72)
PGE1plusSKI	—	—	—	—	PGE1plusSKI	—	—	−0.92 (−2.58, 0.74)
PGE1plusSMI	—	—	—	—	—	PGE1plusSMI	—	—
PGE1plusSXTI	—	—	—	—	—	—	PGE1plusSXTI	—
PGE1plusXBJI	—	—	—	—	—	—	—	PGE1plusXBJI

Significant effects are printed in bold.

SMI, Salvia miltiorrhiza injection; DCI, Danshen-Chuanxiongqin injection; DHI, Danhong injection; HQI, Huangqi injection; SKI, Shenkang injection; SXTI, Shuxuetong injection; XBJI, Xuebijing injection; PGE1, alprostadil injection.

**TABLE 4:** Results of the surface under the cumulative ranking curve (SUCRA) (%).

	PGE1	PGE1plusDCI	PGE1plusDHI	PGE1plusHQI	PGE1plusSKI	PGE1plusSMI	PGE1plusSXTI	PGE1plusXBJI
总有效率	0.6	37.5	70.1	97.4	48.1	54.9	41.4	—
Scr	1.5	69	42.5	30.9	90.7	40.9	68.7	55.8
BUN	2.8	72.6	47.3	37.8	78.6	42.1	64.1	54.7
UAER	4	75	81.8	68.9	48.8	53	35.2	33.3
β2-MG	4.4	40.8	72.8	92.7	—	27.8	77.1	34.3
24h Alb	6.3	47.1	96.4	61	25.4	—	—	63.7

Red is the most likely to be the best intervention, yellow is second and green is third.

SMI, Salvia miltiorrhiza injection; DCI, Danshen-Chuanxiongqin injection; DHI, Danhong injection; HQI, Huangqi injection; SKI, Shenkang injection; SXTI, Shuxuetong injection; XBJI, Xuebijing injection; PGE1, alprostadil injection.

##### 3.3.1.2 Scr

A total of 45 RCTs (Xie and Zhang, 2002; Ru et al., 2008b; Wang et al., 2009; Min et al., 2010; Wu, 2011; Zhou and Lai, 2012a; Xing, 2012; Lin, 2013; Liu, 2013; Pu et al., 2013; Zhang et al., 2013; Ding, 2014; Lan, 2014; Mei et al., 2014; Wang et al., 2014; Zhang, 2014; Zhou et al., 2014; Cai et al., 2015; Liu and Guo, 2015; Yang et al., 2015; Cao et al., 2016; Li et al., 2016; Liu et al., 2016; Liu and Sun, 2016; Zhang, 2016; Zhang and Peng, 2016; Zhang, 2017a; Zhang, 2017b; Cui et al., 2017; Jiang and Qu, 2017; Mai et al., 2017; Chen and Fu, 2018; Jia, 2018; Shen, 2018; Su, 2018; Tian et al., 2018; Wang, 2018; Xu and Liu, 2018; Ye, 2018; Zheng et al., 2018; Xiao, 2019; Zhang and Nan, 2019; Wang, 2021; Wen et al., 2021; Nie, 2022) reported the Scr, including seven TCMIs and 8 interventions. 4 TCMIs combined with PGE1 were better than single used PGE1, including PGE1+DCI (RR: −1.34, CI: −2.11, −0.56), PGE1+DHI (SMD: −0.87, CI: −1.7, −0.03), PGE1+SKI (SMD: −1.78, CI: −2.39, −1.18), and PGE1+SMI (SMD: −0.83, CI: −1.6, −0.06). PGE1+SKI was superior to PGE1+HQI (SMD: −1.13, CI: −2.12, −0.16), indicating its advantages in improving Scr (Table 3). According to the results of the SUCRA ranking (Table 4; Figure 3), PGE1+SKI (90.7%) was the best treatment, followed by PGE1+DCI (69%), and PGE1+ SXTI (68.7%).

#### 3.3.2 Secondary outcome measures

##### 3.3.2.1 BUN

A total of 40 RCTs (Xie and Zhang, 2002; Ru et al., 2008b; Gong and Xie, 2009; Wang et al., 2009; Min et al., 2010; Wu, 2011; Xing, 2012; Zhou and Lai, 2012a; Liu, 2013; Zhang et al., 2013; Ding, 2014; Lan, 2014; Mei et al., 2014; Wang et al., 2014; Zhang, 2014; Zhou et al., 2014; Cai et al., 2015; Liu and Guo, 2015; Cao et al., 2016; Li et al., 2016; Liu et al., 2016; Liu and Sun, 2016; Zhang, 2016; Zhang and Peng, 2016; Cui et al., 2017; Jiang and Qu, 2017; Mai et al., 2017; Zhang, 2017a; Zhang, 2017b; Chen and Fu, 2018; Su, 2018; Wang, 2018; Xu and Liu, 2018; Ye, 2018; Zheng et al., 2018; Xiao, 2019; Zhang and Nan, 2019; Wang, 2021; Nie, 2022) reported the BUN, including 7 TCMIs and 8 interventions. The results showed that PGE1+DCI (SMD: −1.11, CI: −1.93, −0.29), PGE1+SKI (SMD: −1.16, CI: −1.73, −0.61), and PGE1+SXTI (SMD: −1, CI: −1.91, −0.09) were better than single used PGE1 (Table 3). According to the results of the SUCRA ranking (Table 4; Figure 3), PGE1+SKI (78.6%) was the best treatment, followed by PGE1+DCI (72.6%) and PGE1+SXTI (64.1%).

##### 3.3.2.2 UAER

A total of 25 RCTs (Ru et al., 2008b; Gong and Xie, 2009; Min et al., 2010; Wu, 2011; Zhou and Lai, 2012a; Liu, 2013; Pu et al., 2013; Zhang, 2014; Cai et al., 2015; Li and Li, 2015; Liu and Guo, 2015; Cao et al., 2016; Fang et al., 2016; Liu et al., 2016; Liu and Sun, 2016; Zhang, 2016; Jiang and Qu, 2017; Mai et al., 2017; Chen and Fu, 2018; Su, 2018; Xu and Liu, 2018; Lin et al., 2019; Xiao, 2019; Wang, 2021; Wen et al., 2021) reported UAER, including 7 TCMIs and 8 interventions. The results showed that using PGE1+DCI (SMD: −1.47, CI: −2.48, −0.47), PGE1+DHI (SMD: −1.57, CI: −2.25, −0.9), PGE1+HQI (SMD: −1.34, CI: −2.14, −0.56), and PGE1+SMI (SMD: −1.08, CI: −1.94, −0.22) were better than single used PGE1(Table 3). According to the results of the SUCRA ranking (Table 4; Figure 3), PGE1+DHI (81.8%) was the best treatment, followed by PGE1+DCI (75%) and PGE1+HQI (68.9%).

##### 3.3.2.3 *β*2-MG

A total of 15 RCTs (Ru et al., 2008b; Min et al., 2010; Zhou and Lai, 2012a; Xing, 2012; Pu et al., 2013; Wang et al., 2014; Cai et al., 2015; Cao et al., 2016; Liu et al., 2016; Liu and Sun, 2016; Zhang, 2016; Mai et al., 2017; Tian et al., 2018; Xu and Liu, 2018; Lin et al., 2019) reported β2-MG, including 6 TCMIs and 7 interventions. On the one hand, using PGE1+DHI (SMD: −1.37, CI: −2, −0.81), PGE1+HQI (SMD: −1.79, CI: −2.69, −0.89), and PGE1+SXTI (SMD: −1.43, CI: −2.16, −0.73) was better than single used PGE1. On the other hand, PGE1+HQI (SMD:1.35, CI:0.21, 2.53) and PGE1+DHI (SMD:0.92, CI:0.04, 1.93) were better than PGE1+SMI, indicating excellent performance in improving β2-MG (Table 3). According to the results of the SUCRA ranking (Table 4; Figure 3), PGE1+HQI (92.7%) was the best treatment, followed by PGE1+SXTI (77.1%) and PGE1+DHI (72.8%).

##### 3.3.2.4 24 h Alb

A total of 24 RCTs (Xie and Zhang, 2002; Wang et al., 2009; Zhao and Dong, 2010; Lin, 2013; Zhang et al., 2013; Lan, 2014; Mei et al., 2014; Zhang, 2014; Li and Li, 2015; Yang et al., 2015; Cao et al., 2016; Fang et al., 2016; Li et al., 2016; Zhang and Peng, 2016; Cui et al., 2017; Jiang and Qu, 2017; Jia, 2018; Shen, 2018; Ye, 2018; Lin et al., 2019; Zhang and Nan, 2019; Wen et al., 2021; Nie, 2022) reported 24 h Alb, including five TCMIs and six interventions. Using PGE1+DHI (SMD: −2.49, CI: −3.96, −1.03), PGE1+HQI (SMD: −1.15, CI: −2.28, −0.03) was better than using PGE1 alone. PGE1+ DHI (SMD: 2.15, CI: 0.48, 3.84) was better than PGE1+SKI, indicating that it may be better to improve 24 h Alb (Table 3). According to the results of the SUCRA ranking (Table 4; Figure 3), PGE1+DHI (96.4%) was the best treatment, followed by PGE1+XBJI (63.7%) and PGE1+HQI (61%).

### 3.4 Cluster analysis

**FIGURE 4:**
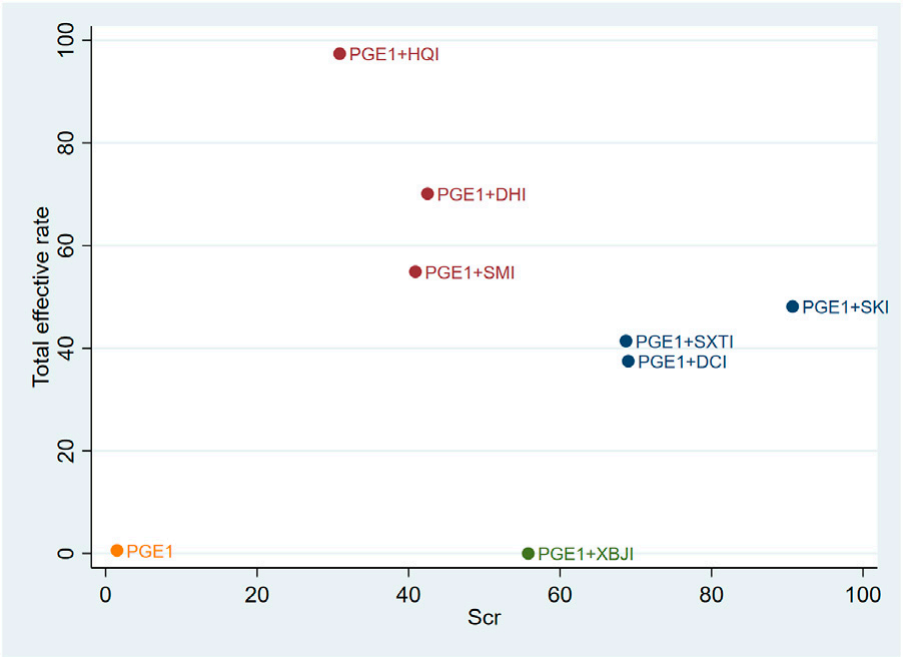
Cluster diagram of primary outcome indicators.

**FIGURE 5:**
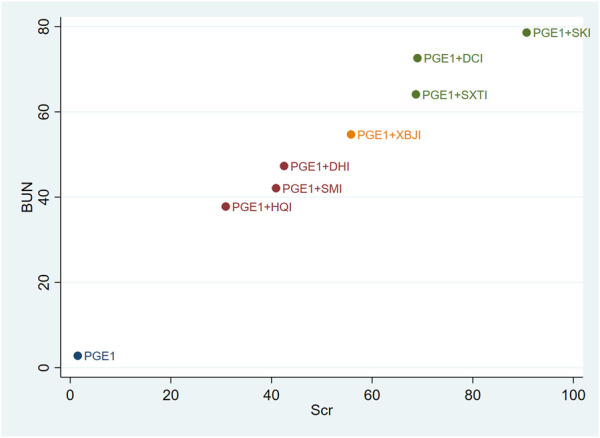
Clustering diagram of Scr and BUN.

**FIGURE 6:**
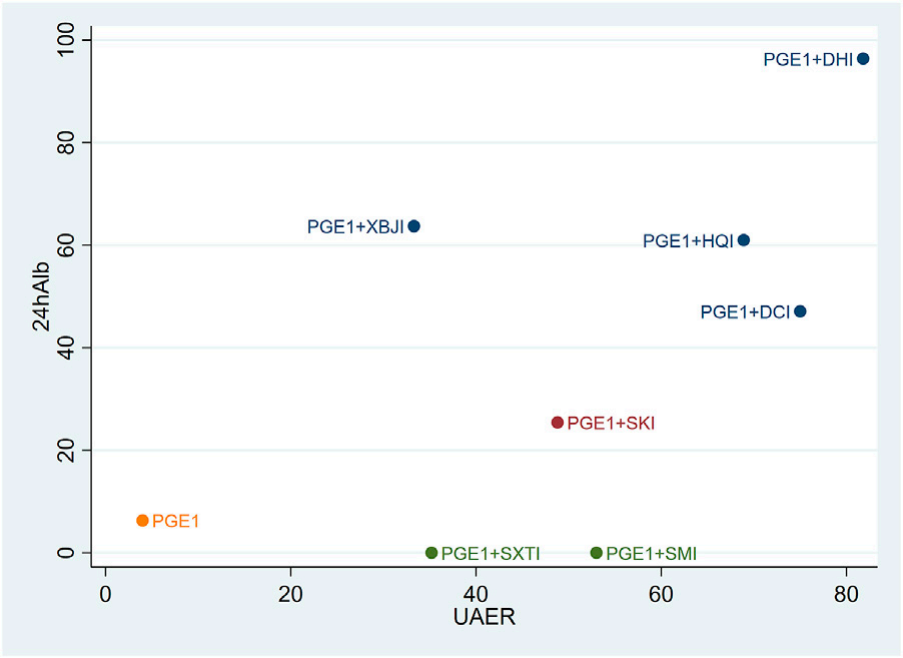
Clustering diagram of 24h Alb and UAER.

Cluster analysis was used to analyse the interventions for multi-dimensional outcomes and identify the best intervention measures under clustering of primary outcome indicators, glomerular filtration function, and urinary protein-related indicators. In terms of the primary outcome measure (total effective rate and Scr), PGE1+HQI and PGE1+SKI may be the best treatments (Figure 4). In terms of glomerular filtration function (Scr and BUN), PGE1+SKI was the best treatment (Figure 5). In terms of urinary protein-related indicators (24 h Alb & UAER), PGE1+DHI was the best treatment (Figure 6).

### 3.5 Minimally contextualized framework

PGE1 was selected as the reference group. Based on the comparison of whether the 95% confidence interval of the effect size of the reference group intersected the decision threshold for each intervention, the intervention measures were divided into “Category 0,” which had no difference compared with the intervention group, and “Category 1,” which was better than the intervention group. Then, a secondary classification was conducted based on the differences between the interventions. The intervention with the smallest effect size in Category 1 was taken as the reference, and the intervention with better effect was classified into Category 2. Interventions were then classified into high and low reliability categories based on GRADE classification, and the consistency of classification was checked by ranking results. In this study, those interventions with the highest ranking were ensured to be among the most effective (see Table 5).

**TABLE 5:** Final classification of 7 interventions for Diabetic kidney disease.

	Certainty of the evidence, and classification of intervention		Intervention	Intervention vs. PGE1 SMD (95% CI)	SUCRA
	High certainty (moderate to high certainty evidence)				
	Category 2: among the most effective		PGE1plusHQI(M)	1.43 (1.26, 1.66)	97.4
	Category 1: inferior to the most effective, or superior to the least effective		PGE1plusDHI(M)	1.28 (1.13, 1.46)	70.1
			PGE1plusSKI(M)	1.2 (1.12, 1.3)	48.1
Total effective rate	Low certainty (low to very low certainty evidence)				
	Category 1: inferior to the most effective, or superior to the least effective		PGE1plusSMI(L)	1.24 (1.15, 1.35)	54.9
			PGE1plusDCI(L)	1.17 (1.02, 1.37)	37.5
	Category 0: among the least effective		PGE1plusSXTI(VL)	1.18 (0.99, 1.42)	41.4
					
	High certainty (moderate to high certainty evidence)				
	Category 1: inferior to the most effective, or superior to the least effective		PGE1plusSMI(M)	−0.83 (−1.6, −0.06)	40.9
	Low certainty (low to very low certainty evidence)				
	Category 2: among the most effective		PGE1plusSKI(L)	−1.78 (−2.39, −1.18)	90.7
Serum Creatinine	Category 1: inferior to the most effective, or superior to the least effective		PGE1plusDCI(VL)	−1.34 (−2.11, −0.56)	69
			PGE1plusDHI(L)	−0.87 (−1.7, −0.03)	42.5
	Category 0: among the least effective		PGE1plusHQI(L)	−0.65 (−1.42, 0.13)	30.9
			PGE1plusSXTI(VL)	−0.91 (−2.09, 0.28)	68.7
			PGE1plusXBJI(L)	−1.1 (−2.27, 0.07)	55.8
	High certainty (moderate to high certainty evidence)				
Blood Urea Nitrogen	Category 1: inferior to the most effective, or superior to the least effective		PGE1plusSKI(M)	−1.16 (−1.73, −0.61)	78.6
	Category 0: among the least effective		PGE1plusDHI(M)	−0.73 (−1.66, 0.19)	47.3
			PGE1plusSMI(M)	−0.66 (−1.35, 0.03)	42.1
	Low certainty (low to very low certainty evidence)				
	Category 1: inferior to the most effective, or superior to the least effective		PGE1plusDCI(L)	−1.11 (−1.93, −0.29)	72.6
			PGE1plusSXTI(L)	−1 (−1.91, −0.09)	64.1
	Category 0: among the least effective		PGE1plusXBJI(L)	−0.83 (−1.89, 0.22)	54.7
			PGE1plusHQI(L)	−0.59 (−1.34, 0.17)	37.8
	Low certainty (low to very low certainty evidence)				
	Category 1: inferior to the most effective, or superior to the least effective		PGE1plusDHI(L)	−2.49 (−3.96, −1.03)	96.4
			PGE1plusHQI(L)	−1.15 (−2.28, −0.03)	61
24h Urine Albumin	Category 0: among the least effective		PGE1plusXBJI(VL)	−1.26 (−2.7, 0.18)	63.7
			PGE1plusDCI(L)	−0.83 (−2.08, 0.43)	47.1
			PGE1plusSKI(L)	−0.34 (−1.17, 0.49)	25.4
	High certainty (moderate to high certainty evidence)				
	Category 1: inferior to the most effective, or superior to the least effective		PGE1plusDHI(M)	−1.57 (−2.25, −0.9)	81.8
			PGE1plusDCI(M)	−1.47 (−2.48, −0.47)	75
Urinary Albumin excretion rates	Category 0: among the least effective		PGE1plusXBJI(M)	−0.65 (−1.86, 0.57)	33.3
	Low certainty (low to very low certainty evidence)				
	Category 1: inferior to the most effective, or superior to the least effective		PGE1plusHQI(L)	−1.34 (−2.14, −0.56)	68.9
	Category 0: among the least effective		PGE1plusSKI(L)	−0.97 (−2.22, 0.26)	48.8
			PGE1plusSXTI(L)	−0.72 (−1.95, 0.5)	35.2
	High certainty (moderate to high certainty evidence)				
Urinary beta 2-microglobulin	Category 1: inferior to the most effective, or superior to the least effective		PGE1plusHQI(M)	−1.79 (−2.69, −0.89)	92.7
	Category 0: among the least effective		PGE1plusSMI(M)	−0.44 (−1.16, 0.31)	27.8
	Low certainty (low to very low certainty evidence)				
	Category 1: inferior to the most effective, or superior to the least effective		PGE1plusSXTI(L)	−1.43 (−2.16, −0.73)	77.1
			PGE1plusDHI(L)	−1.37 (−2, −0.81)	72.8
	Category 0: among the least effective		PGE1plusDCI(VL)	−0.72 (−1.99, 0.53)	40.8
			PGE1plusXBJI(L)	−0.57 (−1.84, 0.7)	34.3

H, high certainty evidence; M, moderate; L, low; VL, very low.

### 3.6 Heterogeneity and consistency tests

This study found that most of the heterogeneity in the heterogeneity assessment was mild to moderate, in which the total effective rate was not substantially heterogeneous, and consistent models fit similarly or better than inconsistent models. See details in Supplementary Figures S2–S7 (page 103–108) and Supplementary Table S13 (page 103–108).

### 3.7 Safety

Seventeen RCTs (Xie and Zhang, 2002; Gong and Xie, 2009; Wang et al., 2009; Zhao and Dong, 2010; Pang et al., 2011; Xing, 2012; Liu, 2013; Pu et al., 2013; Ding, 2014; Li et al., 2016; Liu et al., 2016; Zhang, 2016; Xu and Liu, 2018; Lin et al., 2019; Xiao, 2019; Wang, 2021; Wen et al., 2021) reported the specific adverse reactions and safety of TCMIs, including pain, redness, and swelling at the injection site; dizziness; headache; vomiting; and diarrhoea. Only a descriptive analysis was performed because the description criteria of the various studies were not uniform. The specific information is given in Table 6.

**TABLE 6:** Summary of adverse drug events.

Type of interventions	Number of RCTs	Groups	Total sample size	Incidence	Detailed ADR events (number of cases)
PGE1+SMI vs. PGE1	0	PGE1+SMI	0	—	—
		PGE1	0	—	—
PGE1+DCI vs. PGE1	1	PGE1+DCI	65	3.08%	Anorexia (1), constipation(1)
		PGE1	65	4.62%	Diarrhea (1), Dizziness(1), Fever(1)
PGE1+DHI vs. PGE1	4	PGE1+DHI	147	0.00%	None
		PGE1	144	0.00%	None
PGE1+HQI vs. PGE1	5	PGE1+HQI	433	16.86%	Dizziness (19), headache (17), pain at the injection site (36), allergies (1)
		PGE1	415	14.70%	Dizziness (24), pain at the injection site (27), redness at the injection site (4), vomiting (5), diarrhea (1),
PGE1+SKI vs. PGE1	4	PGE1+SKI	136	0.74%	Pain and flushing at the injection site (1)
		PGE1	116	0.00%	None
PGE1+SXTI vs. PGE1	2	PGE1+SXTI	96	0.00%	None
		PGE1	90	0.00%	None
PGE1+XBJI vs. PGE1	1	PGE1+XBJI	30	0.00%	None
		PGE1	30	0.00%	None

SMI, Salvia miltiorrhiza injection; DCI, Danshen-Chuanxiongqin injection; DHI, Danhong injection; HQI, Huangqi injection; SKI, Shenkang injection; SXTI, Shuxuetong injection; XBJI, Xuebijing injection; PGE1, alprostadil injection.

### 3.8 Sensitivity analysis

Four studies had treatment durations that were not in the 14 days–30 days range (Ru et al., 2008b; Pu et al., 2013; Yin, 2014; Li et al., 2016). A study was included in the primary outcome, the total effective rate (Yin, 2014). The findings indicated that excluding this study did not significantly change the overall analysis. 3 studies were included in the primary outcome measure Scr (Ru et al., 2008b; Pu et al., 2013; Li et al., 2016). And the outcomes demonstrated that removing these studies did not significantly change the overall analysis.

Nine studies were identified as having a high risk of bias (Han and Zhang, 2013; Lin, 2013; Yin, 2014; Cai et al., 2015; He, 2015; Zhang, 2017a; Zhang, 2017b; Liang, 2018; Wen et al., 2021), 7 studies were included in the primary outcome measure total response rate (Han and Zhang, 2013; Lin, 2013; Yin, 2014; He, 2015; Zhang, 2017a; Zhang, 2017b; Wen et al., 2021), and 4 studies were included in the primary outcome measure Scr (Lin, 2013; Cai et al., 2015; Zhang, 2017b; Wen et al., 2021). The results revealed that removing these studies separately had no discernible impact on the overall analysis.

### 3.9 Publication bias

**FIGURE 7:**
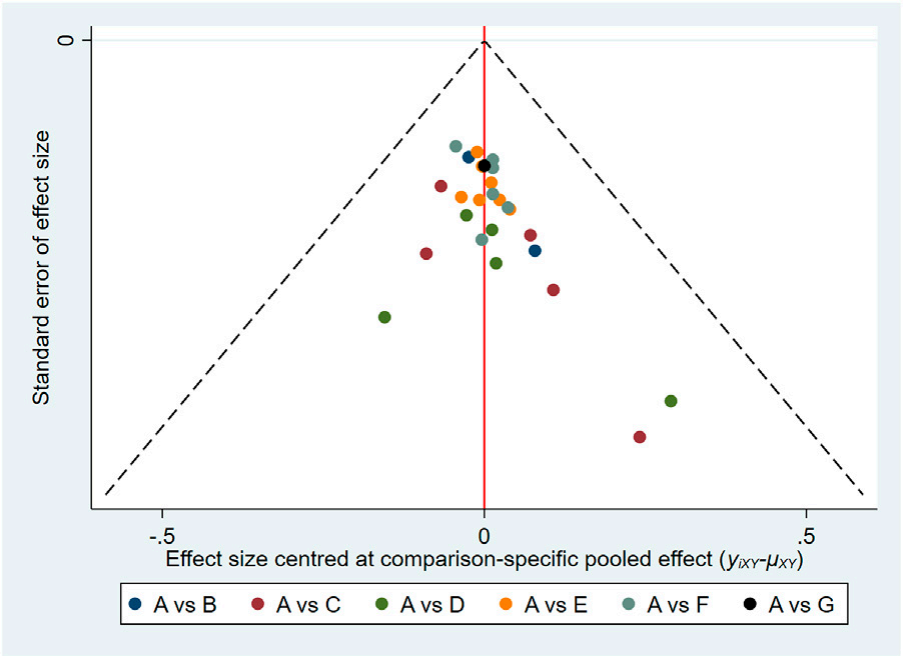
Funnel plots for total effective rate.

**FIGURE 8:**
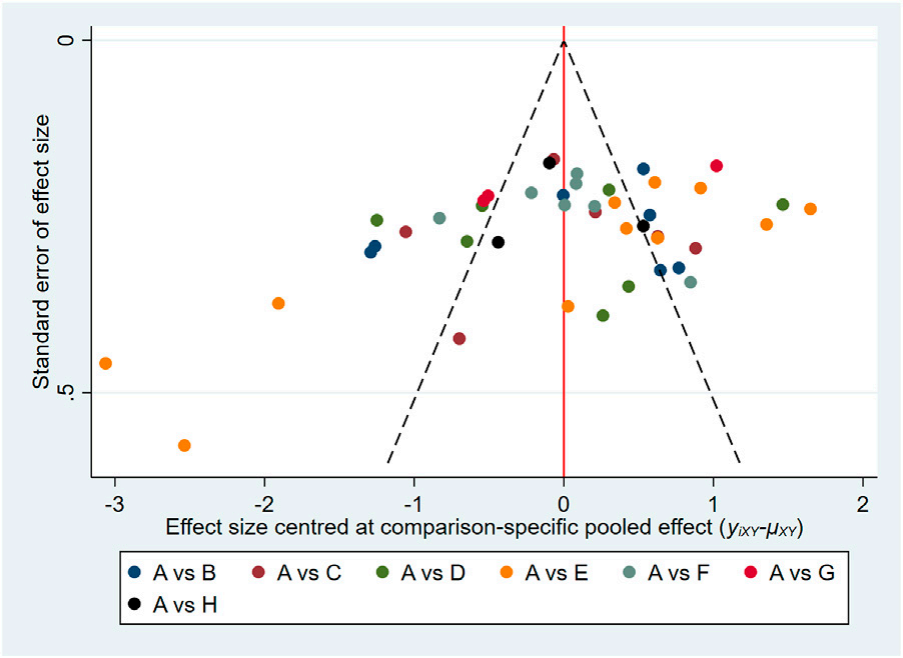
Funnel plots for Scr.

In this study, the funnel plots of the total effective rate (Figure 7) and Scr (Figure 8) were plotted. The results showed that the distribution of the total effective rate and Scr funnel plots were roughly symmetric, without obvious small-sample effect and publication bias.

### 3.10 Trial sequential analysis

For each of the groups of PGE1+SMI vs. PGE1, PGE1+DHI vs. PGE1, PGE1+SKI vs. PGE1, and PGE1+XBJI vs. PGE1, the cumulative Z-curve of Scr crosses the trial sequential monitoring and the required information size, indicating that SMI, DHI, SKI, and XBJI are effective for reducing Scr. Furthermore, the evidence of DCI, HQI, and SXTI was not sufficient. In terms of the total effective rate, the cumulative Z-curve crossed the trial sequential monitoring and the required information size in the comparisons of PGE1+HQI vs. PGE1 and PGE1+SKI vs. PGE1, which proved to be beneficial to the total effective rate, while the evidence of other injections was insufficient (Supplementary Figures S8–S20, page 109–121).

## 4 Discussion

### 4.1 Discussion of survey results

Our study found that SKI had obvious overall advantages in improving Scr and BUN levels. SKI is composed of *Salvia miltiorrhiza Bunge, Astragalus mongholicus Bunge, Carthamus tinctorius L., Rheum palmatum L*, and was approved to use on chronic kidney disease by China’s State Food and Drug Administration in 1999 (Licence No. YBZ08522004). The main components of SKI can reduce albuminuria, inhibit fibrosis, improve microcirculation, and regulate renal haemodynamic, and showed effects on glomerular and tubular lesions (Huang et al., 2012; Wang et al., 2012; Sun et al., 2016; Xu et al., 2020). A study showed that SKI can prevent renal tubular cell senescence under hyperglycaemia situation by reducing the expression of ageing markers P16INK4, cyclin D1, DcR2, and SA-β-Gal activity (Fu et al., 2019). It also can inhibit renal fibrosis and oxidative stress by downregulating the TGF-β/Smad3 signalling pathway. It brought significantly effects on improving Scr and BUN and alleviating renal injury (Wu et al., 2015; Wang et al., 2021). It also reduces glomerular hyperfiltration, hypertension, and hyperfusion situation (Zou et al., 2020).

DHI most effective on improving urinary protein levels. The main components in DHI are *Salvia miltiorrhiza Bunge* and *Carthamus tinctorius L*. Studies showed they can improve energy metabolism, oxidative stress, and autophagy and restore mitochondrial energy (Guo et al., 2021; Zeng et al., 2021). Study showed that DHI can inhibit glomerular hypertrophy, and markedly reduce urinary protein excretion in db/db mice (Liu et al., 2015). It also can delay the progression of renal injury by upregulating microRNA-30D-5P and targeting JAK1 (Deng et al., 2022).

HQI was the most effective for the total effective rate and β2-MG. The main component in HQI is *Astragalus mongholicus Bunge.* Main compounds of *Astragalus mongholicus Bunge* are polysaccharides, astragalus saponins, and flavonoids (Li et al., 2014), which inhibit oxidative stress (Ma et al., 2013), immune adjustment (Cho and Leung, 2007), anti-inflammatory (Zhang et al., 2003), and protect vascular endothelial cells (Wang et al., 2013; Zhu et al., 2013).

Studies have found that DKD occurs earlier than glomerular disease (Magri and Fava, 2009; Hasegawa et al., 2013). Therefore, the proximal renal tubules may be a new therapeutic target for the treatment of DKD. *Astragalus mongholicus Bunge* can ameliorate renal tubular injury and reduce the area, lumen, and wall to nearly normal (Sun et al., 2016), which is consistent with the results of this study.

### 4.2 Relationships and comparisons with other studies

To the best of our knowledge, this study is the first to compare the differences in the efficacy of TCMIs in the treatment of DKD through a network meta-analysis. Most of the previous studies (Zhang et al., 2022; Zhao et al., 2022), only conducted systematic reviews and network meta-analyses on TCM decoction. Those studies could not have stable quality control due to the diversity of ingredients and dose variability. The composition of TCMIs is more stable than TCM decoctions, which has quantitative significance. We comprehensively studied the RCTs of TCMIs combined with PGE1 in the treatment of DKD and ranked the advantage of each outcome index of different TCMIs to guide the clinical use.

### 4.3 Implications for clinical practice

This study found that SKI + PGE1 most effective on glomerular filtration function, DHI + PGE1 most effective on urinary protein, and HQI + PGE1 most effective on total effective rate and reduce clinical symptoms. TCMIs can effectively solve different problems of DKD. Non-study showed the effects of combination of multiple TCMIs in the treatment of DKD. This may be related to the complexity of the components, interactions, and other factors, which need to be further explored in subsequent studies.

Xie et al. found that the UAER of the 3 weeks treatment group decreased the fastest (Xie et al., 2021). A meta-analysis of the treatment of DKD with HQI found that the efficacy of a long course (>4 weeks) was better than that of a short course (<4 weeks) (Zhang and Kong, 2018). In this review, the duration of treatment in the 54 included studies focused on 1 month, 2 studies had longer treatment periods (Ru et al., 2008b; Pu et al., 2013), 1 study was in 7 days (Li et al., 2016) and 1 study was not mentioned (Yin, 2014).

Allergic reactions are the most common adverse events of using TCMIs (Wen et al., 2020). According to the studies included in this review, the adverse reactions of TCMIs are mild and can be relieved or eliminated by reducing the dosage, stopping medication, or symptomatic treatment (Xie and Zhang, 2002; Zhao and Dong, 2010; Pang et al., 2011; Zhang, 2016; Xu and Liu, 2018). The safety of TCMIs greatly improved by standardized the use in clinical application (Li et al., 2022). Li et al. improved the quality standard of solvent-enhancing polysorbate 80 in TCMI to reduce anaphylactic reactions (Li, 2018). However, the adverse reactions of patients still need to be concerned to avoid medical accidents.

TCMIs were widely used and effective in clinical practice. However, it was found that the specific extract components, complex pharmacological mechanisms and methodological descriptions of the botanical drugs were not clear in this included studies. In the future, relevant studies should follow the suggestions of consensus (Heinrich et al., 2020) and conduct more critical pharmacological studies on TCMIs.

### 4.4 Limitations

This study had the following limitations: (a) less reports on adverse reactions, and most of the studies had no clear safety assessment; (b) most of the literatures were “some concerns” in the risk assessment of bias and the quality of literatures was not high; (c) Have clinical heterogeneity due to the differences in botanical drug doses and treatment courses; (d) all included literatures were in China.

## 5 Conclusion

This study suggests that the combination of TCMIs and PGE1 provide additional benefits to patients with DKD. In terms of different outcome indicators, SKI had more effective on improving glomerular filtration function, DHI more effective on reducing urinary protein, and HQI more effective on improving renal tubular function. Despite the low incidence of adverse events, only a few studies have evaluated the safety of TCMIs. Further research on TCMIs treatment is needed for better understanding about TCMIs and guide the clinical application.

## Data Availability

The original contributions presented in the study are included in the article/Supplementary Materials, further inquiries can be directed to the corresponding author.

## Abbreviations

24 h Alb, 24 h urinary albumin; 95% CI, 95% confidence intervals; β2-MG, urinary β2-microglobulin; BUN, blood urea nitrogen; CNKI, China National Knowledge Infrastructure; DCI, Danshen-Chuanxiongqin injection; DHI, Danhong injection; DKD, Diabetic kidney disease; HQI, Huangqi injection; MD, mean differences; NMA, network meta-analysis; PGE1, Prostaglandin E1; PROSPERO, International Prospective Register of Systematic Reviews; RAAS, reninangiotensin-aldosterone system; RCT, randomised controlled trial; RR, risk ratio; Scr, serum creatinine; SGLT-2, sodium-dependent glucose transporter 2; SKI, Shenkang injection; SMD, standardised mean differences; SMI, Salvia miltiorrhiza injection; SUCRA, Surface under the cumulative ranking curve; SXTI, Shuxuetong injection; TCM, Traditional Chinese medicine; TCMI, Traditional Chinese medicine injection; TSA, Trial Sequential Analyses; UAER, urinary albumin excretion rate; VIP, Chinese scientific journal database; XBJI, Xuebijing injection.
