# Supplementary material for: Efficacy of traditional Chinese medicine injection for diabetic kidney disease: A network meta analysis and systematic review
Source: Front Pharmacol. 2023 Feb 17;14:1028257. doi: 10.3389/fphar.2023.1028257 (PMC9981802; doi:10.3389/fphar.2023.1028257)
Supplement: Supplementary file 1 [file DataSheet1.PDF]

***SUPPLEMENTS***

# Supplement the contents of diagrams, tables, PRISMA checklists


*Supplementary figure S1 Risk of Bias of Included Studies* ........ 3
*Supplementary table S1 More details about risk of bias* ........ 4
*Supplementary figure S2-S7: Heterogeneity of outcome measures* ........ 103
Figure S2: Total effective rate ........ 103
Figure S3: Serum Creatinine ........ 104
Figure S4: Blood Urea Nitrogen ........ 105
Figure S5: Urinary Albumin excretion rates ........ 106
Figure S6: 24h Urine Albumin ........ 107
Figure S7: Urinary beta 2-microglobulin ........ 108
*Supplementary figure S8-S20: The Trial Sequential Analysis of the primary outcomes* ........ 109
Figure S8: Trial Sequential Analysis of total effective rate on PGE1+SMI ........ 109
Figure S9: Trial Sequential Analysis of total effective rate on PGE1+DCI ........ 110
Figure S10: Trial Sequential Analysis of total effective rate on PGE1+DHI ........ 111
Figure S11: Trial Sequential Analysis of total effective rate on PGE1+HQI ........ 112
Figure S12: Trial Sequential Analysis of total effective rate on PGE1+SKI ........ 113
Figure S13: Trial Sequential Analysis of total effective rate on PGE1+SXTI ........ 114
Figure S14: Trial Sequential Analysis of serum creatinine on PGE1+SMI ........ 115
Figure S15: Trial Sequential Analysis of serum creatinine on PGE1+DCI ........ 116
Figure S16: Trial Sequential Analysis of serum creatinine on PGE1+DHI ........ 117
Figure S17: Trial Sequential Analysis of serum creatinine on PGE1+HQI ........ 118
Figure S18: Trial Sequential Analysis of serum creatinine on PGE1+SKI ........ 119
Figure S19: Trial Sequential Analysis of serum creatinine on PGE1+SXTI ........ 120

Figure S20: Trial Sequential Analysis of serum creatinine on PGE1+XBJ ........ 121
*Supplementary Table S2: GRADE assessment* ........ 122
*Supplementary Table S3-S10: Search Strategy* ........ 131
Table S3. Search Strategy: PubMed ........ 131
Table S4. Search Strategy: Embase ........ 132
Table S5. Search Strategy: Cochrane Library ........ 134
Table S6. Search Strategy: Web of Science ........ 135
Table S7. Search Strategy: the Chinese Scientific Journal database (VIP) ........ 136
Table S8. Search Strategy: Wanfang database ........ 136
Table S9. Search Strategy: China National Knowledge Infrastructure (CNKI) ........ 136
Table S10. Search Strategy: SinoMed ........ 136
*Supplementary table S11: Summary of All Included Studies* ........ 137
*Supplementary table S12: Extract and extraction process description of TCMIs* ........ 142
*Supplementary table S13: More details about the product information of 7 TCMI* ........ 144
*Supplementary table S14: Specific intervention methods of the study* ........ 148
*Supplementary table S15: Comparisons of the fit of consistency and inconsistency models using deviance information criteria (DIC).* ........ 153
*Supplementary table S16: League table for treatment comparisons in sensitivity analyses by excluding studies that were not within 14 to 30 days of treatment* ........ 154
*Supplementary table S17: League table for treatment comparisons in sensitivity analyses by excluding studies that were high risk bias* ........ 155
*Supplementary table S18: The PRISMA checklist of this meta-analysis* ........ 156

*Supplementary figure S1 Risk of Bias of Included Studies*

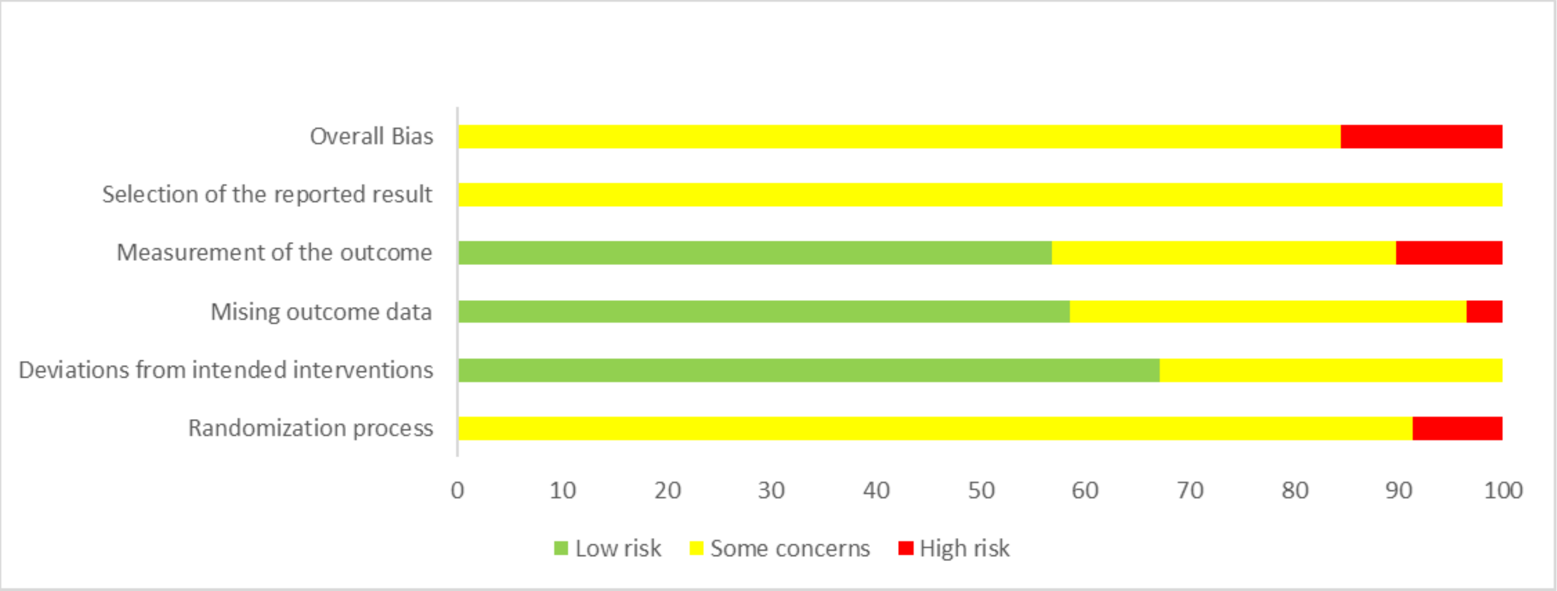

*Supplementary table S1 More details about risk of bias*

| PGE1+SMI | | | | |
|---|---|---|---|---|
| Assessor | Long and Feng | Study ID | Zhoufangmin(2012) | |
| **Domain** | **Signalling question** | | | **Response** |
| Bias arising from the randomization process | 1.1 Was the allocation sequence random? | | | Y |
| | 1.2 Was the allocation sequence concealed until participants were enrolled and assigned to interventions? | | | NI |
| | 1.3 Did baseline differences between intervention groups suggest a problem with the randomization process? | | | N |
| | Risk of bias judgement | | | Some concerns |
| Bias due to deviations from intended interventions | 2.1.Were participants aware of their assigned intervention during the trial? | | | Y |
| | 2.2.Were carers and people delivering the interventions aware of participants' assigned intervention during the trial? | | | Y |
| | 2.3. If Y/PY/NI to 2.1 or 2.2: Were there deviations from the intended intervention that arose because of the experimental context? | | | PN |
| | 2.4 If Y/PY to 2.3: Were these deviations likely to have affected the outcome? | | | NA |
| | 2.5. If Y/PY/NI to 2.4: Were these deviations from intended intervention balanced between groups? | | | NA |
| | 2.6 Was an appropriate analysis used to estimate the effect of assignment to intervention? | | | NI |
| | 2.7 If N/PN/NI to 2.6: Was there potential for a substantial impact (on the result) of the failure to analyse participants in the group to which they were randomized? | | | PN |
| | Risk of bias judgement | | | Some concerns |

| | | |
|---|---|---|
| Bias due to missing outcome data | 3.1 Were data for this outcome available for all, or nearly all, participants randomized? | Y |
| | 3.2 If N/PN/NI to 3.1: Is there evidence that result was not biased by missing outcome data? | NA |
| | 3.3 If N/PN to 3.2: Could missingness in the outcome depend on its true value? | NA |
| | 3.4 If Y/PY/NI to 3.3: Is it likely that missingness in the outcome depended on its true value? | NA |
| | Risk of bias judgement | Low |
| Bias in measurement of the outcome | 4.1 Was the method of measuring the outcome inappropriate? | N |
| | 4.2 Could measurement or ascertainment of the outcome have differed between intervention groups? | N |
| | 4.3 Were outcome assessors aware of the intervention received by study participants? | NI |
| | 4.4 If Y/PY/NI to 4.3: Could assessment of the outcome have been influenced by knowledge of intervention received? | PN |
| | 4.5 If Y/PY/NI to 4.4: Is it likely that assessment of the outcome was influenced by knowledge of intervention received? | NA |
| | Risk of bias judgement | Low |
| Bias in selection of the reported result | 5.1 Were the data that produced this result analysed in accordance with a pre-specified analysis plan that was finalized before unblinded outcome data were available for analysis? | NI |
| | 5.2 ... multiple eligible outcome measurements (e.g. scales, definitions, time points) within the outcome domain? | PN |
| | 5.3 ... multiple eligible analyses of the data? | PN |
| | Risk of bias judgement | Some concerns |
| Overall bias | Risk of bias judgement | Some concerns |

| | | | | |
|---|---|---|---|---|
| | | | | |
| | | | | |
| Assessor | Long and Feng | Study ID | Hanpeng(2013) | |

| Domain | Signalling question | Response |
|---|---|---|
| Bias arising from the randomization process | 1.1 Was the allocation sequence random? | N |
| | 1.2 Was the allocation sequence concealed until participants were enrolled and assigned to interventions? | NI |
| | 1.3 Did baseline differences between intervention groups suggest a problem with the randomization process? | N |
| | Risk of bias judgement | High |
| Bias due to deviations from intended interventions | 2.1.Were participants aware of their assigned intervention during the trial? | Y |
| | 2.2.Were carers and people delivering the interventions aware of participants' assigned intervention during the trial? | Y |
| | 2.3. If Y/PY/NI to 2.1 or 2.2: Were there deviations from the intended intervention that arose because of the experimental context? | PN |
| | 2.4 If Y/PY to 2.3: Were these deviations likely to have affected the outcome? | NA |
| | 2.5. If Y/PY/NI to 2.4: Were these deviations from intended intervention balanced between groups? | NA |
| | 2.6 Was an appropriate analysis used to estimate the effect of assignment to intervention? | NI |
| | 2.7 If N/PN/NI to 2.6: Was there potential for a substantial impact (on the result) of the failure to analyse participants in the group to which they were randomized? | PN |
| | Risk of bias judgement | Some concerns |
| Bias due to missing outcome data | 3.1 Were data for this outcome available for all, or nearly all, participants randomized? | Y |
| | 3.2 If N/PN/NI to 3.1: Is there evidence that result was not biased by missing outcome data? | NA |
| | 3.3 If N/PN to 3.2: Could missingness in the outcome depend on its true value? | NA |
| | 3.4 If Y/PY/NI to 3.3: Is it likely that missingness in the outcome depended on its true value? | NA |
| | Risk of bias judgement | Low |
| Bias in measurement of the outcome | 4.1 Was the method of measuring the outcome inappropriate? | N |
| | 4.2 Could measurement or ascertainment of the outcome have differed between intervention groups? | N |

| | | | | |
|---|---|---|---|---|
| | 4.3 Were outcome assessors aware of the intervention received by study participants? | | | PY |
| | 4.4 If Y/PY/NI to 4.3: Could assessment of the outcome have been influenced by knowledge of intervention received? | | | PY |
| | 4.5 If Y/PY/NI to 4.4: Is it likely that assessment of the outcome was influenced by knowledge of intervention received? | | | PN |
| | Risk of bias judgement | | | some concerns |
| Bias in selection of the reported result | 5.1 Were the data that produced this result analysed in accordance with a pre-specified analysis plan that was finalized before unblinded outcome data were available for analysis? | | | NI |
| | 5.2 ... multiple eligible outcome measurements (e.g. scales, definitions, time points) within the outcome domain? | | | PN |
| | 5.3 ... multiple eligible analyses of the data? | | | PN |
| | Risk of bias judgement | | | Some concerns |
| Overall bias | Risk of bias judgement | | | High |
| | | | | |
| | | | | |
| Assessor | Long and Feng | Study ID | Wangqun(2014) | |
| Domain | Signalling question | | | Response |
| Bias arising from the randomization process | 1.1 Was the allocation sequence random? | | | NI |
| | 1.2 Was the allocation sequence concealed until participants were enrolled and assigned to interventions? | | | NI |
| | 1.3 Did baseline differences between intervention groups suggest a problem with the randomization process? | | | N |
| | Risk of bias judgement | | | Some concerns |

| | | |
|---|---|---|
| **Bias due to deviations from intended interventions** | **2.1.Were participants aware of their assigned intervention during the trial?** | **NI** |
| | **2.2.Were carers and people delivering the interventions aware of participants' assigned intervention during the trial?** | **NI** |
| | **2.3. If Y/PY/NI to 2.1 or 2.2: Were there deviations from the intended intervention that arose because of the experimental context?** | **N** |
| | **2.4 If Y/PY to 2.3: Were these deviations likely to have affected the outcome?** | **NA** |
| | **2.5. If Y/PY/NI to 2.4: Were these deviations from intended intervention balanced between groups?** | **NA** |
| | **2.6 Was an appropriate analysis used to estimate the effect of assignment to intervention?** | **PY** |
| | **2.7 If N/PN/NI to 2.6: Was there potential for a substantial impact (on the result) of the failure to analyse participants in the group to which they were randomized?** | **NA** |
| | **Risk of bias judgement** | **Low** |
| **Bias due to missing outcome data** | **3.1 Were data for this outcome available for all, or nearly all, participants randomized?** | **PY** |
| | **3.2 If N/PN/NI to 3.1: Is there evidence that result was not biased by missing outcome data?** | **NA** |
| | **3.3 If N/PN to 3.2: Could missingness in the outcome depend on its true value?** | **NA** |
| | **3.4 If Y/PY/NI to 3.3: Is it likely that missingness in the outcome depended on its true value?** | **NA** |
| | **Risk of bias judgement** | **Low** |
| **Bias in measurement of the outcome** | **4.1 Was the method of measuring the outcome inappropriate?** | **NI** |
| | **4.2 Could measurement or ascertainment of the outcome have differed between intervention groups?** | **NI** |
| | **4.3 Were outcome assessors aware of the intervention received by study participants?** | **NI** |
| | **4.4 If Y/PY/NI to 4.3: Could assessment of the outcome have been influenced by knowledge of intervention received?** | **PN** |
| | **4.5 If Y/PY/NI to 4.4: Is it likely that assessment of the outcome was influenced by knowledge of intervention received?** | **NA** |
| | **Risk of bias judgement** | **Some concerns** |
| | **5.1 Were the data that produced this result analysed in accordance with a pre-specified analysis plan that was finalized before unblinded outcome data were available for analysis?** | **NI** |

| | | | | |
|---|---|---|---|---|
| Bias in selection of the reported result | 5.2 ... multiple eligible outcome measurements (e.g. scales, definitions, time points) within the outcome domain? | | | PN |
| | 5.3 ... multiple eligible analyses of the data? | | | N |
| | Risk of bias judgement | | | Some concerns |
| Overall bias | Risk of bias judgement | | | Some concerns |
| | | | | |
| | | | | |
| Assessor | Long and Feng | Study ID | Caiwenting(2014) | |

| Domain | Signalling question | Response |
|---|---|---|
| Bias arising from the randomization process | 1.1 Was the allocation sequence random? | PN |
| | 1.2 Was the allocation sequence concealed until participants were enrolled and assigned to interventions? | PN |
| | 1.3 Did baseline differences between intervention groups suggest a problem with the randomization process? | PY |
| | Risk of bias judgement | High |
| Bias due to deviations from intended interventions | 2.1.Were participants aware of their assigned intervention during the trial? | NI |
| | 2.2.Were carers and people delivering the interventions aware of participants' assigned intervention during the trial? | NI |
| | 2.3. If Y/PY/NI to 2.1 or 2.2: Were there deviations from the intended intervention that arose because of the experimental context? | PN |
| | 2.4 If Y/PY to 2.3: Were these deviations likely to have affected the outcome? | NA |
| | 2.5. If Y/PY/NI to 2.4: Were these deviations from intended intervention balanced between groups? | NA |
| | 2.6 Was an appropriate analysis used to estimate the effect of assignment to intervention? | PY |

| | | | | |
|---|---|---|---|---|
| | 2.7 If N/PN/NI to 2.6: Was there potential for a substantial impact (on the result) of the failure to analyse participants in the group to which they were randomized? | | | NA |
| | Risk of bias judgement | | | Low |
| Bias due to missing outcome data | 3.1 Were data for this outcome available for all, or nearly all, participants randomized? | | | PN |
| | 3.2 If N/PN/NI to 3.1: Is there evidence that result was not biased by missing outcome data? | | | PN |
| | 3.3 If N/PN to 3.2: Could missingness in the outcome depend on its true value? | | | NI |
| | 3.4 If Y/PY/NI to 3.3: Is it likely that missingness in the outcome depended on its true value? | | | NI |
| | Risk of bias judgement | | | High |
| Bias in measurement of the outcome | 4.1 Was the method of measuring the outcome inappropriate? | | | NI |
| | 4.2 Could measurement or ascertainment of the outcome have differed between intervention groups? | | | PN |
| | 4.3 Were outcome assessors aware of the intervention received by study participants? | | | NI |
| | 4.4 If Y/PY/NI to 4.3: Could assessment of the outcome have been influenced by knowledge of intervention received? | | | PN |
| | 4.5 If Y/PY/NI to 4.4: Is it likely that assessment of the outcome was influenced by knowledge of intervention received? | | | NA |
| | Risk of bias judgement | | | Low |
| Bias in selection of the reported result | 5.1 Were the data that produced this result analysed in accordance with a pre-specified analysis plan that was finalized before unblinded outcome data were available for analysis? | | | NI |
| | 5.2 ... multiple eligible outcome measurements (e.g. scales, definitions, time points) within the outcome domain? | | | PN |
| | 5.3 ... multiple eligible analyses of the data? | | | NI |
| | Risk of bias judgement | | | Some concerns |
| Overall bias | Risk of bias judgement | | | High |
| | | | | |
| | | | | |
| Assessor | Long and Feng | Study ID | Hemingwu(2015) | |

| Domain | Signalling question | Response |
|---|---|---|
| Bias arising from the randomization process | 1.1 Was the allocation sequence random? | NI |
| | 1.2 Was the allocation sequence concealed until participants were enrolled and assigned to interventions? | NI |
| | 1.3 Did baseline differences between intervention groups suggest a problem with the randomization process? | PN |
| | Risk of bias judgement | Some concerns |
| Bias due to deviations from intended interventions | 2.1.Were participants aware of their assigned intervention during the trial? | PY |
| | 2.2.Were carers and people delivering the interventions aware of participants' assigned intervention during the trial? | PY |
| | 2.3. If Y/PY/NI to 2.1 or 2.2: Were there deviations from the intended intervention that arose because of the experimental context? | PN |
| | 2.4 If Y/PY to 2.3: Were these deviations likely to have affected the outcome? | NA |
| | 2.5. If Y/PY/NI to 2.4: Were these deviations from intended intervention balanced between groups? | NA |
| | 2.6 Was an appropriate analysis used to estimate the effect of assignment to intervention? | PY |
| | 2.7 If N/PN/NI to 2.6: Was there potential for a substantial impact (on the result) of the failure to analyse participants in the group to which they were randomized? | NA |
| | Risk of bias judgement | Low |
| Bias due to missing outcome data | 3.1 Were data for this outcome available for all, or nearly all, participants randomized? | PY |
| | 3.2 If N/PN/NI to 3.1: Is there evidence that result was not biased by missing outcome data? | NA |
| | 3.3 If N/PN to 3.2: Could missingness in the outcome depend on its true value? | NA |
| | 3.4 If Y/PY/NI to 3.3: Is it likely that missingness in the outcome depended on its true value? | NA |
| | Risk of bias judgement | Low |
| Bias in measurement of the outcome | 4.1 Was the method of measuring the outcome inappropriate? | PN |
| | 4.2 Could measurement or ascertainment of the outcome have differed between intervention groups? | PN |

| | | | | |
|---|---|---|---|---|
| | 4.3 Were outcome assessors aware of the intervention received by study participants? | | | NI |
| | 4.4 If Y/PY/NI to 4.3: Could assessment of the outcome have been influenced by knowledge of intervention received? | | | NI |
| | 4.5 If Y/PY/NI to 4.4: Is it likely that assessment of the outcome was influenced by knowledge of intervention received? | | | NI |
| | Risk of bias judgement | | | High |
| Bias in selection of the reported result | 5.1 Were the data that produced this result analysed in accordance with a pre-specified analysis plan that was finalized before unblinded outcome data were available for analysis? | | | NI |
| | 5.2 ... multiple eligible outcome measurements (e.g. scales, definitions, time points) within the outcome domain? | | | PN |
| | 5.3 ... multiple eligible analyses of the data? | | | PN |
| | Risk of bias judgement | | | Some concerns |
| Overall bias | Risk of bias judgement | | | High |
| | | | | |
| | | | | |
| Assessor | Long and Feng | Study ID | Liugang(2015) | |
| **Domain** | **Signalling question** | | | **Response** |
| Bias arising from the randomization process | 1.1 Was the allocation sequence random? | | | NI |
| | 1.2 Was the allocation sequence concealed until participants were enrolled and assigned to interventions? | | | NI |
| | 1.3 Did baseline differences between intervention groups suggest a problem with the randomization process? | | | N |
| | Risk of bias judgement | | | Some concerns |
| | 2.1.Were participants aware of their assigned intervention during the trial? | | | PY |

| | | |
|---|---|---|
| Bias due to deviations from intended interventions | 2.2.Were carers and people delivering the interventions aware of participants' assigned intervention during the trial? | PY |
| | 2.3. If Y/PY/NI to 2.1 or 2.2: Were there deviations from the intended intervention that arose because of the experimental context? | PN |
| | 2.4 If Y/PY to 2.3: Were these deviations likely to have affected the outcome? | NA |
| | 2.5. If Y/PY/NI to 2.4: Were these deviations from intended intervention balanced between groups? | NA |
| | 2.6 Was an appropriate analysis used to estimate the effect of assignment to intervention? | Y |
| | 2.7 If N/PN/NI to 2.6: Was there potential for a substantial impact (on the result) of the failure to analyse participants in the group to which they were randomized? | NA |
| | Risk of bias judgement | Low |
| Bias due to missing outcome data | 3.1 Were data for this outcome available for all, or nearly all, participants randomized? | PY |
| | 3.2 If N/PN/NI to 3.1: Is there evidence that result was not biased by missing outcome data? | NA |
| | 3.3 If N/PN to 3.2: Could missingness in the outcome depend on its true value? | NA |
| | 3.4 If Y/PY/NI to 3.3: Is it likely that missingness in the outcome depended on its true value? | NA |
| | Risk of bias judgement | Low |
| Bias in measurement of the outcome | 4.1 Was the method of measuring the outcome inappropriate? | PN |
| | 4.2 Could measurement or ascertainment of the outcome have differed between intervention groups? | NI |
| | 4.3 Were outcome assessors aware of the intervention received by study participants? | PY |
| | 4.4 If Y/PY/NI to 4.3: Could assessment of the outcome have been influenced by knowledge of intervention received? | PY |
| | 4.5 If Y/PY/NI to 4.4: Is it likely that assessment of the outcome was influenced by knowledge of intervention received? | PN |
| | Risk of bias judgement | Low |
| Bias in selection of the reported result | 5.1 Were the data that produced this result analysed in accordance with a pre-specified analysis plan that was finalized before unblinded outcome data were available for analysis? | PN |
| | 5.2 ... multiple eligible outcome measurements (e.g. scales, definitions, time points) within the outcome domain? | NI |
| | 5.3 ... multiple eligible analyses of the data? | PN |

| | | | | |
|---|---|---|---|---|
| | **Risk of bias judgement** | | | **Some concerns** |
| **Overall bias** | **Risk of bias judgement** | | | **Some concerns** |
| | | | | |
| | | | | |
| **Assessor** | **Long and Feng** | **Study ID** | **Liuying(2017)** | |
| **Domain** | **Signalling question** | | | **Response** |
| **Bias arising from the randomization process** | **1.1 Was the allocation sequence random?** | | | **Y** |
| | **1.2 Was the allocation sequence concealed until participants were enrolled and assigned to interventions?** | | | **NI** |
| | **1.3 Did baseline differences between intervention groups suggest a problem with the randomization process?** | | | **N** |
| | **Risk of bias judgement** | | | **Some concerns** |
| **Bias due to deviations from intended interventions** | **2.1.Were participants aware of their assigned intervention during the trial?** | | | **PY** |
| | **2.2.Were carers and people delivering the interventions aware of participants' assigned intervention during the trial?** | | | **PY** |
| | **2.3. If Y/PY/NI to 2.1 or 2.2: Were there deviations from the intended intervention that arose because of the experimental context?** | | | **N** |
| | **2.4 If Y/PY to 2.3: Were these deviations likely to have affected the outcome?** | | | **NA** |
| | **2.5. If Y/PY/NI to 2.4: Were these deviations from intended intervention balanced between groups?** | | | **NA** |
| | **2.6 Was an appropriate analysis used to estimate the effect of assignment to intervention?** | | | **Y** |
| | **2.7 If N/PN/NI to 2.6: Was there potential for a substantial impact (on the result) of the failure to analyse participants in the group to which they were randomized?** | | | **NA** |
| | **Risk of bias judgement** | | | **Low** |

| | | |
|---|---|---|
| Bias due to missing outcome data | 3.1 Were data for this outcome available for all, or nearly all, participants randomized? | Y |
| | 3.2 If N/PN/NI to 3.1: Is there evidence that result was not biased by missing outcome data? | NA |
| | 3.3 If N/PN to 3.2: Could missingness in the outcome depend on its true value? | NA |
| | 3.4 If Y/PY/NI to 3.3: Is it likely that missingness in the outcome depended on its true value? | NA |
| | Risk of bias judgement | Low |
| Bias in measurement of the outcome | 4.1 Was the method of measuring the outcome inappropriate? | PN |
| | 4.2 Could measurement or ascertainment of the outcome have differed between intervention groups? | NI |
| | 4.3 Were outcome assessors aware of the intervention received by study participants? | PY |
| | 4.4 If Y/PY/NI to 4.3: Could assessment of the outcome have been influenced by knowledge of intervention received? | PY |
| | 4.5 If Y/PY/NI to 4.4: Is it likely that assessment of the outcome was influenced by knowledge of intervention received? | PN |
| | Risk of bias judgement | Some concerns |
| Bias in selection of the reported result | 5.1 Were the data that produced this result analysed in accordance with a pre-specified analysis plan that was finalized before unblinded outcome data were available for analysis? | NI |
| | 5.2 ... multiple eligible outcome measurements (e.g. scales, definitions, time points) within the outcome domain? | NI |
| | 5.3 ... multiple eligible analyses of the data? | NI |
| | Risk of bias judgement | Some concerns |
| Overall bias | Risk of bias judgement | Some concerns |

| | | | | |
|---|---|---|---|---|
| | | | | |
| | | | | |
| Assessor | Long and Feng | Study ID | Zhangyarong(2017) | |

| Domain | Signalling question | Response |
|---|---|---|
| Bias arising from the randomization process | 1.1 Was the allocation sequence random? | N |
| | 1.2 Was the allocation sequence concealed until participants were enrolled and assigned to interventions? | PN |
| | 1.3 Did baseline differences between intervention groups suggest a problem with the randomization process? | PY |
| | Risk of bias judgement | High |
| Bias due to deviations from intended interventions | 2.1.Were participants aware of their assigned intervention during the trial? | NI |
| | 2.2.Were carers and people delivering the interventions aware of participants' assigned intervention during the trial? | NI |
| | 2.3. If Y/PY/NI to 2.1 or 2.2: Were there deviations from the intended intervention that arose because of the experimental context? | NI |
| | 2.4 If Y/PY to 2.3: Were these deviations likely to have affected the outcome? | NA |
| | 2.5. If Y/PY/NI to 2.4: Were these deviations from intended intervention balanced between groups? | NA |
| | 2.6 Was an appropriate analysis used to estimate the effect of assignment to intervention? | PY |
| | 2.7 If N/PN/NI to 2.6: Was there potential for a substantial impact (on the result) of the failure to analyse participants in the group to which they were randomized? | NA |
| | Risk of bias judgement | Low |
| Bias due to missing outcome data | 3.1 Were data for this outcome available for all, or nearly all, participants randomized? | NI |
| | 3.2 If N/PN/NI to 3.1: Is there evidence that result was not biased by missing outcome data? | N |
| | 3.3 If N/PN to 3.2: Could missingness in the outcome depend on its true value? | NI |
| | 3.4 If Y/PY/NI to 3.3: Is it likely that missingness in the outcome depended on its true value? | NI |
| | Risk of bias judgement | High |
| Bias in measurement of the outcome | 4.1 Was the method of measuring the outcome inappropriate? | PN |
| | 4.2 Could measurement or ascertainment of the outcome have differed between intervention groups? | N |
| | 4.3 Were outcome assessors aware of the intervention received by study participants? | NI |

| | | | | |
|---|---|---|---|---|
| | 4.4 If Y/PY/NI to 4.3: Could assessment of the outcome have been influenced by knowledge of intervention received? | | | NI |
| | 4.5 If Y/PY/NI to 4.4: Is it likely that assessment of the outcome was influenced by knowledge of intervention received? | | | NI |
| | Risk of bias judgement | | | High |
| Bias in selection of the reported result | 5.1 Were the data that produced this result analysed in accordance with a pre-specified analysis plan that was finalized before unblinded outcome data were available for analysis? | | | NI |
| | 5.2 ... multiple eligible outcome measurements (e.g. scales, definitions, time points) within the outcome domain? | | | NI |
| | 5.3 ... multiple eligible analyses of the data? | | | NI |
| | Risk of bias judgement | | | High |
| Overall bias | Risk of bias judgement | | | High |
| | | | | |
| | | | | |
| Assessor | Long and Feng | Study ID | Zhongchao(2017) | |
| **Domain** | **Signalling question** | | | **Response** |
| Bias arising from the randomization process | 1.1 Was the allocation sequence random? | | | Y |
| | 1.2 Was the allocation sequence concealed until participants were enrolled and assigned to interventions? | | | NI |
| | 1.3 Did baseline differences between intervention groups suggest a problem with the randomization process? | | | PN |
| | Risk of bias judgement | | | Some concerns |
| | 2.1.Were participants aware of their assigned intervention during the trial? | | | NI |
| | 2.2.Were carers and people delivering the interventions aware of participants' assigned intervention during the trial? | | | NI |

| | | |
|---|---|---|
| **Bias due to deviations from intended interventions** | **2.3. If Y/PY/NI to 2.1 or 2.2: Were there deviations from the intended intervention that arose because of the experimental context?** | **PN** |
| | **2.4 If Y/PY to 2.3: Were these deviations likely to have affected the outcome?** | **NA** |
| | **2.5. If Y/PY/NI to 2.4: Were these deviations from intended intervention balanced between groups?** | **NA** |
| | **2.6 Was an appropriate analysis used to estimate the effect of assignment to intervention?** | **Y** |
| | **2.7 If N/PN/NI to 2.6: Was there potential for a substantial impact (on the result) of the failure to analyse participants in the group to which they were randomized?** | **NA** |
| | **Risk of bias judgement** | **Low** |
| **Bias due to missing outcome data** | **3.1 Were data for this outcome available for all, or nearly all, participants randomized?** | **PY** |
| | **3.2 If N/PN/NI to 3.1: Is there evidence that result was not biased by missing outcome data?** | **NA** |
| | **3.3 If N/PN to 3.2: Could missingness in the outcome depend on its true value?** | **NA** |
| | **3.4 If Y/PY/NI to 3.3: Is it likely that missingness in the outcome depended on its true value?** | **NA** |
| | **Risk of bias judgement** | **Low** |
| **Bias in measurement of the outcome** | **4.1 Was the method of measuring the outcome inappropriate?** | **PN** |
| | **4.2 Could measurement or ascertainment of the outcome have differed between intervention groups?** | **PN** |
| | **4.3 Were outcome assessors aware of the intervention received by study participants?** | **NI** |
| | **4.4 If Y/PY/NI to 4.3: Could assessment of the outcome have been influenced by knowledge of intervention received?** | **PN** |
| | **4.5 If Y/PY/NI to 4.4: Is it likely that assessment of the outcome was influenced by knowledge of intervention received?** | **NA** |
| | **Risk of bias judgement** | **Low** |
| **Bias in selection of the reported result** | **5.1 Were the data that produced this result analysed in accordance with a pre-specified analysis plan that was finalized before unblinded outcome data were available for analysis?** | **NI** |
| | **5.2 ... multiple eligible outcome measurements (e.g. scales, definitions, time points) within the outcome domain?** | **PN** |
| | **5.3 ... multiple eligible analyses of the data?** | **PN** |
| | **Risk of bias judgement** | **Some concerns** |

| | | | | |
|---|---|---|---|---|
| **Overall bias** | **Risk of bias judgement** | | | **Some concerns** |
| | | | | |
| | | | | |
| **Assessor** | **Long and Feng** | **Study ID** | Chenxuan(2018) | |
| **Domain** | **Signalling question** | | | **Response** |
| **Bias arising from the randomization process** | **1.1 Was the allocation sequence random?** | | | **NI** |
| | **1.2 Was the allocation sequence concealed until participants were enrolled and assigned to interventions?** | | | **NI** |
| | **1.3 Did baseline differences between intervention groups suggest a problem with the randomization process?** | | | **N** |
| | **Risk of bias judgement** | | | **Some concerns** |
| **Bias due to deviations from intended interventions** | **2.1.Were participants aware of their assigned intervention during the trial?** | | | **PY** |
| | **2.2.Were carers and people delivering the interventions aware of participants' assigned intervention during the trial?** | | | **PY** |
| | **2.3. If Y/PY/NI to 2.1 or 2.2: Were there deviations from the intended intervention that arose because of the experimental context?** | | | **N** |
| | **2.4 If Y/PY to 2.3: Were these deviations likely to have affected the outcome?** | | | **NA** |
| | **2.5. If Y/PY/NI to 2.4: Were these deviations from intended intervention balanced between groups?** | | | **NA** |
| | **2.6 Was an appropriate analysis used to estimate the effect of assignment to intervention?** | | | **PY** |
| | **2.7 If N/PN/NI to 2.6: Was there potential for a substantial impact (on the result) of the failure to analyse participants in the group to which they were randomized?** | | | **NA** |
| | **Risk of bias judgement** | | | **Low** |
| **Bias due to missing outcome data** | **3.1 Were data for this outcome available for all, or nearly all, participants randomized?** | | | **PY** |
| | **3.2 If N/PN/NI to 3.1: Is there evidence that result was not biased by missing outcome data?** | | | **NA** |

| | | | | |
|---|---|---|---|---|
| | 3.3 If N/PN to 3.2: Could missingness in the outcome depend on its true value? | | | NA |
| | 3.4 If Y/PY/NI to 3.3: Is it likely that missingness in the outcome depended on its true value? | | | NA |
| | Risk of bias judgement | | | Low |
| Bias in measurement of the outcome | 4.1 Was the method of measuring the outcome inappropriate? | | | PN |
| | 4.2 Could measurement or ascertainment of the outcome have differed between intervention groups? | | | PN |
| | 4.3 Were outcome assessors aware of the intervention received by study participants? | | | NI |
| | 4.4 If Y/PY/NI to 4.3: Could assessment of the outcome have been influenced by knowledge of intervention received? | | | PN |
| | 4.5 If Y/PY/NI to 4.4: Is it likely that assessment of the outcome was influenced by knowledge of intervention received? | | | NA |
| | Risk of bias judgement | | | Low |
| Bias in selection of the reported result | 5.1 Were the data that produced this result analysed in accordance with a pre-specified analysis plan that was finalized before unblinded outcome data were available for analysis? | | | NI |
| | 5.2 ... multiple eligible outcome measurements (e.g. scales, definitions, time points) within the outcome domain? | | | PN |
| | 5.3 ... multiple eligible analyses of the data? | | | PN |
| | Risk of bias judgement | | | Some concerns |
| Overall bias | Risk of bias judgement | | | Some concerns |
| | | | | |
| | | | | |
| Assessor | Long and Feng | Study ID | Liangliang(2018) | |
| **Domain** | **Signalling question** | | | **Response** |
| | 1.1 Was the allocation sequence random? | | | NI |

| | | |
|---|---|---|
| **Bias arising from the randomization process** | **1.2 Was the allocation sequence concealed until participants were enrolled and assigned to interventions?** | **NI** |
| | **1.3 Did baseline differences between intervention groups suggest a problem with the randomization process?** | **N** |
| | **Risk of bias judgement** | **Some concerns** |
| **Bias due to deviations from intended interventions** | **2.1.Were participants aware of their assigned intervention during the trial?** | **PY** |
| | **2.2.Were carers and people delivering the interventions aware of participants' assigned intervention during the trial?** | **PY** |
| | **2.3. If Y/PY/NI to 2.1 or 2.2: Were there deviations from the intended intervention that arose because of the experimental context?** | **PN** |
| | **2.4 If Y/PY to 2.3: Were these deviations likely to have affected the outcome?** | **NA** |
| | **2.5. If Y/PY/NI to 2.4: Were these deviations from intended intervention balanced between groups?** | **NA** |
| | **2.6 Was an appropriate analysis used to estimate the effect of assignment to intervention?** | **Y** |
| | **2.7 If N/PN/NI to 2.6: Was there potential for a substantial impact (on the result) of the failure to analyse participants in the group to which they were randomized?** | **NA** |
| | **Risk of bias judgement** | **Some concerns** |
| **Bias due to missing outcome data** | **3.1 Were data for this outcome available for all, or nearly all, participants randomized?** | **PY** |
| | **3.2 If N/PN/NI to 3.1: Is there evidence that result was not biased by missing outcome data?** | **NA** |
| | **3.3 If N/PN to 3.2: Could missingness in the outcome depend on its true value?** | **NA** |
| | **3.4 If Y/PY/NI to 3.3: Is it likely that missingness in the outcome depended on its true value?** | **NA** |
| | **Risk of bias judgement** | **Low** |
| **Bias in measurement of the outcome** | **4.1 Was the method of measuring the outcome inappropriate?** | **NI** |
| | **4.2 Could measurement or ascertainment of the outcome have differed between intervention groups?** | **PN** |
| | **4.3 Were outcome assessors aware of the intervention received by study participants?** | **NI** |
| | **4.4 If Y/PY/NI to 4.3: Could assessment of the outcome have been influenced by knowledge of intervention received?** | **NI** |

| | | | | |
|---|---|---|---|---|
| | 4.5 If Y/PY/NI to 4.4: Is it likely that assessment of the outcome was influenced by knowledge of intervention received? | | | NI |
| | Risk of bias judgement | | | High |
| Bias in selection of the reported result | 5.1 Were the data that produced this result analysed in accordance with a pre-specified analysis plan that was finalized before unblinded outcome data were available for analysis? | | | NI |
| | 5.2 ... multiple eligible outcome measurements (e.g. scales, definitions, time points) within the outcome domain? | | | NI |
| | 5.3 ... multiple eligible analyses of the data? | | | NI |
| | Risk of bias judgement | | | Some concerns |
| Overall bias | Risk of bias judgement | | | High |
| | | | | |
| | | | | |
| Assessor | Long and Feng | Study ID | Wanganwen(2018) | |
| Domain | Signalling question | | | Response |
| Bias arising from the randomization process | 1.1 Was the allocation sequence random? | | | NI |
| | 1.2 Was the allocation sequence concealed until participants were enrolled and assigned to interventions? | | | NI |
| | 1.3 Did baseline differences between intervention groups suggest a problem with the randomization process? | | | PN |
| | Risk of bias judgement | | | Some concerns |
| Bias due to deviations from intended interventions | 2.1.Were participants aware of their assigned intervention during the trial? | | | PY |
| | 2.2.Were carers and people delivering the interventions aware of participants' assigned intervention during the trial? | | | PY |
| | 2.3. If Y/PY/NI to 2.1 or 2.2: Were there deviations from the intended intervention that arose because of the experimental context? | | | PN |

| | | |
|---|---|---|
| | 2.4 If Y/PY to 2.3: Were these deviations likely to have affected the outcome? | NA |
| | 2.5. If Y/PY/NI to 2.4: Were these deviations from intended intervention balanced between groups? | NA |
| | 2.6 Was an appropriate analysis used to estimate the effect of assignment to intervention? | PY |
| | 2.7 If N/PN/NI to 2.6: Was there potential for a substantial impact (on the result) of the failure to analyse participants in the group to which they were randomized? | NA |
| | **Risk of bias judgement** | Low |
| **Bias due to missing outcome data** | 3.1 Were data for this outcome available for all, or nearly all, participants randomized? | NI |
| | 3.2 If N/PN/NI to 3.1: Is there evidence that result was not biased by missing outcome data? | PN |
| | 3.3 If N/PN to 3.2: Could missingness in the outcome depend on its true value? | NI |
| | 3.4 If Y/PY/NI to 3.3: Is it likely that missingness in the outcome depended on its true value? | PN |
| | **Risk of bias judgement** | Some concerns |
| **Bias in measurement of the outcome** | 4.1 Was the method of measuring the outcome inappropriate? | PN |
| | 4.2 Could measurement or ascertainment of the outcome have differed between intervention groups? | PN |
| | 4.3 Were outcome assessors aware of the intervention received by study participants? | PY |
| | 4.4 If Y/PY/NI to 4.3: Could assessment of the outcome have been influenced by knowledge of intervention received? | PN |
| | 4.5 If Y/PY/NI to 4.4: Is it likely that assessment of the outcome was influenced by knowledge of intervention received? | NA |
| | **Risk of bias judgement** | Low |
| **Bias in selection of the reported result** | 5.1 Were the data that produced this result analysed in accordance with a pre-specified analysis plan that was finalized before unblinded outcome data were available for analysis? | NI |
| | 5.2 ... multiple eligible outcome measurements (e.g. scales, definitions, time points) within the outcome domain? | PN |
| | 5.3 ... multiple eligible analyses of the data? | PN |
| | **Risk of bias judgement** | Some concerns |

| | | | | |
|---|---|---|---|---|
| Overall bias | Risk of bias judgement | | | Some concerns |
| | | | | |
| | | | | |
| PGE1+DCI | | | | |
| Assessor | Long and Feng | Study ID | Yangxu(2015) | |
| **Domain** | **Signalling question** | | | **Response** |
| Bias arising from the randomization process | 1.1 Was the allocation sequence random? | | | NI |
| | 1.2 Was the allocation sequence concealed until participants were enrolled and assigned to interventions? | | | NI |
| | 1.3 Did baseline differences between intervention groups suggest a problem with the randomization process? | | | PN |
| | Risk of bias judgement | | | Some concerns |
| Bias due to deviations from intended interventions | 2.1.Were participants aware of their assigned intervention during the trial? | | | NI |
| | 2.2.Were carers and people delivering the interventions aware of participants' assigned intervention during the trial? | | | NI |
| | 2.3. If Y/PY/NI to 2.1 or 2.2: Were there deviations from the intended intervention that arose because of the experimental context? | | | NI |
| | 2.4 If Y/PY to 2.3: Were these deviations likely to have affected the outcome? | | | NA |
| | 2.5. If Y/PY/NI to 2.4: Were these deviations from intended intervention balanced between groups? | | | NA |
| | 2.6 Was an appropriate analysis used to estimate the effect of assignment to intervention? | | | PY |
| | 2.7 If N/PN/NI to 2.6: Was there potential for a substantial impact (on the result) of the failure to analyse participants in the group to which they were randomized? | | | NA |
| | Risk of bias judgement | | | Some concerns |
| | 3.1 Were data for this outcome available for all, or nearly all, participants randomized? | | | NI |

| | | |
|---|---|---|
| Bias due to missing outcome data | 3.2 If N/PN/NI to 3.1: Is there evidence that result was not biased by missing outcome data? | N |
| | 3.3 If N/PN to 3.2: Could missingness in the outcome depend on its true value? | NI |
| | 3.4 If Y/PY/NI to 3.3: Is it likely that missingness in the outcome depended on its true value? | PN |
| | Risk of bias judgement | Some concerns |
| Bias in measurement of the outcome | 4.1 Was the method of measuring the outcome inappropriate? | PN |
| | 4.2 Could measurement or ascertainment of the outcome have differed between intervention groups? | PN |
| | 4.3 Were outcome assessors aware of the intervention received by study participants? | PY |
| | 4.4 If Y/PY/NI to 4.3: Could assessment of the outcome have been influenced by knowledge of intervention received? | PN |
| | 4.5 If Y/PY/NI to 4.4: Is it likely that assessment of the outcome was influenced by knowledge of intervention received? | NA |
| | Risk of bias judgement | Low |
| Bias in selection of the reported result | 5.1 Were the data that produced this result analysed in accordance with a pre-specified analysis plan that was finalized before unblinded outcome data were available for analysis? | NI |
| | 5.2 ... multiple eligible outcome measurements (e.g. scales, definitions, time points) within the outcome domain? | PN |
| | 5.3 ... multiple eligible analyses of the data? | PN |
| | Risk of bias judgement | Some concerns |
| Overall bias | Risk of bias judgement | Some concerns |

| | | | | |
|---|---|---|---|---|
| | | | | |
| | | | | |
| Assessor | Long and Feng | Study ID | Zhangyin(2016) | |

| Domain | Signalling question | Response |
|---|---|---|
| Bias arising from the randomization process | 1.1 Was the allocation sequence random? | Y |
| | 1.2 Was the allocation sequence concealed until participants were enrolled and assigned to interventions? | NI |
| | 1.3 Did baseline differences between intervention groups suggest a problem with the randomization process? | PN |
| | Risk of bias judgement | Some concerns |
| Bias due to deviations from intended interventions | 2.1.Were participants aware of their assigned intervention during the trial? | PY |
| | 2.2.Were carers and people delivering the interventions aware of participants' assigned intervention during the trial? | PY |
| | 2.3. If Y/PY/NI to 2.1 or 2.2: Were there deviations from the intended intervention that arose because of the experimental context? | NI |
| | 2.4 If Y/PY to 2.3: Were these deviations likely to have affected the outcome? | NA |
| | 2.5. If Y/PY/NI to 2.4: Were these deviations from intended intervention balanced between groups? | NA |
| | 2.6 Was an appropriate analysis used to estimate the effect of assignment to intervention? | Y |
| | 2.7 If N/PN/NI to 2.6: Was there potential for a substantial impact (on the result) of the failure to analyse participants in the group to which they were randomized? | NA |
| | Risk of bias judgement | Low |
| Bias due to missing outcome data | 3.1 Were data for this outcome available for all, or nearly all, participants randomized? | PY |
| | 3.2 If N/PN/NI to 3.1: Is there evidence that result was not biased by missing outcome data? | NA |
| | 3.3 If N/PN to 3.2: Could missingness in the outcome depend on its true value? | NA |
| | 3.4 If Y/PY/NI to 3.3: Is it likely that missingness in the outcome depended on its true value? | NA |
| | Risk of bias judgement | Low |
| Bias in measurement of the outcome | 4.1 Was the method of measuring the outcome inappropriate? | PN |
| | 4.2 Could measurement or ascertainment of the outcome have differed between intervention groups? | PN |

| | | | | |
|---|---|---|---|---|
| | 4.3 Were outcome assessors aware of the intervention received by study participants? | | | PY |
| | 4.4 If Y/PY/NI to 4.3: Could assessment of the outcome have been influenced by knowledge of intervention received? | | | PY |
| | 4.5 If Y/PY/NI to 4.4: Is it likely that assessment of the outcome was influenced by knowledge of intervention received? | | | PN |
| | Risk of bias judgement | | | Some concerns |
| Bias in selection of the reported result | 5.1 Were the data that produced this result analysed in accordance with a pre-specified analysis plan that was finalized before unblinded outcome data were available for analysis? | | | NI |
| | 5.2 ... multiple eligible outcome measurements (e.g. scales, definitions, time points) within the outcome domain? | | | NI |
| | 5.3 ... multiple eligible analyses of the data? | | | NI |
| | Risk of bias judgement | | | Some concerns |
| Overall bias | Risk of bias judgement | | | Some concerns |
| | | | | |
| | | | | |
| Assessor | Long and Feng | Study ID | Cuiyi(2017) | |
| **Domain** | **Signalling question** | | | **Response** |
| Bias arising from the randomization process | 1.1 Was the allocation sequence random? | | | PN |
| | 1.2 Was the allocation sequence concealed until participants were enrolled and assigned to interventions? | | | PN |
| | 1.3 Did baseline differences between intervention groups suggest a problem with the randomization process? | | | PN |
| | Risk of bias judgement | | | High |

| | | |
|---|---|---|
| **Bias due to deviations from intended interventions** | **2.1.Were participants aware of their assigned intervention during the trial?** | **PY** |
| | **2.2.Were carers and people delivering the interventions aware of participants' assigned intervention during the trial?** | **PY** |
| | **2.3. If Y/PY/NI to 2.1 or 2.2: Were there deviations from the intended intervention that arose because of the experimental context?** | **N** |
| | **2.4 If Y/PY to 2.3: Were these deviations likely to have affected the outcome?** | **NA** |
| | **2.5. If Y/PY/NI to 2.4: Were these deviations from intended intervention balanced between groups?** | **NA** |
| | **2.6 Was an appropriate analysis used to estimate the effect of assignment to intervention?** | **PY** |
| | **2.7 If N/PN/NI to 2.6: Was there potential for a substantial impact (on the result) of the failure to analyse participants in the group to which they were randomized?** | **NA** |
| | **Risk of bias judgement** | **Low** |
| **Bias due to missing outcome data** | **3.1 Were data for this outcome available for all, or nearly all, participants randomized?** | **PY** |
| | **3.2 If N/PN/NI to 3.1: Is there evidence that result was not biased by missing outcome data?** | **NA** |
| | **3.3 If N/PN to 3.2: Could missingness in the outcome depend on its true value?** | **NA** |
| | **3.4 If Y/PY/NI to 3.3: Is it likely that missingness in the outcome depended on its true value?** | **NA** |
| | **Risk of bias judgement** | **Low** |
| **Bias in measurement of the outcome** | **4.1 Was the method of measuring the outcome inappropriate?** | **NI** |
| | **4.2 Could measurement or ascertainment of the outcome have differed between intervention groups?** | **NI** |
| | **4.3 Were outcome assessors aware of the intervention received by study participants?** | **PY** |
| | **4.4 If Y/PY/NI to 4.3: Could assessment of the outcome have been influenced by knowledge of intervention received?** | **N** |
| | **4.5 If Y/PY/NI to 4.4: Is it likely that assessment of the outcome was influenced by knowledge of intervention received?** | **NA** |
| | **Risk of bias judgement** | **Some concerns** |
| | **5.1 Were the data that produced this result analysed in accordance with a pre-specified analysis plan that was finalized before unblinded outcome data were available for analysis?** | **NI** |

| | | | | |
|---|---|---|---|---|
| Bias in selection of the reported result | 5.2 ... multiple eligible outcome measurements (e.g. scales, definitions, time points) within the outcome domain? | | | PN |
| | 5.3 ... multiple eligible analyses of the data? | | | PN |
| | Risk of bias judgement | | | Some concerns |
| Overall bias | Risk of bias judgement | | | Some concerns |
| | | | | |
| | | | | |
| Assessor | Long and Feng | Study ID | Maigaoyang(2017) | |
| **Domain** | **Signalling question** | | | **Response** |
| Bias arising from the randomization process | 1.1 Was the allocation sequence random? | | | Y |
| | 1.2 Was the allocation sequence concealed until participants were enrolled and assigned to interventions? | | | NI |
| | 1.3 Did baseline differences between intervention groups suggest a problem with the randomization process? | | | N |
| | Risk of bias judgement | | | Some concerns |
| Bias due to deviations from intended interventions | 2.1.Were participants aware of their assigned intervention during the trial? | | | PY |
| | 2.2.Were carers and people delivering the interventions aware of participants' assigned intervention during the trial? | | | PY |
| | 2.3. If Y/PY/NI to 2.1 or 2.2: Were there deviations from the intended intervention that arose because of the experimental context? | | | PN |
| | 2.4 If Y/PY to 2.3: Were these deviations likely to have affected the outcome? | | | NA |
| | 2.5. If Y/PY/NI to 2.4: Were these deviations from intended intervention balanced between groups? | | | NA |
| | 2.6 Was an appropriate analysis used to estimate the effect of assignment to intervention? | | | PY |

| | | | | |
|---|---|---|---|---|
| | 2.7 If N/PN/NI to 2.6: Was there potential for a substantial impact (on the result) of the failure to analyse participants in the group to which they were randomized? | | | NA |
| | Risk of bias judgement | | | Low |
| Bias due to missing outcome data | 3.1 Were data for this outcome available for all, or nearly all, participants randomized? | | | PY |
| | 3.2 If N/PN/NI to 3.1: Is there evidence that result was not biased by missing outcome data? | | | NA |
| | 3.3 If N/PN to 3.2: Could missingness in the outcome depend on its true value? | | | NA |
| | 3.4 If Y/PY/NI to 3.3: Is it likely that missingness in the outcome depended on its true value? | | | NA |
| | Risk of bias judgement | | | Low |
| Bias in measurement of the outcome | 4.1 Was the method of measuring the outcome inappropriate? | | | PN |
| | 4.2 Could measurement or ascertainment of the outcome have differed between intervention groups? | | | PN |
| | 4.3 Were outcome assessors aware of the intervention received by study participants? | | | PY |
| | 4.4 If Y/PY/NI to 4.3: Could assessment of the outcome have been influenced by knowledge of intervention received? | | | PN |
| | 4.5 If Y/PY/NI to 4.4: Is it likely that assessment of the outcome was influenced by knowledge of intervention received? | | | NA |
| | Risk of bias judgement | | | Low |
| Bias in selection of the reported result | 5.1 Were the data that produced this result analysed in accordance with a pre-specified analysis plan that was finalized before unblinded outcome data were available for analysis? | | | NI |
| | 5.2 ... multiple eligible outcome measurements (e.g. scales, definitions, time points) within the outcome domain? | | | PN |
| | 5.3 ... multiple eligible analyses of the data? | | | PN |
| | Risk of bias judgement | | | Some concerns |
| Overall bias | Risk of bias judgement | | | Some concerns |
| | | | | |
| | | | | |

| Assessor | Long and Feng | Study ID | Shenjinsong(2018) | |
|---|---|---|---|---|
| **Domain** | **Signalling question** | | | **Response** |
| Bias arising from the randomization process | 1.1 Was the allocation sequence random? | | | PN |
| | 1.2 Was the allocation sequence concealed until participants were enrolled and assigned to interventions? | | | NI |
| | 1.3 Did baseline differences between intervention groups suggest a problem with the randomization process? | | | N |
| | Risk of bias judgement | | | Some concerns |
| Bias due to deviations from intended interventions | 2.1.Were participants aware of their assigned intervention during the trial? | | | PY |
| | 2.2.Were carers and people delivering the interventions aware of participants' assigned intervention during the trial? | | | PY |
| | 2.3. If Y/PY/NI to 2.1 or 2.2: Were there deviations from the intended intervention that arose because of the experimental context? | | | PN |
| | 2.4 If Y/PY to 2.3: Were these deviations likely to have affected the outcome? | | | NA |
| | 2.5. If Y/PY/NI to 2.4: Were these deviations from intended intervention balanced between groups? | | | NA |
| | 2.6 Was an appropriate analysis used to estimate the effect of assignment to intervention? | | | PY |
| | 2.7 If N/PN/NI to 2.6: Was there potential for a substantial impact (on the result) of the failure to analyse participants in the group to which they were randomized? | | | NA |
| | Risk of bias judgement | | | Low |
| Bias due to missing outcome data | 3.1 Were data for this outcome available for all, or nearly all, participants randomized? | | | NI |
| | 3.2 If N/PN/NI to 3.1: Is there evidence that result was not biased by missing outcome data? | | | PN |
| | 3.3 If N/PN to 3.2: Could missingness in the outcome depend on its true value? | | | NI |
| | 3.4 If Y/PY/NI to 3.3: Is it likely that missingness in the outcome depended on its true value? | | | PN |
| | Risk of bias judgement | | | Some concerns |

| | | | | |
|---|---|---|---|---|
| Bias in measurement of the outcome | 4.1 Was the method of measuring the outcome inappropriate? | | | PN |
| | 4.2 Could measurement or ascertainment of the outcome have differed between intervention groups? | | | N |
| | 4.3 Were outcome assessors aware of the intervention received by study participants? | | | PY |
| | 4.4 If Y/PY/NI to 4.3: Could assessment of the outcome have been influenced by knowledge of intervention received? | | | NI |
| | 4.5 If Y/PY/NI to 4.4: Is it likely that assessment of the outcome was influenced by knowledge of intervention received? | | | PN |
| | Risk of bias judgement | | | Some concerns |
| Bias in selection of the reported result | 5.1 Were the data that produced this result analysed in accordance with a pre-specified analysis plan that was finalized before unblinded outcome data were available for analysis? | | | NI |
| | 5.2 ... multiple eligible outcome measurements (e.g. scales, definitions, time points) within the outcome domain? | | | PN |
| | 5.3 ... multiple eligible analyses of the data? | | | PN |
| | Risk of bias judgement | | | Some concerns |
| Overall bias | Risk of bias judgement | | | Some concerns |
| | | | | |
| | | | | |
| Assessor | Long and Feng | Study ID | Zhongchao(2017) | |
| **Domain** | **Signalling question** | | | **Response** |
| | 1.1 Was the allocation sequence random? | | | Y |
| | 1.2 Was the allocation sequence concealed until participants were enrolled and assigned to interventions? | | | NI |

| | | |
|---|---|---|
| **Bias arising from the randomization process** | **1.3 Did baseline differences between intervention groups suggest a problem with the randomization process?** | **PN** |
| | **Risk of bias judgement** | **Some concerns** |
| **Bias due to deviations from intended interventions** | **2.1.Were participants aware of their assigned intervention during the trial?** | **NI** |
| | **2.2.Were carers and people delivering the interventions aware of participants' assigned intervention during the trial?** | **NI** |
| | **2.3. If Y/PY/NI to 2.1 or 2.2: Were there deviations from the intended intervention that arose because of the experimental context?** | **PN** |
| | **2.4 If Y/PY to 2.3: Were these deviations likely to have affected the outcome?** | **NA** |
| | **2.5. If Y/PY/NI to 2.4: Were these deviations from intended intervention balanced between groups?** | **NA** |
| | **2.6 Was an appropriate analysis used to estimate the effect of assignment to intervention?** | **Y** |
| | **2.7 If N/PN/NI to 2.6: Was there potential for a substantial impact (on the result) of the failure to analyse participants in the group to which they were randomized?** | **NA** |
| | **Risk of bias judgement** | **Low** |
| **Bias due to missing outcome data** | **3.1 Were data for this outcome available for all, or nearly all, participants randomized?** | **PY** |
| | **3.2 If N/PN/NI to 3.1: Is there evidence that result was not biased by missing outcome data?** | **NA** |
| | **3.3 If N/PN to 3.2: Could missingness in the outcome depend on its true value?** | **NA** |
| | **3.4 If Y/PY/NI to 3.3: Is it likely that missingness in the outcome depended on its true value?** | **NA** |
| | **Risk of bias judgement** | **Low** |
| **Bias in measurement of the outcome** | **4.1 Was the method of measuring the outcome inappropriate?** | **PN** |
| | **4.2 Could measurement or ascertainment of the outcome have differed between intervention groups?** | **PN** |
| | **4.3 Were outcome assessors aware of the intervention received by study participants?** | **NI** |
| | **4.4 If Y/PY/NI to 4.3: Could assessment of the outcome have been influenced by knowledge of intervention received?** | **PN** |
| | **4.5 If Y/PY/NI to 4.4: Is it likely that assessment of the outcome was influenced by knowledge of intervention received?** | **NA** |
| | **Risk of bias judgement** | **Low** |

| | | | | |
|---|---|---|---|---|
| **Bias in selection of the reported result** | **5.1 Were the data that produced this result analysed in accordance with a pre-specified analysis plan that was finalized before unblinded outcome data were available for analysis?** | | | **NI** |
| | **5.2 ... multiple eligible outcome measurements (e.g. scales, definitions, time points) within the outcome domain?** | | | **PN** |
| | **5.3 ... multiple eligible analyses of the data?** | | | **PN** |
| | **Risk of bias judgement** | | | **Some concerns** |
| **Overall bias** | **Risk of bias judgement** | | | **Some concerns** |
| | | | | |
| | | | | |
| **Assessor** | **Long and Feng** | **Study ID** | **Wangjing** | |
| **Domain** | **Signalling question** | | | **Response** |
| **Bias arising from the randomization process** | **1.1 Was the allocation sequence random?** | | | **PN** |
| | **1.2 Was the allocation sequence concealed until participants were enrolled and assigned to interventions?** | | | **PY** |
| | **1.3 Did baseline differences between intervention groups suggest a problem with the randomization process?** | | | **N** |
| | **Risk of bias judgement** | | | **Some concerns** |
| **Bias due to deviations from intended interventions** | **2.1.Were participants aware of their assigned intervention during the trial?** | | | **PY** |
| | **2.2.Were carers and people delivering the interventions aware of participants' assigned intervention during the trial?** | | | **PY** |
| | **2.3. If Y/PY/NI to 2.1 or 2.2: Were there deviations from the intended intervention that arose because of the experimental context?** | | | **PN** |
| | **2.4 If Y/PY to 2.3: Were these deviations likely to have affected the outcome?** | | | **NA** |

| | | |
|---|---|---|
| | 2.5. If Y/PY/NI to 2.4: Were these deviations from intended intervention balanced between groups? | NA |
| | 2.6 Was an appropriate analysis used to estimate the effect of assignment to intervention? | PY |
| | 2.7 If N/PN/NI to 2.6: Was there potential for a substantial impact (on the result) of the failure to analyse participants in the group to which they were randomized? | NA |
| | Risk of bias judgement | Low |
| Bias due to missing outcome data | 3.1 Were data for this outcome available for all, or nearly all, participants randomized? | NI |
| | 3.2 If N/PN/NI to 3.1: Is there evidence that result was not biased by missing outcome data? | PN |
| | 3.3 If N/PN to 3.2: Could missingness in the outcome depend on its true value? | NI |
| | 3.4 If Y/PY/NI to 3.3: Is it likely that missingness in the outcome depended on its true value? | PN |
| | Risk of bias judgement | Some concerns |
| Bias in measurement of the outcome | 4.1 Was the method of measuring the outcome inappropriate? | PN |
| | 4.2 Could measurement or ascertainment of the outcome have differed between intervention groups? | PN |
| | 4.3 Were outcome assessors aware of the intervention received by study participants? | PY |
| | 4.4 If Y/PY/NI to 4.3: Could assessment of the outcome have been influenced by knowledge of intervention received? | N |
| | 4.5 If Y/PY/NI to 4.4: Is it likely that assessment of the outcome was influenced by knowledge of intervention received? | NA |
| | Risk of bias judgement | Low |
| Bias in selection of the reported result | 5.1 Were the data that produced this result analysed in accordance with a pre-specified analysis plan that was finalized before unblinded outcome data were available for analysis? | NI |
| | 5.2 ... multiple eligible outcome measurements (e.g. scales, definitions, time points) within the outcome domain? | NI |
| | 5.3 ... multiple eligible analyses of the data? | NI |
| | Risk of bias judgement | Some concerns |

| | | | | |
|---|---|---|---|---|
| **Overall bias** | **Risk of bias judgement** | | | **Some concerns** |
| | | | | |
| PGE1+DHI | | | | |
| **Assessor** | **Long and Feng** | **Study ID** | **Fanmin(2010)** | |
| **Domain** | **Signalling question** | | | **Response** |
| **Bias arising from the randomization process** | **1.1 Was the allocation sequence random?** | | | **NI** |
| | **1.2 Was the allocation sequence concealed until participants were enrolled and assigned to interventions?** | | | **NI** |
| | **1.3 Did baseline differences between intervention groups suggest a problem with the randomization process?** | | | **N** |
| | **Risk of bias judgement** | | | **Some concerns** |
| **Bias due to deviations from intended interventions** | **2.1.Were participants aware of their assigned intervention during the trial?** | | | **PY** |
| | **2.2.Were carers and people delivering the interventions aware of participants' assigned intervention during the trial?** | | | **PY** |
| | **2.3. If Y/PY/NI to 2.1 or 2.2: Were there deviations from the intended intervention that arose because of the experimental context?** | | | **NI** |
| | **2.4 If Y/PY to 2.3: Were these deviations likely to have affected the outcome?** | | | **NA** |
| | **2.5. If Y/PY/NI to 2.4: Were these deviations from intended intervention balanced between groups?** | | | **NA** |
| | **2.6 Was an appropriate analysis used to estimate the effect of assignment to intervention?** | | | **PY** |
| | **2.7 If N/PN/NI to 2.6: Was there potential for a substantial impact (on the result) of the failure to analyse participants in the group to which they were randomized?** | | | **NA** |
| | **Risk of bias judgement** | | | **Some concerns** |
| | **3.1 Were data for this outcome available for all, or nearly all, participants randomized?** | | | **NI** |

| | | | | |
|---|---|---|---|---|
| Bias due to missing outcome data | 3.2 If N/PN/NI to 3.1: Is there evidence that result was not biased by missing outcome data? | | | PN |
| | 3.3 If N/PN to 3.2: Could missingness in the outcome depend on its true value? | | | NI |
| | 3.4 If Y/PY/NI to 3.3: Is it likely that missingness in the outcome depended on its true value? | | | PN |
| | Risk of bias judgement | | | Some concerns |
| Bias in measurement of the outcome | 4.1 Was the method of measuring the outcome inappropriate? | | | PN |
| | 4.2 Could measurement or ascertainment of the outcome have differed between intervention groups? | | | N |
| | 4.3 Were outcome assessors aware of the intervention received by study participants? | | | PY |
| | 4.4 If Y/PY/NI to 4.3: Could assessment of the outcome have been influenced by knowledge of intervention received? | | | N |
| | 4.5 If Y/PY/NI to 4.4: Is it likely that assessment of the outcome was influenced by knowledge of intervention received? | | | NA |
| | Risk of bias judgement | | | Low |
| Bias in selection of the reported result | 5.1 Were the data that produced this result analysed in accordance with a pre-specified analysis plan that was finalized before unblinded outcome data were available for analysis? | | | NI |
| | 5.2 ... multiple eligible outcome measurements (e.g. scales, definitions, time points) within the outcome domain? | | | NI |
| | 5.3 ... multiple eligible analyses of the data? | | | NI |
| | Risk of bias judgement | | | Some concerns |
| Overall bias | Risk of bias judgement | | | Some concerns |
| | | | | |
| | | | | |
| Assessor | Long and Feng | Study ID | Puhongmei(2010) | |

| Domain | Signalling question | Response |
|---|---|---|
| Bias arising from the randomization process | 1.1 Was the allocation sequence random? | NI |
| | 1.2 Was the allocation sequence concealed until participants were enrolled and assigned to interventions? | NI |
| | 1.3 Did baseline differences between intervention groups suggest a problem with the randomization process? | N |
| | Risk of bias judgement | Some concerns |
| Bias due to deviations from intended interventions | 2.1.Were participants aware of their assigned intervention during the trial? | PY |
| | 2.2.Were carers and people delivering the interventions aware of participants' assigned intervention during the trial? | PY |
| | 2.3. If Y/PY/NI to 2.1 or 2.2: Were there deviations from the intended intervention that arose because of the experimental context? | PN |
| | 2.4 If Y/PY to 2.3: Were these deviations likely to have affected the outcome? | NA |
| | 2.5. If Y/PY/NI to 2.4: Were these deviations from intended intervention balanced between groups? | NA |
| | 2.6 Was an appropriate analysis used to estimate the effect of assignment to intervention? | PY |
| | 2.7 If N/PN/NI to 2.6: Was there potential for a substantial impact (on the result) of the failure to analyse participants in the group to which they were randomized? | NA |
| | Risk of bias judgement | Low |
| Bias due to missing outcome data | 3.1 Were data for this outcome available for all, or nearly all, participants randomized? | PY |
| | 3.2 If N/PN/NI to 3.1: Is there evidence that result was not biased by missing outcome data? | NA |
| | 3.3 If N/PN to 3.2: Could missingness in the outcome depend on its true value? | NA |
| | 3.4 If Y/PY/NI to 3.3: Is it likely that missingness in the outcome depended on its true value? | NA |
| | Risk of bias judgement | Low |
| Bias in measurement of the outcome | 4.1 Was the method of measuring the outcome inappropriate? | PN |
| | 4.2 Could measurement or ascertainment of the outcome have differed between intervention groups? | PN |

| | | | | |
|---|---|---|---|---|
| | 4.3 Were outcome assessors aware of the intervention received by study participants? | | | PN |
| | 4.4 If Y/PY/NI to 4.3: Could assessment of the outcome have been influenced by knowledge of intervention received? | | | NA |
| | 4.5 If Y/PY/NI to 4.4: Is it likely that assessment of the outcome was influenced by knowledge of intervention received? | | | NA |
| | Risk of bias judgement | | | Low |
| Bias in selection of the reported result | 5.1 Were the data that produced this result analysed in accordance with a pre-specified analysis plan that was finalized before unblinded outcome data were available for analysis? | | | NI |
| | 5.2 ... multiple eligible outcome measurements (e.g. scales, definitions, time points) within the outcome domain? | | | NI |
| | 5.3 ... multiple eligible analyses of the data? | | | NI |
| | Risk of bias judgement | | | Some concerns |
| Overall bias | Risk of bias judgement | | | Some concerns |
| | | | | |
| | | | | |
| Assessor | Long and Feng | Study ID | Liqiuxia(2015) | |
| **Domain** | **Signalling question** | | | **Response** |
| Bias arising from the randomization process | 1.1 Was the allocation sequence random? | | | NI |
| | 1.2 Was the allocation sequence concealed until participants were enrolled and assigned to interventions? | | | NI |
| | 1.3 Did baseline differences between intervention groups suggest a problem with the randomization process? | | | PN |
| | Risk of bias judgement | | | Some concerns |

| | | |
|---|---|---|
| Bias due to deviations from intended interventions | 2.1.Were participants aware of their assigned intervention during the trial? | PY |
| | 2.2.Were carers and people delivering the interventions aware of participants' assigned intervention during the trial? | PY |
| | 2.3. If Y/PY/NI to 2.1 or 2.2: Were there deviations from the intended intervention that arose because of the experimental context? | N |
| | 2.4 If Y/PY to 2.3: Were these deviations likely to have affected the outcome? | NA |
| | 2.5. If Y/PY/NI to 2.4: Were these deviations from intended intervention balanced between groups? | NA |
| | 2.6 Was an appropriate analysis used to estimate the effect of assignment to intervention? | PY |
| | 2.7 If N/PN/NI to 2.6: Was there potential for a substantial impact (on the result) of the failure to analyse participants in the group to which they were randomized? | NA |
| | Risk of bias judgement | Low |
| Bias due to missing outcome data | 3.1 Were data for this outcome available for all, or nearly all, participants randomized? | PY |
| | 3.2 If N/PN/NI to 3.1: Is there evidence that result was not biased by missing outcome data? | NA |
| | 3.3 If N/PN to 3.2: Could missingness in the outcome depend on its true value? | NA |
| | 3.4 If Y/PY/NI to 3.3: Is it likely that missingness in the outcome depended on its true value? | NA |
| | Risk of bias judgement | Low |
| Bias in measurement of the outcome | 4.1 Was the method of measuring the outcome inappropriate? | N |
| | 4.2 Could measurement or ascertainment of the outcome have differed between intervention groups? | N |
| | 4.3 Were outcome assessors aware of the intervention received by study participants? | NI |
| | 4.4 If Y/PY/NI to 4.3: Could assessment of the outcome have been influenced by knowledge of intervention received? | NI |
| | 4.5 If Y/PY/NI to 4.4: Is it likely that assessment of the outcome was influenced by knowledge of intervention received? | NI |
| | Risk of bias judgement | Some concerns |
| | 5.1 Were the data that produced this result analysed in accordance with a pre-specified analysis plan that was finalized before unblinded outcome data were available for analysis? | NI |

| | | | | |
|---|---|---|---|---|
| **Bias in selection of the reported result** | **5.2 ... multiple eligible outcome measurements (e.g. scales, definitions, time points) within the outcome domain?** | | | **NI** |
| | **5.3 ... multiple eligible analyses of the data?** | | | **NI** |
| | **Risk of bias judgement** | | | **Some concerns** |
| **Overall bias** | **Risk of bias judgement** | | | **Some concerns** |
| | | | | |
| | | | | |
| **Assessor** | **Long and Feng** | **Study ID** | **Liuqingyuan(2016)** | |
| **Domain** | **Signalling question** | | | **Response** |
| **Bias arising from the randomization process** | **1.1 Was the allocation sequence random?** | | | **NI** |
| | **1.2 Was the allocation sequence concealed until participants were enrolled and assigned to interventions?** | | | **NI** |
| | **1.3 Did baseline differences between intervention groups suggest a problem with the randomization process?** | | | **PN** |
| | **Risk of bias judgement** | | | **Some concerns** |
| **Bias due to deviations from intended interventions** | **2.1.Were participants aware of their assigned intervention during the trial?** | | | **PY** |
| | **2.2.Were carers and people delivering the interventions aware of participants' assigned intervention during the trial?** | | | **PY** |
| | **2.3. If Y/PY/NI to 2.1 or 2.2: Were there deviations from the intended intervention that arose because of the experimental context?** | | | **PN** |
| | **2.4 If Y/PY to 2.3: Were these deviations likely to have affected the outcome?** | | | **NA** |
| | **2.5. If Y/PY/NI to 2.4: Were these deviations from intended intervention balanced between groups?** | | | **NA** |
| | **2.6 Was an appropriate analysis used to estimate the effect of assignment to intervention?** | | | **PY** |

| | | |
|---|---|---|
| | 2.7 If N/PN/NI to 2.6: Was there potential for a substantial impact (on the result) of the failure to analyse participants in the group to which they were randomized? | NA |
| | Risk of bias judgement | Low |
| Bias due to missing outcome data | 3.1 Were data for this outcome available for all, or nearly all, participants randomized? | Y |
| | 3.2 If N/PN/NI to 3.1: Is there evidence that result was not biased by missing outcome data? | NA |
| | 3.3 If N/PN to 3.2: Could missingness in the outcome depend on its true value? | NA |
| | 3.4 If Y/PY/NI to 3.3: Is it likely that missingness in the outcome depended on its true value? | NA |
| | Risk of bias judgement | Low |
| Bias in measurement of the outcome | 4.1 Was the method of measuring the outcome inappropriate? | PN |
| | 4.2 Could measurement or ascertainment of the outcome have differed between intervention groups? | PN |
| | 4.3 Were outcome assessors aware of the intervention received by study participants? | N |
| | 4.4 If Y/PY/NI to 4.3: Could assessment of the outcome have been influenced by knowledge of intervention received? | NA |
| | 4.5 If Y/PY/NI to 4.4: Is it likely that assessment of the outcome was influenced by knowledge of intervention received? | NA |
| | Risk of bias judgement | Low |
| Bias in selection of the reported result | 5.1 Were the data that produced this result analysed in accordance with a pre-specified analysis plan that was finalized before unblinded outcome data were available for analysis? | NI |
| | 5.2 ... multiple eligible outcome measurements (e.g. scales, definitions, time points) within the outcome domain? | NI |
| | 5.3 ... multiple eligible analyses of the data? | NI |
| | Risk of bias judgement | Some concerns |
| Overall bias | Risk of bias judgement | Some concerns |
| | | |
| | | |

| Assessor | Long and Feng | Study ID | Liuzhen(2016) | |
|---|---|---|---|---|
| **Domain** | **Signalling question** | | | **Response** |
| Bias arising from the randomization process | 1.1 Was the allocation sequence random? | | | NI |
| | 1.2 Was the allocation sequence concealed until participants were enrolled and assigned to interventions? | | | NI |
| | 1.3 Did baseline differences between intervention groups suggest a problem with the randomization process? | | | PN |
| | Risk of bias judgement | | | Some concerns |
| Bias due to deviations from intended interventions | 2.1.Were participants aware of their assigned intervention during the trial? | | | PY |
| | 2.2.Were carers and people delivering the interventions aware of participants' assigned intervention during the trial? | | | PY |
| | 2.3. If Y/PY/NI to 2.1 or 2.2: Were there deviations from the intended intervention that arose because of the experimental context? | | | PN |
| | 2.4 If Y/PY to 2.3: Were these deviations likely to have affected the outcome? | | | NA |
| | 2.5. If Y/PY/NI to 2.4: Were these deviations from intended intervention balanced between groups? | | | NA |
| | 2.6 Was an appropriate analysis used to estimate the effect of assignment to intervention? | | | NI |
| | 2.7 If N/PN/NI to 2.6: Was there potential for a substantial impact (on the result) of the failure to analyse participants in the group to which they were randomized? | | | PN |
| | Risk of bias judgement | | | Some concerns |
| Bias due to missing outcome data | 3.1 Were data for this outcome available for all, or nearly all, participants randomized? | | | PY |
| | 3.2 If N/PN/NI to 3.1: Is there evidence that result was not biased by missing outcome data? | | | NA |
| | 3.3 If N/PN to 3.2: Could missingness in the outcome depend on its true value? | | | NA |
| | 3.4 If Y/PY/NI to 3.3: Is it likely that missingness in the outcome depended on its true value? | | | NA |
| | Risk of bias judgement | | | Low |

| | | | | |
|---|---|---|---|---|
| **Bias in measurement of the outcome** | **4.1 Was the method of measuring the outcome inappropriate?** | | | **PN** |
| | **4.2 Could measurement or ascertainment of the outcome have differed between intervention groups?** | | | **PN** |
| | **4.3 Were outcome assessors aware of the intervention received by study participants?** | | | **PY** |
| | **4.4 If Y/PY/NI to 4.3: Could assessment of the outcome have been influenced by knowledge of intervention received?** | | | **PN** |
| | **4.5 If Y/PY/NI to 4.4: Is it likely that assessment of the outcome was influenced by knowledge of intervention received?** | | | **NA** |
| | **Risk of bias judgement** | | | **Low** |
| **Bias in selection of the reported result** | **5.1 Were the data that produced this result analysed in accordance with a pre-specified analysis plan that was finalized before unblinded outcome data were available for analysis?** | | | **NI** |
| | **5.2 ... multiple eligible outcome measurements (e.g. scales, definitions, time points) within the outcome domain?** | | | **NI** |
| | **5.3 ... multiple eligible analyses of the data?** | | | **NI** |
| | **Risk of bias judgement** | | | **Some concerns** |
| **Overall bias** | **Risk of bias judgement** | | | **Some concerns** |
| | | | | |
| | | | | |
| **Assessor** | **Long and Feng** | **Study ID** | **Jiayinji(2018)** | |
| **Domain** | **Signalling question** | | | **Response** |
| **Bias arising from the randomization process** | **1.1 Was the allocation sequence random?** | | | **NI** |
| | **1.2 Was the allocation sequence concealed until participants were enrolled and assigned to interventions?** | | | **NI** |
| | **1.3 Did baseline differences between intervention groups suggest a problem with the randomization process?** | | | **N** |

| | | |
|---|---|---|
| | **Risk of bias judgement** | **Some concerns** |
| **Bias due to deviations from intended interventions** | **2.1.Were participants aware of their assigned intervention during the trial?** | **PY** |
| | **2.2.Were carers and people delivering the interventions aware of participants' assigned intervention during the trial?** | **PY** |
| | **2.3. If Y/PY/NI to 2.1 or 2.2: Were there deviations from the intended intervention that arose because of the experimental context?** | **N** |
| | **2.4 If Y/PY to 2.3: Were these deviations likely to have affected the outcome?** | **NA** |
| | **2.5. If Y/PY/NI to 2.4: Were these deviations from intended intervention balanced between groups?** | **NA** |
| | **2.6 Was an appropriate analysis used to estimate the effect of assignment to intervention?** | **PY** |
| | **2.7 If N/PN/NI to 2.6: Was there potential for a substantial impact (on the result) of the failure to analyse participants in the group to which they were randomized?** | **NA** |
| | **Risk of bias judgement** | **Low** |
| **Bias due to missing outcome data** | **3.1 Were data for this outcome available for all, or nearly all, participants randomized?** | **NI** |
| | **3.2 If N/PN/NI to 3.1: Is there evidence that result was not biased by missing outcome data?** | **PN** |
| | **3.3 If N/PN to 3.2: Could missingness in the outcome depend on its true value?** | **NI** |
| | **3.4 If Y/PY/NI to 3.3: Is it likely that missingness in the outcome depended on its true value?** | **PN** |
| | **Risk of bias judgement** | **Some concerns** |
| **Bias in measurement of the outcome** | **4.1 Was the method of measuring the outcome inappropriate?** | **PN** |
| | **4.2 Could measurement or ascertainment of the outcome have differed between intervention groups?** | **PN** |
| | **4.3 Were outcome assessors aware of the intervention received by study participants?** | **PY** |
| | **4.4 If Y/PY/NI to 4.3: Could assessment of the outcome have been influenced by knowledge of intervention received?** | **NI** |
| | **4.5 If Y/PY/NI to 4.4: Is it likely that assessment of the outcome was influenced by knowledge of intervention received?** | **PN** |

| | | | | |
|---|---|---|---|---|
| | Risk of bias judgement | | | Some concerns |
| Bias in selection of the reported result | 5.1 Were the data that produced this result analysed in accordance with a pre-specified analysis plan that was finalized before unblinded outcome data were available for analysis? | | | NI |
| | 5.2 ... multiple eligible outcome measurements (e.g. scales, definitions, time points) within the outcome domain? | | | NI |
| | 5.3 ... multiple eligible analyses of the data? | | | NI |
| | Risk of bias judgement | | | Some concerns |
| Overall bias | Risk of bias judgement | | | Some concerns |
| | | | | |
| | | | | |
| Assessor | Long and Feng | Study ID | Linchenxin(2019) | |
| **Domain** | **Signalling question** | | | **Response** |
| Bias arising from the randomization process | 1.1 Was the allocation sequence random? | | | Y |
| | 1.2 Was the allocation sequence concealed until participants were enrolled and assigned to interventions? | | | NI |
| | 1.3 Did baseline differences between intervention groups suggest a problem with the randomization process? | | | N |
| | Risk of bias judgement | | | Some concerns |
| Bias due to deviations from intended interventions | 2.1.Were participants aware of their assigned intervention during the trial? | | | PY |
| | 2.2.Were carers and people delivering the interventions aware of participants' assigned intervention during the trial? | | | PN |
| | 2.3. If Y/PY/NI to 2.1 or 2.2: Were there deviations from the intended intervention that arose because of the experimental context? | | | PN |

| | | |
|---|---|---|
| | **2.4 If Y/PY to 2.3: Were these deviations likely to have affected the outcome?** | **NA** |
| | **2.5. If Y/PY/NI to 2.4: Were these deviations from intended intervention balanced between groups?** | **NA** |
| | **2.6 Was an appropriate analysis used to estimate the effect of assignment to intervention?** | **PN** |
| | **2.7 If N/PN/NI to 2.6: Was there potential for a substantial impact (on the result) of the failure to analyse participants in the group to which they were randomized?** | **PN** |
| | **Risk of bias judgement** | **Some concerns** |
| **Bias due to missing outcome data** | **3.1 Were data for this outcome available for all, or nearly all, participants randomized?** | **Y** |
| | **3.2 If N/PN/NI to 3.1: Is there evidence that result was not biased by missing outcome data?** | **NA** |
| | **3.3 If N/PN to 3.2: Could missingness in the outcome depend on its true value?** | **NA** |
| | **3.4 If Y/PY/NI to 3.3: Is it likely that missingness in the outcome depended on its true value?** | **NA** |
| | **Risk of bias judgement** | **Low** |
| **Bias in measurement of the outcome** | **4.1 Was the method of measuring the outcome inappropriate?** | **PN** |
| | **4.2 Could measurement or ascertainment of the outcome have differed between intervention groups?** | **PN** |
| | **4.3 Were outcome assessors aware of the intervention received by study participants?** | **PI** |
| | **4.4 If Y/PY/NI to 4.3: Could assessment of the outcome have been influenced by knowledge of intervention received?** | **NI** |
| | **4.5 If Y/PY/NI to 4.4: Is it likely that assessment of the outcome was influenced by knowledge of intervention received?** | **PN** |
| | **Risk of bias judgement** | **Some concerns** |
| **Bias in selection of the reported result** | **5.1 Were the data that produced this result analysed in accordance with a pre-specified analysis plan that was finalized before unblinded outcome data were available for analysis?** | **NI** |
| | **5.2 ... multiple eligible outcome measurements (e.g. scales, definitions, time points) within the outcome domain?** | **NI** |
| | **5.3 ... multiple eligible analyses of the data?** | **NI** |

| | | | | |
|---|---|---|---|---|
| | Risk of bias judgement | | | Some concerns |
| Overall bias | Risk of bias judgement | | | Some concerns |
| | | | | |
| | | | | |
| Assessor | Long and Feng | Study ID | Xiaoqianfeng(2019) | |
| **Domain** | **Signalling question** | | | **Response** |
| Bias arising from the randomization process | 1.1 Was the allocation sequence random? | | | PN |
| | 1.2 Was the allocation sequence concealed until participants were enrolled and assigned to interventions? | | | NI |
| | 1.3 Did baseline differences between intervention groups suggest a problem with the randomization process? | | | N |
| | Risk of bias judgement | | | Some concerns |
| Bias due to deviations from intended interventions | 2.1.Were participants aware of their assigned intervention during the trial? | | | PY |
| | 2.2.Were carers and people delivering the interventions aware of participants' assigned intervention during the trial? | | | PY |
| | 2.3. If Y/PY/NI to 2.1 or 2.2: Were there deviations from the intended intervention that arose because of the experimental context? | | | PN |
| | 2.4 If Y/PY to 2.3: Were these deviations likely to have affected the outcome? | | | NA |
| | 2.5. If Y/PY/NI to 2.4: Were these deviations from intended intervention balanced between groups? | | | NA |
| | 2.6 Was an appropriate analysis used to estimate the effect of assignment to intervention? | | | PY |
| | 2.7 If N/PN/NI to 2.6: Was there potential for a substantial impact (on the result) of the failure to analyse participants in the group to which they were randomized? | | | NA |
| | Risk of bias judgement | | | Low |

| | | | | |
|---|---|---|---|---|
| **Bias due to missing outcome data** | **3.1 Were data for this outcome available for all, or nearly all, participants randomized?** | | | **NI** |
| | **3.2 If N/PN/NI to 3.1: Is there evidence that result was not biased by missing outcome data?** | | | **PN** |
| | **3.3 If N/PN to 3.2: Could missingness in the outcome depend on its true value?** | | | **PY** |
| | **3.4 If Y/PY/NI to 3.3: Is it likely that missingness in the outcome depended on its true value?** | | | **PN** |
| | **Risk of bias judgement** | | | **Some concerns** |
| **Bias in measurement of the outcome** | **4.1 Was the method of measuring the outcome inappropriate?** | | | **PN** |
| | **4.2 Could measurement or ascertainment of the outcome have differed between intervention groups?** | | | **PN** |
| | **4.3 Were outcome assessors aware of the intervention received by study participants?** | | | **PY** |
| | **4.4 If Y/PY/NI to 4.3: Could assessment of the outcome have been influenced by knowledge of intervention received?** | | | **PN** |
| | **4.5 If Y/PY/NI to 4.4: Is it likely that assessment of the outcome was influenced by knowledge of intervention received?** | | | **NA** |
| | **Risk of bias judgement** | | | **Low** |
| **Bias in selection of the reported result** | **5.1 Were the data that produced this result analysed in accordance with a pre-specified analysis plan that was finalized before unblinded outcome data were available for analysis?** | | | **NI** |
| | **5.2 ... multiple eligible outcome measurements (e.g. scales, definitions, time points) within the outcome domain?** | | | **N** |
| | **5.3 ... multiple eligible analyses of the data?** | | | **N** |
| | **Risk of bias judgement** | | | **Some concerns** |
| **Overall bias** | **Risk of bias judgement** | | | **Some concerns** |
| | | | | |
| PGE1+HQI | | | | |
| **Assessor** | **Long and Feng** | **Study ID** | **Xiebinxuan(2002)** | |

| Domain | Signalling question | Response |
|---|---|---|
| Bias arising from the randomization process | 1.1 Was the allocation sequence random? | NI |
| | 1.2 Was the allocation sequence concealed until participants were enrolled and assigned to interventions? | NI |
| | 1.3 Did baseline differences between intervention groups suggest a problem with the randomization process? | N |
| | Risk of bias judgement | Some concerns |
| Bias due to deviations from intended interventions | 2.1.Were participants aware of their assigned intervention during the trial? | PY |
| | 2.2.Were carers and people delivering the interventions aware of participants' assigned intervention during the trial? | PY |
| | 2.3. If Y/PY/NI to 2.1 or 2.2: Were there deviations from the intended intervention that arose because of the experimental context? | N |
| | 2.4 If Y/PY to 2.3: Were these deviations likely to have affected the outcome? | NA |
| | 2.5. If Y/PY/NI to 2.4: Were these deviations from intended intervention balanced between groups? | NA |
| | 2.6 Was an appropriate analysis used to estimate the effect of assignment to intervention? | PY |
| | 2.7 If N/PN/NI to 2.6: Was there potential for a substantial impact (on the result) of the failure to analyse participants in the group to which they were randomized? | NA |
| | Risk of bias judgement | Low |
| Bias due to missing outcome data | 3.1 Were data for this outcome available for all, or nearly all, participants randomized? | NI |
| | 3.2 If N/PN/NI to 3.1: Is there evidence that result was not biased by missing outcome data? | PN |
| | 3.3 If N/PN to 3.2: Could missingness in the outcome depend on its true value? | PY |
| | 3.4 If Y/PY/NI to 3.3: Is it likely that missingness in the outcome depended on its true value? | PN |
| | Risk of bias judgement | Some concerns |
| | 4.1 Was the method of measuring the outcome inappropriate? | PN |

| | | |
|---|---|---|
| Bias in measurement of the outcome | 4.2 Could measurement or ascertainment of the outcome have differed between intervention groups? | N |
| | 4.3 Were outcome assessors aware of the intervention received by study participants? | PY |
| | 4.4 If Y/PY/NI to 4.3: Could assessment of the outcome have been influenced by knowledge of intervention received? | N |
| | 4.5 If Y/PY/NI to 4.4: Is it likely that assessment of the outcome was influenced by knowledge of intervention received? | NA |
| | Risk of bias judgement | Low |
| Bias in selection of the reported result | 5.1 Were the data that produced this result analysed in accordance with a pre-specified analysis plan that was finalized before unblinded outcome data were available for analysis? | NI |
| | 5.2 ... multiple eligible outcome measurements (e.g. scales, definitions, time points) within the outcome domain? | N |
| | 5.3 ... multiple eligible analyses of the data? | N |
| | Risk of bias judgement | Some concerns |
| Overall bias | Risk of bias judgement | Some concerns |

| | | | | |
|---|---|---|---|---|
| | | | | |
| Assessor | Long and Feng | Study ID | Zhaolijun(2010) | |

| Domain | Signalling question | Response |
|---|---|---|
| Bias arising from the randomization process | 1.1 Was the allocation sequence random? | NI |
| | 1.2 Was the allocation sequence concealed until participants were enrolled and assigned to interventions? | NI |
| | 1.3 Did baseline differences between intervention groups suggest a problem with the randomization process? | N |
| | Risk of bias judgement | Some concerns |

| | | |
|---|---|---|
| **Bias due to deviations from intended interventions** | **2.1.Were participants aware of their assigned intervention during the trial?** | **PY** |
| | **2.2.Were carers and people delivering the interventions aware of participants' assigned intervention during the trial?** | **PY** |
| | **2.3. If Y/PY/NI to 2.1 or 2.2: Were there deviations from the intended intervention that arose because of the experimental context?** | **PN** |
| | **2.4 If Y/PY to 2.3: Were these deviations likely to have affected the outcome?** | **NA** |
| | **2.5. If Y/PY/NI to 2.4: Were these deviations from intended intervention balanced between groups?** | **NA** |
| | **2.6 Was an appropriate analysis used to estimate the effect of assignment to intervention?** | **PY** |
| | **2.7 If N/PN/NI to 2.6: Was there potential for a substantial impact (on the result) of the failure to analyse participants in the group to which they were randomized?** | **NA** |
| | **Risk of bias judgement** | **Low** |
| **Bias due to missing outcome data** | **3.1 Were data for this outcome available for all, or nearly all, participants randomized?** | **Y** |
| | **3.2 If N/PN/NI to 3.1: Is there evidence that result was not biased by missing outcome data?** | **NA** |
| | **3.3 If N/PN to 3.2: Could missingness in the outcome depend on its true value?** | **NA** |
| | **3.4 If Y/PY/NI to 3.3: Is it likely that missingness in the outcome depended on its true value?** | **NA** |
| | **Risk of bias judgement** | **Low** |
| **Bias in measurement of the outcome** | **4.1 Was the method of measuring the outcome inappropriate?** | **PN** |
| | **4.2 Could measurement or ascertainment of the outcome have differed between intervention groups?** | **PN** |
| | **4.3 Were outcome assessors aware of the intervention received by study participants?** | **PY** |
| | **4.4 If Y/PY/NI to 4.3: Could assessment of the outcome have been influenced by knowledge of intervention received?** | **PN** |
| | **4.5 If Y/PY/NI to 4.4: Is it likely that assessment of the outcome was influenced by knowledge of intervention received?** | **NA** |
| | **Risk of bias judgement** | **Low** |
| **Bias in selection of the reported result** | **5.1 Were the data that produced this result analysed in accordance with a pre-specified analysis plan that was finalized before unblinded outcome data were available for analysis?** | **NI** |
| | **5.2 ... multiple eligible outcome measurements (e.g. scales, definitions, time points) within the outcome domain?** | **N** |

| | | | | |
|---|---|---|---|---|
| | 5.3 ... multiple eligible analyses of the data? | | | NI |
| | Risk of bias judgement | | | Some concerns |
| Overall bias | Risk of bias judgement | | | Some concerns |
| | | | | |
| | | | | |
| Assessor | Long and Feng | Study ID | Wuyanbo(2011) | |
| **Domain** | **Signalling question** | | | **Response** |
| Bias arising from the randomization process | 1.1 Was the allocation sequence random? | | | NI |
| | 1.2 Was the allocation sequence concealed until participants were enrolled and assigned to interventions? | | | NI |
| | 1.3 Did baseline differences between intervention groups suggest a problem with the randomization process? | | | PN |
| | Risk of bias judgement | | | Some concerns |
| Bias due to deviations from intended interventions | 2.1.Were participants aware of their assigned intervention during the trial? | | | PY |
| | 2.2.Were carers and people delivering the interventions aware of participants' assigned intervention during the trial? | | | PY |
| | 2.3. If Y/PY/NI to 2.1 or 2.2: Were there deviations from the intended intervention that arose because of the experimental context? | | | PN |
| | 2.4 If Y/PY to 2.3: Were these deviations likely to have affected the outcome? | | | NA |
| | 2.5. If Y/PY/NI to 2.4: Were these deviations from intended intervention balanced between groups? | | | NA |
| | 2.6 Was an appropriate analysis used to estimate the effect of assignment to intervention? | | | PY |
| | 2.7 If N/PN/NI to 2.6: Was there potential for a substantial impact (on the result) of the failure to analyse participants in the group to which they were randomized? | | | NA |

| | | | | |
|---|---|---|---|---|
| | **Risk of bias judgement** | | | Low |
| **Bias due to missing outcome data** | 3.1 Were data for this outcome available for all, or nearly all, participants randomized? | | | NI |
| | 3.2 If N/PN/NI to 3.1: Is there evidence that result was not biased by missing outcome data? | | | PN |
| | 3.3 If N/PN to 3.2: Could missingness in the outcome depend on its true value? | | | NI |
| | 3.4 If Y/PY/NI to 3.3: Is it likely that missingness in the outcome depended on its true value? | | | PN |
| | **Risk of bias judgement** | | | Some concerns |
| **Bias in measurement of the outcome** | 4.1 Was the method of measuring the outcome inappropriate? | | | PN |
| | 4.2 Could measurement or ascertainment of the outcome have differed between intervention groups? | | | PN |
| | 4.3 Were outcome assessors aware of the intervention received by study participants? | | | PY |
| | 4.4 If Y/PY/NI to 4.3: Could assessment of the outcome have been influenced by knowledge of intervention received? | | | PN |
| | 4.5 If Y/PY/NI to 4.4: Is it likely that assessment of the outcome was influenced by knowledge of intervention received? | | | NA |
| | **Risk of bias judgement** | | | Low |
| **Bias in selection of the reported result** | 5.1 Were the data that produced this result analysed in accordance with a pre-specified analysis plan that was finalized before unblinded outcome data were available for analysis? | | | NI |
| | 5.2 ... multiple eligible outcome measurements (e.g. scales, definitions, time points) within the outcome domain? | | | PN |
| | 5.3 ... multiple eligible analyses of the data? | | | PN |
| | **Risk of bias judgement** | | | Some concerns |
| **Overall bias** | **Risk of bias judgement** | | | Some concerns |
| | | | | |
| | | | | |

| Assessor | Long and Feng | Study ID | Linjixiang(2013) | |
|---|---|---|---|---|
| **Domain** | **Signalling question** | | | **Response** |
| Bias arising from the randomization process | 1.1 Was the allocation sequence random? | | | NI |
| | 1.2 Was the allocation sequence concealed until participants were enrolled and assigned to interventions? | | | NI |
| | 1.3 Did baseline differences between intervention groups suggest a problem with the randomization process? | | | N |
| | Risk of bias judgement | | | Some concerns |
| Bias due to deviations from intended interventions | 2.1.Were participants aware of their assigned intervention during the trial? | | | PY |
| | 2.2.Were carers and people delivering the interventions aware of participants' assigned intervention during the trial? | | | PY |
| | 2.3. If Y/PY/NI to 2.1 or 2.2: Were there deviations from the intended intervention that arose because of the experimental context? | | | PN |
| | 2.4 If Y/PY to 2.3: Were these deviations likely to have affected the outcome? | | | NA |
| | 2.5. If Y/PY/NI to 2.4: Were these deviations from intended intervention balanced between groups? | | | NA |
| | 2.6 Was an appropriate analysis used to estimate the effect of assignment to intervention? | | | PY |
| | 2.7 If N/PN/NI to 2.6: Was there potential for a substantial impact (on the result) of the failure to analyse participants in the group to which they were randomized? | | | NA |
| | Risk of bias judgement | | | Low |
| Bias due to missing outcome data | 3.1 Were data for this outcome available for all, or nearly all, participants randomized? | | | PY |
| | 3.2 If N/PN/NI to 3.1: Is there evidence that result was not biased by missing outcome data? | | | NA |
| | 3.3 If N/PN to 3.2: Could missingness in the outcome depend on its true value? | | | NA |
| | 3.4 If Y/PY/NI to 3.3: Is it likely that missingness in the outcome depended on its true value? | | | NA |
| | Risk of bias judgement | | | Low |
| | 4.1 Was the method of measuring the outcome inappropriate? | | | PN |

| | | | | |
|---|---|---|---|---|
| Bias in measurement of the outcome | 4.2 Could measurement or ascertainment of the outcome have differed between intervention groups? | | | PN |
| | 4.3 Were outcome assessors aware of the intervention received by study participants? | | | PY |
| | 4.4 If Y/PY/NI to 4.3: Could assessment of the outcome have been influenced by knowledge of intervention received? | | | PY |
| | 4.5 If Y/PY/NI to 4.4: Is it likely that assessment of the outcome was influenced by knowledge of intervention received? | | | NI |
| | Risk of bias judgement | | | High |
| Bias in selection of the reported result | 5.1 Were the data that produced this result analysed in accordance with a pre-specified analysis plan that was finalized before unblinded outcome data were available for analysis? | | | NI |
| | 5.2 ... multiple eligible outcome measurements (e.g. scales, definitions, time points) within the outcome domain? | | | N |
| | 5.3 ... multiple eligible analyses of the data? | | | N |
| | Risk of bias judgement | | | Some concerns |
| Overall bias | Risk of bias judgement | | | High |
| | | | | |
| | | | | |
| Assessor | Long and Feng | Study ID | Liusiyan(2013) | |
| **Domain** | **Signalling question** | | | **Response** |
| Bias arising from the randomization process | 1.1 Was the allocation sequence random? | | | NI |
| | 1.2 Was the allocation sequence concealed until participants were enrolled and assigned to interventions? | | | NI |
| | 1.3 Did baseline differences between intervention groups suggest a problem with the randomization process? | | | PN |
| | Risk of bias judgement | | | Some concerns |

| | | |
|---|---|---|
| **Bias due to deviations from intended interventions** | **2.1.Were participants aware of their assigned intervention during the trial?** | **PY** |
| | **2.2.Were carers and people delivering the interventions aware of participants' assigned intervention during the trial?** | **PY** |
| | **2.3. If Y/PY/NI to 2.1 or 2.2: Were there deviations from the intended intervention that arose because of the experimental context?** | **PN** |
| | **2.4 If Y/PY to 2.3: Were these deviations likely to have affected the outcome?** | **NA** |
| | **2.5. If Y/PY/NI to 2.4: Were these deviations from intended intervention balanced between groups?** | **NA** |
| | **2.6 Was an appropriate analysis used to estimate the effect of assignment to intervention?** | **NI** |
| | **2.7 If N/PN/NI to 2.6: Was there potential for a substantial impact (on the result) of the failure to analyse participants in the group to which they were randomized?** | **N** |
| | **Risk of bias judgement** | **Some concerns** |
| **Bias due to missing outcome data** | **3.1 Were data for this outcome available for all, or nearly all, participants randomized?** | **NI** |
| | **3.2 If N/PN/NI to 3.1: Is there evidence that result was not biased by missing outcome data?** | **N** |
| | **3.3 If N/PN to 3.2: Could missingness in the outcome depend on its true value?** | **NI** |
| | **3.4 If Y/PY/NI to 3.3: Is it likely that missingness in the outcome depended on its true value?** | **PN** |
| | **Risk of bias judgement** | **Some concerns** |
| **Bias in measurement of the outcome** | **4.1 Was the method of measuring the outcome inappropriate?** | **NI** |
| | **4.2 Could measurement or ascertainment of the outcome have differed between intervention groups?** | **PN** |
| | **4.3 Were outcome assessors aware of the intervention received by study participants?** | **PY** |
| | **4.4 If Y/PY/NI to 4.3: Could assessment of the outcome have been influenced by knowledge of intervention received?** | **NI** |
| | **4.5 If Y/PY/NI to 4.4: Is it likely that assessment of the outcome was influenced by knowledge of intervention received?** | **PN** |
| | **Risk of bias judgement** | **Some concerns** |

| | | | | |
|---|---|---|---|---|
| Bias in selection of the reported result | 5.1 Were the data that produced this result analysed in accordance with a pre-specified analysis plan that was finalized before unblinded outcome data were available for analysis? | | | NI |
| | 5.2 ... multiple eligible outcome measurements (e.g. scales, definitions, time points) within the outcome domain? | | | N |
| | 5.3 ... multiple eligible analyses of the data? | | | N |
| | Risk of bias judgement | | | Some concerns |
| Overall bias | Risk of bias judgement | | | Some concerns |
| | | | | |
| | | | | |
| Assessor | Long and Feng | Study ID | Zhoubin(2013) | |
| **Domain** | **Signalling question** | | | **Response** |
| Bias arising from the randomization process | 1.1 Was the allocation sequence random? | | | NI |
| | 1.2 Was the allocation sequence concealed until participants were enrolled and assigned to interventions? | | | NI |
| | 1.3 Did baseline differences between intervention groups suggest a problem with the randomization process? | | | N |
| | Risk of bias judgement | | | Some concerns |
| Bias due to deviations from intended interventions | 2.1.Were participants aware of their assigned intervention during the trial? | | | PY |
| | 2.2.Were carers and people delivering the interventions aware of participants' assigned intervention during the trial? | | | PY |
| | 2.3. If Y/PY/NI to 2.1 or 2.2: Were there deviations from the intended intervention that arose because of the experimental context? | | | N |
| | 2.4 If Y/PY to 2.3: Were these deviations likely to have affected the outcome? | | | NA |

| | | |
|---|---|---|
| | 2.5. If Y/PY/NI to 2.4: Were these deviations from intended intervention balanced between groups? | NA |
| | 2.6 Was an appropriate analysis used to estimate the effect of assignment to intervention? | PY |
| | 2.7 If N/PN/NI to 2.6: Was there potential for a substantial impact (on the result) of the failure to analyse participants in the group to which they were randomized? | NA |
| | Risk of bias judgement | Low |
| Bias due to missing outcome data | 3.1 Were data for this outcome available for all, or nearly all, participants randomized? | PY |
| | 3.2 If N/PN/NI to 3.1: Is there evidence that result was not biased by missing outcome data? | NA |
| | 3.3 If N/PN to 3.2: Could missingness in the outcome depend on its true value? | NA |
| | 3.4 If Y/PY/NI to 3.3: Is it likely that missingness in the outcome depended on its true value? | NA |
| | Risk of bias judgement | Low |
| Bias in measurement of the outcome | 4.1 Was the method of measuring the outcome inappropriate? | PN |
| | 4.2 Could measurement or ascertainment of the outcome have differed between intervention groups? | PN |
| | 4.3 Were outcome assessors aware of the intervention received by study participants? | PY |
| | 4.4 If Y/PY/NI to 4.3: Could assessment of the outcome have been influenced by knowledge of intervention received? | PN |
| | 4.5 If Y/PY/NI to 4.4: Is it likely that assessment of the outcome was influenced by knowledge of intervention received? | NA |
| | Risk of bias judgement | Low |
| Bias in selection of the reported result | 5.1 Were the data that produced this result analysed in accordance with a pre-specified analysis plan that was finalized before unblinded outcome data were available for analysis? | NI |
| | 5.2 ... multiple eligible outcome measurements (e.g. scales, definitions, time points) within the outcome domain? | NI |
| | 5.3 ... multiple eligible analyses of the data? | NI |
| | Risk of bias judgement | Some concerns |
| Overall bias | Risk of bias judgement | Some concerns |

| | | | | |
|---|---|---|---|---|
| | | | | |
| | | | | |
| Assessor | Long and Feng | Study ID | Fangwenjuan(2016) | |

| Domain | Signalling question | Response |
|---|---|---|
| Bias arising from the randomization process | 1.1 Was the allocation sequence random? | NI |
| | 1.2 Was the allocation sequence concealed until participants were enrolled and assigned to interventions? | NI |
| | 1.3 Did baseline differences between intervention groups suggest a problem with the randomization process? | N |
| | Risk of bias judgement | Some concerns |
| Bias due to deviations from intended interventions | 2.1.Were participants aware of their assigned intervention during the trial? | PY |
| | 2.2.Were carers and people delivering the interventions aware of participants' assigned intervention during the trial? | PY |
| | 2.3. If Y/PY/NI to 2.1 or 2.2: Were there deviations from the intended intervention that arose because of the experimental context? | PN |
| | 2.4 If Y/PY to 2.3: Were these deviations likely to have affected the outcome? | NA |
| | 2.5. If Y/PY/NI to 2.4: Were these deviations from intended intervention balanced between groups? | NA |
| | 2.6 Was an appropriate analysis used to estimate the effect of assignment to intervention? | NI |
| | 2.7 If N/PN/NI to 2.6: Was there potential for a substantial impact (on the result) of the failure to analyse participants in the group to which they were randomized? | N |
| | Risk of bias judgement | Some concerns |
| Bias due to missing outcome data | 3.1 Were data for this outcome available for all, or nearly all, participants randomized? | PY |
| | 3.2 If N/PN/NI to 3.1: Is there evidence that result was not biased by missing outcome data? | NA |
| | 3.3 If N/PN to 3.2: Could missingness in the outcome depend on its true value? | NA |

| | | | | |
|---|---|---|---|---|
| | 3.4 If Y/PY/NI to 3.3: Is it likely that missingness in the outcome depended on its true value? | | | NA |
| | **Risk of bias judgement** | | | Low |
| **Bias in measurement of the outcome** | 4.1 Was the method of measuring the outcome inappropriate? | | | NI |
| | 4.2 Could measurement or ascertainment of the outcome have differed between intervention groups? | | | PN |
| | 4.3 Were outcome assessors aware of the intervention received by study participants? | | | PY |
| | 4.4 If Y/PY/NI to 4.3: Could assessment of the outcome have been influenced by knowledge of intervention received? | | | PY |
| | 4.5 If Y/PY/NI to 4.4: Is it likely that assessment of the outcome was influenced by knowledge of intervention received? | | | NI |
| | **Risk of bias judgement** | | | Some concerns |
| **Bias in selection of the reported result** | 5.1 Were the data that produced this result analysed in accordance with a pre-specified analysis plan that was finalized before unblinded outcome data were available for analysis? | | | NI |
| | 5.2 ... multiple eligible outcome measurements (e.g. scales, definitions, time points) within the outcome domain? | | | NI |
| | 5.3 ... multiple eligible analyses of the data? | | | NI |
| | **Risk of bias judgement** | | | Some concerns |
| **Overall bias** | **Risk of bias judgement** | | | Some concerns |
| | | | | |
| | | | | |
| Assessor | Long and Feng | Study ID | Zhangliang(2016) | |
| **Domain** | **Signalling question** | | | **Response** |
| | 1.1 Was the allocation sequence random? | | | PY |

| | | |
|---|---|---|
| Bias arising from the randomization process | 1.2 Was the allocation sequence concealed until participants were enrolled and assigned to interventions? | NI |
| | 1.3 Did baseline differences between intervention groups suggest a problem with the randomization process? | N |
| | Risk of bias judgement | Some concerns |
| Bias due to deviations from intended interventions | 2.1.Were participants aware of their assigned intervention during the trial? | PY |
| | 2.2.Were carers and people delivering the interventions aware of participants' assigned intervention during the trial? | PY |
| | 2.3. If Y/PY/NI to 2.1 or 2.2: Were there deviations from the intended intervention that arose because of the experimental context? | PN |
| | 2.4 If Y/PY to 2.3: Were these deviations likely to have affected the outcome? | NA |
| | 2.5. If Y/PY/NI to 2.4: Were these deviations from intended intervention balanced between groups? | NA |
| | 2.6 Was an appropriate analysis used to estimate the effect of assignment to intervention? | NI |
| | 2.7 If N/PN/NI to 2.6: Was there potential for a substantial impact (on the result) of the failure to analyse participants in the group to which they were randomized? | NI |
| | Risk of bias judgement | Some concerns |
| Bias due to missing outcome data | 3.1 Were data for this outcome available for all, or nearly all, participants randomized? | PY |
| | 3.2 If N/PN/NI to 3.1: Is there evidence that result was not biased by missing outcome data? | NA |
| | 3.3 If N/PN to 3.2: Could missingness in the outcome depend on its true value? | NA |
| | 3.4 If Y/PY/NI to 3.3: Is it likely that missingness in the outcome depended on its true value? | NA |
| | Risk of bias judgement | Low |
| Bias in measurement of the outcome | 4.1 Was the method of measuring the outcome inappropriate? | NI |
| | 4.2 Could measurement or ascertainment of the outcome have differed between intervention groups? | PN |
| | 4.3 Were outcome assessors aware of the intervention received by study participants? | PY |
| | 4.4 If Y/PY/NI to 4.3: Could assessment of the outcome have been influenced by knowledge of intervention received? | PN |

| | | | | |
|---|---|---|---|---|
| | 4.5 If Y/PY/NI to 4.4: Is it likely that assessment of the outcome was influenced by knowledge of intervention received? | | | NA |
| | **Risk of bias judgement** | | | Low |
| **Bias in selection of the reported result** | 5.1 Were the data that produced this result analysed in accordance with a pre-specified analysis plan that was finalized before unblinded outcome data were available for analysis? | | | NI |
| | 5.2 ... multiple eligible outcome measurements (e.g. scales, definitions, time points) within the outcome domain? | | | NI |
| | 5.3 ... multiple eligible analyses of the data? | | | NI |
| | **Risk of bias judgement** | | | Some concerns |
| **Overall bias** | **Risk of bias judgement** | | | Some concerns |
| | | | | |
| | | | | |
| Assessor | Long and Feng | Study ID | Xufei(2018) | |
| **Domain** | **Signalling question** | | | **Response** |
| **Bias arising from the randomization process** | 1.1 Was the allocation sequence random? | | | NI |
| | 1.2 Was the allocation sequence concealed until participants were enrolled and assigned to interventions? | | | NI |
| | 1.3 Did baseline differences between intervention groups suggest a problem with the randomization process? | | | N |
| | **Risk of bias judgement** | | | Some concerns |
| | 2.1.Were participants aware of their assigned intervention during the trial? | | | PY |
| | 2.2.Were carers and people delivering the interventions aware of participants' assigned intervention during the trial? | | | PY |

| | | |
|---|---|---|
| **Bias due to deviations from intended interventions** | **2.3. If Y/PY/NI to 2.1 or 2.2: Were there deviations from the intended intervention that arose because of the experimental context?** | **NI** |
| | **2.4 If Y/PY to 2.3: Were these deviations likely to have affected the outcome?** | **NA** |
| | **2.5. If Y/PY/NI to 2.4: Were these deviations from intended intervention balanced between groups?** | **NA** |
| | **2.6 Was an appropriate analysis used to estimate the effect of assignment to intervention?** | **NI** |
| | **2.7 If N/PN/NI to 2.6: Was there potential for a substantial impact (on the result) of the failure to analyse participants in the group to which they were randomized?** | **PN** |
| | **Risk of bias judgement** | **Some concerns** |
| **Bias due to missing outcome data** | **3.1 Were data for this outcome available for all, or nearly all, participants randomized?** | **NI** |
| | **3.2 If N/PN/NI to 3.1: Is there evidence that result was not biased by missing outcome data?** | **PN** |
| | **3.3 If N/PN to 3.2: Could missingness in the outcome depend on its true value?** | **NI** |
| | **3.4 If Y/PY/NI to 3.3: Is it likely that missingness in the outcome depended on its true value?** | **PN** |
| | **Risk of bias judgement** | **Some concerns** |
| **Bias in measurement of the outcome** | **4.1 Was the method of measuring the outcome inappropriate?** | **N** |
| | **4.2 Could measurement or ascertainment of the outcome have differed between intervention groups?** | **N** |
| | **4.3 Were outcome assessors aware of the intervention received by study participants?** | **N** |
| | **4.4 If Y/PY/NI to 4.3: Could assessment of the outcome have been influenced by knowledge of intervention received?** | **NA** |
| | **4.5 If Y/PY/NI to 4.4: Is it likely that assessment of the outcome was influenced by knowledge of intervention received?** | **NA** |
| | **Risk of bias judgement** | **Low** |
| **Bias in selection of the reported result** | **5.1 Were the data that produced this result analysed in accordance with a pre-specified analysis plan that was finalized before unblinded outcome data were available for analysis?** | **NI** |
| | **5.2 ... multiple eligible outcome measurements (e.g. scales, definitions, time points) within the outcome domain?** | **NI** |
| | **5.3 ... multiple eligible analyses of the data?** | **NI** |

| | | | | |
|---|---|---|---|---|
| | Risk of bias judgement | | | Some concerns |
| Overall bias | Risk of bias judgement | | | Some concerns |
| | | | | |
| PGE1+SKI | | | | |
| Assessor | Long and Feng | Study ID | Wanghuibin(2009) | |
| **Domain** | **Signalling question** | | | **Response** |
| Bias arising from the randomization process | 1.1 Was the allocation sequence random? | | | NI |
| | 1.2 Was the allocation sequence concealed until participants were enrolled and assigned to interventions? | | | NI |
| | 1.3 Did baseline differences between intervention groups suggest a problem with the randomization process? | | | PN |
| | Risk of bias judgement | | | Some concerns |
| Bias due to deviations from intended interventions | 2.1.Were participants aware of their assigned intervention during the trial? | | | PY |
| | 2.2.Were carers and people delivering the interventions aware of participants' assigned intervention during the trial? | | | PY |
| | 2.3. If Y/PY/NI to 2.1 or 2.2: Were there deviations from the intended intervention that arose because of the experimental context? | | | PN |
| | 2.4 If Y/PY to 2.3: Were these deviations likely to have affected the outcome? | | | NA |
| | 2.5. If Y/PY/NI to 2.4: Were these deviations from intended intervention balanced between groups? | | | NA |
| | 2.6 Was an appropriate analysis used to estimate the effect of assignment to intervention? | | | PY |
| | 2.7 If N/PN/NI to 2.6: Was there potential for a substantial impact (on the result) of the failure to analyse participants in the group to which they were randomized? | | | NA |
| | Risk of bias judgement | | | Low |

| | | | | |
|---|---|---|---|---|
| Bias due to missing outcome data | 3.1 Were data for this outcome available for all, or nearly all, participants randomized? | | | NI |
| | 3.2 If N/PN/NI to 3.1: Is there evidence that result was not biased by missing outcome data? | | | PN |
| | 3.3 If N/PN to 3.2: Could missingness in the outcome depend on its true value? | | | NI |
| | 3.4 If Y/PY/NI to 3.3: Is it likely that missingness in the outcome depended on its true value? | | | PN |
| | Risk of bias judgement | | | Some concerns |
| Bias in measurement of the outcome | 4.1 Was the method of measuring the outcome inappropriate? | | | PN |
| | 4.2 Could measurement or ascertainment of the outcome have differed between intervention groups? | | | PN |
| | 4.3 Were outcome assessors aware of the intervention received by study participants? | | | PY |
| | 4.4 If Y/PY/NI to 4.3: Could assessment of the outcome have been influenced by knowledge of intervention received? | | | PN |
| | 4.5 If Y/PY/NI to 4.4: Is it likely that assessment of the outcome was influenced by knowledge of intervention received? | | | NA |
| | Risk of bias judgement | | | Low |
| Bias in selection of the reported result | 5.1 Were the data that produced this result analysed in accordance with a pre-specified analysis plan that was finalized before unblinded outcome data were available for analysis? | | | NI |
| | 5.2 ... multiple eligible outcome measurements (e.g. scales, definitions, time points) within the outcome domain? | | | NI |
| | 5.3 ... multiple eligible analyses of the data? | | | NI |
| | Risk of bias judgement | | | Some concerns |
| Overall bias | Risk of bias judgement | | | Some concerns |
| | | | | |
| | | | | |
| Assessor | Long and Feng | Study ID | Pangjialiang(2011) | |

| Domain | Signalling question | Response |
|---|---|---|
| Bias arising from the randomization process | 1.1 Was the allocation sequence random? | NI |
| | 1.2 Was the allocation sequence concealed until participants were enrolled and assigned to interventions? | NI |
| | 1.3 Did baseline differences between intervention groups suggest a problem with the randomization process? | N |
| | Risk of bias judgement | Some concerns |
| Bias due to deviations from intended interventions | 2.1.Were participants aware of their assigned intervention during the trial? | PY |
| | 2.2.Were carers and people delivering the interventions aware of participants' assigned intervention during the trial? | PY |
| | 2.3. If Y/PY/NI to 2.1 or 2.2: Were there deviations from the intended intervention that arose because of the experimental context? | NI |
| | 2.4 If Y/PY to 2.3: Were these deviations likely to have affected the outcome? | NA |
| | 2.5. If Y/PY/NI to 2.4: Were these deviations from intended intervention balanced between groups? | NA |
| | 2.6 Was an appropriate analysis used to estimate the effect of assignment to intervention? | NI |
| | 2.7 If N/PN/NI to 2.6: Was there potential for a substantial impact (on the result) of the failure to analyse participants in the group to which they were randomized? | PN |
| | Risk of bias judgement | Some concerns |
| Bias due to missing outcome data | 3.1 Were data for this outcome available for all, or nearly all, participants randomized? | NI |
| | 3.2 If N/PN/NI to 3.1: Is there evidence that result was not biased by missing outcome data? | N |
| | 3.3 If N/PN to 3.2: Could missingness in the outcome depend on its true value? | NI |
| | 3.4 If Y/PY/NI to 3.3: Is it likely that missingness in the outcome depended on its true value? | NI |
| | Risk of bias judgement | Some concerns |

| | | | | |
|---|---|---|---|---|
| Bias in measurement of the outcome | 4.1 Was the method of measuring the outcome inappropriate? | | | PN |
| | 4.2 Could measurement or ascertainment of the outcome have differed between intervention groups? | | | PN |
| | 4.3 Were outcome assessors aware of the intervention received by study participants? | | | PY |
| | 4.4 If Y/PY/NI to 4.3: Could assessment of the outcome have been influenced by knowledge of intervention received? | | | N |
| | 4.5 If Y/PY/NI to 4.4: Is it likely that assessment of the outcome was influenced by knowledge of intervention received? | | | NA |
| | Risk of bias judgement | | | Low |
| Bias in selection of the reported result | 5.1 Were the data that produced this result analysed in accordance with a pre-specified analysis plan that was finalized before unblinded outcome data were available for analysis? | | | NI |
| | 5.2 ... multiple eligible outcome measurements (e.g. scales, definitions, time points) within the outcome domain? | | | NI |
| | 5.3 ... multiple eligible analyses of the data? | | | NI |
| | Risk of bias judgement | | | Some concerns |
| Overall bias | Risk of bias judgement | | | Some concerns |
| | | | | |
| | | | | |
| Assessor | Long and Feng | Study ID | Zhanghong(2013) | |
| **Domain** | **Signalling question** | | | **Response** |
| Bias arising from the randomization process | 1.1 Was the allocation sequence random? | | | NI |
| | 1.2 Was the allocation sequence concealed until participants were enrolled and assigned to interventions? | | | NI |
| | 1.3 Did baseline differences between intervention groups suggest a problem with the randomization process? | | | N |

| | | |
|---|---|---|
| | **Risk of bias judgement** | **Some concerns** |
| **Bias due to deviations from intended interventions** | **2.1.Were participants aware of their assigned intervention during the trial?** | **PY** |
| | **2.2.Were carers and people delivering the interventions aware of participants' assigned intervention during the trial?** | **PY** |
| | **2.3. If Y/PY/NI to 2.1 or 2.2: Were there deviations from the intended intervention that arose because of the experimental context?** | **PN** |
| | **2.4 If Y/PY to 2.3: Were these deviations likely to have affected the outcome?** | **NA** |
| | **2.5. If Y/PY/NI to 2.4: Were these deviations from intended intervention balanced between groups?** | **NA** |
| | **2.6 Was an appropriate analysis used to estimate the effect of assignment to intervention?** | **PY** |
| | **2.7 If N/PN/NI to 2.6: Was there potential for a substantial impact (on the result) of the failure to analyse participants in the group to which they were randomized?** | **NA** |
| | **Risk of bias judgement** | **Low** |
| **Bias due to missing outcome data** | **3.1 Were data for this outcome available for all, or nearly all, participants randomized?** | **PY** |
| | **3.2 If N/PN/NI to 3.1: Is there evidence that result was not biased by missing outcome data?** | **NA** |
| | **3.3 If N/PN to 3.2: Could missingness in the outcome depend on its true value?** | **NA** |
| | **3.4 If Y/PY/NI to 3.3: Is it likely that missingness in the outcome depended on its true value?** | **NA** |
| | **Risk of bias judgement** | **Low** |
| **Bias in measurement of the outcome** | **4.1 Was the method of measuring the outcome inappropriate?** | **NI** |
| | **4.2 Could measurement or ascertainment of the outcome have differed between intervention groups?** | **PN** |
| | **4.3 Were outcome assessors aware of the intervention received by study participants?** | **PY** |
| | **4.4 If Y/PY/NI to 4.3: Could assessment of the outcome have been influenced by knowledge of intervention received?** | **PN** |
| | **4.5 If Y/PY/NI to 4.4: Is it likely that assessment of the outcome was influenced by knowledge of intervention received?** | **NA** |
| | **Risk of bias judgement** | **Low** |
| | **5.1 Were the data that produced this result analysed in accordance with a pre-specified analysis plan that was finalized before unblinded outcome data were available for analysis?** | **NI** |

| | | | | |
|---|---|---|---|---|
| **Bias in selection of the reported result** | **5.2 ... multiple eligible outcome measurements (e.g. scales, definitions, time points) within the outcome domain?** | | | **PN** |
| | **5.3 ... multiple eligible analyses of the data?** | | | **PN** |
| | **Risk of bias judgement** | | | **Some concerns** |
| **Overall bias** | **Risk of bias judgement** | | | **Some concerns** |
| | | | | |
| | | | | |
| **Assessor** | **Long and Feng** | **Study ID** | **Dingxuemei(2014)** | |

| **Domain** | **Signalling question** | **Response** |
|---|---|---|
| **Bias arising from the randomization process** | **1.1 Was the allocation sequence random?** | **NI** |
| | **1.2 Was the allocation sequence concealed until participants were enrolled and assigned to interventions?** | **NI** |
| | **1.3 Did baseline differences between intervention groups suggest a problem with the randomization process?** | **PN** |
| | **Risk of bias judgement** | **Some concerns** |
| **Bias due to deviations from intended interventions** | **2.1.Were participants aware of their assigned intervention during the trial?** | **PY** |
| | **2.2.Were carers and people delivering the interventions aware of participants' assigned intervention during the trial?** | **PY** |
| | **2.3. If Y/PY/NI to 2.1 or 2.2: Were there deviations from the intended intervention that arose because of the experimental context?** | **PN** |
| | **2.4 If Y/PY to 2.3: Were these deviations likely to have affected the outcome?** | **NA** |
| | **2.5. If Y/PY/NI to 2.4: Were these deviations from intended intervention balanced between groups?** | **NA** |
| | **2.6 Was an appropriate analysis used to estimate the effect of assignment to intervention?** | **NI** |

| | | |
|---|---|---|
| | 2.7 If N/PN/NI to 2.6: Was there potential for a substantial impact (on the result) of the failure to analyse participants in the group to which they were randomized? | PN |
| | Risk of bias judgement | Some concerns |
| Bias due to missing outcome data | 3.1 Were data for this outcome available for all, or nearly all, participants randomized? | NI |
| | 3.2 If N/PN/NI to 3.1: Is there evidence that result was not biased by missing outcome data? | PN |
| | 3.3 If N/PN to 3.2: Could missingness in the outcome depend on its true value? | NI |
| | 3.4 If Y/PY/NI to 3.3: Is it likely that missingness in the outcome depended on its true value? | PN |
| | Risk of bias judgement | Some concerns |
| Bias in measurement of the outcome | 4.1 Was the method of measuring the outcome inappropriate? | PN |
| | 4.2 Could measurement or ascertainment of the outcome have differed between intervention groups? | PN |
| | 4.3 Were outcome assessors aware of the intervention received by study participants? | PY |
| | 4.4 If Y/PY/NI to 4.3: Could assessment of the outcome have been influenced by knowledge of intervention received? | PY |
| | 4.5 If Y/PY/NI to 4.4: Is it likely that assessment of the outcome was influenced by knowledge of intervention received? | N |
| | Risk of bias judgement | Some concerns |
| Bias in selection of the reported result | 5.1 Were the data that produced this result analysed in accordance with a pre-specified analysis plan that was finalized before unblinded outcome data were available for analysis? | NI |
| | 5.2 ... multiple eligible outcome measurements (e.g. scales, definitions, time points) within the outcome domain? | NI |
| | 5.3 ... multiple eligible analyses of the data? | NI |
| | Risk of bias judgement | Some concerns |
| Overall bias | Risk of bias judgement | Some concerns |

| | | | | |
|---|---|---|---|---|
| | | | | |
| | | | | |
| **Assessor** | **Long and Feng** | **Study ID** | **Lanchunying(2014)** | |

| Domain | Signalling question | Response |
|---|---|---|
| **Bias arising from the randomization process** | **1.1 Was the allocation sequence random?** | **NI** |
| | **1.2 Was the allocation sequence concealed until participants were enrolled and assigned to interventions?** | **NI** |
| | **1.3 Did baseline differences between intervention groups suggest a problem with the randomization process?** | **N** |
| | **Risk of bias judgement** | **Some concerns** |
| **Bias due to deviations from intended interventions** | **2.1.Were participants aware of their assigned intervention during the trial?** | **PY** |
| | **2.2.Were carers and people delivering the interventions aware of participants' assigned intervention during the trial?** | **PY** |
| | **2.3. If Y/PY/NI to 2.1 or 2.2: Were there deviations from the intended intervention that arose because of the experimental context?** | **PN** |
| | **2.4 If Y/PY to 2.3: Were these deviations likely to have affected the outcome?** | **NA** |
| | **2.5. If Y/PY/NI to 2.4: Were these deviations from intended intervention balanced between groups?** | **NA** |
| | **2.6 Was an appropriate analysis used to estimate the effect of assignment to intervention?** | **PY** |
| | **2.7 If N/PN/NI to 2.6: Was there potential for a substantial impact (on the result) of the failure to analyse participants in the group to which they were randomized?** | **NA** |
| | **Risk of bias judgement** | **Low** |
| **Bias due to missing outcome data** | **3.1 Were data for this outcome available for all, or nearly all, participants randomized?** | **NI** |
| | **3.2 If N/PN/NI to 3.1: Is there evidence that result was not biased by missing outcome data?** | **PN** |
| | **3.3 If N/PN to 3.2: Could missingness in the outcome depend on its true value?** | **NI** |
| | **3.4 If Y/PY/NI to 3.3: Is it likely that missingness in the outcome depended on its true value?** | **PN** |

| | | | | |
|---|---|---|---|---|
| | Risk of bias judgement | | | Some concerns |
| Bias in measurement of the outcome | 4.1 Was the method of measuring the outcome inappropriate? | | | PN |
| | 4.2 Could measurement or ascertainment of the outcome have differed between intervention groups? | | | PN |
| | 4.3 Were outcome assessors aware of the intervention received by study participants? | | | PY |
| | 4.4 If Y/PY/NI to 4.3: Could assessment of the outcome have been influenced by knowledge of intervention received? | | | PY |
| | 4.5 If Y/PY/NI to 4.4: Is it likely that assessment of the outcome was influenced by knowledge of intervention received? | | | NI |
| | Risk of bias judgement | | | Some concerns |
| Bias in selection of the reported result | 5.1 Were the data that produced this result analysed in accordance with a pre-specified analysis plan that was finalized before unblinded outcome data were available for analysis? | | | NI |
| | 5.2 ... multiple eligible outcome measurements (e.g. scales, definitions, time points) within the outcome domain? | | | NI |
| | 5.3 ... multiple eligible analyses of the data? | | | NI |
| | Risk of bias judgement | | | Some concerns |
| Overall bias | Risk of bias judgement | | | Some concerns |
| | | | | |
| | | | | |
| Assessor | Long and Feng | Study ID | Meidongdong(2014) | |
| **Domain** | **Signalling question** | | | **Response** |
| | 1.1 Was the allocation sequence random? | | | NI |

| | | |
|---|---|---|
| **Bias arising from the randomization process** | **1.2 Was the allocation sequence concealed until participants were enrolled and assigned to interventions?** | **NI** |
| | **1.3 Did baseline differences between intervention groups suggest a problem with the randomization process?** | **N** |
| | **Risk of bias judgement** | **Some concerns** |
| **Bias due to deviations from intended interventions** | **2.1.Were participants aware of their assigned intervention during the trial?** | **PY** |
| | **2.2.Were carers and people delivering the interventions aware of participants' assigned intervention during the trial?** | **PY** |
| | **2.3. If Y/PY/NI to 2.1 or 2.2: Were there deviations from the intended intervention that arose because of the experimental context?** | **PN** |
| | **2.4 If Y/PY to 2.3: Were these deviations likely to have affected the outcome?** | **NA** |
| | **2.5. If Y/PY/NI to 2.4: Were these deviations from intended intervention balanced between groups?** | **NA** |
| | **2.6 Was an appropriate analysis used to estimate the effect of assignment to intervention?** | **PY** |
| | **2.7 If N/PN/NI to 2.6: Was there potential for a substantial impact (on the result) of the failure to analyse participants in the group to which they were randomized?** | **NA** |
| | **Risk of bias judgement** | **Low** |
| **Bias due to missing outcome data** | **3.1 Were data for this outcome available for all, or nearly all, participants randomized?** | **NI** |
| | **3.2 If N/PN/NI to 3.1: Is there evidence that result was not biased by missing outcome data?** | **PN** |
| | **3.3 If N/PN to 3.2: Could missingness in the outcome depend on its true value?** | **NI** |
| | **3.4 If Y/PY/NI to 3.3: Is it likely that missingness in the outcome depended on its true value?** | **PN** |
| | **Risk of bias judgement** | **Some concerns** |
| **Bias in measurement of the outcome** | **4.1 Was the method of measuring the outcome inappropriate?** | **PN** |
| | **4.2 Could measurement or ascertainment of the outcome have differed between intervention groups?** | **PN** |
| | **4.3 Were outcome assessors aware of the intervention received by study participants?** | **PY** |
| | **4.4 If Y/PY/NI to 4.3: Could assessment of the outcome have been influenced by knowledge of intervention received?** | **PN** |

| | | | | |
|---|---|---|---|---|
| | 4.5 If Y/PY/NI to 4.4: Is it likely that assessment of the outcome was influenced by knowledge of intervention received? | | | NA |
| | **Risk of bias judgement** | | | Low |
| **Bias in selection of the reported result** | 5.1 Were the data that produced this result analysed in accordance with a pre-specified analysis plan that was finalized before unblinded outcome data were available for analysis? | | | NI |
| | 5.2 ... multiple eligible outcome measurements (e.g. scales, definitions, time points) within the outcome domain? | | | NI |
| | 5.3 ... multiple eligible analyses of the data? | | | NI |
| | **Risk of bias judgement** | | | Some concerns |
| **Overall bias** | **Risk of bias judgement** | | | Some concerns |
| | | | | |
| | | | | |
| Assessor | Long and Feng | Study ID | Yinlili(2014) | |
| **Domain** | **Signalling question** | | | **Response** |
| **Bias arising from the randomization process** | 1.1 Was the allocation sequence random? | | | NI |
| | 1.2 Was the allocation sequence concealed until participants were enrolled and assigned to interventions? | | | NI |
| | 1.3 Did baseline differences between intervention groups suggest a problem with the randomization process? | | | N |
| | **Risk of bias judgement** | | | Some concerns |
| | 2.1.Were participants aware of their assigned intervention during the trial? | | | PY |
| | 2.2.Were carers and people delivering the interventions aware of participants' assigned intervention during the trial? | | | PY |

| | | |
|---|---|---|
| **Bias due to deviations from intended interventions** | **2.3. If Y/PY/NI to 2.1 or 2.2: Were there deviations from the intended intervention that arose because of the experimental context?** | **NI** |
| | **2.4 If Y/PY to 2.3: Were these deviations likely to have affected the outcome?** | **NA** |
| | **2.5. If Y/PY/NI to 2.4: Were these deviations from intended intervention balanced between groups?** | **NA** |
| | **2.6 Was an appropriate analysis used to estimate the effect of assignment to intervention?** | **NI** |
| | **2.7 If N/PN/NI to 2.6: Was there potential for a substantial impact (on the result) of the failure to analyse participants in the group to which they were randomized?** | **NI** |
| | **Risk of bias judgement** | **High** |
| **Bias due to missing outcome data** | **3.1 Were data for this outcome available for all, or nearly all, participants randomized?** | **PY** |
| | **3.2 If N/PN/NI to 3.1: Is there evidence that result was not biased by missing outcome data?** | **NA** |
| | **3.3 If N/PN to 3.2: Could missingness in the outcome depend on its true value?** | **NA** |
| | **3.4 If Y/PY/NI to 3.3: Is it likely that missingness in the outcome depended on its true value?** | **NA** |
| | **Risk of bias judgement** | **Low** |
| **Bias in measurement of the outcome** | **4.1 Was the method of measuring the outcome inappropriate?** | **PN** |
| | **4.2 Could measurement or ascertainment of the outcome have differed between intervention groups?** | **PN** |
| | **4.3 Were outcome assessors aware of the intervention received by study participants?** | **PY** |
| | **4.4 If Y/PY/NI to 4.3: Could assessment of the outcome have been influenced by knowledge of intervention received?** | **PY** |
| | **4.5 If Y/PY/NI to 4.4: Is it likely that assessment of the outcome was influenced by knowledge of intervention received?** | **PY** |
| | **Risk of bias judgement** | **High** |
| **Bias in selection of the reported result** | **5.1 Were the data that produced this result analysed in accordance with a pre-specified analysis plan that was finalized before unblinded outcome data were available for analysis?** | **NI** |
| | **5.2 ... multiple eligible outcome measurements (e.g. scales, definitions, time points) within the outcome domain?** | **NI** |
| | **5.3 ... multiple eligible analyses of the data?** | **NI** |
| | **Risk of bias judgement** | **Some concerns** |

| Overall bias | Risk of bias judgement | | | High |
|---|---|---|---|---|
| | | | | |
| | | | | |
| Assessor | Long and Feng | Study ID | Zhangbailin(2014) | |
| **Domain** | **Signalling question** | | | **Response** |
| Bias arising from the randomization process | 1.1 Was the allocation sequence random? | | | NI |
| | 1.2 Was the allocation sequence concealed until participants were enrolled and assigned to interventions? | | | NI |
| | 1.3 Did baseline differences between intervention groups suggest a problem with the randomization process? | | | PN |
| | Risk of bias judgement | | | Some concerns |
| Bias due to deviations from intended interventions | 2.1.Were participants aware of their assigned intervention during the trial? | | | PY |
| | 2.2.Were carers and people delivering the interventions aware of participants' assigned intervention during the trial? | | | PY |
| | 2.3. If Y/PY/NI to 2.1 or 2.2: Were there deviations from the intended intervention that arose because of the experimental context? | | | PN |
| | 2.4 If Y/PY to 2.3: Were these deviations likely to have affected the outcome? | | | NA |
| | 2.5. If Y/PY/NI to 2.4: Were these deviations from intended intervention balanced between groups? | | | NA |
| | 2.6 Was an appropriate analysis used to estimate the effect of assignment to intervention? | | | NI |
| | 2.7 If N/PN/NI to 2.6: Was there potential for a substantial impact (on the result) of the failure to analyse participants in the group to which they were randomized? | | | NI |
| | Risk of bias judgement | | | Some concerns |
| Bias due to missing outcome data | 3.1 Were data for this outcome available for all, or nearly all, participants randomized? | | | PY |
| | 3.2 If N/PN/NI to 3.1: Is there evidence that result was not biased by missing outcome data? | | | NA |

| | | | | |
|---|---|---|---|---|
| | 3.3 If N/PN to 3.2: Could missingness in the outcome depend on its true value? | | | NA |
| | 3.4 If Y/PY/NI to 3.3: Is it likely that missingness in the outcome depended on its true value? | | | NA |
| | **Risk of bias judgement** | | | Low |
| **Bias in measurement of the outcome** | 4.1 Was the method of measuring the outcome inappropriate? | | | PN |
| | 4.2 Could measurement or ascertainment of the outcome have differed between intervention groups? | | | PN |
| | 4.3 Were outcome assessors aware of the intervention received by study participants? | | | N |
| | 4.4 If Y/PY/NI to 4.3: Could assessment of the outcome have been influenced by knowledge of intervention received? | | | NA |
| | 4.5 If Y/PY/NI to 4.4: Is it likely that assessment of the outcome was influenced by knowledge of intervention received? | | | NA |
| | **Risk of bias judgement** | | | Low |
| **Bias in selection of the reported result** | 5.1 Were the data that produced this result analysed in accordance with a pre-specified analysis plan that was finalized before unblinded outcome data were available for analysis? | | | NI |
| | 5.2 ... multiple eligible outcome measurements (e.g. scales, definitions, time points) within the outcome domain? | | | NI |
| | 5.3 ... multiple eligible analyses of the data? | | | NI |
| | **Risk of bias judgement** | | | Some concerns |
| **Overall bias** | **Risk of bias judgement** | | | Some concerns |
| | | | | |
| | | | | |
| Assessor | Long and Feng | Study ID | Zhangyunpin(2017) | |
| **Domain** | **Signalling question** | | | **Response** |
| | 1.1 Was the allocation sequence random? | | | NI |

| | | |
|---|---|---|
| **Bias arising from the randomization process** | **1.2 Was the allocation sequence concealed until participants were enrolled and assigned to interventions?** | **NI** |
| | **1.3 Did baseline differences between intervention groups suggest a problem with the randomization process?** | **N** |
| | **Risk of bias judgement** | **Some concerns** |
| **Bias due to deviations from intended interventions** | **2.1.Were participants aware of their assigned intervention during the trial?** | **PY** |
| | **2.2.Were carers and people delivering the interventions aware of participants' assigned intervention during the trial?** | **PY** |
| | **2.3. If Y/PY/NI to 2.1 or 2.2: Were there deviations from the intended intervention that arose because of the experimental context?** | **NI** |
| | **2.4 If Y/PY to 2.3: Were these deviations likely to have affected the outcome?** | **NA** |
| | **2.5. If Y/PY/NI to 2.4: Were these deviations from intended intervention balanced between groups?** | **NA** |
| | **2.6 Was an appropriate analysis used to estimate the effect of assignment to intervention?** | **PN** |
| | **2.7 If N/PN/NI to 2.6: Was there potential for a substantial impact (on the result) of the failure to analyse participants in the group to which they were randomized?** | **PN** |
| | **Risk of bias judgement** | **Some concerns** |
| **Bias due to missing outcome data** | **3.1 Were data for this outcome available for all, or nearly all, participants randomized?** | **PY** |
| | **3.2 If N/PN/NI to 3.1: Is there evidence that result was not biased by missing outcome data?** | **NA** |
| | **3.3 If N/PN to 3.2: Could missingness in the outcome depend on its true value?** | **NA** |
| | **3.4 If Y/PY/NI to 3.3: Is it likely that missingness in the outcome depended on its true value?** | **NA** |
| | **Risk of bias judgement** | **Low** |
| **Bias in measurement of the outcome** | **4.1 Was the method of measuring the outcome inappropriate?** | **NI** |
| | **4.2 Could measurement or ascertainment of the outcome have differed between intervention groups?** | **PN** |
| | **4.3 Were outcome assessors aware of the intervention received by study participants?** | **PY** |
| | **4.4 If Y/PY/NI to 4.3: Could assessment of the outcome have been influenced by knowledge of intervention received?** | **PY** |

| | | | | |
|---|---|---|---|---|
| | 4.5 If Y/PY/NI to 4.4: Is it likely that assessment of the outcome was influenced by knowledge of intervention received? | | | NI |
| | Risk of bias judgement | | | High |
| Bias in selection of the reported result | 5.1 Were the data that produced this result analysed in accordance with a pre-specified analysis plan that was finalized before unblinded outcome data were available for analysis? | | | NI |
| | 5.2 ... multiple eligible outcome measurements (e.g. scales, definitions, time points) within the outcome domain? | | | NI |
| | 5.3 ... multiple eligible analyses of the data? | | | NI |
| | Risk of bias judgement | | | Some concerns |
| Overall bias | Risk of bias judgement | | | High |
| | | | | |
| | | | | |
| Assessor | Long and Feng | Study ID | Quanye(2018) | |
| **Domain** | **Signalling question** | | | **Response** |
| Bias arising from the randomization process | 1.1 Was the allocation sequence random? | | | NI |
| | 1.2 Was the allocation sequence concealed until participants were enrolled and assigned to interventions? | | | NI |
| | 1.3 Did baseline differences between intervention groups suggest a problem with the randomization process? | | | N |
| | Risk of bias judgement | | | Some concerns |
| Bias due to deviations from intended interventions | 2.1.Were participants aware of their assigned intervention during the trial? | | | PY |
| | 2.2.Were carers and people delivering the interventions aware of participants' assigned intervention during the trial? | | | PY |
| | 2.3. If Y/PY/NI to 2.1 or 2.2: Were there deviations from the intended intervention that arose because of the experimental context? | | | NI |

| | | |
|---|---|---|
| | 2.4 If Y/PY to 2.3: Were these deviations likely to have affected the outcome? | NA |
| | 2.5. If Y/PY/NI to 2.4: Were these deviations from intended intervention balanced between groups? | NA |
| | 2.6 Was an appropriate analysis used to estimate the effect of assignment to intervention? | PY |
| | 2.7 If N/PN/NI to 2.6: Was there potential for a substantial impact (on the result) of the failure to analyse participants in the group to which they were randomized? | NA |
| | **Risk of bias judgement** | Some concerns |
| **Bias due to missing outcome data** | 3.1 Were data for this outcome available for all, or nearly all, participants randomized? | NI |
| | 3.2 If N/PN/NI to 3.1: Is there evidence that result was not biased by missing outcome data? | N |
| | 3.3 If N/PN to 3.2: Could missingness in the outcome depend on its true value? | NI |
| | 3.4 If Y/PY/NI to 3.3: Is it likely that missingness in the outcome depended on its true value? | NI |
| | **Risk of bias judgement** | Some concerns |
| **Bias in measurement of the outcome** | 4.1 Was the method of measuring the outcome inappropriate? | NI |
| | 4.2 Could measurement or ascertainment of the outcome have differed between intervention groups? | PN |
| | 4.3 Were outcome assessors aware of the intervention received by study participants? | PY |
| | 4.4 If Y/PY/NI to 4.3: Could assessment of the outcome have been influenced by knowledge of intervention received? | PN |
| | 4.5 If Y/PY/NI to 4.4: Is it likely that assessment of the outcome was influenced by knowledge of intervention received? | NA |
| | **Risk of bias judgement** | Some concerns |
| **Bias in selection of the reported result** | 5.1 Were the data that produced this result analysed in accordance with a pre-specified analysis plan that was finalized before unblinded outcome data were available for analysis? | NI |
| | 5.2 ... multiple eligible outcome measurements (e.g. scales, definitions, time points) within the outcome domain? | NI |
| | 5.3 ... multiple eligible analyses of the data? | NI |

| | | | | |
|---|---|---|---|---|
| | Risk of bias judgement | | | Some concerns |
| Overall bias | Risk of bias judgement | | | Some concerns |
| | | | | |
| | | | | |
| Assessor | Long and Feng | Study ID | Zhengwenwu(2018) | |

| Domain | Signalling question | Response |
|---|---|---|
| Bias arising from the randomization process | 1.1 Was the allocation sequence random? | NI |
| | 1.2 Was the allocation sequence concealed until participants were enrolled and assigned to interventions? | NI |
| | 1.3 Did baseline differences between intervention groups suggest a problem with the randomization process? | N |
| | Risk of bias judgement | Some concerns |
| Bias due to deviations from intended interventions | 2.1.Were participants aware of their assigned intervention during the trial? | PY |
| | 2.2.Were carers and people delivering the interventions aware of participants' assigned intervention during the trial? | PY |
| | 2.3. If Y/PY/NI to 2.1 or 2.2: Were there deviations from the intended intervention that arose because of the experimental context? | PN |
| | 2.4 If Y/PY to 2.3: Were these deviations likely to have affected the outcome? | NA |
| | 2.5. If Y/PY/NI to 2.4: Were these deviations from intended intervention balanced between groups? | NA |
| | 2.6 Was an appropriate analysis used to estimate the effect of assignment to intervention? | PY |
| | 2.7 If N/PN/NI to 2.6: Was there potential for a substantial impact (on the result) of the failure to analyse participants in the group to which they were randomized? | NA |
| | Risk of bias judgement | Low |

| | | |
|---|---|---|
| Bias due to missing outcome data | 3.1 Were data for this outcome available for all, or nearly all, participants randomized? | PY |
| | 3.2 If N/PN/NI to 3.1: Is there evidence that result was not biased by missing outcome data? | NA |
| | 3.3 If N/PN to 3.2: Could missingness in the outcome depend on its true value? | NA |
| | 3.4 If Y/PY/NI to 3.3: Is it likely that missingness in the outcome depended on its true value? | NA |
| | Risk of bias judgement | Low |
| Bias in measurement of the outcome | 4.1 Was the method of measuring the outcome inappropriate? | PN |
| | 4.2 Could measurement or ascertainment of the outcome have differed between intervention groups? | PN |
| | 4.3 Were outcome assessors aware of the intervention received by study participants? | PY |
| | 4.4 If Y/PY/NI to 4.3: Could assessment of the outcome have been influenced by knowledge of intervention received? | PN |
| | 4.5 If Y/PY/NI to 4.4: Is it likely that assessment of the outcome was influenced by knowledge of intervention received? | NA |
| | Risk of bias judgement | Low |
| Bias in selection of the reported result | 5.1 Were the data that produced this result analysed in accordance with a pre-specified analysis plan that was finalized before unblinded outcome data were available for analysis? | NI |
| | 5.2 ... multiple eligible outcome measurements (e.g. scales, definitions, time points) within the outcome domain? | NI |
| | 5.3 ... multiple eligible analyses of the data? | NI |
| | Risk of bias judgement | Some concerns |
| Overall bias | Risk of bias judgement | Some concerns |

| | | | | |
|---|---|---|---|---|
| | | | | |
| | | | | |
| Assessor | Long and Feng | Study ID | Wangxiaojun(2019) | |

| Domain | Signalling question | Response |
|---|---|---|
| Bias arising from the randomization process | 1.1 Was the allocation sequence random? | NI |
| | 1.2 Was the allocation sequence concealed until participants were enrolled and assigned to interventions? | NI |
| | 1.3 Did baseline differences between intervention groups suggest a problem with the randomization process? | N |
| | Risk of bias judgement | Some concerns |
| Bias due to deviations from intended interventions | 2.1.Were participants aware of their assigned intervention during the trial? | PY |
| | 2.2.Were carers and people delivering the interventions aware of participants' assigned intervention during the trial? | PY |
| | 2.3. If Y/PY/NI to 2.1 or 2.2: Were there deviations from the intended intervention that arose because of the experimental context? | PN |
| | 2.4 If Y/PY to 2.3: Were these deviations likely to have affected the outcome? | NA |
| | 2.5. If Y/PY/NI to 2.4: Were these deviations from intended intervention balanced between groups? | NA |
| | 2.6 Was an appropriate analysis used to estimate the effect of assignment to intervention? | PY |
| | 2.7 If N/PN/NI to 2.6: Was there potential for a substantial impact (on the result) of the failure to analyse participants in the group to which they were randomized? | NA |
| | Risk of bias judgement | Low |
| Bias due to missing outcome data | 3.1 Were data for this outcome available for all, or nearly all, participants randomized? | NI |
| | 3.2 If N/PN/NI to 3.1: Is there evidence that result was not biased by missing outcome data? | PN |
| | 3.3 If N/PN to 3.2: Could missingness in the outcome depend on its true value? | NI |
| | 3.4 If Y/PY/NI to 3.3: Is it likely that missingness in the outcome depended on its true value? | PN |
| | Risk of bias judgement | Some concerns |
| | 4.1 Was the method of measuring the outcome inappropriate? | PN |

| | | | | |
|---|---|---|---|---|
| **Bias in measurement of the outcome** | **4.2 Could measurement or ascertainment of the outcome have differed between intervention groups?** | | | **PN** |
| | **4.3 Were outcome assessors aware of the intervention received by study participants?** | | | **PY** |
| | **4.4 If Y/PY/NI to 4.3: Could assessment of the outcome have been influenced by knowledge of intervention received?** | | | **PY** |
| | **4.5 If Y/PY/NI to 4.4: Is it likely that assessment of the outcome was influenced by knowledge of intervention received?** | | | **NI** |
| | **Risk of bias judgement** | | | **Some concerns** |
| **Bias in selection of the reported result** | **5.1 Were the data that produced this result analysed in accordance with a pre-specified analysis plan that was finalized before unblinded outcome data were available for analysis?** | | | **NI** |
| | **5.2 ... multiple eligible outcome measurements (e.g. scales, definitions, time points) within the outcome domain?** | | | **NI** |
| | **5.3 ... multiple eligible analyses of the data?** | | | **NI** |
| | **Risk of bias judgement** | | | **Some concerns** |
| **Overall bias** | **Risk of bias judgement** | | | **Some concerns** |
| | | | | |
| | | | | |
| **Assessor** | **Long and Feng** | **Study ID** | **Zhangmailang(2019)** | |
| **Domain** | **Signalling question** | | | **Response** |
| **Bias arising from the randomization process** | **1.1 Was the allocation sequence random?** | | | **NI** |
| | **1.2 Was the allocation sequence concealed until participants were enrolled and assigned to interventions?** | | | **NI** |
| | **1.3 Did baseline differences between intervention groups suggest a problem with the randomization process?** | | | **PN** |

| | | |
|---|---|---|
| | **Risk of bias judgement** | **Some concerns** |
| **Bias due to deviations from intended interventions** | **2.1.Were participants aware of their assigned intervention during the trial?** | **PY** |
| | **2.2.Were carers and people delivering the interventions aware of participants' assigned intervention during the trial?** | **PY** |
| | **2.3. If Y/PY/NI to 2.1 or 2.2: Were there deviations from the intended intervention that arose because of the experimental context?** | **PN** |
| | **2.4 If Y/PY to 2.3: Were these deviations likely to have affected the outcome?** | **NA** |
| | **2.5. If Y/PY/NI to 2.4: Were these deviations from intended intervention balanced between groups?** | **NA** |
| | **2.6 Was an appropriate analysis used to estimate the effect of assignment to intervention?** | **PY** |
| | **2.7 If N/PN/NI to 2.6: Was there potential for a substantial impact (on the result) of the failure to analyse participants in the group to which they were randomized?** | **NA** |
| | **Risk of bias judgement** | **Low** |
| **Bias due to missing outcome data** | **3.1 Were data for this outcome available for all, or nearly all, participants randomized?** | **PY** |
| | **3.2 If N/PN/NI to 3.1: Is there evidence that result was not biased by missing outcome data?** | **NA** |
| | **3.3 If N/PN to 3.2: Could missingness in the outcome depend on its true value?** | **NA** |
| | **3.4 If Y/PY/NI to 3.3: Is it likely that missingness in the outcome depended on its true value?** | **NA** |
| | **Risk of bias judgement** | **Low** |
| **Bias in measurement of the outcome** | **4.1 Was the method of measuring the outcome inappropriate?** | **NI** |
| | **4.2 Could measurement or ascertainment of the outcome have differed between intervention groups?** | **PN** |
| | **4.3 Were outcome assessors aware of the intervention received by study participants?** | **PY** |
| | **4.4 If Y/PY/NI to 4.3: Could assessment of the outcome have been influenced by knowledge of intervention received?** | **PY** |
| | **4.5 If Y/PY/NI to 4.4: Is it likely that assessment of the outcome was influenced by knowledge of intervention received?** | **PY** |
| | **Risk of bias judgement** | **Some concerns** |

| | | | | |
|---|---|---|---|---|
| Bias in selection of the reported result | 5.1 Were the data that produced this result analysed in accordance with a pre-specified analysis plan that was finalized before unblinded outcome data were available for analysis? | | | NI |
| | 5.2 ... multiple eligible outcome measurements (e.g. scales, definitions, time points) within the outcome domain? | | | NI |
| | 5.3 ... multiple eligible analyses of the data? | | | NI |
| | Risk of bias judgement | | | Some concerns |
| Overall bias | Risk of bias judgement | | | Some concerns |
| | | | | |
| | | | | |
| Assessor | Long and Feng | Study ID | Wenxusheng(2021) | |
| **Domain** | **Signalling question** | | | **Response** |
| Bias arising from the randomization process | 1.1 Was the allocation sequence random? | | | PN |
| | 1.2 Was the allocation sequence concealed until participants were enrolled and assigned to interventions? | | | PN |
| | 1.3 Did baseline differences between intervention groups suggest a problem with the randomization process? | | | N |
| | Risk of bias judgement | | | High |
| Bias due to deviations from intended interventions | 2.1.Were participants aware of their assigned intervention during the trial? | | | PY |
| | 2.2.Were carers and people delivering the interventions aware of participants' assigned intervention during the trial? | | | PY |
| | 2.3. If Y/PY/NI to 2.1 or 2.2: Were there deviations from the intended intervention that arose because of the experimental context? | | | N |
| | 2.4 If Y/PY to 2.3: Were these deviations likely to have affected the outcome? | | | NA |
| | 2.5. If Y/PY/NI to 2.4: Were these deviations from intended intervention balanced between groups? | | | NA |

| | | |
|---|---|---|
| | 2.6 Was an appropriate analysis used to estimate the effect of assignment to intervention? | PY |
| | 2.7 If N/PN/NI to 2.6: Was there potential for a substantial impact (on the result) of the failure to analyse participants in the group to which they were randomized? | NA |
| | Risk of bias judgement | Low |
| Bias due to missing outcome data | 3.1 Were data for this outcome available for all, or nearly all, participants randomized? | Y |
| | 3.2 If N/PN/NI to 3.1: Is there evidence that result was not biased by missing outcome data? | NA |
| | 3.3 If N/PN to 3.2: Could missingness in the outcome depend on its true value? | NA |
| | 3.4 If Y/PY/NI to 3.3: Is it likely that missingness in the outcome depended on its true value? | NA |
| | Risk of bias judgement | Low |
| Bias in measurement of the outcome | 4.1 Was the method of measuring the outcome inappropriate? | NI |
| | 4.2 Could measurement or ascertainment of the outcome have differed between intervention groups? | PN |
| | 4.3 Were outcome assessors aware of the intervention received by study participants? | PY |
| | 4.4 If Y/PY/NI to 4.3: Could assessment of the outcome have been influenced by knowledge of intervention received? | PY |
| | 4.5 If Y/PY/NI to 4.4: Is it likely that assessment of the outcome was influenced by knowledge of intervention received? | NI |
| | Risk of bias judgement | Some concerns |
| Bias in selection of the reported result | 5.1 Were the data that produced this result analysed in accordance with a pre-specified analysis plan that was finalized before unblinded outcome data were available for analysis? | NI |
| | 5.2 ... multiple eligible outcome measurements (e.g. scales, definitions, time points) within the outcome domain? | NI |
| | 5.3 ... multiple eligible analyses of the data? | NI |
| | Risk of bias judgement | Some concerns |
| Overall bias | Risk of bias judgement | High |
| | | |

| | | | | |
|---|---|---|---|---|
| Assessor | Long and Feng | Study ID | Niexing(2022) | |
| **Domain** | **Signalling question** | | | **Response** |
| Bias arising from the randomization process | 1.1 Was the allocation sequence random? | | | NI |
| | 1.2 Was the allocation sequence concealed until participants were enrolled and assigned to interventions? | | | NI |
| | 1.3 Did baseline differences between intervention groups suggest a problem with the randomization process? | | | PN |
| | Risk of bias judgement | | | Some concerns |
| Bias due to deviations from intended interventions | 2.1.Were participants aware of their assigned intervention during the trial? | | | PY |
| | 2.2.Were carers and people delivering the interventions aware of participants' assigned intervention during the trial? | | | PY |
| | 2.3. If Y/PY/NI to 2.1 or 2.2: Were there deviations from the intended intervention that arose because of the experimental context? | | | PN |
| | 2.4 If Y/PY to 2.3: Were these deviations likely to have affected the outcome? | | | NA |
| | 2.5. If Y/PY/NI to 2.4: Were these deviations from intended intervention balanced between groups? | | | NA |
| | 2.6 Was an appropriate analysis used to estimate the effect of assignment to intervention? | | | PY |
| | 2.7 If N/PN/NI to 2.6: Was there potential for a substantial impact (on the result) of the failure to analyse participants in the group to which they were randomized? | | | NA |
| | Risk of bias judgement | | | Low |
| Bias due to missing outcome data | 3.1 Were data for this outcome available for all, or nearly all, participants randomized? | | | NI |
| | 3.2 If N/PN/NI to 3.1: Is there evidence that result was not biased by missing outcome data? | | | PY |
| | 3.3 If N/PN to 3.2: Could missingness in the outcome depend on its true value? | | | NA |
| | 3.4 If Y/PY/NI to 3.3: Is it likely that missingness in the outcome depended on its true value? | | | NA |
| | Risk of bias judgement | | | Low |

| | | | | |
|---|---|---|---|---|
| **Bias in measurement of the outcome** | **4.1 Was the method of measuring the outcome inappropriate?** | | | **PN** |
| | **4.2 Could measurement or ascertainment of the outcome have differed between intervention groups?** | | | **PN** |
| | **4.3 Were outcome assessors aware of the intervention received by study participants?** | | | **PY** |
| | **4.4 If Y/PY/NI to 4.3: Could assessment of the outcome have been influenced by knowledge of intervention received?** | | | **PN** |
| | **4.5 If Y/PY/NI to 4.4: Is it likely that assessment of the outcome was influenced by knowledge of intervention received?** | | | **NA** |
| | **Risk of bias judgement** | | | **Low** |
| **Bias in selection of the reported result** | **5.1 Were the data that produced this result analysed in accordance with a pre-specified analysis plan that was finalized before unblinded outcome data were available for analysis?** | | | **NI** |
| | **5.2 ... multiple eligible outcome measurements (e.g. scales, definitions, time points) within the outcome domain?** | | | **NI** |
| | **5.3 ... multiple eligible analyses of the data?** | | | **NI** |
| | **Risk of bias judgement** | | | **Some concerns** |
| **Overall bias** | **Risk of bias judgement** | | | **Some concerns** |
| | | | | |
| PGE1+SXTI | | | | |
| **Assessor** | **Long and Feng** | **Study ID** | **Rujianyong(2008)** | |
| **Domain** | **Signalling question** | | | **Response** |
| **Bias arising from the randomization process** | **1.1 Was the allocation sequence random?** | | | **NI** |
| | **1.2 Was the allocation sequence concealed until participants were enrolled and assigned to interventions?** | | | **NI** |
| | **1.3 Did baseline differences between intervention groups suggest a problem with the randomization process?** | | | **PN** |

| | | |
|---|---|---|
| | **Risk of bias judgement** | **Some concerns** |
| **Bias due to deviations from intended interventions** | **2.1.Were participants aware of their assigned intervention during the trial?** | **PY** |
| | **2.2.Were carers and people delivering the interventions aware of participants' assigned intervention during the trial?** | **PY** |
| | **2.3. If Y/PY/NI to 2.1 or 2.2: Were there deviations from the intended intervention that arose because of the experimental context?** | **PN** |
| | **2.4 If Y/PY to 2.3: Were these deviations likely to have affected the outcome?** | **NA** |
| | **2.5. If Y/PY/NI to 2.4: Were these deviations from intended intervention balanced between groups?** | **NA** |
| | **2.6 Was an appropriate analysis used to estimate the effect of assignment to intervention?** | **PY** |
| | **2.7 If N/PN/NI to 2.6: Was there potential for a substantial impact (on the result) of the failure to analyse participants in the group to which they were randomized?** | **NA** |
| | **Risk of bias judgement** | **Low** |
| **Bias due to missing outcome data** | **3.1 Were data for this outcome available for all, or nearly all, participants randomized?** | **NI** |
| | **3.2 If N/PN/NI to 3.1: Is there evidence that result was not biased by missing outcome data?** | **PN** |
| | **3.3 If N/PN to 3.2: Could missingness in the outcome depend on its true value?** | **NI** |
| | **3.4 If Y/PY/NI to 3.3: Is it likely that missingness in the outcome depended on its true value?** | **PN** |
| | **Risk of bias judgement** | **Some concerns** |
| **Bias in measurement of the outcome** | **4.1 Was the method of measuring the outcome inappropriate?** | **PN** |
| | **4.2 Could measurement or ascertainment of the outcome have differed between intervention groups?** | **PN** |
| | **4.3 Were outcome assessors aware of the intervention received by study participants?** | **PY** |
| | **4.4 If Y/PY/NI to 4.3: Could assessment of the outcome have been influenced by knowledge of intervention received?** | **PN** |
| | **4.5 If Y/PY/NI to 4.4: Is it likely that assessment of the outcome was influenced by knowledge of intervention received?** | **NA** |
| | **Risk of bias judgement** | **Low** |

| | | | | |
|---|---|---|---|---|
| Bias in selection of the reported result | 5.1 Were the data that produced this result analysed in accordance with a pre-specified analysis plan that was finalized before unblinded outcome data were available for analysis? | | | NI |
| | 5.2 ... multiple eligible outcome measurements (e.g. scales, definitions, time points) within the outcome domain? | | | NI |
| | 5.3 ... multiple eligible analyses of the data? | | | NI |
| | Risk of bias judgement | | | Some concerns |
| Overall bias | Risk of bias judgement | | | Some concerns |
| | | | | |
| | | | | |
| Assessor | Long and Feng | Study ID | Gongyun(2009) | |
| **Domain** | **Signalling question** | | | **Response** |
| Bias arising from the randomization process | 1.1 Was the allocation sequence random? | | | NI |
| | 1.2 Was the allocation sequence concealed until participants were enrolled and assigned to interventions? | | | NI |
| | 1.3 Did baseline differences between intervention groups suggest a problem with the randomization process? | | | N |
| | Risk of bias judgement | | | Some concerns |
| Bias due to deviations from intended interventions | 2.1.Were participants aware of their assigned intervention during the trial? | | | PY |
| | 2.2.Were carers and people delivering the interventions aware of participants' assigned intervention during the trial? | | | PY |
| | 2.3. If Y/PY/NI to 2.1 or 2.2: Were there deviations from the intended intervention that arose because of the experimental context? | | | PN |
| | 2.4 If Y/PY to 2.3: Were these deviations likely to have affected the outcome? | | | NA |

| | | |
|---|---|---|
| | 2.5. If Y/PY/NI to 2.4: Were these deviations from intended intervention balanced between groups? | NA |
| | 2.6 Was an appropriate analysis used to estimate the effect of assignment to intervention? | NI |
| | 2.7 If N/PN/NI to 2.6: Was there potential for a substantial impact (on the result) of the failure to analyse participants in the group to which they were randomized? | NI |
| | **Risk of bias judgement** | Some concerns |
| **Bias due to missing outcome data** | 3.1 Were data for this outcome available for all, or nearly all, participants randomized? | PY |
| | 3.2 If N/PN/NI to 3.1: Is there evidence that result was not biased by missing outcome data? | NA |
| | 3.3 If N/PN to 3.2: Could missingness in the outcome depend on its true value? | NA |
| | 3.4 If Y/PY/NI to 3.3: Is it likely that missingness in the outcome depended on its true value? | NA |
| | **Risk of bias judgement** | Low |
| **Bias in measurement of the outcome** | 4.1 Was the method of measuring the outcome inappropriate? | PN |
| | 4.2 Could measurement or ascertainment of the outcome have differed between intervention groups? | PN |
| | 4.3 Were outcome assessors aware of the intervention received by study participants? | PY |
| | 4.4 If Y/PY/NI to 4.3: Could assessment of the outcome have been influenced by knowledge of intervention received? | PN |
| | 4.5 If Y/PY/NI to 4.4: Is it likely that assessment of the outcome was influenced by knowledge of intervention received? | NA |
| | **Risk of bias judgement** | Low |
| **Bias in selection of the reported result** | 5.1 Were the data that produced this result analysed in accordance with a pre-specified analysis plan that was finalized before unblinded outcome data were available for analysis? | NI |
| | 5.2 ... multiple eligible outcome measurements (e.g. scales, definitions, time points) within the outcome domain? | NI |
| | 5.3 ... multiple eligible analyses of the data? | NI |
| | **Risk of bias judgement** | Some concerns |

| | | | | |
|---|---|---|---|---|
| **Overall bias** | **Risk of bias judgement** | | | **Some concerns** |
| | | | | |
| | | | | |
| **Assessor** | **Long and Feng** | **Study ID** | **Xingyelan(2012)** | |
| **Domain** | **Signalling question** | | | **Response** |
| **Bias arising from the randomization process** | **1.1 Was the allocation sequence random?** | | | **NI** |
| | **1.2 Was the allocation sequence concealed until participants were enrolled and assigned to interventions?** | | | **NI** |
| | **1.3 Did baseline differences between intervention groups suggest a problem with the randomization process?** | | | **PN** |
| | **Risk of bias judgement** | | | **Some concerns** |
| **Bias due to deviations from intended interventions** | **2.1.Were participants aware of their assigned intervention during the trial?** | | | **PY** |
| | **2.2.Were carers and people delivering the interventions aware of participants' assigned intervention during the trial?** | | | **PY** |
| | **2.3. If Y/PY/NI to 2.1 or 2.2: Were there deviations from the intended intervention that arose because of the experimental context?** | | | **PN** |
| | **2.4 If Y/PY to 2.3: Were these deviations likely to have affected the outcome?** | | | **NA** |
| | **2.5. If Y/PY/NI to 2.4: Were these deviations from intended intervention balanced between groups?** | | | **NA** |
| | **2.6 Was an appropriate analysis used to estimate the effect of assignment to intervention?** | | | **PY** |
| | **2.7 If N/PN/NI to 2.6: Was there potential for a substantial impact (on the result) of the failure to analyse participants in the group to which they were randomized?** | | | **NA** |
| | **Risk of bias judgement** | | | **Low** |
| **Bias due to missing outcome data** | **3.1 Were data for this outcome available for all, or nearly all, participants randomized?** | | | **NI** |
| | **3.2 If N/PN/NI to 3.1: Is there evidence that result was not biased by missing outcome data?** | | | **N** |

| | | | | |
|---|---|---|---|---|
| | 3.3 If N/PN to 3.2: Could missingness in the outcome depend on its true value? | | | NI |
| | 3.4 If Y/PY/NI to 3.3: Is it likely that missingness in the outcome depended on its true value? | | | NI |
| | **Risk of bias judgement** | | | Some concerns |
| **Bias in measurement of the outcome** | 4.1 Was the method of measuring the outcome inappropriate? | | | PN |
| | 4.2 Could measurement or ascertainment of the outcome have differed between intervention groups? | | | PN |
| | 4.3 Were outcome assessors aware of the intervention received by study participants? | | | PY |
| | 4.4 If Y/PY/NI to 4.3: Could assessment of the outcome have been influenced by knowledge of intervention received? | | | PN |
| | 4.5 If Y/PY/NI to 4.4: Is it likely that assessment of the outcome was influenced by knowledge of intervention received? | | | NA |
| | **Risk of bias judgement** | | | Low |
| **Bias in selection of the reported result** | 5.1 Were the data that produced this result analysed in accordance with a pre-specified analysis plan that was finalized before unblinded outcome data were available for analysis? | | | NI |
| | 5.2 ... multiple eligible outcome measurements (e.g. scales, definitions, time points) within the outcome domain? | | | NI |
| | 5.3 ... multiple eligible analyses of the data? | | | NI |
| | **Risk of bias judgement** | | | Some concerns |
| **Overall bias** | **Risk of bias judgement** | | | Some concerns |
| | | | | |
| | | | | |
| Assessor | Long and Feng | Study ID | Tiantian(2018) | |
| **Domain** | **Signalling question** | | | **Response** |

| | | |
|---|---|---|
| **Bias arising from the randomization process** | **1.1 Was the allocation sequence random?** | **Y** |
| | **1.2 Was the allocation sequence concealed until participants were enrolled and assigned to interventions?** | **NI** |
| | **1.3 Did baseline differences between intervention groups suggest a problem with the randomization process?** | **N** |
| | **Risk of bias judgement** | **Some concerns** |
| **Bias due to deviations from intended interventions** | **2.1.Were participants aware of their assigned intervention during the trial?** | **PY** |
| | **2.2.Were carers and people delivering the interventions aware of participants' assigned intervention during the trial?** | **PY** |
| | **2.3. If Y/PY/NI to 2.1 or 2.2: Were there deviations from the intended intervention that arose because of the experimental context?** | **PN** |
| | **2.4 If Y/PY to 2.3: Were these deviations likely to have affected the outcome?** | **NA** |
| | **2.5. If Y/PY/NI to 2.4: Were these deviations from intended intervention balanced between groups?** | **NA** |
| | **2.6 Was an appropriate analysis used to estimate the effect of assignment to intervention?** | **PY** |
| | **2.7 If N/PN/NI to 2.6: Was there potential for a substantial impact (on the result) of the failure to analyse participants in the group to which they were randomized?** | **NA** |
| | **Risk of bias judgement** | **Low** |
| **Bias due to missing outcome data** | **3.1 Were data for this outcome available for all, or nearly all, participants randomized?** | **NI** |
| | **3.2 If N/PN/NI to 3.1: Is there evidence that result was not biased by missing outcome data?** | **PY** |
| | **3.3 If N/PN to 3.2: Could missingness in the outcome depend on its true value?** | **NA** |
| | **3.4 If Y/PY/NI to 3.3: Is it likely that missingness in the outcome depended on its true value?** | **NA** |
| | **Risk of bias judgement** | **Low** |
| **Bias in measurement of the outcome** | **4.1 Was the method of measuring the outcome inappropriate?** | **PN** |
| | **4.2 Could measurement or ascertainment of the outcome have differed between intervention groups?** | **PN** |
| | **4.3 Were outcome assessors aware of the intervention received by study participants?** | **PY** |
| | **4.4 If Y/PY/NI to 4.3: Could assessment of the outcome have been influenced by knowledge of intervention received?** | **PY** |

| | | | | |
|---|---|---|---|---|
| | 4.5 If Y/PY/NI to 4.4: Is it likely that assessment of the outcome was influenced by knowledge of intervention received? | | | NI |
| | Risk of bias judgement | | | Some concerns |
| Bias in selection of the reported result | 5.1 Were the data that produced this result analysed in accordance with a pre-specified analysis plan that was finalized before unblinded outcome data were available for analysis? | | | NI |
| | 5.2 ... multiple eligible outcome measurements (e.g. scales, definitions, time points) within the outcome domain? | | | NI |
| | 5.3 ... multiple eligible analyses of the data? | | | NI |
| | Risk of bias judgement | | | Some concerns |
| Overall bias | Risk of bias judgement | | | Some concerns |
| | | | | |
| PGE1+XBJI | | | | |
| Assessor | Long and Feng | Study ID | Caoli(2016) | |
| **Domain** | **Signalling question** | | | **Response** |
| Bias arising from the randomization process | 1.1 Was the allocation sequence random? | | | Y |
| | 1.2 Was the allocation sequence concealed until participants were enrolled and assigned to interventions? | | | NI |
| | 1.3 Did baseline differences between intervention groups suggest a problem with the randomization process? | | | N |
| | Risk of bias judgement | | | Some concerns |
| | 2.1.Were participants aware of their assigned intervention during the trial? | | | PY |

| | | |
|---|---|---|
| Bias due to deviations from intended interventions | 2.2.Were carers and people delivering the interventions aware of participants' assigned intervention during the trial? | PY |
| | 2.3. If Y/PY/NI to 2.1 or 2.2: Were there deviations from the intended intervention that arose because of the experimental context? | PN |
| | 2.4 If Y/PY to 2.3: Were these deviations likely to have affected the outcome? | NA |
| | 2.5. If Y/PY/NI to 2.4: Were these deviations from intended intervention balanced between groups? | NA |
| | 2.6 Was an appropriate analysis used to estimate the effect of assignment to intervention? | PY |
| | 2.7 If N/PN/NI to 2.6: Was there potential for a substantial impact (on the result) of the failure to analyse participants in the group to which they were randomized? | NA |
| | Risk of bias judgement | Low |
| Bias due to missing outcome data | 3.1 Were data for this outcome available for all, or nearly all, participants randomized? | PY |
| | 3.2 If N/PN/NI to 3.1: Is there evidence that result was not biased by missing outcome data? | NA |
| | 3.3 If N/PN to 3.2: Could missingness in the outcome depend on its true value? | NA |
| | 3.4 If Y/PY/NI to 3.3: Is it likely that missingness in the outcome depended on its true value? | NA |
| | Risk of bias judgement | Low |
| Bias in measurement of the outcome | 4.1 Was the method of measuring the outcome inappropriate? | PN |
| | 4.2 Could measurement or ascertainment of the outcome have differed between intervention groups? | PN |
| | 4.3 Were outcome assessors aware of the intervention received by study participants? | PY |
| | 4.4 If Y/PY/NI to 4.3: Could assessment of the outcome have been influenced by knowledge of intervention received? | PN |
| | 4.5 If Y/PY/NI to 4.4: Is it likely that assessment of the outcome was influenced by knowledge of intervention received? | NA |
| | Risk of bias judgement | Low |
| Bias in selection of the reported result | 5.1 Were the data that produced this result analysed in accordance with a pre-specified analysis plan that was finalized before unblinded outcome data were available for analysis? | NI |
| | 5.2 ... multiple eligible outcome measurements (e.g. scales, definitions, time points) within the outcome domain? | NI |
| | 5.3 ... multiple eligible analyses of the data? | NI |

| | | | | |
|---|---|---|---|---|
| | Risk of bias judgement | | | Some concerns |
| Overall bias | Risk of bias judgement | | | Some concerns |
| | | | | |
| | | | | |
| Assessor | Long and Feng | Study ID | Liqing(2016) | |

| Domain | Signalling question | Response |
|---|---|---|
| Bias arising from the randomization process | 1.1 Was the allocation sequence random? | Y |
| | 1.2 Was the allocation sequence concealed until participants were enrolled and assigned to interventions? | NI |
| | 1.3 Did baseline differences between intervention groups suggest a problem with the randomization process? | N |
| | Risk of bias judgement | Some concerns |
| Bias due to deviations from intended interventions | 2.1.Were participants aware of their assigned intervention during the trial? | PY |
| | 2.2.Were carers and people delivering the interventions aware of participants' assigned intervention during the trial? | PY |
| | 2.3. If Y/PY/NI to 2.1 or 2.2: Were there deviations from the intended intervention that arose because of the experimental context? | PN |
| | 2.4 If Y/PY to 2.3: Were these deviations likely to have affected the outcome? | NA |
| | 2.5. If Y/PY/NI to 2.4: Were these deviations from intended intervention balanced between groups? | NA |
| | 2.6 Was an appropriate analysis used to estimate the effect of assignment to intervention? | PY |
| | 2.7 If N/PN/NI to 2.6: Was there potential for a substantial impact (on the result) of the failure to analyse participants in the group to which they were randomized? | NA |
| | Risk of bias judgement | Low |

| | | | | |
|---|---|---|---|---|
| **Bias due to missing outcome data** | **3.1 Were data for this outcome available for all, or nearly all, participants randomized?** | | | **PY** |
| | **3.2 If N/PN/NI to 3.1: Is there evidence that result was not biased by missing outcome data?** | | | **NA** |
| | **3.3 If N/PN to 3.2: Could missingness in the outcome depend on its true value?** | | | **NA** |
| | **3.4 If Y/PY/NI to 3.3: Is it likely that missingness in the outcome depended on its true value?** | | | **NA** |
| | **Risk of bias judgement** | | | **Low** |
| **Bias in measurement of the outcome** | **4.1 Was the method of measuring the outcome inappropriate?** | | | **PN** |
| | **4.2 Could measurement or ascertainment of the outcome have differed between intervention groups?** | | | **PN** |
| | **4.3 Were outcome assessors aware of the intervention received by study participants?** | | | **PY** |
| | **4.4 If Y/PY/NI to 4.3: Could assessment of the outcome have been influenced by knowledge of intervention received?** | | | **PN** |
| | **4.5 If Y/PY/NI to 4.4: Is it likely that assessment of the outcome was influenced by knowledge of intervention received?** | | | **NA** |
| | **Risk of bias judgement** | | | **Low** |
| **Bias in selection of the reported result** | **5.1 Were the data that produced this result analysed in accordance with a pre-specified analysis plan that was finalized before unblinded outcome data were available for analysis?** | | | **NI** |
| | **5.2 ... multiple eligible outcome measurements (e.g. scales, definitions, time points) within the outcome domain?** | | | **NI** |
| | **5.3 ... multiple eligible analyses of the data?** | | | **NI** |
| | **Risk of bias judgement** | | | **Some concerns** |
| **Overall bias** | **Risk of bias judgement** | | | **Some concerns** |
| | | | | |
| | | | | |
| **Assessor** | **Long and Feng** | **Study ID** | **Jiangqiang(2017)** | |

| Domain | Signalling question | Response |
|---|---|---|
| Bias arising from the randomization process | 1.1 Was the allocation sequence random? | Y |
| | 1.2 Was the allocation sequence concealed until participants were enrolled and assigned to interventions? | NI |
| | 1.3 Did baseline differences between intervention groups suggest a problem with the randomization process? | N |
| | Risk of bias judgement | Some concerns |
| Bias due to deviations from intended interventions | 2.1.Were participants aware of their assigned intervention during the trial? | PY |
| | 2.2.Were carers and people delivering the interventions aware of participants' assigned intervention during the trial? | PY |
| | 2.3. If Y/PY/NI to 2.1 or 2.2: Were there deviations from the intended intervention that arose because of the experimental context? | PN |
| | 2.4 If Y/PY to 2.3: Were these deviations likely to have affected the outcome? | NA |
| | 2.5. If Y/PY/NI to 2.4: Were these deviations from intended intervention balanced between groups? | NA |
| | 2.6 Was an appropriate analysis used to estimate the effect of assignment to intervention? | PY |
| | 2.7 If N/PN/NI to 2.6: Was there potential for a substantial impact (on the result) of the failure to analyse participants in the group to which they were randomized? | NA |
| | Risk of bias judgement | Low |
| Bias due to missing outcome data | 3.1 Were data for this outcome available for all, or nearly all, participants randomized? | PY |
| | 3.2 If N/PN/NI to 3.1: Is there evidence that result was not biased by missing outcome data? | NA |
| | 3.3 If N/PN to 3.2: Could missingness in the outcome depend on its true value? | NA |
| | 3.4 If Y/PY/NI to 3.3: Is it likely that missingness in the outcome depended on its true value? | NA |
| | Risk of bias judgement | Low |
| Bias in measurement of the outcome | 4.1 Was the method of measuring the outcome inappropriate? | PN |
| | 4.2 Could measurement or ascertainment of the outcome have differed between intervention groups? | PN |

| | | |
|---|---|---|
| | **4.3 Were outcome assessors aware of the intervention received by study participants?** | **PY** |
| | **4.4 If Y/PY/NI to 4.3: Could assessment of the outcome have been influenced by knowledge of intervention received?** | **PN** |
| | **4.5 If Y/PY/NI to 4.4: Is it likely that assessment of the outcome was influenced by knowledge of intervention received?** | **NA** |
| | **Risk of bias judgement** | **Low** |
| **Bias in selection of the reported result** | **5.1 Were the data that produced this result analysed in accordance with a pre-specified analysis plan that was finalized before unblinded outcome data were available for analysis?** | **NI** |
| | **5.2 ... multiple eligible outcome measurements (e.g. scales, definitions, time points) within the outcome domain?** | **NI** |
| | **5.3 ... multiple eligible analyses of the data?** | **NI** |
| | **Risk of bias judgement** | **Some concerns** |
| **Overall bias** | **Risk of bias judgement** | **Some concerns** |

## *Supplementary figure S2-S7: Heterogeneity of outcome measures*

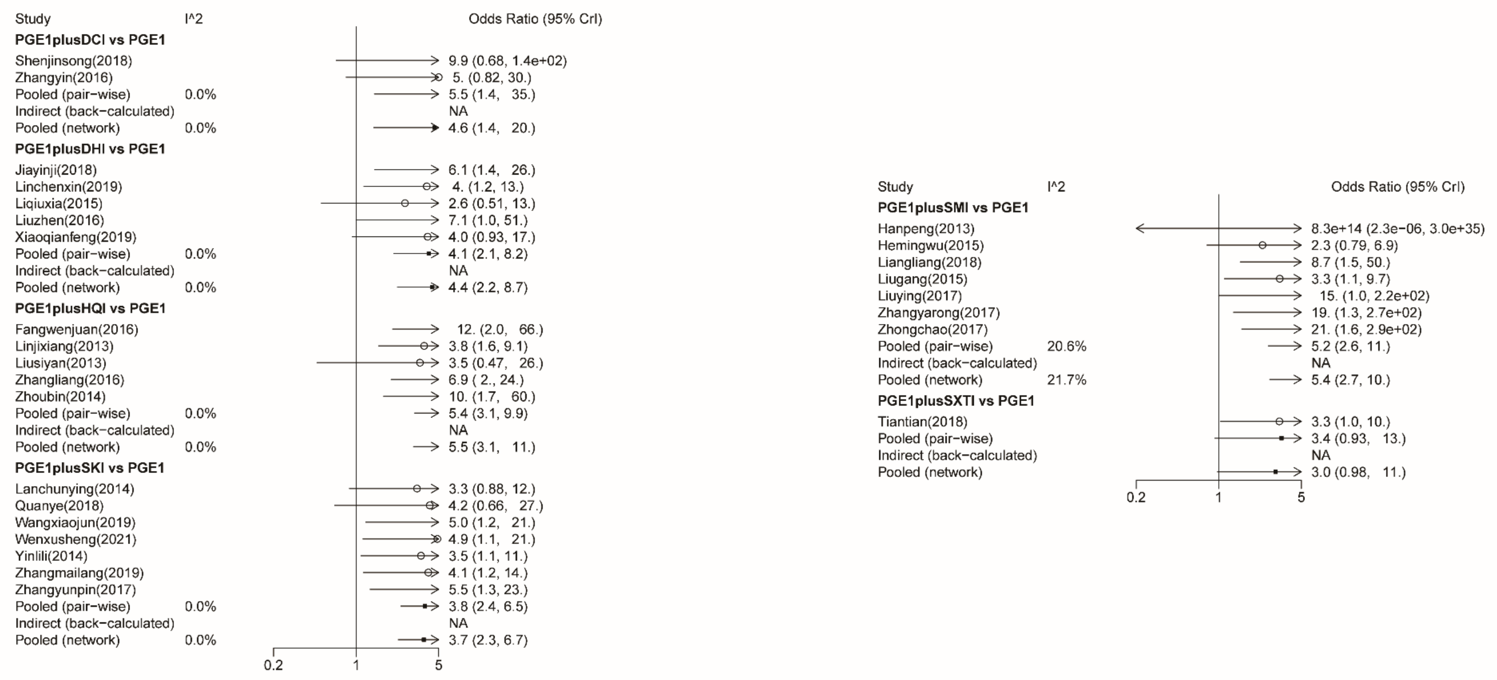


**Figure S2: Total effective rate**

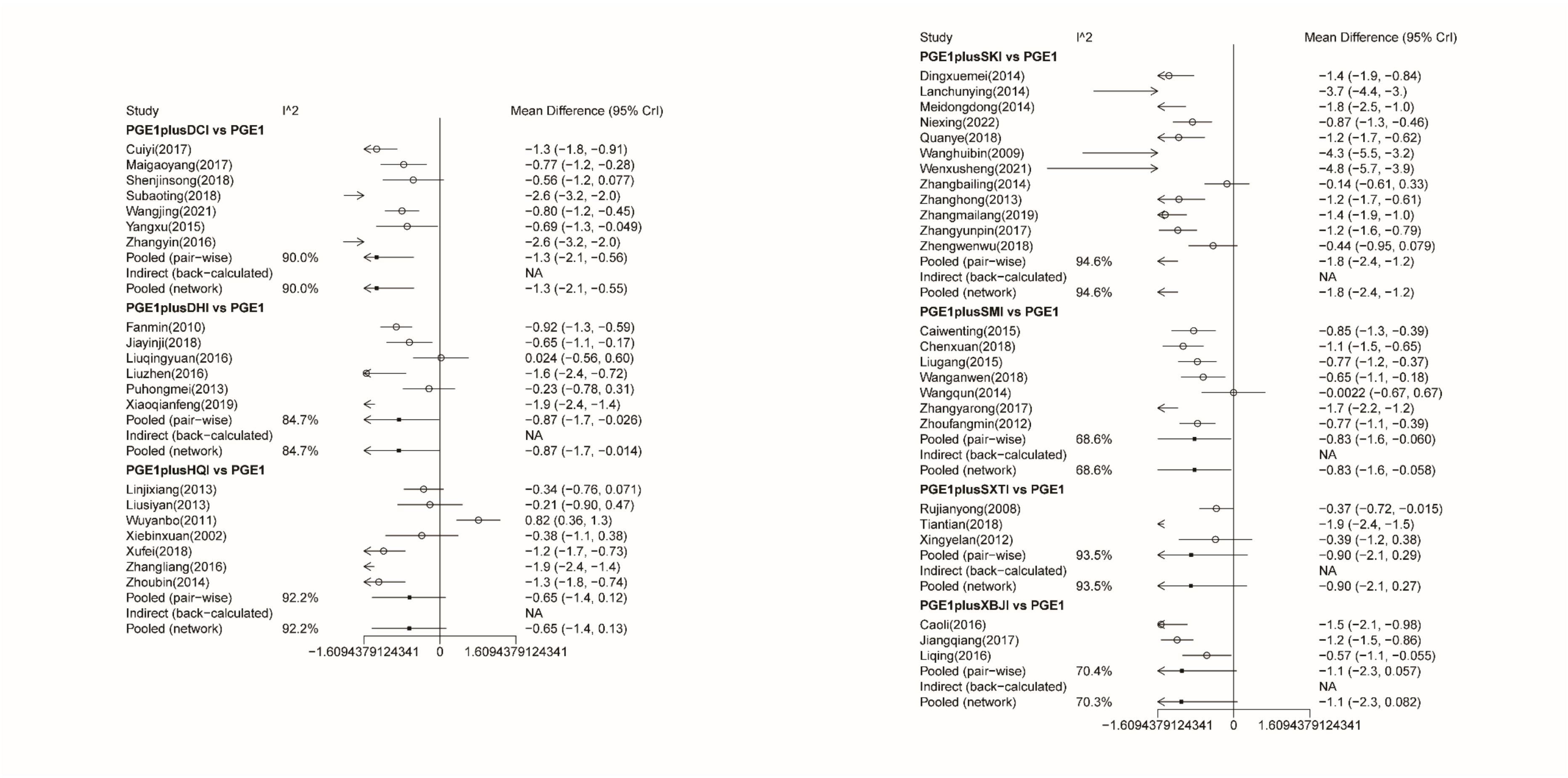


**Figure S3: Serum Creatinine**

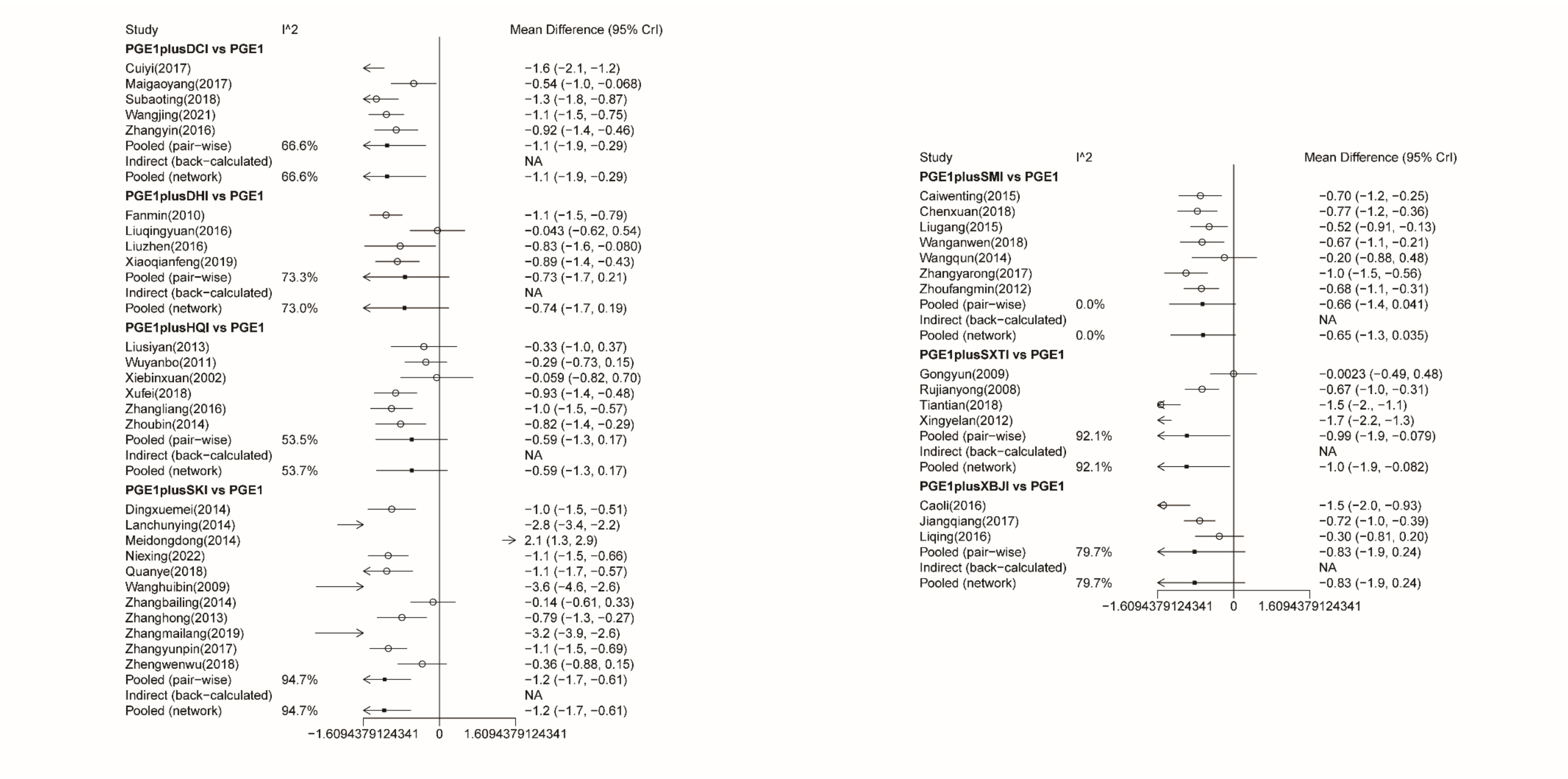


**Figure S4: Blood Urea Nitrogen**

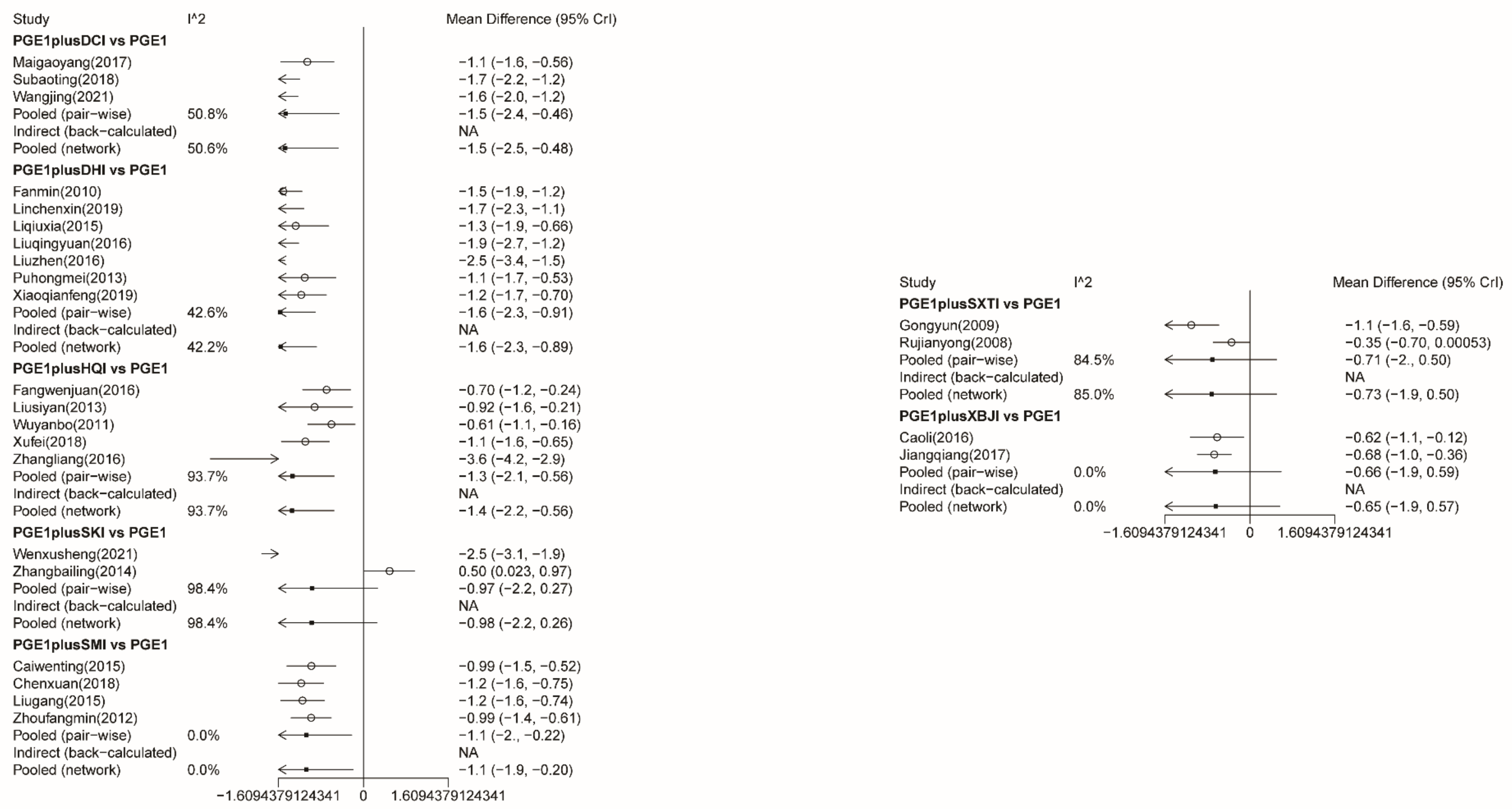


**Figure S5: Urinary Albumin excretion rates**

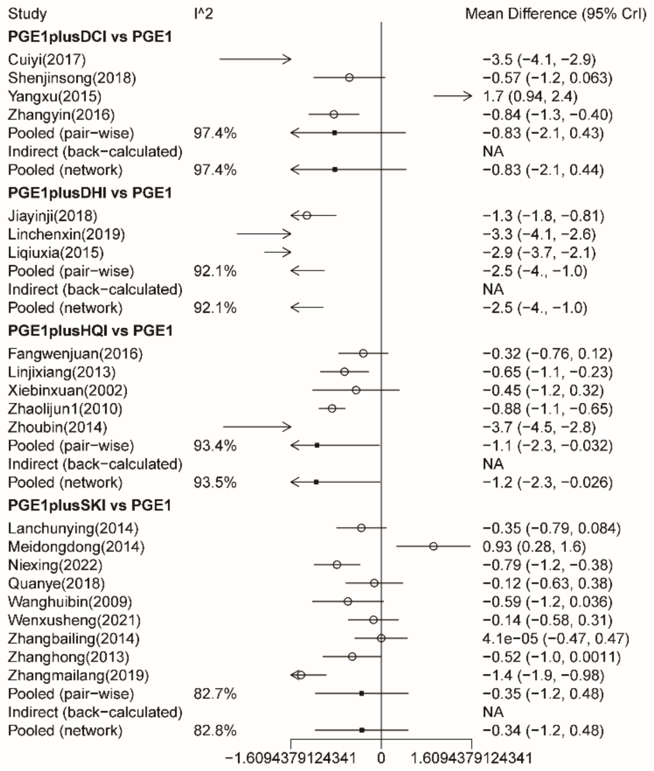


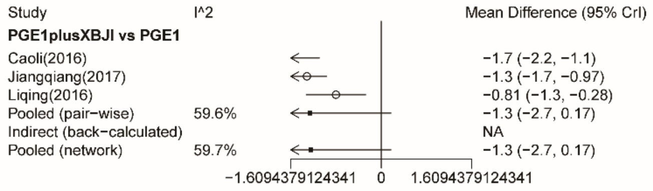


**Figure S6: 24h Urine Albumin**

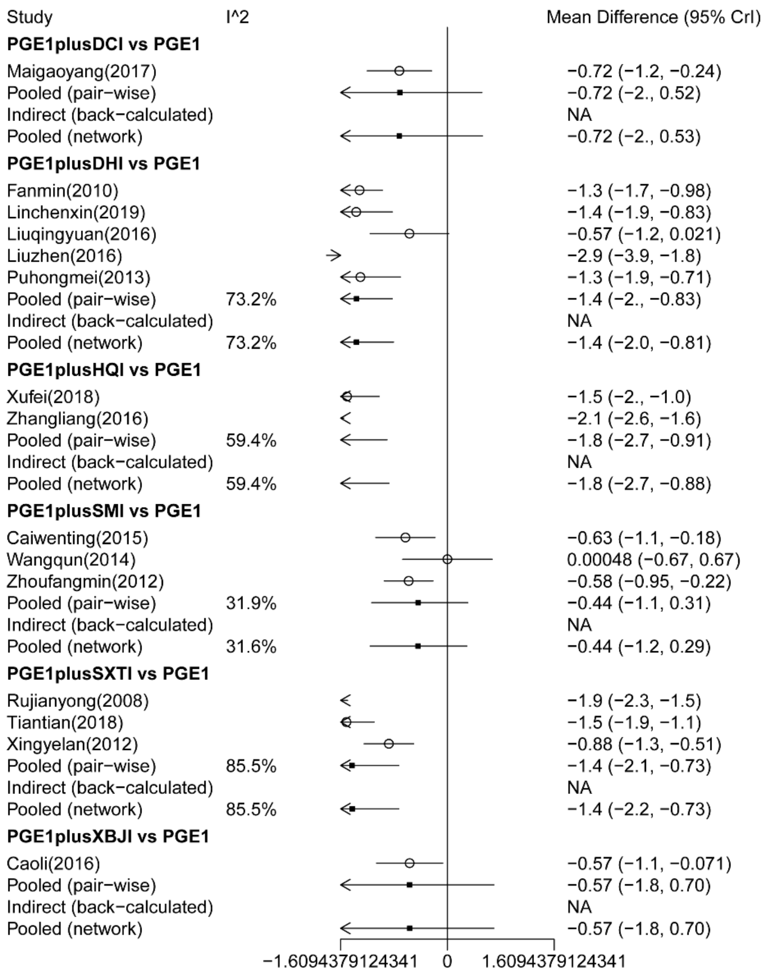


**Figure S7: Urinary beta 2-microglobulin**

# *Supplementary figure S8-S20: The Trial Sequential Analysis of the primary outcomes*

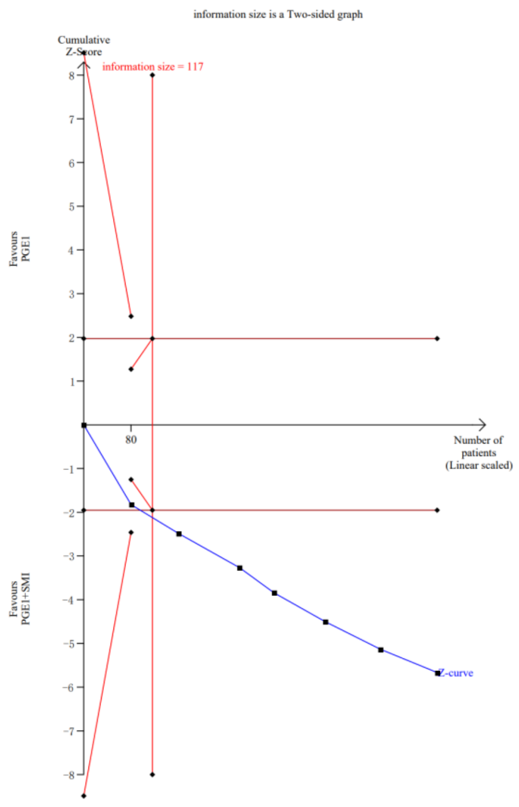


**Figure S8: Trial Sequential Analysis of total effective rate on PGE1+SMI**

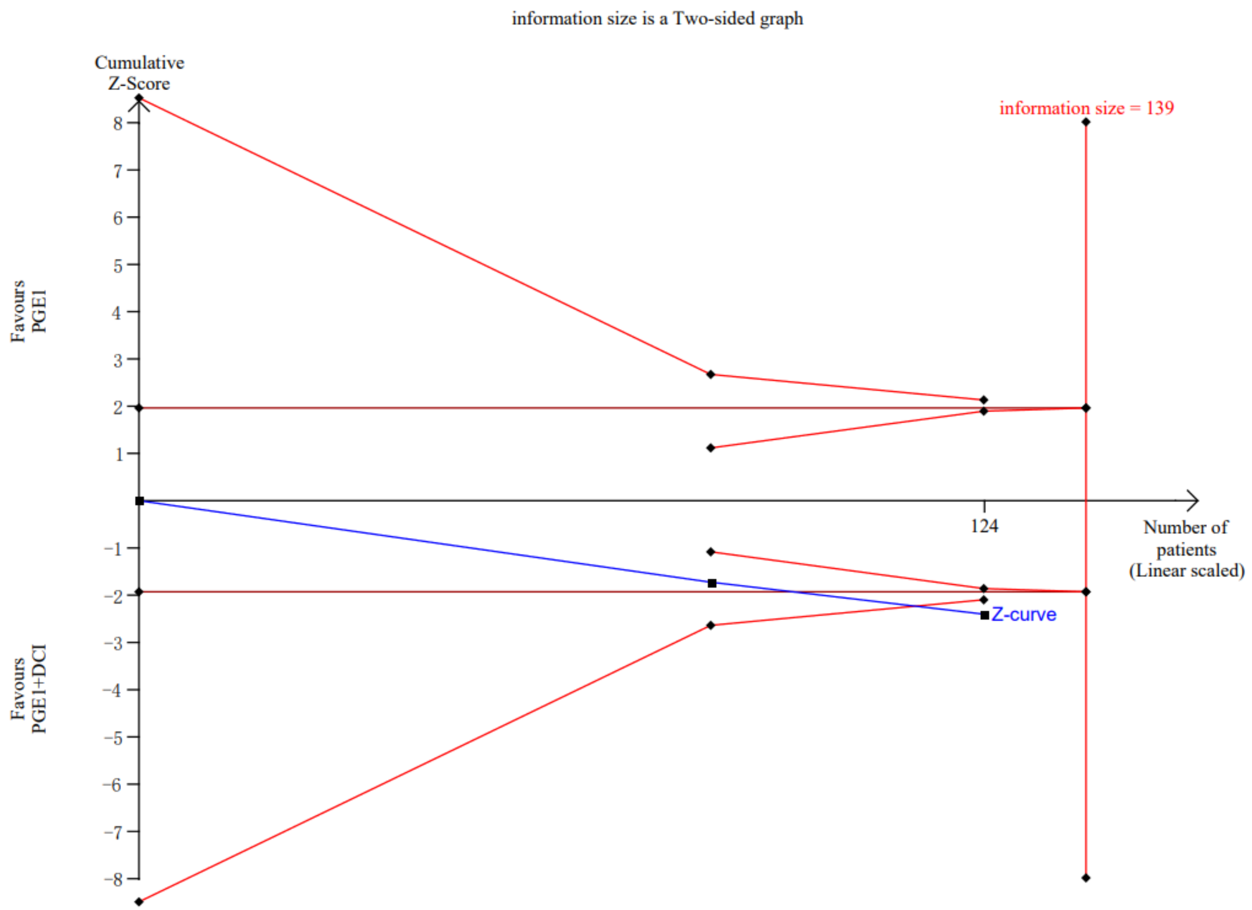


**Figure S9: Trial Sequential Analysis of total effective rate on PGE1+DCI**

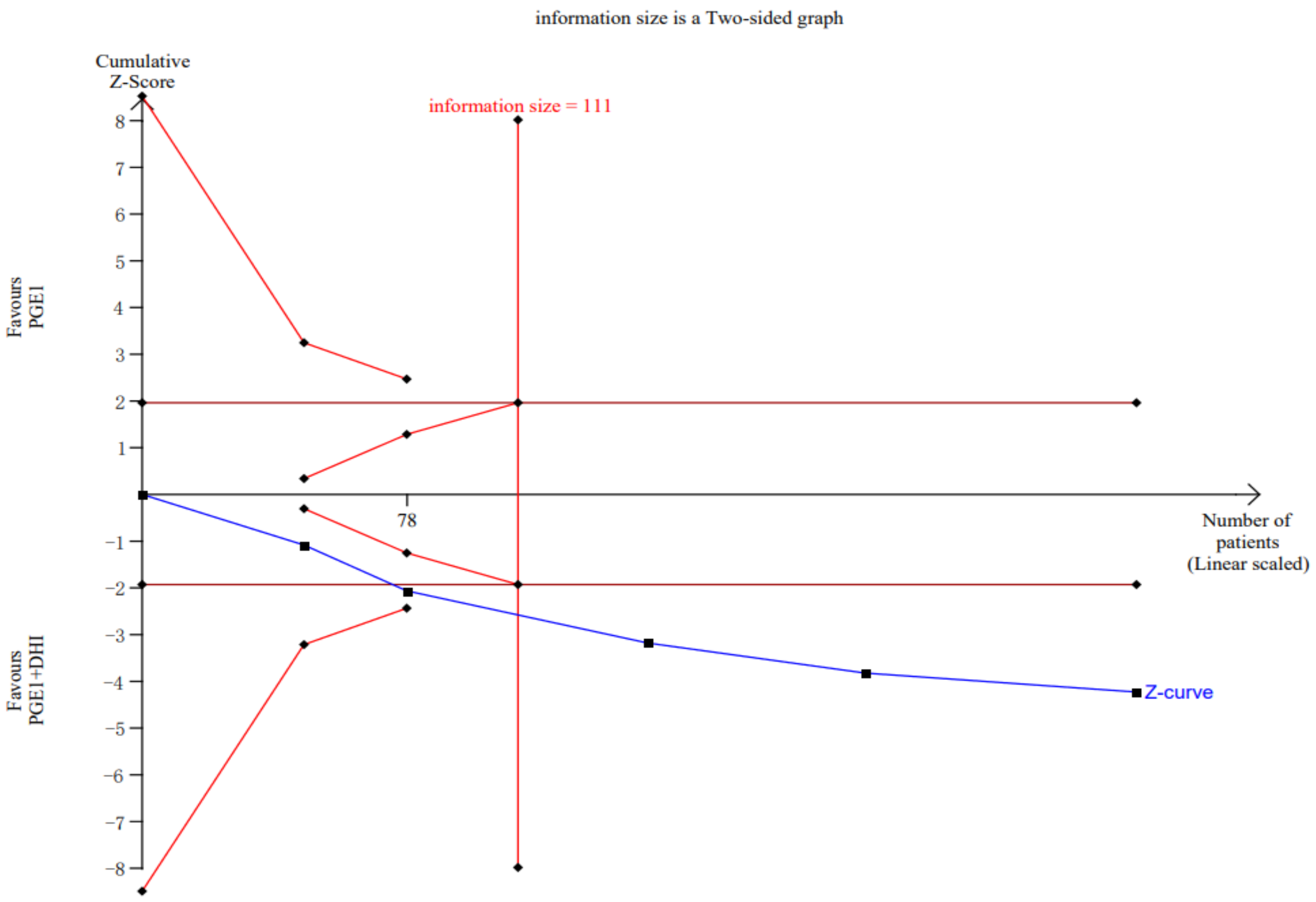


**Figure S10: Trial Sequential Analysis of total effective rate on PGE1+DHI**

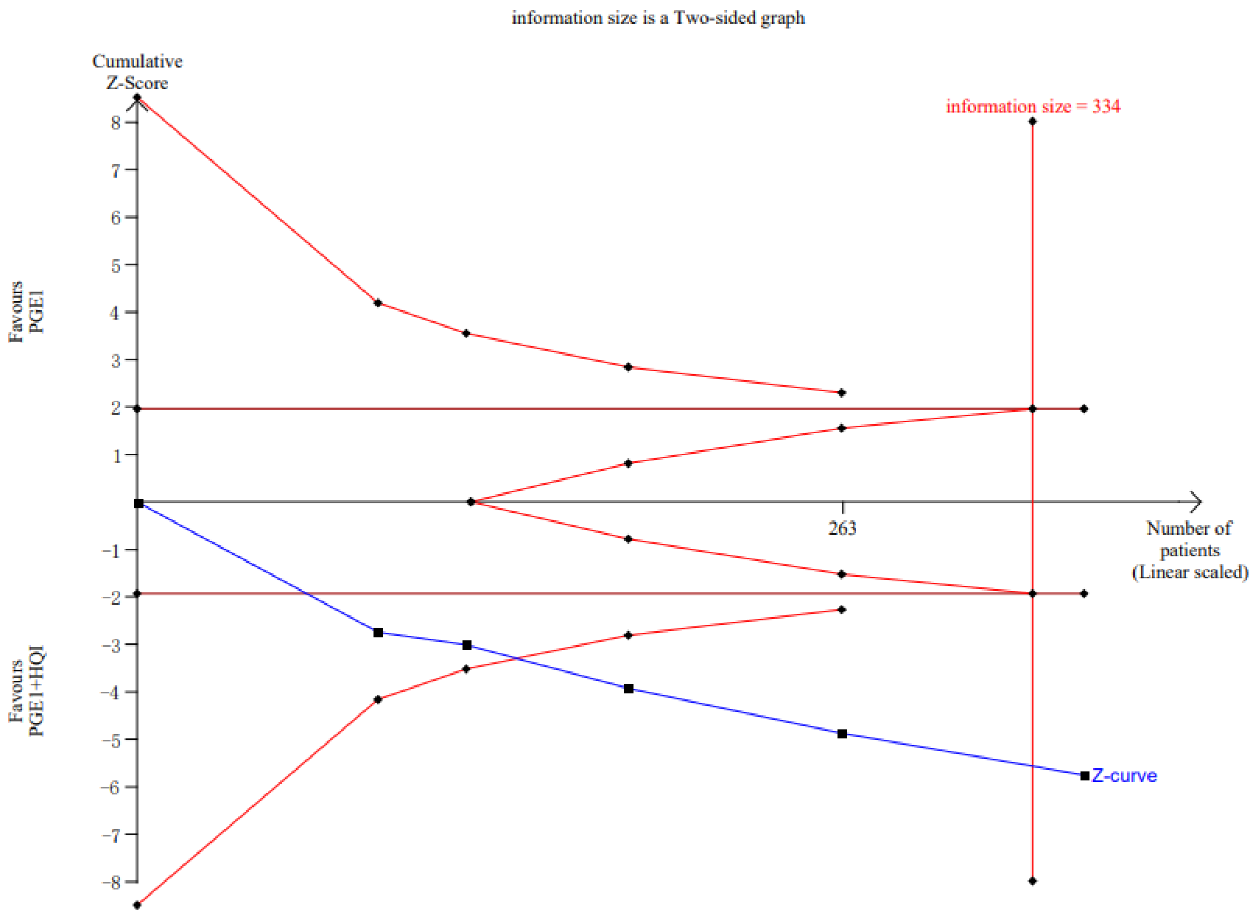


**Figure S11: Trial Sequential Analysis of total effective rate on PGE1+HQI**

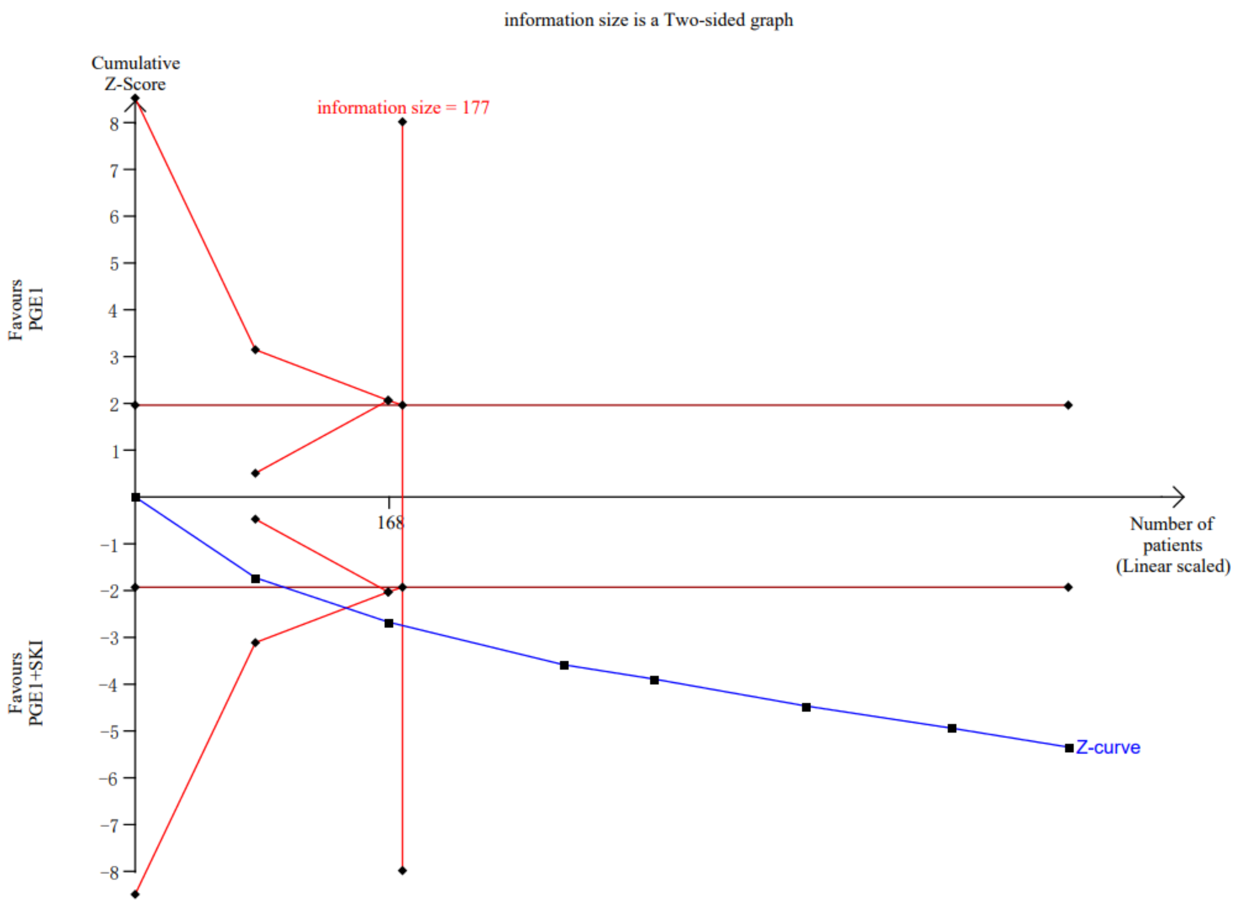


**Figure S12: Trial Sequential Analysis of total effective rate on PGE1+SKI**

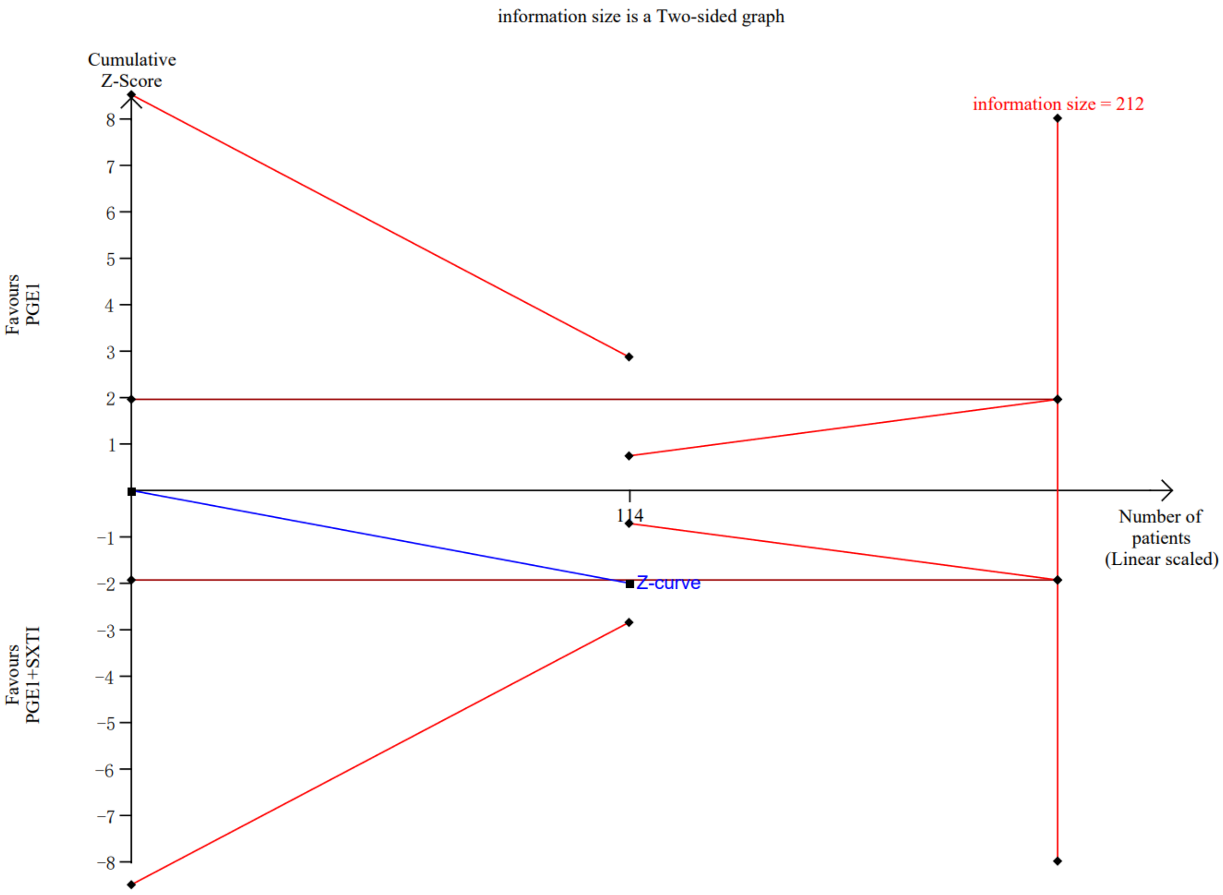


**Figure S13: Trial Sequential Analysis of total effective rate on PGE1+SXTI**

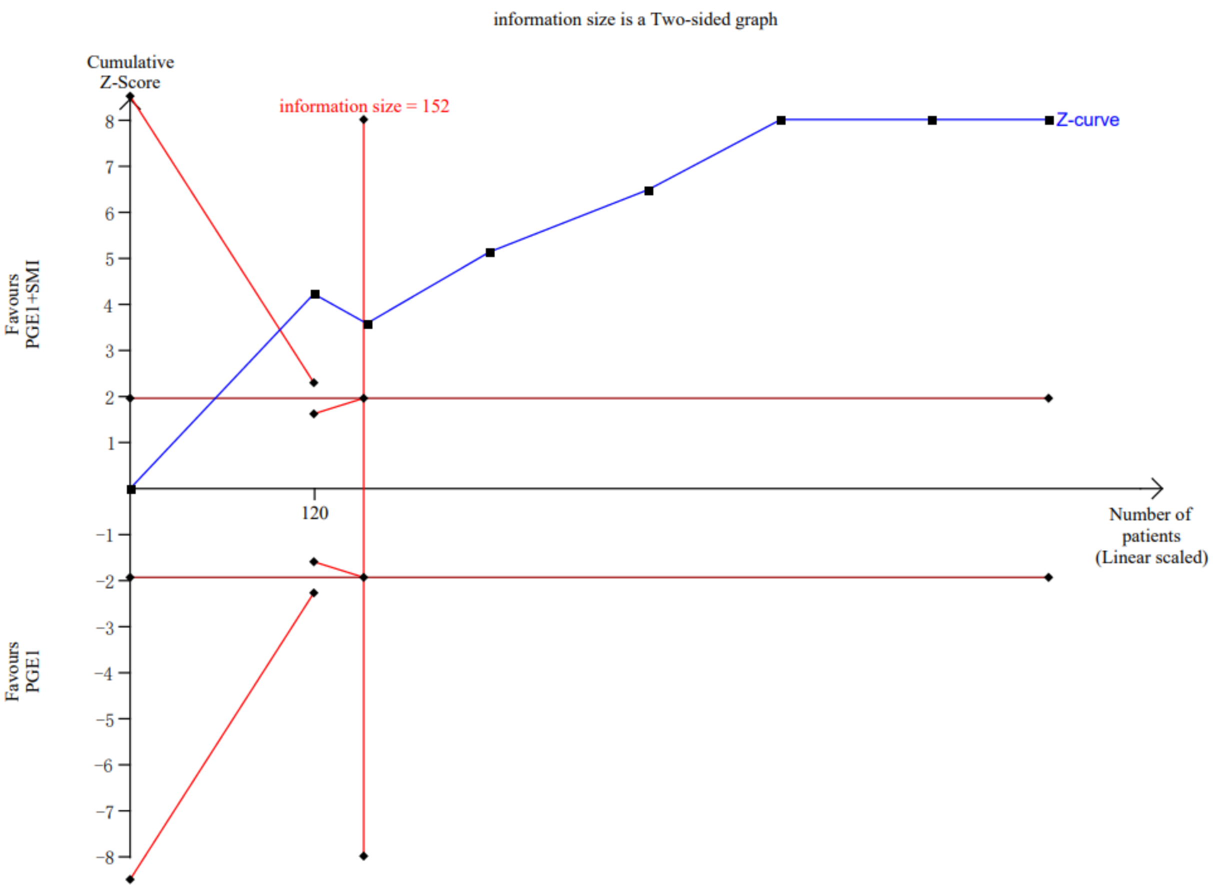


**Figure S14: Trial Sequential Analysis of serum creatinine on PGE1+SMI**

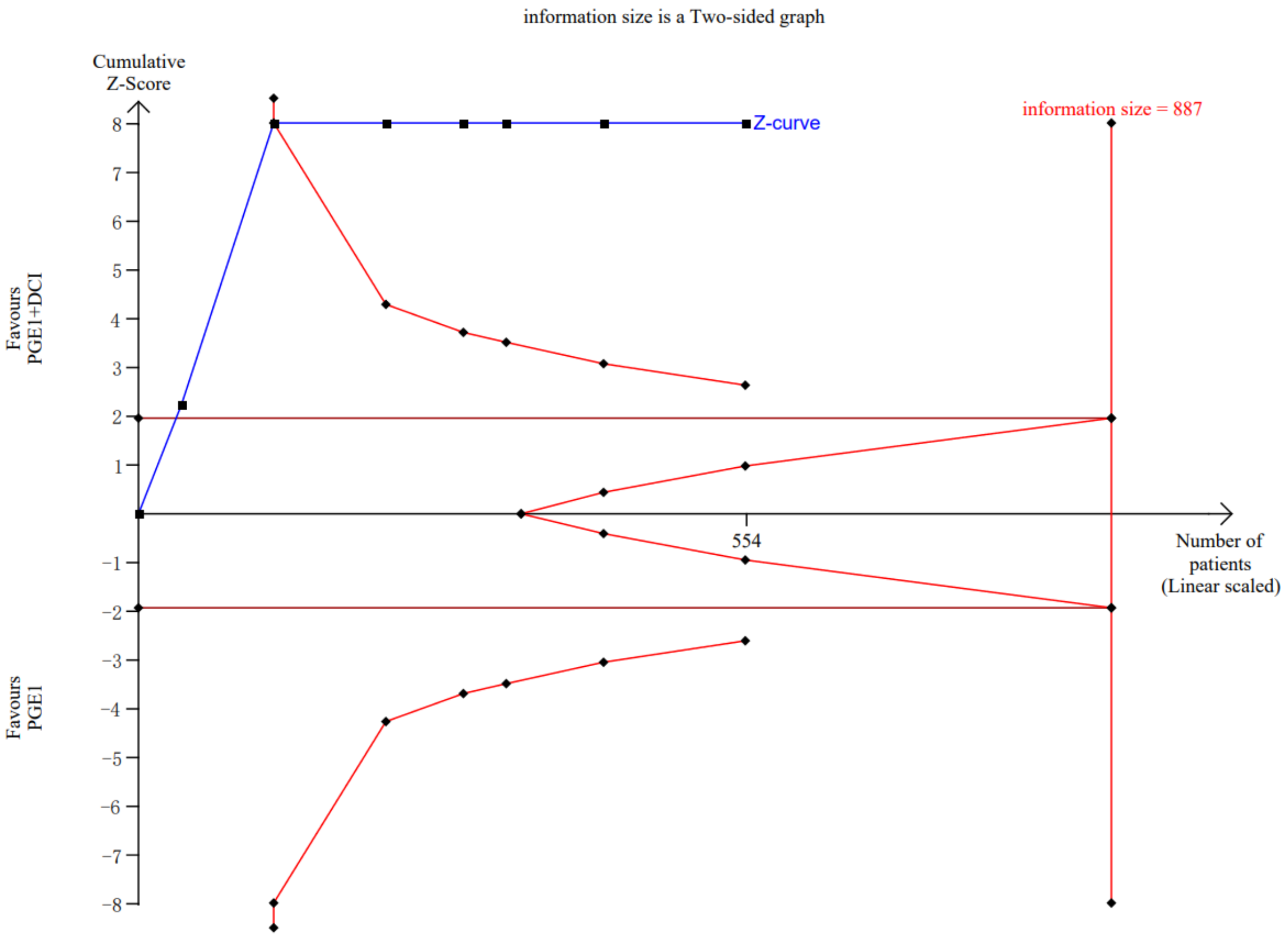


**Figure S15: Trial Sequential Analysis of serum creatinine on PGE1+DCI**

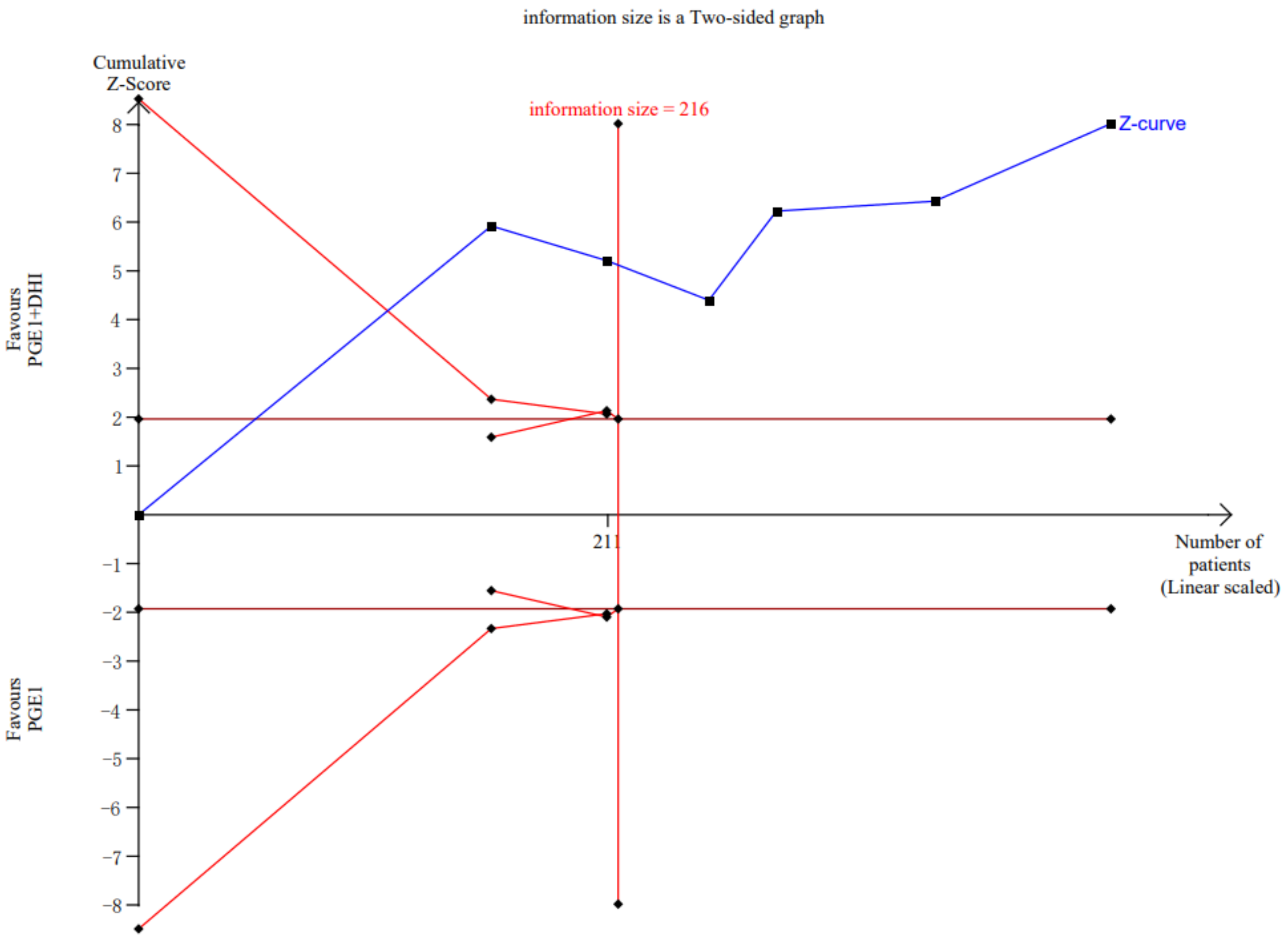


**Figure S16: Trial Sequential Analysis of serum creatinine on PGE1+DHI**

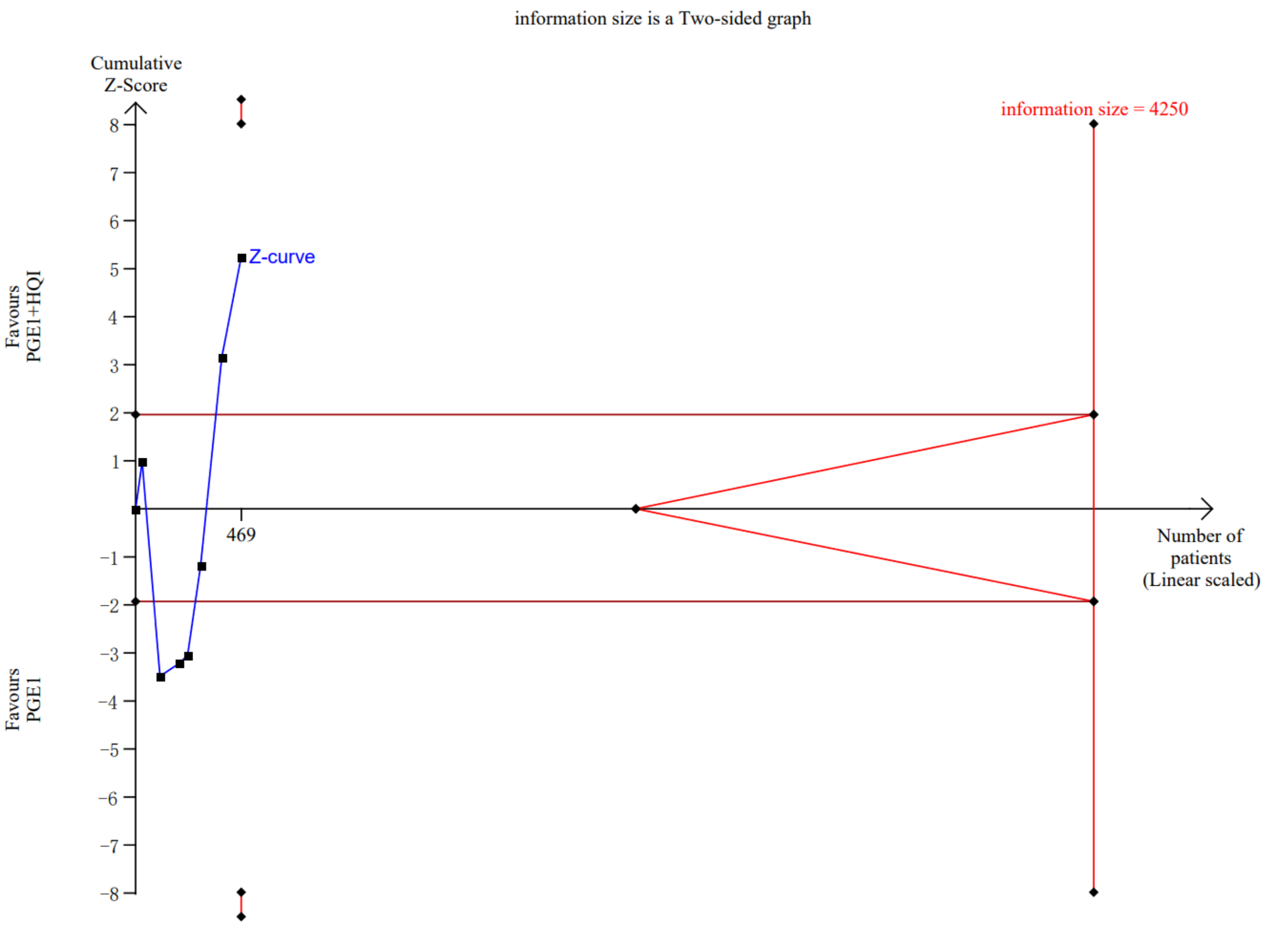


**Figure S17: Trial Sequential Analysis of serum creatinine on PGE1+HQI**

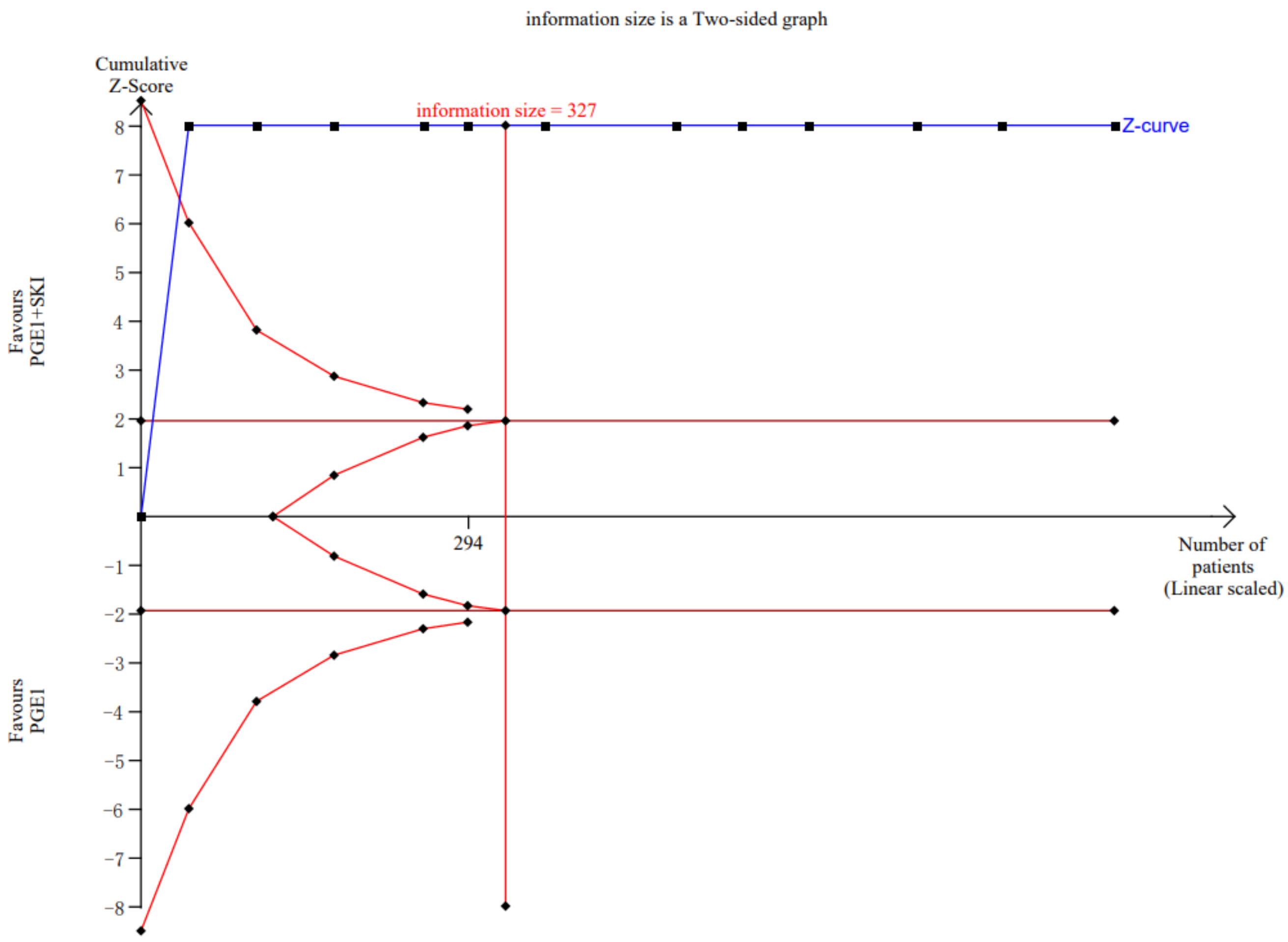


**Figure S18: Trial Sequential Analysis of serum creatinine on PGE1+SKI**

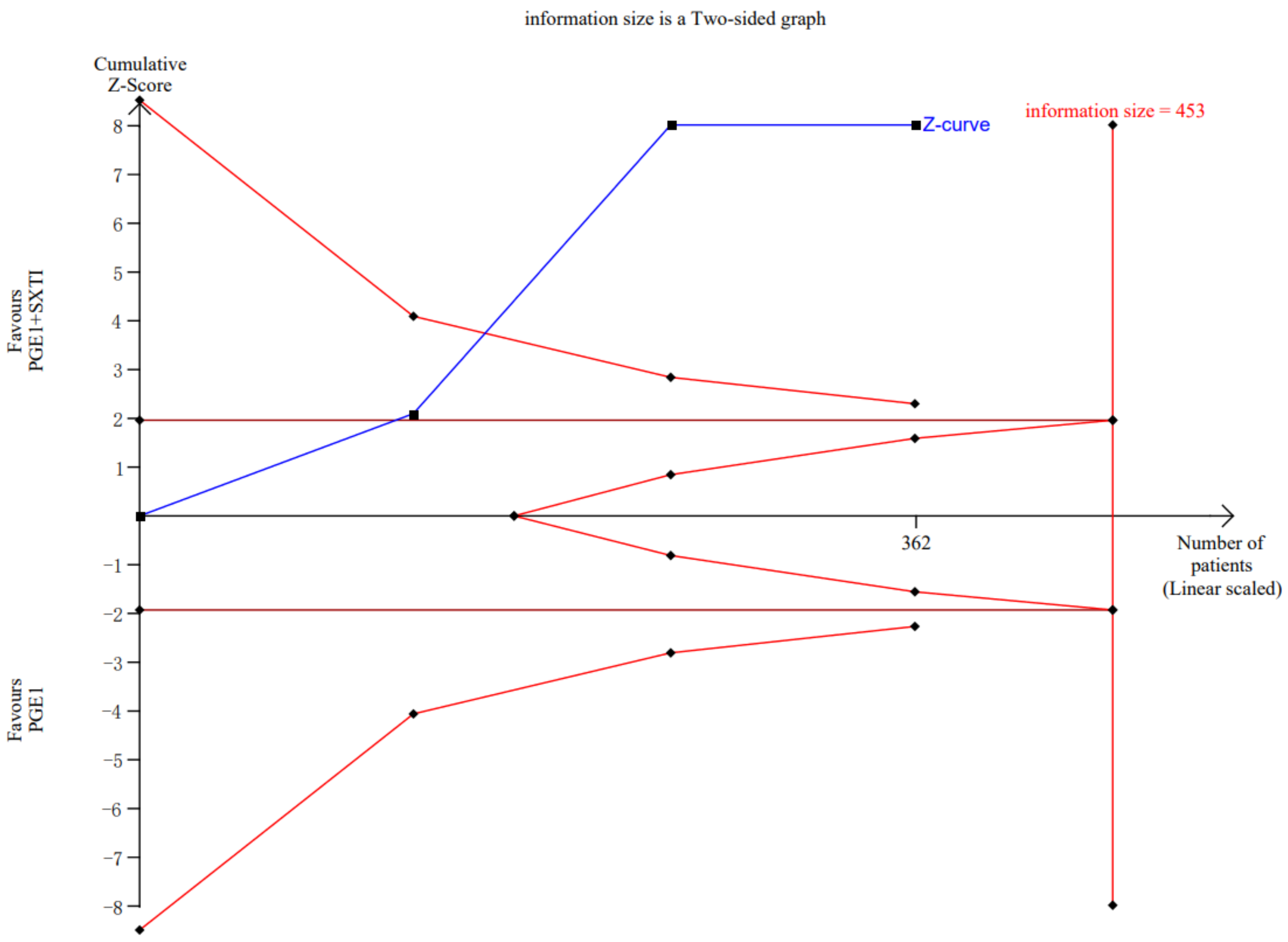


**Figure S19: Trial Sequential Analysis of serum creatinine on PGE1+SXTI**

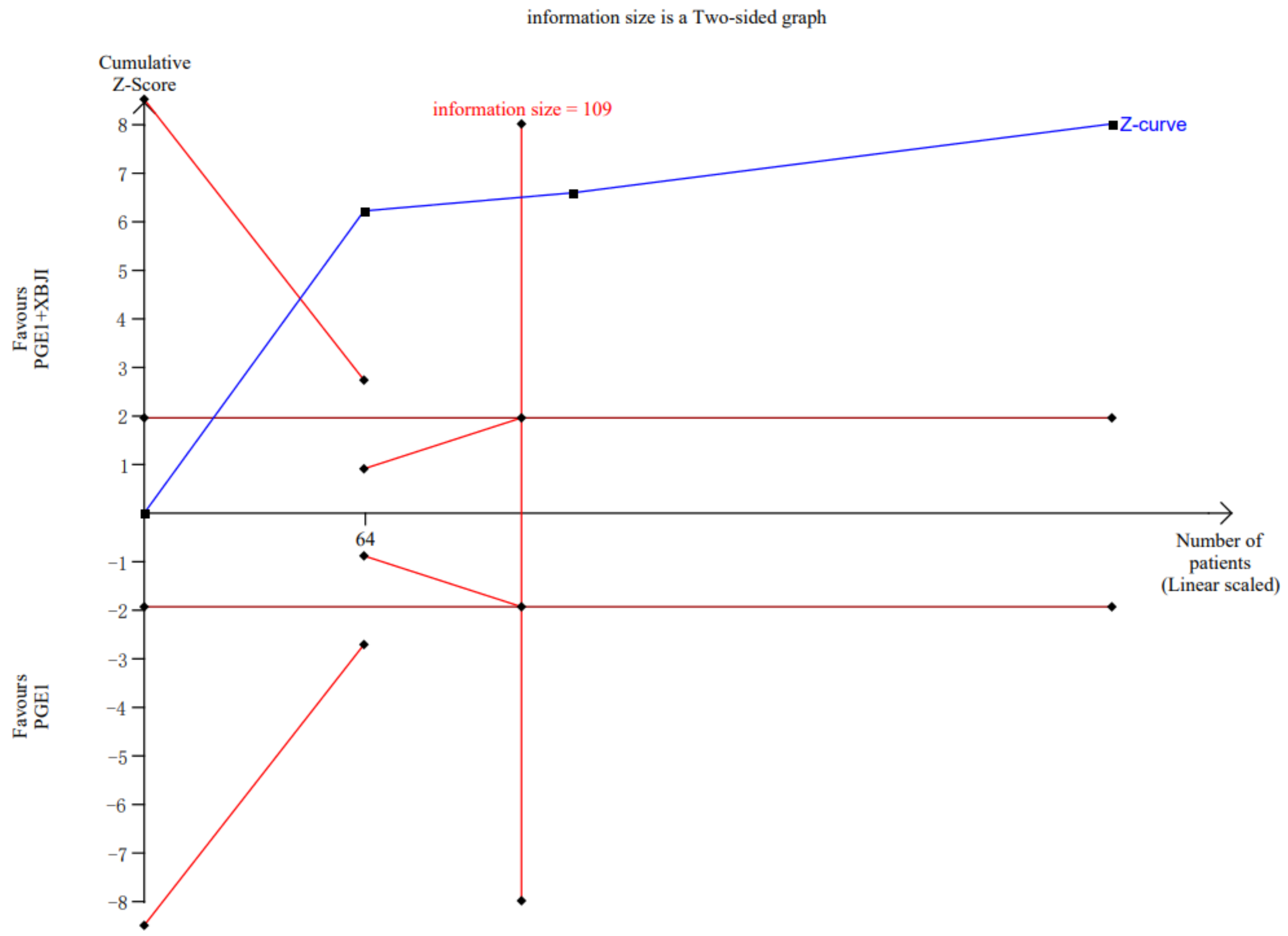


**Figure S20: Trial Sequential Analysis of serum creatinine on PGE1+XBJ**

## *Supplementary Table S2: GRADE assessment*

| **Total effective rate** | direct evidence | | indirect evidence | | Network meta-analysis | |
|---|---|---|---|---|---|---|
| comparison | Risk ratio(95% confidence interval) | Quality of evidence | Risk ratio(95% confidence interval) | Quality of evidence | Risk ratio(95% confidence interval) | Quality of evidence |
| PGE1+DCI v PGE1 | 1.17 (1.02, 1.37) | Low | Not estimable†† | Not estimable†† | 1.17 (1.02, 1.37) | Low |
| PGE1+DHI v PGE1 | 1.28 (1.13, 1.46) | Moderate | Not estimable†† | Not estimable†† | 1.28 (1.13, 1.46) | Moderate |
| PGE1+HQI v PGE1 | 1.43 (1.26, 1.66) | Moderate | Not estimable†† | Not estimable†† | 1.43 (1.26, 1.66) | Moderate |
| PGE1+SKI v PGE1 | 1.2 (1.12, 1.3) | Moderate | Not estimable†† | Not estimable†† | 1.2 (1.12, 1.3) | Moderate |
| PGE1+SMI v PGE1 | 1.24 (1.15, 1.35) | Low | Not estimable†† | Not estimable†† | 1.24 (1.15, 1.35) | Low |
| PGE1+SXTI v PGE1 | 1.18 (0.99, 1.42) | very low | Not estimable†† | Not estimable†† | 1.18 (0.99, 1.42) | very low |
| PGE1+DHI v PGE1+DCI | / | / | 1.09 (0.9, 1.34) | very low | 1.09 (0.9, 1.34) | very low |
| PGE1+HQI v PGE1+DCI | / | / | 1.23 (1, 1.51) | very low | 1.23 (1, 1.51) | very low |
| PGE1+SKI v PGE1+DCI | / | / | 1.03 (0.86, 1.21) | Low | 1.03 (0.86, 1.21) | Low |
| PGE1+SMI v PGE1+DCI | / | / | 1.06 (0.89, 1.24) | Low | 1.06 (0.89, 1.24) | Low |
| PGE1+SXTI v PGE1+DCI | / | / | 1.01 (0.8, 1.28) | Low | 1.01 (0.8, 1.28) | Low |
| PGE1+HQI v PGE1+DHI | / | / | 1.12 (0.93, 1.36) | very low | 1.12 (0.93, 1.36) | very low |
| PGE1+SKI v PGE1+DHI | / | / | 0.94 (0.8, 1.08) | very low | 0.94 (0.8, 1.08) | very low |
| PGE1+SMI v PGE1+DHI | / | / | 0.97 (0.83, 1.13) | Low | 0.97 (0.83, 1.13) | Low |
| PGE1+SXTI v PGE1+DHI | / | / | 0.92 (0.74, 1.15) | Low | 0.92 (0.74, 1.15) | Low |
| PGE1+SKI v PGE1+HQI | / | / | 0.84 (0.71, 0.97) | very low | 0.84 (0.71, 0.97) | very low |
| PGE1+SMI v PGE1+HQI | / | / | 0.87 (0.73, 1) | Low | 0.87 (0.73, 1) | Low |
| PGE1+SXTI v PGE1+HQI | / | / | 0.82 (0.65, 1.04) | Low | 0.82 (0.65, 1.04) | Low |

| | | | | | | |
|---|---|---|---|---|---|---|
| PGE1+SMI v PGE1+SKI | / | / | 1.03 (0.93, 1.15) | very low | 1.03 (0.93, 1.15) | very low |
| PGE1+SXTI v PGE1+SKI | / | / | 0.98 (0.82, 1.2) | Low | 0.98 (0.82, 1.2) | Low |
| PGE1+SXTI v PGE1+SMI | / | / | 0.95 (0.79, 1.16) | very low | 0.95 (0.79, 1.16) | very low |

††Cannot be estimated because the drug was not connected in a loop in the evidence network.

| **Serum Creatinine** | direct evidence | | indirect evidence | | Network meta-analysis | |
|---|---|---|---|---|---|---|
| comparison | Mean difference(95% confidence interval) | Quality of evidence | Mean difference(95% confidence interval) | Quality of evidence | Mean difference(95% confidence interval) | Quality of evidence |
| PGE1+DCI v PGE1 | -1.34 (-2.11, -0.56) | very low | Not estimable†† | Not estimable†† | -1.34 (-2.11, -0.56) | very low |
| PGE1+DHI v PGE1 | -0.87 (-1.7, -0.03) | Low | Not estimable†† | Not estimable†† | -0.87 (-1.7, -0.03) | Low |
| PGE1+HQI v PGE1 | -0.65 (-1.42, 0.13) | Low | Not estimable†† | Not estimable†† | -0.65 (-1.42, 0.13) | Low |
| PGE1+SKI v PGE1 | -1.78 (-2.39, -1.18) | Low | Not estimable†† | Not estimable†† | -1.78 (-2.39, -1.18) | Low |
| PGE1+SMI v PGE1 | -0.83 (-1.6, -0.06) | Moderate | Not estimable†† | Not estimable†† | -0.83 (-1.6, -0.06) | Moderate |
| PGE1+SXTI v PGE1 | -0.91 (-2.09, 0.28) | very low | Not estimable†† | Not estimable†† | -0.91 (-2.09, 0.28) | very low |
| PGE1+XBJI v PGE1 | -1.1 (-2.27, 0.07) | Low | Not estimable†† | Not estimable†† | -1.1 (-2.27, 0.07) | Low |
| PGE1+DHI v PGE1+DCI | / | / | 0.48 (-0.67, 1.62) | Low | 0.48 (-0.67, 1.62) | Low |
| PGE1+HQI v PGE1+DCI | / | / | 0.69 (-0.41, 1.8) | Low | 0.69 (-0.41, 1.8) | Low |
| PGE1+SKI v PGE1+DCI | / | / | -0.44 (-1.43, 0.54) | very low | -0.44 (-1.43, 0.54) | very low |
| PGE1+SMI v PGE1+DCI | / | / | 0.51 (-0.59, 1.6) | Low | 0.51 (-0.59, 1.6) | Low |

| | | | | | | |
|---|---|---|---|---|---|---|
| PGE1+SXTI v PGE1+DCI | / | / | 0.43 (-0.98, 1.86) | Low | 0.43 (-0.98, 1.86) | Low |
| PGE1+XBJI v PGE1+DCI | / | / | 0.24 (-1.17, 1.65) | very low | 0.24 (-1.17, 1.65) | very low |
| PGE1+HQI v PGE1+DHI | / | / | 0.22 (-0.93, 1.36) | very low | 0.22 (-0.93, 1.36) | very low |
| PGE1+SKI v PGE1+DHI | / | / | -0.91 (-1.95, 0.11) | Low | -0.91 (-1.95, 0.11) | Low |
| PGE1+SMI v PGE1+DHI | / | / | 0.03 (-1.1, 1.17) | Low | 0.03 (-1.1, 1.17) | Low |
| PGE1+SXTI v PGE1+DHI | / | / | -0.04 (-1.49, 1.42) | Low | -0.04 (-1.49, 1.42) | Low |
| PGE1+XBJI v PGE1+DHI | / | / | -0.24 (-1.68, 1.21) | very low | -0.24 (-1.68, 1.21) | very low |
| PGE1+SKI v PGE1+HQI | / | / | -1.13 (-2.12, -0.16) | very low | -1.13 (-2.12, -0.16) | very low |
| PGE1+SMI v PGE1+HQI | / | / | -0.19 (-1.28, 0.91) | very low | -0.19 (-1.28, 0.91) | very low |
| PGE1+SXTI v PGE1+HQI | / | / | -0.26 (-1.68, 1.16) | Low | -0.26 (-1.68, 1.16) | Low |
| PGE1+XBJI v PGE1+HQI | / | / | -0.46 (-1.86, 0.95) | Low | -0.46 (-1.86, 0.95) | Low |
| PGE1+SMI v PGE1+SKI | / | / | 0.94 (-0.03, 1.93) | Low | 0.94 (-0.03, 1.93) | Low |
| PGE1+SXTI v PGE1+SKI | / | / | 0.87 (-0.45, 2.21) | Low | 0.87 (-0.45, 2.21) | Low |
| PGE1+XBJI v PGE1+SKI | / | / | 0.67 (-0.64, 2) | very low | 0.67 (-0.64, 2) | very low |
| PGE1+SXTI v PGE1+SMI | / | / | -0.07 (-1.48, 1.34) | Low | -0.07 (-1.48, 1.34) | Low |
| PGE1+XBJI v PGE1+SMI | / | / | -0.27 (-1.67, 1.14) | very low | -0.27 (-1.67, 1.14) | very low |
| PGE1+XBJI v PGE1+SXTI | / | / | -0.2 (-1.87, 1.47) | Low | -0.2 (-1.87, 1.47) | Low |

††Cannot be estimated because the drug was not connected in a loop in the evidence network.

| Blood Urea Nitrogen | direct evidence | | indirect evidence | | Network meta-analysis | |
|---|---|---|---|---|---|---|
| comparison | Mean difference(95% confidence interval) | Quality of evidence | Mean difference(95% confidence interval) | Quality of evidence | Mean difference(95% confidence interval) | Quality of evidence |
| PGE1+DCI v PGE1 | -1.11 (-1.93, -0.29) | Low | Not estimable†† | Not estimable†† | -1.1(-1.9,-0.29) | Low |
| PGE1+DHI v PGE1 | -0.73 (-1.66, 0.19) | Moderate | Not estimable†† | Not estimable†† | -0.74(-1.7,0.19) | Moderate |
| PGE1+HQI v PGE1 | -0.59 (-1.34, 0.17) | Low | Not estimable†† | Not estimable†† | -0.59(-1.3,0.17) | Low |
| PGE1+SKI v PGE1 | -1.16 (-1.73, -0.61) | Moderate | Not estimable†† | Not estimable†† | -1.2(-1.7,-0.61) | Moderate |
| PGE1+SMI v PGE1 | -0.66 (-1.35, 0.03) | Moderate | Not estimable†† | Not estimable†† | -0.65(-1.3,0.035) | Moderate |
| PGE1+SXTI v PGE1 | -1 (-1.91, -0.09) | Low | Not estimable†† | Not estimable†† | -1.0(-1.9,-0.082) | Low |
| PGE1+XBJI v PGE1 | -0.83 (-1.89, 0.22) | Low | Not estimable†† | Not estimable†† | -0.83(-1.9,0.24) | Low |
| PGE1+DHI v PGE1+DCI | / | / | 0.38 (-0.86, 1.61) | Low | 0.38 (-0.86, 1.61) | Low |
| PGE1+HQI v PGE1+DCI | / | / | 0.52 (-0.59, 1.64) | very low | 0.52 (-0.59, 1.64) | very low |
| PGE1+SKI v PGE1+DCI | / | / | -0.06 (-1.06, 0.93) | Low | -0.06 (-1.06, 0.93) | Low |
| PGE1+SMI v PGE1+DCI | / | / | 0.45 (-0.62, 1.53) | Low | 0.45 (-0.62, 1.53) | Low |
| PGE1+SXTI v PGE1+DCI | / | / | 0.11 (-1.11, 1.34) | very low | 0.11 (-1.11, 1.34) | very low |
| PGE1+XBJI v PGE1+DCI | / | / | 0.28 (-1.05, 1.61) | very low | 0.28 (-1.05, 1.61) | very low |
| PGE1+HQI v PGE1+DHI | / | / | 0.15 (-1.05, 1.34) | Low | 0.15 (-1.05, 1.34) | Low |

| | | | | | | |
|---|---|---|---|---|---|---|
| PGE1+SKI v PGE1+DHI | / | / | -0.43 (-1.52, 0.65) | Low | -0.43 (-1.52, 0.65) | Low |
| PGE1+SMI v PGE1+DHI | / | / | 0.08 (-1.08, 1.23) | Low | 0.08 (-1.08, 1.23) | Low |
| PGE1+SXTI v PGE1+DHI | / | / | -0.26 (-1.57, 1.04) | Low | -0.26 (-1.57, 1.04) | Low |
| PGE1+XBJI v PGE1+DHI | / | / | -0.1 (-1.5, 1.31) | very low | -0.1 (-1.5, 1.31) | very low |
| PGE1+SKI v PGE1+HQI | / | / | -0.58 (-1.53, 0.36) | Low | -0.58 (-1.53, 0.36) | Low |
| PGE1+SMI v PGE1+HQI | / | / | -0.07 (-1.09, 0.95) | very low | -0.07 (-1.09, 0.95) | very low |
| PGE1+SXTI v PGE1+HQI | / | / | -0.41 (-1.59, 0.77) | very low | -0.41 (-1.59, 0.77) | very low |
| PGE1+XBJI v PGE1+HQI | / | / | -0.25 (-1.54, 1.05) | Low | -0.25 (-1.54, 1.05) | Low |
| PGE1+SMI v PGE1+SKI | / | / | 0.51 (-0.38, 1.41) | Low | 0.51 (-0.38, 1.41) | Low |
| PGE1+SXTI v PGE1+SKI | / | / | 0.17 (-0.9, 1.25) | very low | 0.17 (-0.9, 1.25) | very low |
| PGE1+XBJI v PGE1+SKI | / | / | 0.33 (-0.86, 1.53) | Low | 0.33 (-0.86, 1.53) | Low |
| PGE1+SXTI v PGE1+SMI | / | / | -0.34 (-1.48, 0.8) | very low | -0.34 (-1.48, 0.8) | very low |
| PGE1+XBJI v PGE1+SMI | / | / | -0.17 (-1.44, 1.08) | Low | -0.17 (-1.44, 1.08) | Low |
| PGE1+XBJI v PGE1+SXTI | / | / | 0.16 (-1.23, 1.57) | Low | 0.16 (-1.23, 1.57) | Low |

††Cannot be estimated because the drug was not connected in a loop in the evidence network.

| 24h Urine Albumin | direct evidence | | indirect evidence | | Network meta-analysis | |
|---|---|---|---|---|---|---|
| comparison | Mean difference(95% confidence interval) | Quality of evidence | Mean difference(95% confidence interval) | Quality of evidence | Mean difference(95% confidence interval) | Quality of evidence |

| | | | | | | |
|---|---|---|---|---|---|---|
| PGE1+DCI v PGE1 | -0.83 (-2.08, 0.43) | Low | Not estimable†† | Not estimable†† | -0.83 (-2.08, 0.43) | Low |
| PGE1+DHI v PGE1 | -2.49 (-3.96, -1.03) | Low | Not estimable†† | Not estimable†† | -2.49 (-3.96, -1.03) | Low |
| PGE1+HQI v PGE1 | -1.15 (-2.28, -0.03) | Low | Not estimable†† | Not estimable†† | -1.15 (-2.28, -0.03) | Low |
| PGE1+SKI v PGE1 | -0.34 (-1.17, 0.49) | Low | Not estimable†† | Not estimable†† | -0.34 (-1.17, 0.49) | Low |
| PGE1+XBJI v PGE1 | -1.26 (-2.7, 0.18) | Very low | Not estimable†† | Not estimable†† | -1.26 (-2.7, 0.18) | Very low |
| PGE1+DHI v PGE1+DCI | / | / | -1.67 (-3.6, 0.25) | Low | -1.67 (-3.6, 0.25) | Low |
| PGE1+HQI v PGE1+DCI | / | / | -0.32 (-2.03, 1.35) | Very low | -0.32 (-2.03, 1.35) | Low |
| PGE1+SKI v PGE1+DCI | / | / | 0.48 (-1.02, 1.99) | Low | 0.48 (-1.02, 1.99) | Low |
| PGE1+XBJI v PGE1+DCI | / | / | -0.43 (-2.35, 1.47) | Low | -0.43 (-2.35, 1.47) | Low |
| PGE1+HQI v PGE1+DHI | / | / | 1.34 (-0.49, 3.19) | Very low | 1.34 (-0.49, 3.19) | Very low |
| PGE1+SKI v PGE1+DHI | / | / | 2.15 (0.48, 3.84) | Low | 2.15 (0.48, 3.84) | Low |
| PGE1+XBJI v PGE1+DHI | / | / | 1.23 (-0.81, 3.29) | Low | 1.23 (-0.81, 3.29) | Low |
| PGE1+SKI v PGE1+HQI | / | / | 0.81 (-0.57, 2.21) | Low | 0.81 (-0.57, 2.21) | Low |
| PGE1+XBJI v PGE1+HQI | / | / | -0.11 (-1.93, 1.72) | Low | -0.11 (-1.93, 1.72) | Low |
| PGE1+XBJI v PGE1+SKI | / | / | -0.92 (-2.58, 0.74) | Very low | -0.92 (-2.58, 0.74) | Very low |

††Cannot be estimated because the drug was not connected in a loop in the evidence network.

| **Urinary Albumin excretion rates** | **direct evidence** | | **indirect evidence** | | **Network meta-analysis** | |
|---|---|---|---|---|---|---|

| comparison | Mean difference(95% confidence interval) | Quality of evidence | Mean difference(95% confidence interval) | Quality of evidence | Mean difference(95% confidence interval) | Quality of evidence |
|---|---|---|---|---|---|---|
| PGE1+DCI v PGE1 | -1.47 (-2.48, -0.47) | Moderate | Not estimable†† | Not estimable†† | -1.47 (-2.48, -0.47) | Moderate |
| PGE1+DHI v PGE1 | -1.57 (-2.25, -0.9) | Moderate | Not estimable†† | Not estimable†† | -1.57 (-2.25, -0.9) | Moderate |
| PGE1+HQI v PGE1 | -1.34 (-2.14, -0.56) | Low | Not estimable†† | Not estimable†† | -1.34 (-2.14, -0.56) | Low |
| PGE1+SKI v PGE1 | -0.97 (-2.22, 0.26) | Low | Not estimable†† | Not estimable†† | -0.97 (-2.22, 0.26) | Low |
| PGE1+SMI v PGE1 | -1.08 (-1.94, -0.22) | Moderate | Not estimable†† | Not estimable†† | -1.08 (-1.94, -0.22) | Moderate |
| PGE1+SXTI v PGE1 | -0.72 (-1.95, 0.5) | Low | Not estimable†† | Not estimable†† | -0.72 (-1.95, 0.5) | Low |
| PGE1+XBJI v PGE1 | -0.65 (-1.86, 0.57) | Moderate | Not estimable†† | Not estimable†† | -0.65 (-1.86, 0.57) | Moderate |
| PGE1+DHI v PGE1+DCI | / | / | -0.1 (-1.31, 1.1) | Low | -0.1 (-1.31, 1.1) | Low |
| PGE1+HQI v PGE1+DCI | / | / | 0.13 (-1.15, 1.4) | very low | 0.13 (-1.15, 1.4) | very low |
| PGE1+SKI v PGE1+DCI | / | / | 0.5 (-1.11, 2.09) | Low | 0.5 (-1.11, 2.09) | Low |
| PGE1+SMI v PGE1+DCI | / | / | 0.39 (-0.93, 1.7) | Low | 0.39 (-0.93, 1.7) | Low |
| PGE1+SXTI v PGE1+DCI | / | / | 0.75 (-0.83, 2.34) | very low | 0.75 (-0.83, 2.34) | very low |
| PGE1+XBJI v PGE1+DCI | / | / | 0.82 (-0.75, 2.4) | Low | 0.82 (-0.75, 2.4) | Low |
| PGE1+HQI v PGE1+DHI | / | / | 0.23 (-0.81, 1.26) | very low | 0.23 (-0.81, 1.26) | very low |
| PGE1+SKI v PGE1+DHI | / | / | 0.6 (-0.81, 2.01) | Low | 0.6 (-0.81, 2.01) | Low |
| PGE1+SMI v PGE1+DHI | / | / | 0.49 (-0.59, 1.59) | Low | 0.49 (-0.59, 1.59) | Low |
| PGE1+SXTI v PGE1+DHI | / | / | 0.85 (-0.54, 2.26) | very low | 0.85 (-0.54, 2.26) | very low |

| | | | | | | |
|---|---|---|---|---|---|---|
| PGE1+XBJI v PGE1+DHI | / | / | 0.92 (-0.46, 2.32) | very low | 0.92 (-0.46, 2.32) | very low |
| PGE1+SKI v PGE1+HQI | / | / | 0.37 (-1.1, 1.84) | very low | 0.37 (-1.1, 1.84) | very low |
| PGE1+SMI v PGE1+HQI | / | / | 0.26 (-0.9, 1.44) | Low | 0.26 (-0.9, 1.44) | Low |
| PGE1+SXTI v PGE1+HQI | / | / | 0.63 (-0.83, 2.09) | Low | 0.63 (-0.83, 2.09) | Low |
| PGE1+XBJI v PGE1+HQI | / | / | 0.7 (-0.75, 2.16) | Low | 0.7 (-0.75, 2.16) | Low |
| PGE1+SMI v PGE1+SKI | / | / | -0.11 (-1.61, 1.41) | very low | -0.11 (-1.61, 1.41) | very low |
| PGE1+SXTI v PGE1+SKI | / | / | 0.26 (-1.48, 2.01) | Low | 0.26 (-1.48, 2.01) | Low |
| PGE1+XBJI v PGE1+SKI | / | / | 0.33 (-1.41, 2.07) | very low | 0.33 (-1.41, 2.07) | very low |
| PGE1+SXTI v PGE1+SMI | / | / | 0.37 (-1.14, 1.85) | very low | 0.37 (-1.14, 1.85) | very low |
| PGE1+XBJI v PGE1+SMI | / | / | 0.43 (-1.06, 1.92) | Low | 0.43 (-1.06, 1.92) | Low |
| PGE1+XBJI v PGE1+SXTI | / | / | 0.07 (-1.65, 1.8) | Low | 0.07 (-1.65, 1.8) | Low |

††Cannot be estimated because the drug was not connected in a loop in the evidence network.

| Urinary beta 2-microglobulin | direct evidence | | indirect evidence | | Network meta-analysis | |
|---|---|---|---|---|---|---|
| comparison | Mean difference(95% confidence interval) | Quality of evidence | Mean difference(95% confidence interval) | Quality of evidence | Mean difference(95% confidence interval) | Quality of evidence |
| PGE1+DCI v PGE1 | -0.72 (-1.99, 0.53) | very low | Not estimable†† | Not estimable†† | -0.72 (-1.99, 0.53) | very low |
| PGE1+DHI v PGE1 | -1.37 (-2, -0.81) | Low | Not estimable†† | Not estimable†† | -1.37 (-2, -0.81) | Low |
| PGE1+HQI v PGE1 | -1.79 (-2.69, -0.89) | Moderate | Not estimable†† | Not estimable†† | -1.79 (-2.69, -0.89) | Moderate |
| PGE1+SMI v PGE1 | -0.44 (-1.16, 0.31) | Moderate | Not estimable†† | Not estimable†† | -0.44 (-1.16, 0.31) | Moderate |
| PGE1+SXTI v PGE1 | -1.43 (-2.16, -0.73) | Low | Not estimable†† | Not estimable†† | -1.43 (-2.16, -0.73) | Low |

| | | | | | | |
|---|---|---|---|---|---|---|
| PGE1+XBJI v PGE1 | -0.57 (-1.84, 0.7) | Low | Not estimable†† | Not estimable†† | -0.57 (-1.84, 0.7) | Low |
| PGE1+DHI v PGE1+DCI | / | / | -0.65 (-2.08, 0.71) | Low | -0.65 (-2.08, 0.71) | Low |
| PGE1+HQI v PGE1+DCI | / | / | -1.07 (-2.62, 0.48) | Low | -1.07 (-2.62, 0.48) | Low |
| PGE1+SMI v PGE1+DCI | / | / | 0.28 (-1.16, 1.77) | very low | 0.28 (-1.16, 1.77) | very low |
| PGE1+SXTI v PGE1+DCI | / | / | -0.71 (-2.17, 0.74) | very low | -0.71 (-2.17, 0.74) | very low |
| PGE1+XBJI v PGE1+DCI | / | / | 0.15 (-1.64, 1.95) | very low | 0.15 (-1.64, 1.95) | very low |
| PGE1+HQI v PGE1+DHI | / | / | -0.42 (-1.46, 0.69) | Low | -0.42 (-1.46, 0.69) | Low |
| PGE1+SMI v PGE1+DHI | / | / | 0.92 (0.04, 1.93) | Low | 0.92 (0.04, 1.93) | Low |
| PGE1+SXTI v PGE1+DHI | / | / | -0.07 (-0.96, 0.89) | Low | -0.07 (-0.96, 0.89) | Low |
| PGE1+XBJI v PGE1+DHI | / | / | 0.8 (-0.56, 2.24) | very low | 0.8 (-0.56, 2.24) | very low |
| PGE1+SMI v PGE1+HQI | / | / | 1.35 (0.21, 2.53) | Low | 1.35 (0.21, 2.53) | Low |
| PGE1+SXTI v PGE1+HQI | / | / | 0.36 (-0.79, 1.5) | very low | 0.36 (-0.79, 1.5) | very low |
| PGE1+XBJI v PGE1+HQI | / | / | 1.22 (-0.34, 2.79) | very low | 1.22 (-0.34, 2.79) | very low |
| PGE1+SXTI v PGE1+SMI | / | / | -0.99 (-2.05, 0.01) | Low | -0.99 (-2.05, 0.01) | Low |
| PGE1+XBJI v PGE1+SMI | / | / | -0.13 (-1.61, 1.32) | very low | -0.13 (-1.61, 1.32) | very low |
| PGE1+XBJI v PGE1+SXTI | / | / | 0.86 (-0.59, 2.33) | very low | 0.86 (-0.59, 2.33) | very low |

††Cannot be estimated because the drug was not connected in a loop in the evidence network.

## *Supplementary Table S3-S10: Search Strategy*

**Table S3. Search Strategy: PubMed**

| | |
|---|---|
| #1 | "Diabetic Nephropathies"[Mesh] |
| #2 | (((((((((((((((Nephropathies, Diabetic[Title/Abstract]) OR (Nephropathy, Diabetic[Title/Abstract])) OR (Diabetic Nephropathy[Title/Abstract])) OR (Diabetic Kidney Disease[Title/Abstract])) OR (Diabetic Kidney Diseases[Title/Abstract])) OR (Kidney Disease, Diabetic[Title/Abstract])) OR (Kidney Diseases, Diabetic[Title/Abstract])) OR (Diabetic Glomerulosclerosis[Title/Abstract])) OR (Glomerulosclerosis, Diabetic[Title/Abstract])) OR (Intracapillary Glomerulosclerosis[Title/Abstract])) OR (Nodular Glomerulosclerosis[Title/Abstract])) OR (Glomerulosclerosis, Nodular[Title/Abstract])) OR (Kimmelstiel-Wilson Syndrome[Title/Abstract])) OR (Kimmelstiel Wilson Syndrome[Title/Abstract])) OR (Syndrome, Kimmelstiel-Wilson[Title/Abstract])) OR (Kimmelstiel-Wilson Disease[Title/Abstract])) OR (Kimmelstiel Wilson Disease[Title/Abstract]) |
| #3 | #1 OR #2 |
| #4 | injection*[Title/Abstract] |
| #5 | "Alprostadil"[Mesh] |
| #6 | (((((((((((((((PGE1alpha[Title/Abstract]) OR (Prostaglandin E1alpha[Title/Abstract])) OR (PGE1[Title/Abstract])) OR (Lipo-PGE1[Title/Abstract])) OR (Lipo PGE1[Title/Abstract])) OR (Prostaglandin E1[Title/Abstract])) OR (Caverject[Title/Abstract])) OR (Edex[Title/Abstract])) OR (Prostavasin[Title/Abstract])) OR (Muse[Title/Abstract])) OR (Viridal[Title/Abstract])) OR (Vasaprostan[Title/Abstract])) OR (Minprog[Title/Abstract])) OR (Sugiran[Title/Abstract])) OR (Prostin VR[Title/Abstract])) OR (Prostine VR[Title/Abstract]) |
| #7 | #5 OR #6 |
| #8 | #3 AND #4 AND #7 |

**Table S4. Search Strategy: Embase**

| | |
|---|---|
| #1 | 'diabetic nephropathy'/exp |
| #2 | 'nephropathies, diabetic':ab,ti,kw |
| #3 | 'nephropathy, diabetic':ab,ti,kw |
| #4 | 'diabetic nephropathy':ab,ti,kw |
| #5 | 'diabetic kidney disease':ab,ti,kw |
| #6 | 'diabetic kidney diseases':ab,ti,kw |
| #7 | 'kidney disease, diabetic':ab,ti,kw |
| #8 | 'kidney diseases, diabetic':ab,ti,kw |
| #9 | 'diabetic glomerulosclerosis':ab,ti,kw |
| #10 | 'glomerulosclerosis, diabetic':ab,ti,kw |
| #11 | 'intracapillary glomerulosclerosis':ab,ti,kw |
| #12 | 'nodular glomerulosclerosis':ab,ti,kw |
| #13 | 'glomerulosclerosis, nodular':ab,ti,kw |
| #14 | 'kimmelstiel-wilson syndrome':ab,ti,kw |
| #15 | 'kimmelstiel wilson syndrome':ab,ti,kw |
| #16 | 'syndrome, kimmelstiel-wilson':ab,ti,kw |

| | |
|---|---|
| #17 | 'kimmelstiel-wilson disease':ab,ti,kw |
| #18 | 'kimmelstiel wilson disease':ab,ti,kw |
| #19 | #1 OR #2 OR #3 OR #4 OR #5 OR #6 OR #7 OR #8 OR #9 OR #10 OR #11 OR #12 OR #13 OR #14 OR #15 OR #16 OR #17 OR #18 |
| #20 | 'injection*':ab,ti,kw |
| #21 | 'prostaglandin e1'/exp |
| #22 | 'alprostadil':ab,ti,kw |
| #23 | 'pge1alpha':ab,ti,kw |
| #24 | 'prostaglandin e1alpha':ab,ti,kw |
| #25 | 'pge1':ab,ti,kw |
| #26 | 'lipo-pge1':ab,ti,kw |
| #27 | 'lipo pge1':ab,ti,kw |
| #28 | 'prostaglandin e1':ab,ti,kw |
| #29 | 'caverject':ab,ti,kw |
| #30 | 'edex':ab,ti,kw |
| #31 | 'prostavasin':ab,ti,kw |
| #32 | 'muse':ab,ti,kw |
| #33 | 'viridal':ab,ti,kw |
| #34 | 'vasaprostan':ab,ti,kw |

| | |
|---|---|
| #35 | 'minprog':ab,ti,kw |
| #36 | 'sugiran':ab,ti,kw |
| #37 | 'prostin vr':ab,ti,kw |
| #38 | 'prostine vr':ab,ti,kw |
| #39 | #21 OR #22 OR #23 OR #24 OR #25 OR #26 OR #27 OR #28 OR #29 OR #30 OR #31 OR #32 OR #33 OR #34 OR #35 OR #36 OR #37 OR #38 |
| #40 | #19 AND #20 AND #39 |

**Table S5. Search Strategy: Cochrane Library**

| | |
|---|---|
| #1 | MeSH descriptor: [Diabetic Nephropathies] explode all trees |
| #2 | (Nephropathies, Diabetic):ti,ab,kw OR Nephropathy, Diabetic):ti,ab,kw OR (Diabetic Nephropathy):ti,ab,kw OR (Diabetic Kidney Disease):ti,ab,kw OR Diabetic Kidney Diseases):ti,ab,kw |
| #3 | (Kidney Disease, Diabetic):ti,ab,kw OR (Kidney Diseases, Diabetic):ti,ab,kw OR (Diabetic Glomerulosclerosis):ti,ab,kw OR (Glomerulosclerosis, Diabetic):ti,ab,kw OR (Intracapillary Glomerulosclerosis):ti,ab,kw |
| #4 | (Nodular Glomerulosclerosis):ti,ab,kw OR (Glomerulosclerosis, Nodular):ti,ab,kw OR (Kimmelstiel-Wilson Syndrome):ti,ab,kw OR (Kimmelstiel Wilson Syndrome):ti,ab,kw OR (Syndrome, Kimmelstiel-Wilson):ti,ab,kw |
| #5 | (Kimmelstiel-Wilson Disease):ti,ab,kw OR (Kimmelstiel Wilson Disease):ti,ab,kw |
| #6 | #1 OR #2 OR #3 OR #4 OR #5 |
| #7 | (injection*):ti,ab,kw |
| #8 | MeSH descriptor: [Alprostadil] explode all trees |

| | |
|---|---|
| #9 | (PGE1alpha):ti,ab,kw OR (Prostaglandin E1alpha):ti,ab,kw OR (PGE1):ti,ab,kw OR (Lipo-PGE1):ti,ab,kw OR (Lipo PGE1):ti,ab,kw |
| #10 | (Prostaglandin E1):ti,ab,kw OR (Caverject):ti,ab,kw OR (Edex):ti,ab,kw OR (Prostavasin):ti,ab,kw OR (Muse):ti,ab,kw |
| #11 | (Viridal):ti,ab,kw OR (Vasaprostan):ti,ab,kw OR (Minprog):ti,ab,kw OR (Sugiran):ti,ab,kw OR (Prostin VR):ti,ab,kw |
| #12 | (Prostine VR):ti,ab,kw |
| #13 | #8 OR #9 OR #10 OR #11 OR #12 |
| #14 | #6 AND #7 AND #13 |

**Table S6. Search Strategy: Web of Science**

| | |
|---|---|
| #1 | ((((TS=(Diabetic Nephropathies)) OR TS=(Nephropathies, Diabetic)) OR TS=(Nephropathy, Diabetic)) OR TS=(Diabetic Nephropathy)) OR TS=(Diabetic Kidney Disease) OR TS=(Diabetic Kidney Diseases) OR TS=(Kidney Disease, Diabetic) OR TS=(Kidney Diseases, Diabetic) OR TS=(Diabetic Glomerulosclerosis) OR TS=(Glomerulosclerosis, Diabetic) OR TS=(Intracapillary Glomerulosclerosis) OR TS=(Nodular Glomerulosclerosis) OR TS=(Glomerulosclerosis, Nodular) OR TS=(Kimmelstiel-Wilson Syndrome) OR TS=(Kimmelstiel Wilson Syndrome) OR TS=(Syndrome, Kimmelstiel-Wilson) OR TS=(Kimmelstiel-Wilson Disease) OR TS=(Kimmelstiel Wilson Disease) |
| #2 | ((((TS=(Alprostadil)) OR TS=(PGE1alpha)) OR TS=(Prostaglandin E1alpha)) OR TS=(PGE1)) OR TS=(Lipo-PGE1) OR TS=(Lipo PGE1) OR TS=(Prostaglandin E1) OR TS=(Caverject) OR TS=(Edex) OR TS=(Prostavasin) OR TS=(Muse) OR TS=(Viridal) OR TS=(Vasaprostan) OR TS=(Minprog) OR TS=(Sugiran) OR TS=(Prostin VR) OR TS=(Prostine VR) |
| #3 | TS=(injection*) |
| #4 | #1 AND #2 AND #3 |

**Table S7. Search Strategy: the Chinese Scientific Journal database (VIP)**

| | |
|---|---|
| #1 | 任意字段=糖尿病肾病+糖尿病性肾小球硬化症+糖尿病性肾病 |
| #2 | 任意字段= 注射液+注射剂+注射 |
| #3 | 任意字段=前列地尔 |
| #4 | #1 AND #2 AND #3 |

**Table S8. Search Strategy: Wanfang database**

| | |
|---|---|
| #1 | 检索表达式（中英文扩展&主题词扩展）： 全部:(糖尿病肾病 + 糖尿病性肾小球硬化症 + 糖尿病性肾病) and 全部:(注射液 + 注射剂 + 注射) and 全部:(前列地尔) |

**Table S9. Search Strategy: China National Knowledge Infrastructure (CNKI)**

| | |
|---|---|
| #1 | (篇关摘=糖尿病肾病 + 糖尿病性肾小球硬化症 + 糖尿病性肾病) AND (篇关摘=注射液 + 注射剂 + 注射) AND (篇关摘=前列地尔) |

**Table S10. Search Strategy: SinoMed**

| | |
|---|---|
| #1 | ("注射液"[全部字段:智能] OR "注射剂"[全部字段:智能] OR "注射"[全部字段:智能]) AND ("糖尿病肾病"[全部字段:智能] OR "糖尿病性肾病"[全部字段:智能] OR "糖尿病性肾小球硬化症"[全部字段:智能]) AND ("前列地尔"[全部字段:智能]) |

## *Supplementary table S11: Summary of All Included Studies*

| Study | classification of DKD | duration of DM | | Sample size | | Sex(M/F) | | Age (mean or range) | | Interventions | | Course of treatment | Outcomes |
|---|---|---|---|---|---|---|---|---|---|---|---|---|---|
| | | T | C | T | C | T | C | T | C | T | C | | |
| Zhoufang min(2012) | NA | NA | NA | 60 | 60 | 37/23 | 35/25 | 60.2±9.4 | 58.6±8.7 | PGE1 (20 μ g/d) + SMI(20ml/d) | PGE1 (20 μ g/d) | 4w | ②③④⑤ |
| Hanpeng(2013) | III-IV | 8.25±0.44 | | 40 | 40 | 44/36 | | 55.92±11.40 | | PGE1 (10 μ g/d) + SMI(1ml/d) | PGE1 (10 μ g/d) | 15d | ① |
| Wangqun(2014) | III | NA | NA | 16 | 18 | 32/21a | | 56.3±16.8 | | PGE1 (20 μ g/d) + SMI (20ml/d) | PGE1 (20 μ g/d) | 14d | ②③⑤ |
| Caiwenting(2015) | NA | 10.7±3.4 | 9.7±3.4 | 40 | 40 | 24/16 | 22/18 | 58±9.7 | 53.6±4.7 | PGE1 (20 μ g/d) + SMI (20ml/d) | PGE1 (20 μ g/d) | 4w | ②③④⑤ |
| Hemingwu(2015) | NA | 6-13 | 7-14 | 40 | 40 | 21/19 | 18/22 | 53.2±7.6 | 51.7±8.4 | PGE1 (20 μ g/d) + SMI (20ml/d) | PGE1 (20 μ g/d) | 4w | ① |
| Liugang(2015) | early stage | NA | NA | 52 | 52 | 28/24 | 30/22 | 59.33±5.16 | 60.25±4.94 | PGE1 (20 μ g/d) + SMI (20ml/d) | PGE1 (20 μ g/d) | 1m | ①②③④ |
| Liuying(2017) | early stage | NA | NA | 29 | 29 | 14/15 | 15/14 | 58.65±6.23 | 59.36±6.54 | PGE1 (20 μ g/d) + SMI (20ml/d) | PGE1 (20 μ g/d) | 1m | ① |

| | | | | | | | | | | | | | |
|---|---|---|---|---|---|---|---|---|---|---|---|---|---|
| Zhangyarong(2017) | early stage | NA | NA | 43 | 43 | 51/35 | | 48.21±4.67 | | PGE1 (20 μ g/d) + SMI (20ml/d) | PGE1 (20 μ g/d) | 4w | ①③ |
| Zhongchao(2017) | III | 9.2±1.1 | 9.3±1.2 | 47 | 47 | 28/19 | 31/16 | 62.21±2.21 | 63.34±2.18 | PGE1 (10 μ g/d) + SMI (20ml/d) | PGE1 (10 μ g/d) | 4w | ① |
| Chenxuan (2018) | III | NA | NA | 49 | 49 | 29/20 | 27/22 | 61.5±2.0 | 60.2±4.5 | PGE1 (10 μ g/d) + SMI (20ml/d) | PGE1 (10 μ g/d) | 4w | ②③④ |
| Lianglian g(2018) | NA | NA | NA | 48 | 48 | 21/27 | 22/26 | 55.7±3.3 | 55.9±2.5 | PGE1 (20 μ g/d) + SMI (20ml/d) | PGE1 (20 μ g/d) | 4w | ① |
| Wanganwen(2018) | I-III | 8.38±4.22 | 8.38±4.22 | 38 | 38 | 22/16 | 23/15 | 63.28±8.45 | 63.28±8.45 | PGE1 (20 μ g/d) + SMI (20ml/d) | PGE1 (20 μ g/d) | 4w | ②③ |
| Yangxu(2015) | early stage | 9.8±1.9 | 9.0±1.7 | 20 | 20 | 12/8 | 10/10 | 9.8±1.9 | 9.0±1.7 | PGE1 (10 μ g/d) + DCI(10ml/d) | PGE1 (10 μ g/d) | 2w | ②⑥ |
| Zhangyin(2016) | NA | 3.5±1.4 | 4.2±3.1 | 42 | 42 | 27/15 | 24/18 | 56.7±0.8 | 56.5±0.8 | PGE1 (5 μ g/d) + DCI(5ml/d) | PGE1 (5 μ g/d) | 4w | ①②③ |
| Cuiyi(2017) | early stage | NA | NA | 51 | 51 | 27/24 | 26/25 | 61.5±14.2 | 60.3±14.7 | PGE1 (10 μ g/d) + DCI(10ml/d) | PGE1 (10 μ g/d) | 1m | ②③ |
| Maigaoyang(2017) | III | 10.2±4.0 | 10.6±4.2 | 35 | 35 | 24/11 | 22/13 | 59.1±9.3 | 58.2±8.9 | PGE1 (20 μ g/d) + DCI(20ml/d) | PGE1 (20 μ g/d) | 14d | ②③④⑤ |
| Shenjinsong(2018) | early stage | 10.3±3.9 | 10.2±3.6 | 20 | 20 | 11/9 | 9/11 | 58.1±9.1 | 57.9±8.7 | PGE1 (20 μ g/d) + DCI(20ml/d) | PGE1 (20 μ g/d) | 4w | ①②⑥ |
| Subaoting (2018) | early stage | 5.21±1.08 | 5.30±1.01 | 44 | 44 | 26/18 | 24/20 | 60.42±5.75 | 60.29±5.90 | PGE1 (10 μ g/d) + DCI(10ml/d) | PGE1 (10 μ g/d) | 4w | ②③④ |
| Wangjing(2021) | early stage | 11.47±1.66 | 11.65±1.72 | 65 | 65 | 34/31 | 33/32 | 64.14±3.12 | 64.58±3.17 | PGE1 (10 μ g/d) + DCI(10ml/d) | PGE1 (10 μ g/d) | 3w | ②③④⑦ |
| Fanmin(2010) | III | NA | NA | 92 | 67 | 44/48 | 36/31 | 56-91 | 57-85 | PGE1 (10 μ g/d) + DHI(30-50ml/d) | PGE1 (10 μ g/d) | 2w | ②③④⑤ |
| Puhongmei(2013) | III | NA | NA | 26 | 26 | NA | NA | NA | NA | PGE1 (10 μ g/d) + DHI(20ml/d) | PGE1 (10 μ g/d) | 3m | ②④⑤⑦ |

| | | | | | | | | | | | | | |
|---|---|---|---|---|---|---|---|---|---|---|---|---|---|
| Liqiuxia(2015) | III | 11.2±2.3 | 10.4±2.6 | 24 | 24 | 12/12 | 13/11 | 55.7±7.5 | 56.8±7.2 | PGE1 (10 μ g/d) + DHI(40ml/d) | PGE1 (10 μ g/d) | 2w | ①④⑥ |
| Liuqingyuan(2016) | III | 13.21±8.25 | 11.35±9.18 | 24 | 21 | 11/13 | 10/11 | 52.10±11.86 | 52.50±12.16 | PGE1 (10 μ g/d) + DHI(20ml/d) | PGE1 (10 μ g/d) | 2w | ②③④⑤⑦ |
| Liuzhen(2016) | III-IV | 5.65±1.90 | 5.54±1.85 | 15 | 15 | 9/6 | 8/7 | 59.94±5.21 | 60.24±5.13 | PGE1 (10 μ g/d) + DHI(20-40ml/d) | PGE1 (10 μ g/d) | 3w | ①②③④⑤ |
| Jiayinji(2018) | IV | 8.8±1.3 | 7.7±1.5 | 35 | 36 | 18/17 | 18/18 | 62.7±2.4 | 65.2±2.1 | PGE1 (20 μ g/d) + DHI(30ml/d) | PGE1 (20 μ g/d) | 4w | ①②⑥ |
| Linchenxin(2019) | NA | 14.62±3.05 | 14.58±3.10 | 32 | 32 | 17/15 | 19/13 | 82.09±4.42 | 82.11±4.23 | PGE1 (10 μ g/d) + DHI(20ml/d) | PGE1 (10 μ g/d) | 2w | ①④⑤⑥⑦ |
| Xiaoqianfeng(2019) | NA | 7.2±2.1 | 7.1±1.8 | 40 | 40 | 22/18 | 24/16 | 58.3±7.6 | 60.8±8.2 | PGE1 (10 μ g/d) + DHI(20-40ml/d) | PGE1 (10 μ g/d) | 3w | ①②③④⑦ |
| Xiebinxuan(2002) | NA | 3.52 | 3.48 | 20 | 10 | 12/8 | 7/3 | 64.32±6.94 | 63.47±7.11 | PGE1 (100 μ g/d) + HQI (40ml/d) | PGE1 (100 μ g/d) | 3w | ③⑥⑦ |
| Zhaolijun(2010) | early stage | 10±3 | 8±3 | 169 | 157 | 82/87 | 84/73 | 55±12 | 57±10 | PGE1 (100ml/d) + HQI (60ml/d) | PGE1 (100ml/d) | 2w | ⑥⑦ |
| Wuyanbo(2011) | III | NA | NA | 40 | 40 | 22/18 | 23/17 | 55.3±6.9 | 56.9±7.1 | PGE1 (10 μ g/d) + HQI (30ml/d) | PGE1 (10 μ g/d) | 14d | ②③④ |
| Linjixiang(2013) | IV | NA | NA | 45 | 45 | 22/23 | 24/21 | 64.1±11.2 | 63.4±10.6 | PGE1 (20 μ g/d) + HQI (15ml/d) | PGE1 (20 μ g/d) | 15d | ①②⑥ |
| Liusiyan(2013) | NA | 2-13 | 2-11 | 16 | 17 | 10/6 | 11/6 | 44.6±7.1 | 44.5±7.2 | PGE1 (10 μ g/d) + HQI (30ml/d) | PGE1 (10 μ g/d) | 2w | ①②③④⑦ |
| Zhoubin(2014) | NA | NA | NA | 30 | 30 | 15/15 | 16/14 | 41.2±11.3 | 43.1±9.8 | PGE1 (20 μ g/d) + HQI (20ml/d) | PGE1 (20 μ g/d) | 4w | ①②③⑥ |
| Fangwenjuan(2016) | NA | 12.1±2.3 | | 40 | 40 | 47/33 | | 61.9±3.6 | | PGE1 (10 μ g/d) + HQI (30ml/d) | PGE1 (10 μ g/d) | 2w | ①④⑥ |
| Zhangliang(2016) | NA | 2-8 | 3-8 | 45 | 45 | 28/20 | 27/18 | 46-68 | 45-70 | PGE1 (10 μ g/d) + HQI (30ml/d) | PGE1 (10 μ g/d) | 4w | ①②③④⑤⑦ |

| | | | | | | | | | | | | | |
|---|---|---|---|---|---|---|---|---|---|---|---|---|---|
| Xufei(2018) | III | 9.8±2.7 | 10.2 ±2.8 | 43 | 43 | 26/17 | 27/16 | 58.4±8.5 | 59.2±9.1 | PGE1 (20 μ g/d) + HQI (30ml/d) | PGE1 (20 μ g/d) | 4w | ②③④⑤⑦ |
| Wanghuibin(2009) | III | NA | NA | 28 | 16 | 14/14 | 7/9 | 47±7 | 45±10 | PGE1 (10 μ g/d) + SKI(100ml/d) | PGE1 (10 μ g/d) | 14d | ②③⑥⑦ |
| Pangjialiang(2011) | III | 8.1±4.6 | 8.3± 5.1 | 30 | 30 | 16/14 | 17/13 | 53.5±7.3 | 52.2±6.8 | PGE1 (10 μ g/d) + SKI(60ml/d) | PGE1 (10 μ g/d) | 2w | ⑦ |
| Zhanghong(2013) | NA | 3.45±0.76 | | 30 | 30 | 14/16 | 17/13 | 53.73 | 52.41 | PGE1 (10 μ g/d) + SKI(100ml/d) | PGE1 (10 μ g/d) | 28d | ②③⑥ |
| Dingxuemei(2014) | III-IV | NA | NA | 36 | 34 | NA | NA | NA | NA | PGE1 (10 μ g/d) + SKI(100ml/d) | PGE1 (10 μ g/d) | 28d | ②③⑦ |
| Lanchunying(2014) | III-IV | 10.0±3.0 | | 40 | 40 | 19/21 | 22/18 | 55.0±6.0 | | PGE1 (10 μ g/d) + SKI(60ml/d) | PGE1 (10 μ g/d) | 4w | ①②③⑥ |
| Meidongdong(2014) | II-IV | 10.1± 2.5 | 10.4 ±2.7 | 20 | 20 | 12/8 | 12/8 | 60.9±8.7 | 61.6±7.6 | PGE1 (10 μ g/d) + SKI(60ml/d) | PGE1 (10 μ g/d) | 14d | ②③⑥ |
| Yinlili(2014) | NA | 7.1±0.5 | | 44 | 44 | NA | NA | 50.43±6.90 | | PGE1 + SKI(100ml/d) | PGE1 | NA | ① |
| Zhangbailing(2014) | IV | 13.9±4.3 | | 35 | 35 | 38/32 | | 54.5±8.3 | | PGE1 (10 μ g/d) + SKI(100ml/d) | PGE1 (10 μ g/d) | 4w | ②③④⑥ |
| Zhangyunpin(2017) | NA | 7.6±2.3 | 7.5± 2.1 | 58 | 58 | 26/22 | 28/20 | 64.8±8.3 | 64.7±9.5 | PGE1 (10 μ g/d) + SKI(100ml/d) | PGE1 (10 μ g/d) | 28d | ①②③ |
| Quanye(2018) | NA | 8.6±2.4 | 8.1± 2.3 | 30 | 30 | 18/12 | 17/13 | 64.8±8.7 | 64.4±8.5 | PGE1 (10 μ g/d) + SKI(60ml/d) | PGE1 (10 μ g/d) | 2w | ①②③⑥ |
| Zhengwenwu(2018) | early stage | 4.35± 1.26 | 4.61 ± 1.18 | 30 | 30 | 16/14 | 17/13 | 58.37± 4.49 | 58.19± 4.21 | PGE1 (10 μ g/d) + SKI(100ml/d) | PGE1 (10 μ g/d) | 4w | ②③ |
| Wangxiaojun(2019) | NA | 7.5±1.2 | 7.7± 1.3 | 50 | 50 | 31/19 | 32/18 | 65.1±3.1 | 65.3±3.2 | PGE1 (10 μ g/d) + SKI(100ml/d) | PGE1 (10 μ g/d) | 30d | ① |
| Zhangmailang(2019) | III-IV | NA | NA | 48 | 48 | 25/23 | 26/22 | 54.26± 13.85 | 53.79± 13.42 | PGE1 (10 μ g/d) + SKI(60ml/d) | PGE1 (10 μ g/d) | 30d | ①②③⑥ |

| | | | | | | | | | | | | | |
|---|---|---|---|---|---|---|---|---|---|---|---|---|---|
| Wenxusheng(2021) | IV | 9.69±2.26 | 10.23±2.13 | 36 | 36 | 25/17 | 23/13 | 51.46±3.37 | 52.37±3.11 | PGE1 (10 μ g/d) + SKI(60ml/d) | PGE1 (10 μ g/d) | 4w | ①②④⑥⑦ |
| Niexing(2022) | NA | NA | NA | 50 | 50 | 29/21 | 27/23 | 63.12±7.71 | 63.85±7.62 | PGE1 (10 μ g/d) + SKI(60ml/d) | PGE1 (10 μ g/d) | 28d | ②③⑥ |
| Rujianyong(2008) | III | 12±10 | 13±9 | 64 | 64 | 34/30 | 35/29 | 58±10 | 56±9 | PGE1 (10 μ g/d) + SXTI(6ml/d) | PGE1 (10 μ g/d) | 8w | ②③④⑤ |
| Gongyun(2009) | III | NA | NA | 36 | 30 | NA | NA | NA | NA | PGE1 (20 μ g/d) + SXTI(6ml/d) | PGE1 (20 μ g/d) | 15-20d | ③④⑦ |
| Xingyelan (2012) | III-IV | 6.2±2.6 | 5.7±2.8 | 60 | 60 | 38/22 | 47/13 | 66.4±9.7 | 65.2±9.8 | PGE1 (100 μ g/d) + SXTI(6ml/d) | PGE1 (100 μ g/d) | 4w | ②③⑤⑦ |
| Tiantian(2018) | III-IV | 5.71±0.72 | 5.68±0.70 | 57 | 57 | 34/23 | 37/20 | 56.24±6.12 | 55.78±7.32 | PGE1 (20 μ g/d) + SXTI(2ml/d) | PGE1 (20 μ g/d) | 2w | ①②③⑤ |
| Caoli(2016) | IV | 8.3±1.2 | 9.2±1.3 | 33 | 31 | 18/15 | 17/14 | 54.2±9.86 | 53.9±12.5 | PGE1 (2ml/d) +XBJI (10ml/d） | PGE1 (2ml/d) | 14d | ②③④⑤⑥ |
| Liqing(2016) | NA | 5.9±2.6 | 5.6±2.7 | 30 | 30 | 17/13 | 13/17 | 65.6±7.3 | 63.1±9.5 | PGE1 (10 μ g/d) + XBJI (40ml/d) | PGE1 (10 μ g/d) | 7d | ②③⑥⑦ |
| Jiangqiang(2017) | NA | 6.02±2.20 | 6.11±2.44 | 78 | 78 | 48/30 | 44/34 | 59.54±10.13 | 60.52±10.34 | PGE1 (20 μ g/d) + XBJI (50ml/d) | PGE1 (20 μ g/d) | 2w | ②③④⑥ |

① Total effective rate，② Serum Creatinine，③ Blood Urea Nitrogen, ④ Urinary Albumin excretion rates, ⑤ Urinary beta 2-microglobulin，⑥ 24h Urine Albumin, ⑦ Adverse reactions, SMI: Salvia miltiorrhiza injection, DCI: Danshen-Chuanxiongqin injection, DHI: Danhong injection, HQI: Huangqi injection, SKI: Shenkang injection, SXTI: Shuxuetong injection, XBJI: Xuebijing injection. PGE1: alprostadil injection

*Supplementary table S12: Extract and extraction process description of TCMIs*

| Injection | Extracts and Extraction Process Description |
|---|---|
| **Danshen Injection** | 1500 g *Salvia miltiorrhiza Bunge* was decocted three times with water, the first 2 hours, and the second and third 1.5 hours each. And the decoction was combined, filtered, and the filtrate was concentrated to 750 mL under reduced pressure. Add ethanol to precipitate twice, using 75% ethanol for the first time and 85% ethanol for the second time, and filter after refrigeration each time. Then recover the ethanol from the filtrate, and concentrate to about 250 mL. After that, add injection water to 400ml, mix well, and then refrigerate. Then filter, adjust the pH value to 6.8 with 10% sodium hydroxide solution, and boil for half an hour. And filter again, add injection water to 1000 mL, fill in a container and seal, sterilize, and the Danshen injection was ready. |
| **Danshen-Chuanxiongqin injection** | Take the pre-processed formula amount of Salvia miltiorrhiza Bunge ligustrazine, and add an appropriate amount of water to soak. They were decocted and extracted three times with water and filtered. Filtrates were combined, and clarifiers were added while stirring during the filtrate concentration process. And then, they were left to centrifuge with a high-speed centrifuge. Collect the supernatant, concentrate the supernatant, add ethanol to achieve an alcohol content of 70-80%, and refrigerate. Filter on filter paper and add ethanol to the filtrate to achieve an alcohol content of 80% and refrigerate. After paper pulp filtration, the filtrate was refluxed in an extractor to recover ethanol and concentrate filtrate, followed by adding purified water for refrigeration. After paper pulp filtration again, add sodium hydroxide solution to the filtrate, adjust pH to 6.5- 8.5, and allow static settlement. Then pulp filter again, and the filtrate was adjusted to pH 3.0 with hydrochloric acid solution and allowed to static settlement. Once more pulp filtration, then filtered with a microporous membrane, and finally ultrafiltered with an ultrafilter to obtain Salvia miltiorrhiza extract, which was then added with formulated amounts of Tetramethylpyrazine drug substance and glycerol. Stir thoroughly, adjust to the appropriate volume and pH 3.0-4.0, and the qualified intermediate product was filled in ampoules and sterilized. |
| **Danhong Injection** | Mix 250 g *Carthamus tinctorius L.*, 750 g *Salvia miltiorrhiza Bunge*, and 7 g Sodium Chloride for Injection to make a 1000 mL solution. Before that, *Salvia miltiorrhiza Bunge* was soaked twice with dilute ethanol for 1 hour each time, filtered, and the filtrate was stored for future use; the residue was mixed with safflower, soaked twice with water for 1 hour each time, filtered, and |

| | |
|---|---|
| | the filtrate was combined and concentrated to an ointment with a relative density of 1.10 to 1.20 (65 °C), sodium chloride for injection was added to reach isotonicity, pH was adjusted to 6 to 7, filtered, and refrigerated for 24 hours. Injection water was added to the specified amount. Then filter, fill in a container and seal, sterilize, and the Danhong injection was ready. |
| **Huangqi Injection** | 2000 g of *Astragalus mongholicus Bunge* was decocted with water three times for 1.5 hours each time. The decoction was combined, filtered, and the filtrate was concentrated to 1 mL equivalent to 1 ~ 2g of the original medicinal material. Then the solution was precipitated with ethanol twice. The ethanol content in the first solution was 75% and 85% in the second, and it was refrigerated each time. Ethanol was recovered and concentrated to 1 mL equivalent to 10g of the original medicinal material. Diluted with injection water to 1 mL equivalent to 0.75 ~ 1g of the original medicinal material, placed for 12 hours under refrigeration. Then filter, and concentrate the filtrate to 1 mL equivalent to 5 ~ 6g of the original medicinal material. Cool it down, and adjust pH to 7.5 with 20% sodium hydroxide solution. Boil the solution, add 0.125% activated carbon, then boil again for 5 minutes. Filter it while hot, add injection water to 1000ml, filter, then adjust pH to 7.5 with 20% sodium hydroxide solution. Filter again, fill in a container and seal, sterilize, and the Huangqi injection was ready. |
| **Shenkang Injection** | The prescription contains four herbs: *Astragalus mongholicus Bunge, Rheum palmatum L, Salvia miltiorrhiza Bunge, Carthamus tinctorius L.* Specification: 20mL/vial. 'Shenkang injection and its preparation technology' is an injection extracted from *Astragalus mongholicus Bunge, Rheum palmatum L, Salvia miltiorrhiza Bunge, Carthamus tinctorius L.*, and its formula contains 70-150 g *Rheum palmatum L*, 60-120 g *Salvia miltiorrhiza Bunge*, 150-280 g *Astragalus mongholicus Bunge*, and 80-150 g *Carthamus tinctorius L.*. In the preparation, *Rheum palmatum L* and *Salvia miltiorrhiza Bunge* were decocted according to the formula amount → primary alcohol precipitation → de-tanning → secondary alcohol precipitation → tertiary alcohol precipitation → dilution, and then *Astragalus mongholicus Bunge* and *Carthamus tinctorius L* were decocted according to the formula amount → primary alcohol precipitation → secondary alcohol precipitation → dilution, combined with the above two groups of diluents, and fine filtered→ sub-packaging → sterilization → quality inspection → packaging. |
| **Shuxuetong Injection** | *Pheretima* and *Hirudo* awere soaked in 10% normal saline at room temperature and ground to homogenate at low temperature on ice. After the homogenate was frozen for 12 h, the frozen homogenate was fully thawed at low temperature and then frozen again after thorough melting, and the above process was repeated. After repeated five times, the homogenate was centrifuged at 3000 r/min for 5 min, and the supernatant was removed and filtered with a filter column. Then the filtrate was desalted. Ethanol was added into the filtrate of the supernatant, followed by reflux extraction. Then the extract was centrifuged again, and ethanol in the filtrate |

| | |
|---|---|
| | was evaporated to dryness. After the filtrate was combined, it was concentrated under reduced pressure, dried, and sieved. Medicinal starch was added and mixed to make capsules. Make sure the amount of drug substance in each capsule at 0.5 g. |
| **Xuebijing Injection** | We did not find the specific preparation process of the drug, considering that it may be related to the confidentiality of the product |

## *Supplementary table S13: More details about the product information of 7 TCMI*

| Injection name | Source | Therapeutic claims in TCM | Indications | Adverse drug reactions | Quality control reported ? | Chemical analysis reported? |
|---|---|---|---|---|---|---|
| Danshen injection | Zhengda Qingchun Bao Pharmaceutical Co., Ltd. Chengdu Tiantaishan Pharmaceutical Co., Ltd. Shanghai Xinya Pharmaceutical Gaoyou Co., Ltd. Qiqihaer No.2 Pharmaceutical | Activating blood circulation and removing blood stasis, dredging pulse and nourishing heart. | Used for coronary heart disease, chest tightness, and angina pectoris. | 1. Allergic reactions: skin flushing or pallor, rash, itching, chills, laryngeal edema, dyspnea, palpitations, cyanosis, decreased blood pressure and even shock.<br>2. Skin and its appendages: rash (including erythema, papules, wheals, etc.), itching, sweating, local skin reactions, etc.<br>3. Systemic reactions: chills, chills, fever and even high fever, fatigue, body pain, pallor, edema, anaphylactic shock, etc.<br>4. Respiratory system: cough, throat discomfort, chest tightness, breath holding, breathing difficulties, etc.<br>5. Cardiovascular system: palpitations, chest tightness, breath holding, cyanosis, arrhythmia, increase or decrease in blood pressure, etc.<br>6. Digestive system: nausea, vomiting, abdominal pain, bloating, dry mouth, etc. | YES. Z33020177 issued by China Food and Drug Administration | NO. |

| | | | | | | |
|---|---|---|---|---|---|---|
| | Co., Ltd.<br>Sichuan Shenghe pharmaceutical co., Ltd.<br>Shanghai Zhongxi Pharmaceutical Co., Ltd. | | | 7. Mental and nervous system: dizziness, headache, convulsions, tremors, local or peripheral numbness, etc.<br>8. Medication site: flushing, pain, purpura, etc.<br>9. Others: visual abnormalities, facial discomfort, etc. | | |
| Danshen - Chuanxiongqin injection | Guizhou Beit Pharmaceutical Co., Ltd.<br>Jilin Sichang pharmaceutical co., Ltd. | / | For occlusive cerebrovascular diseases | 1. Allergic reactions: skin flushing or pallor, rash, itching, chills, laryngeal edema, dyspnea, palpitations, cyanosis, decreased blood pressure and even shock.<br>2. Systemic damage: chills, chills, fever (even high fever), pain, chest tightness, chest pain, fatigue, trembling, edema, pale face, etc.<br>3. Skin and its adnexal damage: rash, itching, hyperhidrosis.<br>4. Cardiovascular system damage: phlebitis, flushing, cyanosis, palpitations, arrhythmias, increased or decreased blood pressure, etc.<br>5. Nervous system damage: dizziness, headache, bloating, local and general numbness, convulsions, irritability, tremor, dizziness, insomnia, mental disorders, drowsiness, etc.<br>6. Digestive system damage: nausea, vomiting, abdominal pain, diarrhea, bloating, dry mouth.<br>Respiratory damage: dyspnea, shortness of breath, chest tightness, breath holding, cough, throat discomfort, etc.<br>7. Damage to the musculoskeletal system: myalgia, back pain, joint pain, etc.<br>8. Blood system damage: purpura, bleeding, etc.<br>9. Damage at the site of medication: pain at the injection site, lump at the injection site.<br>10. Others: visual abnormalities, tinnitus, hearing loss, etc. | YES. H22026448 issued by China Food and Drug Administration | NO |
| Danhong injection | Jinan Buchang Pharmaceutical Co., Ltd.<br>Shandong Danhong | Activating blood circulation and removing | For chest arthralgia and stroke | 1. Allergic reactions: facial flushing, rash, itching, urticaria, laryngeal edema, dyspnea, breath holding, palpitations, cyanosis, decreased blood pressure, anaphylactic shock, etc.<br>2. Systemic damage: chills, high fever, etc.<br>3. Cardiovascular system damage: chest tightness, palpitations, increased | YES. Z2016866 issued by China Food | NO. |

| | | | | | | |
|---|---|---|---|---|---|---|
| | Pharmaceutical Co., Ltd. | blood stasis | | blood pressure, etc.<br>4. Damage to the digestive system: nausea. Vomiting, abdominal pain, diarrhea, etc., there are cases of abnormal liver biochemical indicators.<br>5. Nervous system damage: dizziness, headache, convulsions, coma, etc.<br>6. Others: there are case reports of purpura, hematuria, nose, gum bleeding, conjunctival bleeding, gastrointestinal bleeding, subcutaneous bleeding spots and ecchymosis. | and Drug Adminis tration | |
| Huangqi injection | Produced by Shenwei Pharmaceutical Co., Ltd. Heilongjiang Zhenbaodao pharmaceutical co., Ltd | Nourish the qi and nourish the yuan, help the righteous to dispel evil, nourish the heart and pulse, and strengthen the spleen and dampness | Viral myocarditis of heart qi deficiency and blood stasis, cardiac insufficiency and hepatitis of spleen deficiency and dampness obstruction. | 1.Allergic reactions: common drug fever, drug eruption, injection site redness and swelling, rare acute allergic reactions, anaphylactic shock and other serious adverse reactions.<br>2. Respiratory system: common laryngeal edema, dyspnea, asthma, chest tightness.<br>3. Circulatory system: occasional hypotension delayed phlebitis; rare rapid atrial fibrillation.<br>4. Digestive system: even liver function damage, vomiting, diarrhea.<br>5. Others: occasional severe headache, renal function damage; rare hemolytic anemia; pyrogen reaction caused by intravenous drip. | YES. Z130209 99 issued by China Food and Drug Adminis tration | NO. |
| Shenkan g injection | Xi'an Century Shengkang Pharmaceutical Co., Ltd. Xi'an Jiahui Pharmaceutical Co., Ltd. | Descending adverse and purging turbid, invigorating qi and activating blood circulation, dredging viscera and promoting dampness | It is suitable for the syndrome of dampness and blood stasis in chronic renal failure. | 1. Systemic damage: chills, chills, fever, pain, fatigue, anaphylactoid reactions, etc.<br>2. Respiratory system: chest tightness, dyspnea, shortness of breath, cough, etc.<br>3. Skin and its appendages: rash, itching, sweating, skin redness, etc.<br>4. Digestive system: nausea, vomiting, abdominal pain, diarrhea, bloating, dry mouth, stomach discomfort, etc.<br>5. Cardiovascular system: palpitations, flushing, increased blood pressure, tachycardia, etc.<br>6. Medication site: pain at the injection site.<br>7. Nervous system: dizziness, headache.<br>8. Others: Phlebitis, etc. | Z200401 10 issued by China Food and Drug Adminis tration | NO |

| | | | | | | |
|---|---|---|---|---|---|---|
| Shuxuetong injection | Mudanjiang Youbao Pharmaceutical Co., Ltd. | Promoting blood circulation and removing blood stasis, dredging channels and activating collaterals. | It is used in the acute stage of meridian in ischemic stroke caused by blood stasis. | 1. Allergic reactions: systemic skin flushing, rash, itching, urticaria, laryngeal edema, dyspnea, breath holding, heart Palpitations, cyanosis, decreased blood pressure, anaphylactic shock, etc.<br>2. Systemic damage: chills, fever, hyperthermia, chills, fatigue, etc.<br>Respiratory system: chest tightness, dyspnea, shortness of breath, cough, breath holding, etc.<br>3. Cardiovascular system: palpitations, etc.<br>4. Digestive system: nausea, vomiting, abdominal pain, diarrhea, etc.<br>5. Nervous system: dizziness, headache, convulsions, etc.<br>6. Skin and its appendages: rash, urticaria, maculopapular rash, erythema, itching, sweating, etc.<br>7. Others: purpura, hematuria, gastrointestinal bleeding, conjunctival bleeding, subcutaneous bleeding, prothrombin time abnormality, etc.; There have been case studies of thrombocytopenia and fundus hemorrhage. | Z20010100 issued by China Food and Drug Administration | NO |
| Xuebijing injection | Tianjin Hongri Pharmaceutical Co., Ltd. | Detoxify stasis | Used for warm diseases, symptoms such as fever, wheezing, palpitations, irritability and other stasis mutual evidence: suitable for systemic inflammatory response syndrome induced by infection; It can also be combined with the treatment of | 1. Allergic reactions: skin flushing, rash, itching, dyspnea, palpitations, cyanosis, increased or decreased blood pressure Laryngeal edema, anaphylactic shock, etc.<br>2. Systemic damage: anaphylactic shock, chills, fever, pallor, fatigue, sweating, convulsions.<br>3. Skin damage: skin allergies, rashes, itching, skin flushing.<br>4. Cardiovascular system; Palpitations, cyanosis, increased or decreased blood pressure, arrhythmias.<br>5. Nervous system: dizziness, headache.<br>Respiratory system: dyspnea, chest tightness, breath holding, shortness of breath, cough, laryngeal edema.<br>6. Digestive system: nausea, vomiting, abdominal pain, diarrhea, abnormal liver function.<br>Urinary system: urinary frequency, urgency, dysuria, hematuria.<br>7. Others: facial edema, conjunctival hyperemia, lacrimation abnormalities, phlebitis, low back pain, back pain, local numbness. | Z20040033 issued by China Food and Drug Administration | NO |

| | | |
|---|---|---|
| | multi-organ dysfunction syndrome in the period of impaired organ function. | |

## *Supplementary table S14: Specific intervention methods of the study*

| The serial number | Intervention | |
|---|---|---|
| | The intervention group | The control group |
| Zhoufangmin(2012) | On the basis of the control group, SMI 20 ml was added. | Routine treatment: low-salt, low-protein diabetic diet, insulin or oral hypoglycemic therapy, ACEI/ARB therapy is preferred in patients with hypertension, and statin or fibrate lipid modulators are given in patients with disorders of lipid metabolism.<br>On this basis, PGE1 20 μg was given. |
| Hanpeng(2013) | On the basis of control group, SMI 1ml was injected at Zusanli acupoint. | Routine treatment: reasonable diet, diabetes education.<br>On this basis, PGE1 10 mg was given. |
| Wangqun(2014) | On the basis of the control group, SMI 20 ml was added. | Routine treatment: diabetic diet, insulin or oral hypoglycemic drugs to control blood glucose; ACEI drugs to control blood pressure in patients with hypertension; statins to treat hyperlipidemia.<br>On this basis, PGE1 20 ug was given. |
| Caiwenting(2015) | On the basis of the control group, SMI 20 ml was added. | Routine treatment: Control blood pressure, blood glucose and blood lipids.<br>On this basis, PGE1 20 ug was given. |
| Hemingwu(2015) | On the basis of the control group, SMI 20 ml was added. | Routine treatment: diet control, hypoglycemia, blood pressure, lipid control, anemia correction.<br>On this basis, PGE1 20 ug was given. |
| Liugang(2015) | On the basis of the control group, SMI 20 ml was added. | Routine treatment: diet was amainly low protein and low salt, and oral hypoglycemic drugs and insulin injections were used to control blood glucose. Meanwhile, comorbidities such as hypertension and lipid metabolism disorders were actively treated.<br>On this basis, PGE1 20 ug was given. |
| Liuying(2017) | On the basis of the control group, SMI 20 ml was added. | Routine treatment: all patients were given low-salt, low-protein food, insulin injection and oral hypoglycemic drugs to control blood glucose.<br>On this basis, PGE1 20 ug was given. |

| | | |
|---|---|---|
| Zhangyarong(2017) | On the basis of the control group, SMI 20 ml was added. | Routine treatment: lowering glucose, controlling diet, adjusting blood lipids and lowering blood pressure.<br>On this basis, PGE1 20 ug was given. |
| Zhongchao(2017) | On the basis of the control group, SMI 20 ml was added. | Routine treatment: including dietary intervention, eating low salt, low protein diet; The blood glucose and blood pressure of patients were controlled, and blood lipids were regulated. Insulin and oral antidiabetic drugs were used for hypoglycemia.<br>On this basis, PGE1 10 ug was given. |
| Chenxuan(2018) | On the basis of the control group, SMI 20 ml was added. | Routine treatment: low-salt and low-protein diet, oral hypoglycemic drugs or insulin injection to control blood glucose, and ACEI/ARB treatment for patients with hypertension.<br>On this basis, PGE1 10 ug was given. |
| Liangliang(2018) | On the basis of the control group, SMI 20 ml was added. | Routine treatment: low salt and low protein diet, oral hypoglycemic drugs or insulin injection to control blood glucose, blood pressure and blood lipids.<br>On this basis, PGE1 20 ug was given |
| Wanganwen(2018) | On the basis of the control group, SMI 20 ml was added. | Routine treatment: low-salt and low-protein diabetic diet, and medication for lowering glucose, regulating lipid, controlling blood pressure, etc.<br>On this basis, PGE1 20 ug was given. |
| Yangxu(2015) | On the basis of the control group, DCI 10 ml was added. | Routine treatment: diabetic diet, proper exercise, blood glucose control.<br>on this basis, PGE1 10 ug was given. |
| Zhangyin(2016) | On the basis of the control group, DCI 10 ml was added. | Routine treatment: including insulin injection, diet therapy and exercise therapy, etc., to control patients' blood glucose at a stable level.<br>On this basis, PGE1 5 ug was given. |
| Cuiyi(2017) | On the basis of the control group, DCI 10 ml was added. | PGE1 10 ug was given. |
| Maigaoyang(2017) | On the basis of the control group, DCI 20 ml was added. | Routine treatment: including dietary guidance (low-salt, low-protein, low-glucose diet), oral hypoglycemic drugs or insulin injection to control blood glucose level, try to control the fasting blood glucose to 4-6mmol /L, 2h postprandial blood glucose to 4-8mmol /L.<br>ACEI/ARB was used to control blood pressure in patients with obvious hypertension or severe hypertension, keeping diastolic blood pressure ≤80mmHg and systolic blood pressure ≤130mmHg.<br>Patients with lipid metabolism can be treated with statins or bettes for lipid regulation.<br>On this basis, PGE1 20 ug was given. |
| Shenjinsong(2018) | On the basis of the control group, DCI 20 ml was added. | Routine treatment: lipid-lowering, diet control, blood pressure, glucose, and other therapeutic measures.<br>On this basis, PGE1 20 ug was given. |
| Subaoting(2018) | On the basis of the control group, DCI 20 ml was added. | Routine treatment: insulin injection, antihypertensive, antiplatelet aggregation and other basic treatment, and strict implementation of diabetic diet, regular exercise.<br>On this basis, PGE1 10 ug was given. |
| Wangjing(2021) | On the basis of the control group, DCI 10 ml was added. | Routine treatment: all patients maintained a healthy diet and oral hypoglycemic drugs. Obese or insulin-resistant patients took insulin to control blood glucose and kept fasting blood glucose at 5-7mmol/L.<br>On this basis, PGE1 10 ug was given. |

| | | |
|---|---|---|
| Fanmin(2010) | On the basis of the control group, DHI 30-50 ml was added. | Routine treatment: Diabetic diet, oral hypoglycemic drugs and/or insulin to control blood glucose.<br>On this basis, PGE1 10 ug was given. |
| Puhongmei(2013) | On the basis of the control group, DHI 20 ml was added. | Routine treatment: including diabetes education, low salt, low phosphorus, low protein and diabetic diet, blood pressure control, insulin control of blood glucose, fasting blood glucose control in 3.9-6.7mmol/L, 2 hours postprandial blood glucose control in 7.2-10.0mmol/L.<br>On this basis, PGE1 10 ug was given. |
| Liqiuxia(2015) | On the basis of the control group, DHI 40 ml was added. | Routine treatment: all patients were given basic diabetes knowledge education, guidance on rational diet, appropriate exercise, blood glucose monitoring, and combined with oral hypoglycemic drugs and/or insulin and other basic treatment.<br>On this basis, PGE1 10 ug was give. |
| Liuqingyuan(2016) | On the basis of the control group, DHI 20 ml was added. | Routine treatment: diabetic high quality low protein diet, insulin subcutaneous injection to control blood glucose, ACEI/ARB to control blood pressure, oral administration of compound alpha keto acid.<br>On this basis, PGE1 10 ug was given. |
| Liuzhen(2016) | On the basis of the control group, DHI 20-40 ml was added. | Routine treatment: (1) Blood glucose control: insulin injection or oral hypoglycemic drug treatment, so that fasting blood glucose < 7mmol/L, 2h postprandial blood glucose < 10mmol/L, glycated hemoglobin control ≤7%; (2) Blood pressure control: oral routine antihypertensive drugs to control blood pressure < 130/80 mmHg; (3) Control blood lipid: oral simvastatin and other drugs treatment, the total cholesterol < 4.5mmol/L, triglyceride < 1.5mmol/L, High-Density Lipoprotein Cholesterol > 1.1mmol/L, Low-Density Lipoprotein Cholesterol < 2.5mmol/L; (4) All patients were given diabetes knowledge education, reasonable diet, exercise guidance, etc.<br>On this basis, PGE1 10 ug was given. |
| Jiayinji(2018) | On the basis of the control group, DHI 30 ml was added. | Routine treatment: diabetes education and diet control, insulin and lipid-lowering (atorvastatin 20mg once a day), aspirin 100mg once a day and Irbesartan 150mg once a day as the basic treatment. Fasting blood glucose was controlled below 7.0mmol/L, and random blood glucose was controlled at 11.0mmol/L. Keep blood pressure under 130/80 mmHg.<br>On this basis, PGE1 20 ug was given. |
| Linchenxin(2019) | On the basis of the control group, DHI 20 ml was added. | Routine treatment: strict diet control, moderate exercise, injection of insulin or oral hypoglycemic drugs to control blood glucose, control blood pressure and regulate blood lipids.<br>On this basis, PGE1 10 ug was given static drops. |
| Xiaoqianfeng(2019) | On the basis of the control group, DHI 20-40 ml was added. | Routine treatment: control of blood glucose, blood pressure and lowering of blood lipids were given, and diabetes knowledge education, and reasonable diet and exercise guidance were given.<br>On this basis, PGE1 10 ug was given. |
| Xiebinxuan(2002) | On the basis of the control group, HQI 40 ml was added. | Routine treatment: routine diabetic diet, hypoglycemic drugs should be used to control fasting blood glucose at 6-7mmol/L, and hypoglycemic drugs should be used to stabilize blood pressure at 18/11kpa for patients with hypertension.<br>On this basis, PGE1 100 ug was given. |
| Zhaolijun(2010) | On the basis of the control group, HQI 60 ml was added. | Routine treatment: diet control, dietary protein intake limited to 0.8g/ (kg·d), hypoglycemic and antihypertensive treatment, so that fasting blood glucose < 6.1mmol/L, glycosylated hemoglobin < 7%, blood pressure < 130/80mmHg.<br>On this basis, PGE1 10 ug was given. |

| | | |
|---|---|---|
| Wuyanbo(2011) | On the basis of the control group, HQI 30 ml was added. | Routine treatment: all patients were given a high-quality low protein, low salt diet to control blood pressure and blood glucose.<br>On this basis, PGE1 10 ug was given. |
| Linjixiang(2013) | On the basis of the control group, HQI 15 ml was added. | Routine treatment: diet therapy, diabetes health education, etc.<br>On this basis, PGE1 20 ug was given. |
| Liusiyan(2013) | On the basis of the control group, HQI 30 ml was added. | Routine treatment: Regular treatment such as oral hypoglycemic drugs or subcutaneous insulin injection can control the blood glucose level of patients.<br>On this basis, PGE1 20 ug was given. |
| Zhoubin(2014) | On the basis of the control group, HQI 20 ml was added. | Routine treatment: all patients were given high-quality low protein diabetic diet, while controlling blood glucose, blood pressure, diuresis and detumescence of edema patients and other conventional treatments; Oral hypoglycemic drugs or insulin were used to control blood glucose. Fasting blood glucose was controlled at 5.6-7.2mmol/L and postprandial blood glucose was less than 11.1mmol/L. Blood pressure was controlled below 130/80mmHg with oral calcium antagonists, beta-blockers, vasodilators, and other drugs.<br>On this basis, PGE1 20 ug was given. |
| Fangwenjuan(2016) | On the basis of the control group, HQI 30 ml was added. | Routine treatment: low salt, low fat, high-quality protein diet, blood pressure, blood glucose, diuretics, albumin preparation and other comprehensive treatment.<br>On this basis, PGE1 10 ug was given. |
| Zhangliang(2016) | On the basis of the control group, HQI 30 ml was added. | Routine treatment: routine diet, diabetes education, oral hypoglycemic drugs, insulin and other treatment, maintain random blood glucose < 10mmol/L, fasting blood glucose < 7.2mmol/L.<br>On this basis, PGE1 10 ug was given. |
| Xufei(2018) | On the basis of the control group, HQI 30 ml was added. | Routine treatment: blood glucose control and diabetic diet control.<br>On this basis, PGE1 20 ug was given. |
| Wanghuibin(2009) | On the basis of the control group, SKI 60 ml was added. | Routine treatment: low protein, high-quality protein diet, insulin control fasting blood glucose around 6.0mmol/L, hypertension patients were given antihypertensive drugs to control blood pressure below 130/80 mmHg.<br>On this basis, PGE1 10 ug was given. |
| Pangjialiang(2011) | On the basis of the control group, SKI 60 ml was added. | Routine treatment: Diabetic diet and a high-quality low protein diet were given, and insulin was given to control blood glucose.<br>On this basis, PGE1 10 ug was given. |
| Zhanghong(2013) | On the basis of the control group, SKI 100 ml was added. | Routine treatment: high-quality low protein diet, active treatment to control blood glucose, blood pressure, blood lipids, and anemia correction.<br>On this basis, PGE1 10 ug was given. |
| Dingxuemei(2014) | On the basis of the control group, SKI 100 ml was added. | Routine treatment: all patients were treated with dietary restriction of protein, hypoglycemia, blood pressure, lipid regulation, anemia correction and other treatments.<br>On this basis, PGE1 10 ug was given. |
| Lanchunying(2014) | On the basis of the control group, SKI 60 ml was added. | Routine treatment: Including low salt, low glucose and low protein diet, atorvastatin for lipid-lowering, blood pressure lowering (blood pressure control at 120-150/80-100mmHg), oral hypoglycemic drugs or insulin injection for strict blood glucose control (fasting blood glucose < 7.0mmol/L, 2h postprandial blood glucose < 10.0mmol/L).<br>On this basis, PGE1 10 ug was given. |

| | | |
|---|---|---|
| Meidongdong(2014) | On the basis of the control group, SKI 60 ml was added. | Routine treatment: diabetic diet and high-quality low protein diet, insulin control glucose, Calcium Antagonist to control blood pressure.<br>On this basis, PGE1 10 ug was given. |
| Yinlili(2014) | On the basis of the control group, SKI 100 ml was added. | Routine treatment: All patients were given routine hypoglycemic treatment, diet control, diabetes education and appropriate exercise.<br>On this basis, PGE1 was given. |
| Zhangbailing(2014) | On the basis of the control group, SKI 100 ml was added. | Routine treatment: high quality, low protein, low salt, low fat, low phosphorus diet, control blood pressure, blood glucose, increase islet sensitivity, correct acid-base balance and ion disorder and other basic treatment, maintain defecation 1-2 times a day.<br>On this basis, PGE1 10 ug was given. |
| Zhangyunpin(2017) | On the basis of the control group, SKI 100 ml was added. | Routine treatment includes limiting protein intake, lowering blood glucose, lowering blood pressure and regulating blood lipids according to the patient's condition.<br>On this basis, PGE1 10 ug was given. |
| Quanye(2018) | On the basis of the control group, SKI 60 ml was added. | Routine treatment: hypoglycemic, lipid-lowering, hypotensive and other treatments, while following diet control and moderate exercise.<br>On this basis, PGE1 10 ug was given. |
| Zhengwenwu(2018) | On the basis of the control group, SKI 100 ml was added. | Routine treatment: routine hypoglycemic, hypotensive and lipid regulating treatments were given, and daily protein intake was limited.<br>On this basis, PGE1 10 ug was given. |
| Wangxiaojun(2019) | On the basis of the control group, SKI 100 ml was added. | Routine treatment: All patients were treated with routine treatment such as hypoglycemic, hypotensive and lipid regulation.<br>On this basis, PGE1 10 ug was given. |
| Zhangmailang(2019) | On the basis of the control group, SKI 60 ml was added. | Routine treatment: high-quality low protein diet and diabetic diet, subcutaneous insulin control blood sugar.<br>On this basis, PGE1 10 ug was given. |
| Wenxusheng(2021) | On the basis of the control group, SKI 60 ml was added. | Routine treatment: a diet low in sugar, salt and protein; Oral hypoglycemic drugs or insulin injection to control blood sugar; Daily blood glucose self-measurement, exercise, lipid adjustment, etc.<br>On this basis, PGE1 10 ug was given. |
| Niexing(2022) | On the basis of the control group, SKI 60 ml was added. | Routine treatment: limit protein intake, and give hypoglycemic, lipid, blood pressure and other basic treatment.<br>On this basis, PGE1 10 ug was given. |
| Rujianyong(2008) | On the basis of the control group, SXTI 6 ml was added. | Routine treatment: diabetic diet, oral hypoglycemic drugs or insulin were used to control blood glucose, so that fasting blood glucose was stable at about 7.0mmol/L, and blood pressure was controlled at about 130/80 mmHg.<br>On this basis, PGE1 10 ug was given. |
| Gongyun(2009) | On the basis of the control group, SXTI 6 ml was added. | Routine treatment: the original oral hypoglycemic drugs or insulin should be continued to control blood glucose, with the same diet and activity level.<br>On this basis, PGE1 20 ug was given. |
| Xingyelan(2012) | On the basis of the control group, SXTI 6 ml was added. | Routine treatment: Routine treatment, diabetes health education, high quality low protein diet, moderate exercise and insulin hypoglycemia were given.<br>On this basis, PGE1 100 ug was given. |
| Tiantian(2018) | On the basis of the control group, SXTI 2 ml was added. | Routine treatment: Routine comprehensive treatment such as hypoglycemia, lipid regulation, diet intervention and exercise intervention were given. |

| | | |
|---|---|---|
| | | On this basis, PGE1 20 ug was given. |
| Caoli(2016) | On the basis of the control group, XBJI 10 ml was added. | Routine treatment: including control of blood glucose, blood pressure, blood lipids, low salt, and high quality protein diet, etc.<br>On this basis, PGE1 2 ml was given. |
| Liqing(2016) | On the basis of the control group, XBJI 40 ml was added. | Routine treatment: high quality low-protein diabetic diet, blood glucose and blood pressure control, lipid-lowering and other conventional treatment, oral hypoglycemic drugs or insulin used during the study were unchanged, fasting blood glucose was controlled at 5.6-7.8 mmol/L, and 2h postprandial blood glucose was less than 11.1 mmol/L.<br>On this basis, PGE1 10 ug was given. |
| Jiangqiang(2017) | On the basis of the control group, XBJI 50 ml was added. | Routine treatment: including high-quality low protein diabetic diet, blood glucose and blood pressure control, lipid reduction, avoiding fatigue, staying up late, spicy food stimulation, smoking and drinking, and other bad habits.<br>On this basis, PGE1 20 ug was given. |

SMI: Salvia miltiorrhiza injection, DCI: Danshen-Chuanxiongqin injection, DHI: Danhong injection, HQI: Huangqi injection, SKI: Shenkang injection, SXTI: Shuxuetong injection, XBJI: Xuebijing injection.

## *Supplementary table S15: Comparisons of the fit of consistency and inconsistency models using deviance information criteria (DIC).*

| Total effective rate | Scr | BUN | UAER | 24hAlb | β2-MG |
|---|---|---|---|---|---|
| 68.587 | 89.226 | 80.179 | 49.03 | 47.737 | 30.415 |
| 68.648 | 89.245 | 80.158 | 49.073 | 47.67 | 30.455 |

## ***Supplementary table S16: League table for treatment comparisons in sensitivity analyses by excluding studies that were not within 14 to 30 days of treatment***

| Total effective rate | PGE1 | PGE1plusDCI | PGE1plusDHI | PGE1plusHQI | PGE1plusSKI | PGE1plusSMI | PGE1plusSXTI | PGE1plusXBJI |
|---|---|---|---|---|---|---|---|---|
| PGE1 | PGE1 | **1.16 (1.02, 1.37)** | **1.27 (1.13, 1.46)** | **1.43 (1.26, 1.66)** | **1.19 (1.11, 1.3)** | **1.24 (1.15, 1.35)** | 1.18 (1, 1.42) | **** |
| PGE1plusDCI | 0.86 (0.73, 0.98) | PGE1plusDCI | 1.09 (0.9, 1.33) | **1.23 (1.01, 1.49)** | 1.02 (0.86, 1.21) | 1.06 (0.89, 1.25) | 1.01 (0.8, 1.27) | \\ |
| PGE1plusDHI | 0.79 (0.68, 0.89) | 0.92 (0.75, 1.11) | PGE1plusDHI | 1.13 (0.93, 1.36) | 0.94 (0.8, 1.09) | 0.98 (0.83, 1.12) | 0.93 (0.75, 1.15) | \\ |
| PGE1plusHQI | 0.7 (0.6, 0.79) | 0.81 (0.67, 0.99) | 0.89 (0.74, 1.07) | PGE1plusHQI | 0.83 (0.71, 0.97) | 0.87 (0.73, 1.01) | 0.82 (0.66, 1.03) | \\ |
| PGE1plusSKI | 0.84 (0.77, 0.9) | 0.98 (0.83, 1.16) | 1.07 (0.92, 1.25) | 1.2 (1.03, 1.41) | PGE1plusSKI | 1.04 (0.93, 1.16) | 0.99 (0.82, 1.21) | \\ |
| PGE1plusSMI | 0.81 (0.74, 0.87) | 0.94 (0.8, 1.12) | 1.02 (0.89, 1.21) | 1.15 (0.99, 1.36) | 0.96 (0.86, 1.07) | PGE1plusSMI | 0.95 (0.79, 1.16) | \\ |
| PGE1plusSXTI | 0.85 (0.7, 1) | 0.99 (0.79, 1.24) | 1.08 (0.87, 1.33) | 1.21 (0.97, 1.52) | 1.01 (0.83, 1.21) | 1.05 (0.86, 1.27) | PGE1plusSXTI | \\ |
| PGE1plusXBJI | \\ | \\ | \\ | \\ | \\ | \\ | \\ | PGE1plusXBJI |
| **SCR** | PGE1 | PGE1plusDCI | PGE1plusDHI | PGE1plusHQI | PGE1plusSKI | PGE1plusSMI | PGE1plusSXTI | PGE1plusXBJI |
| PGE1 | PGE1 | **-1.34 (-2.14, -0.54)** | **-0.99 (-1.94, -0.05)** | -0.64 (-1.44, 0.16) | **-1.78 (-2.41, -1.17)** | **-0.83 (-1.62, -0.04)** | -1.19 (-2.69, 0.32) | -1.36 (-2.83, 0.12) |
| PGE1plusDCI | \\ | PGE1plusDCI | 0.34 (-0.89, 1.58) | 0.69 (-0.43, 1.83) | -0.44 (-1.46, 0.56) | 0.51 (-0.62, 1.63) | 0.15 (-1.55, 1.86) | -0.02 (-1.7, 1.65) |
| PGE1plusDHI | \\ | \\ | PGE1plusDHI | 0.35 (-0.89, 1.59) | -0.78 (-1.92, 0.34) | 0.16 (-1.07, 1.39) | -0.2 (-1.97, 1.58) | -0.37 (-2.12, 1.39) |

| | | | | | | | | |
|---|---|---|---|---|---|---|---|---|
| PGE1plusHQI | \ | \ | \ | PGE1plusHQI | **-1.14 (-2.16, -0.13)** | -0.19 (-1.31, 0.94) | -0.55 (-2.25, 1.16) | -0.72 (-2.4, 0.97) |
| PGE1plusSKI | \ | \ | \ | \ | PGE1plusSKI | 0.95 (-0.04, 1.96) | 0.59 (-1.03, 2.23) | 0.42 (-1.17, 2.03) |
| PGE1plusSMI | \ | \ | \ | \ | \ | PGE1plusSMI | -0.36 (-2.06, 1.36) | -0.53 (-2.21, 1.15) |
| PGE1plusSXTI | \ | \ | \ | \ | \ | \ | PGE1plusSXTI | -0.17 (-2.29, 1.93) |
| PGE1plusXBJI | \ | \ | \ | \ | \ | \ | \ | PGE1plusXBJI |

* Significant effects are printed in bold.

## *Supplementary table S17: League table for treatment comparisons in sensitivity analyses by excluding studies that were high risk bias*

| Total effective rate | PGE1 | PGE1plusDCI | PGE1plusDHI | PGE1plusHQI | PGE1plusSKI | PGE1plusSMI | PGE1plusSXTI | PGE1plusXBJI |
|---|---|---|---|---|---|---|---|---|
| PGE1 | PGE1 | **1.17 (1.02, 1.37)** | **1.28 (1.13, 1.47)** | **1.4 (1.21, 1.62)** | **1.19 (1.08, 1.33)** | **1.23 (1.13, 1.37)** | 1.18 (0.99, 1.44) | \ |
| PGE1plusDCI | \ | PGE1plusDCI | 1.1 (0.89, 1.33) | 1.19 (0.97, 1.48) | 1.02 (0.84, 1.22) | 1.06 (0.88, 1.26) | 1.01 (0.79, 1.3) | \ |
| PGE1plusDHI | \ | \ | PGE1plusDHI | 1.09 (0.9, 1.33) | 0.93 (0.78, 1.1) | 0.96 (0.81, 1.14) | 0.92 (0.74, 1.17) | \ |
| PGE1plusHQI | \ | \ | \ | PGE1plusHQI | 0.85 (0.71, 1.02) | 0.89 (0.74, 1.05) | 0.85 (0.67, 1.08) | \ |
| PGE1plusSKI | \ | \ | \ | \ | PGE1plusSKI | 1.04 (0.9, 1.2) | 0.99 (0.81, 1.23) | \ |
| PGE1plusSMI | \ | \ | \ | \ | \ | PGE1plusSMI | 0.96 (0.77, 1.19) | \ |
| PGE1plusSXTI | \ | \ | \ | \ | \ | \\ | PGE1plusSXTI | \ |
| PGE1plusXBJI | \ | \ | \ | \ | \ | \ | \ | PGE1plusXBJI |
| SCR | PGE1 | PGE1plusDCI | PGE1plusDHI | PGE1plusHQI | PGE1plusSKI | PGE1plusSMI | PGE1plusSXTI | PGE1plusXBJI |

| | | | | | | | | | |
|---|---|---|---|---|---|---|---|---|---|
| PGE1 | PGE1 | **-1.34 (-2.06, -0.62)** | **-0.86 (-1.64, -0.08)** | -0.7 (-1.47, 0.08) | **-1.55 (-2.17, -0.95)** | **-0.83 (-1.6, -0.06)** | -0.91 (-2, 0.19) | -1.1 (-2.19, -0.01) |
| PGE1plusDCI | | PGE1plusDCI | 0.48 (-0.58, 1.54) | 0.64 (-0.42, 1.7) | -0.21 (-1.17, 0.72) | 0.51 (-0.54, 1.57) | 0.43 (-0.88, 1.75) | 0.24 (-1.06, 1.55) |
| PGE1plusDHI | | | PGE1plusDHI | 0.16 (-0.93, 1.27) | -0.69 (-1.69, 0.29) | 0.03 (-1.06, 1.13) | -0.04 (-1.39, 1.31) | -0.24 (-1.57, 1.1) |
| PGE1plusHQI | | | | PGE1plusHQI | -0.85 (-1.85, 0.13) | -0.14 (-1.23, 0.97) | -0.21 (-1.55, 1.14) | -0.4 (-1.74, 0.94) |
| PGE1plusSKI | | | | | PGE1plusSKI | 0.72 (-0.25, 1.72) | 0.65 (-0.6, 1.92) | 0.45 (-0.79, 1.71) |
| PGE1plusSMI | | | | | | PGE1plusSMI | -0.08 (-1.42, 1.27) | -0.27 (-1.6, 1.07) |
| PGE1plusSXTI | | | | | | | PGE1plusSXTI | -0.19 (-1.74, 1.36) |
| PGE1plusXBJI | | | | | | | | PGE1plusXBJI |

* Significant effects are printed in bold.

## *Supplementary table S18: The PRISMA checklist of this meta-analysis*

| Section and Topic | Item # | Checklist item | Location where item is reported |
|---|---|---|---|
| **TITLE** | | | |
| Title | 1 | Identify the report as a systematic review. | 1 |
| **ABSTRACT** | | | |
| Abstract | 2 | See the PRISMA 2020 for Abstracts checklist. | 1-2 |
| **INTRODUCTION** | | | |
| Rationale | 3 | Describe the rationale for the review in the context of existing knowledge. | 2-3 |
| Objectives | 4 | Provide an explicit statement of the objective(s) or question(s) the review addresses. | 3 |
| **METHODS** | | | |
| Eligibility criteria | 5 | Specify the inclusion and exclusion criteria for the review and how studies were grouped for the syntheses. | 4 |

| Section and Topic | Item # | Checklist item | Location where item is reported |
|---|---|---|---|
| Information sources | 6 | Specify all databases, registers, websites, organisations, reference lists and other sources searched or consulted to identify studies. Specify the date when each source was last searched or consulted. | 3-4 |
| Search strategy | 7 | Present the full search strategies for all databases, registers and websites, including any filters and limits used. | Supplementary table S3-S10 |
| Selection process | 8 | Specify the methods used to decide whether a study met the inclusion criteria of the review, including how many reviewers screened each record and each report retrieved, whether they worked independently, and if applicable, details of automation tools used in the process. | 4 |
| Data collection process | 9 | Specify the methods used to collect data from reports, including how many reviewers collected data from each report, whether they worked independently, any processes for obtaining or confirming data from study investigators, and if applicable, details of automation tools used in the process. | 4 |
| Data items | 10a | List and define all outcomes for which data were sought. Specify whether all results that were compatible with each outcome domain in each study were sought (e.g. for all measures, time points, analyses), and if not, the methods used to decide which results to collect. | 4 |
| | 10b | List and define all other variables for which data were sought (e.g. participant and intervention characteristics, funding sources). Describe any assumptions made about any missing or unclear information. | 4 |
| Study risk of bias assessment | 11 | Specify the methods used to assess risk of bias in the included studies, including details of the tool(s) used, how many reviewers assessed each study and whether they worked independently, and if applicable, details of automation tools used in the process. | 5 |
| Effect measures | 12 | Specify for each outcome the effect measure(s) (e.g. risk ratio, mean difference) used in the synthesis or presentation of results. | 5 |
| Synthesis methods | 13a | Describe the processes used to decide which studies were eligible for each synthesis (e.g. tabulating the study intervention characteristics and comparing against the planned groups for each synthesis (item #5)). | 5, and Supplementary table S11. |
| | 13b | Describe any methods required to prepare the data for presentation or synthesis, such as handling of missing summary statistics, or data conversions. | 5 |
| | 13c | Describe any methods used to tabulate or visually display results of individual studies and syntheses. | 5 |
| | 13d | Describe any methods used to synthesize results and provide a rationale for the choice(s). If meta-analysis was performed, describe the model(s), method(s) to identify the presence and extent of statistical heterogeneity, and software package(s) used. | 5 |
| | 13e | Describe any methods used to explore possible causes of heterogeneity among study results (e.g. subgroup analysis, meta-regression). | 5 |
| | 13f | Describe any sensitivity analyses conducted to assess robustness of the synthesized results. | 5 |
| Reporting bias assessment | 14 | Describe any methods used to assess risk of bias due to missing results in a synthesis (arising from reporting biases). | 5 |

| Section and Topic | Item # | Checklist item | Location where item is reported |
|---|---|---|---|
| Certainty assessment | 15 | Describe any methods used to assess certainty (or confidence) in the body of evidence for an outcome. | 5-6 |
| **RESULTS** | | | |
| Study selection | 16a | Describe the results of the search and selection process, from the number of records identified in the search to the number of studies included in the review, ideally using a flow diagram. | 6 |
| | 16b | Cite studies that might appear to meet the inclusion criteria, but which were excluded, and explain why they were excluded. | 6 |
| Study characteristics | 17 | Cite each included study and present its characteristics. | 6 |
| Risk of bias in studies | 18 | Present assessments of risk of bias for each included study. | 7 |
| Results of individual studies | 19 | For all outcomes, present, for each study: (a) summary statistics for each group (where appropriate) and (b) an effect estimate and its precision (e.g. confidence/credible interval), ideally using structured tables or plots. | 21-27 |
| Results of syntheses | 20a | For each synthesis, briefly summarise the characteristics and risk of bias among contributing studies. | 7 |
| | 20b | Present results of all statistical syntheses conducted. If meta-analysis was done, present for each the summary estimate and its precision (e.g. confidence/credible interval) and measures of statistical heterogeneity. If comparing groups, describe the direction of the effect. | 8-9 |
| | 20c | Present results of all investigations of possible causes of heterogeneity among study results. | 10 |
| | 20d | Present results of all sensitivity analyses conducted to assess the robustness of the synthesized results. | 9, and Supplementary figure S2-S7 |
| Reporting biases | 21 | Present assessments of risk of bias due to missing results (arising from reporting biases) for each synthesis assessed. | 11 |
| Certainty of evidence | 22 | Present assessments of certainty (or confidence) in the body of evidence for each outcome assessed. | Supplementary Table S1 |
| **DISCUSSION** | | | |
| Discussion | 23a | Provide a general interpretation of the results in the context of other evidence. | 10-13 |
| | 23b | Discuss any limitations of the evidence included in the review. | 13 |
| | 23c | Discuss any limitations of the review processes used. | 13 |
| | 23d | Discuss implications of the results for practice, policy, and future research. | 13 |

| Section and Topic | Item # | Checklist item | Location where item is reported |
|---|---|---|---|
| **OTHER INFORMATION** | | | |
| Registration and protocol | 24a | Provide registration information for the review, including register name and registration number, or state that the review was not registered. | 3 |
| | 24b | Indicate where the review protocol can be accessed, or state that a protocol was not prepared. | 3 |
| | 24c | Describe and explain any amendments to information provided at registration or in the protocol. | 3 |
| Support | 25 | Describe sources of financial or non-financial support for the review, and the role of the funders or sponsors in the review. | 14 |
| Competing interests | 26 | Declare any competing interests of review authors. | 14 |
| Availability of data, code and other materials | 27 | Report which of the following are publicly available and where they can be found: template data collection forms; data extracted from included studies; data used for all analyses; analytic code; any other materials used in the review. | 14 |
